# The positive–negative–competence (PNC) model of psychological responses to representations of robots

**DOI:** 10.1038/s41562-023-01705-7

**Published:** 2023-10-02

**Authors:** Dario Krpan, Jonathan E. Booth, Andreea Damien

**Affiliations:** 1 Department of Psychological and Behavioural Science, London School of Economics and Political Science, London, UK; 2 Department of Management, London School of Economics and Political Science, London, UK

**Keywords:** Human behaviour, Science, technology and society

## Abstract

Robots are becoming an increasingly prominent part of society. Despite their growing importance, there exists no overarching model that synthesizes people’s psychological reactions to robots and identifies what factors shape them. To address this, we created a taxonomy of affective, cognitive and behavioural processes in response to a comprehensive stimulus sample depicting robots from 28 domains of human activity (for example, education, hospitality and industry) and examined its individual difference predictors. Across seven studies that tested 9,274 UK and US participants recruited via online panels, we used a data-driven approach combining qualitative and quantitative techniques to develop the positive–negative–competence model, which categorizes all psychological processes in response to the stimulus sample into three dimensions: positive, negative and competence-related. We also established the main individual difference predictors of these dimensions and examined the mechanisms for each predictor. Overall, this research provides an in-depth understanding of psychological functioning regarding representations of robots.

## Abstract (web summary)

The authors find that psychological responses towards representations of robots fall into three dimensions: positive, negative and competence. They also examine their individual difference predictors.

## Main

Various projections indicate that robots will soon become a constituent part of society and will need to be increasingly integrated into it^1–5^. This trend highlights the importance of understanding people’s psychological processes (for example, feelings, thoughts and actions) towards robots. Indeed, these processes form the basis of human–robot relationships and are therefore likely to shape the dynamics of the new world permeated by robots^6–10^. In this respect, although various processes have been investigated^6^, this research area is still in its infancy for several reasons.

First, scholars have not synthesized psychological processes towards robots into an overarching framework that clarifies how they function as a whole and allows for building theories that would explain them. Second, it is unclear whether, and how many, important psychological processes remain hidden due to the lack of systematic research on this topic. Third, previous studies have mainly focused on specific robot types (for example, social^6^) rather than examining the full content space of robots across all domains of human activity (for example, education, hospitality and industry). Finally, most research has been conducted outside of psychology (for example, healthcare and robotics^6,11,12^). Consequently, there has been little effort to integrate people’s responses to robots with important constructs from psychology in a way that would allow the field to study this topic more systematically and establish a coherent research stream around it.

To address this, the present research has two objectives: (1) to develop an integrative and comprehensive taxonomy of psychological processes in response to robots from all domains of human activity that organizes these processes into dimensions; and (2) to establish which individual differences widely studied in psychology are the most important predictors of these dimensions and to understand the mechanisms behind their relationships.

In this context we use the term ‘psychological processes’ in reference to people’s affective (that is, feelings towards robots), cognitive (that is, thoughts about robots) and behavioural responses (that is, actions towards them). This rule-of-thumb classification is often used to summarize and investigate psychological processes in an all-encompassing way^13–15^, because an official taxonomy does not exist. We adopt it because it is useful as a guiding principle when (1) eliciting diverse psychological processes and (2) identifying and organizing previous literature, considering that psychological functioning involving robots is typically not studied as a uniform construct and comprises studies from numerous areas.

Next, we briefly review previous research on psychological processes regarding robots in terms of affective, cognitive and behavioural responses (for a detailed review see Supplementary Notes). Before this review, we first clarify how we define robots because their definition is often confined to various specific types (for example, autonomous and social^6,16–20^), and describing them as an overarching category can be less straightforward^19–21^.

We adopt a general definition proposed by the Institute of Electrical and Electronics Engineers (IEEE^22^), according to which robots are devices that can act in the physical world to accomplish different tasks and are made of mechanical and electronic parts. These devices can be autonomous or subordinated to humans or software agents acting on behalf of humans. Robots can also form groups (that is, robotic systems) in which they cooperate to accomplish collective goals (for example, car manufacturing).

Previous research has documented diverse affective responses to robots, which can be classified as negative or positive^6^. Regarding negative feelings, fear and anxiety are typically experienced concerning robots taking people’s jobs^23–27^. Individuals can also find robots creepy if they are designed to be human-like but look unnatural and inconsistent with human appearance^28^. Regarding positive feelings, individuals can experience happiness, amazement, amusement, enjoyment, pleasure, warmth and empathy towards robots^6,10,25,26,29–38^. Interestingly, people can also become emotionally attached, feel attracted and be in love with robots^39–44^. Whereas these romantic feelings are perceived by many as taboo, they are becoming increasingly frequent nowadays^42^.

In terms of cognitive responses, people’s thoughts about robots can be organized into several themes. A key theme is the level of competence displayed by robots concerning tasks in which they are specialized^19,30,45,46^. For example, robots are often seen as efficient and accurate in what they do and as more physically endurant than humans^19,47,48^. Individuals can also consider robots helpful and appreciate their effectiveness in accomplishing various tasks, from household chores to carrying heavy loads^49–52^. Another important theme is anthropomorphism (that is, ascribing human characteristics to non-living entities^7^). For instance, people may perceive robots as sentient beings that have feelings^53–59^ but they may also see them as distinct from humans (for example, cold or soulless^19,60–62^) and question whether robots can be trusted in their capacities as companions, coworkers and other roles they assume^63–65^.

In terms of behavioural responses, actions towards robots can be classified as either approach (for example, engaging with them) or avoidance (for example, evading them)^10,66–68^. Common approach behaviours involve communicating, cooperating, playing and requesting information^10,26,69,70^. More negative approach behaviours have also been documented, including several instances of robot abuse^71–73^. In contrast to approach, avoidance behaviours (for example, hiding from robots) are infrequently mentioned in the literature and may typically occur in environments where robots could potentially injure humans^74,75^.

Overall, the reviewed literature indicates that various psychological responses to robots have been observed. However, because this topic is not studied under a common umbrella of psychological processes but in relation to diverse topics (for example, anthropomorphism, robotic job replacement or robot acceptance^7,49,76^), it is unclear how these processes are interlinked, what shapes them and whether all important processes have been discovered.

For these reasons, our research adopted a data-driven rather than a theory-driven approach^77–79^. Contrary to theory-driven studies that are inherently deductive because they test hypotheses deduced from general principles (that is, theory), data-driven research is inductive because it starts with empirical observations that are not guided by hypotheses and can progressively evolve into theory^77–82^.

A data-driven approach is recommended if (1) a construct is in its early stages of development and/or (2) its theoretical foundations have not been established^77–80,82,83^. Based on this, a data-driven approach is optimal for our research for both reasons. First, as previously indicated, the conceptual bases of our topic are at an early stage because different affective, cognitive and behavioural responses to robots have not been studied under an all-encompassing construct (that is, psychological processes). Second, theoretical foundations have not yet been developed, because encapsulating the entirety of psychological functioning regarding robots by identifying, organizing and predicting the psychological processes triggered by robots is beyond the scope of existing models of human–technology relationships. To illustrate this, the technology acceptance model^84–86^ and its extensions—the unified theory of acceptance and use of technology^87–89^ and the Almere model^90^—examine the factors that make people accept technology (for example, perceived usefulness, ease of use or social influence) whereas the media equation^91–93^ examines whether people interact with media (for example, computers) similarly to how they interact with other humans.

Data-driven approaches have three main benefits. First, they allow the study of novel topics without engaging in premature theorizing that can lead to post hoc hypothesizing and false-positive findings^77,78,94–97^. Second, because the emphasis is on inferences from data that are not constrained by previous theories and findings, these approaches can diversify knowledge of human psychology and spark unexpected insights^79,81,98^. Third, they can be more beneficial to previous research on the topic than deductive approaches directly informed by this research. In behavioural sciences, failed replications are common and researchers examining the same research questions and hypotheses, even with identical data, can often obtain different findings^99–103^. Therefore, if a data-driven study produces a finding consistent with previous research and theorizing, despite using a methodological approach that is solely guided by data and not constrained by their assumptions, this is a compelling case of support for the previous work. It is thus important to emphasize that using a data-driven approach does not imply conducting a research project that disregards previous literature. Quite the contrary, it is essential to comprehensively evaluate and discuss how the findings are linked to previous work to illuminate how the present research has extended this work and moved the field forward—a process labelled inductive integration^77^.

**Fig. 1:**
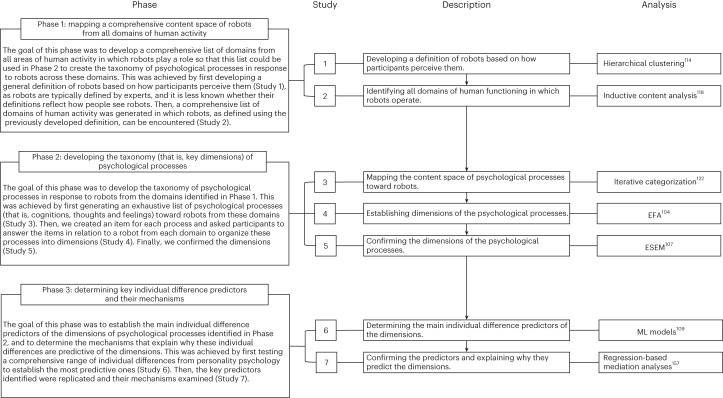
Overview of the present research. ‘Phase’ outlines the goals of each research phase, how these goals were achieved and the link between successive phases. ‘Study’ and ‘description’ indicate the number of each study and its goal while ‘analysis’ specifies the statistical analyses that were used in each study.

Drawing on data-driven approaches, our research objectives—(1) establishing a taxonomy of psychological processes involving robots and (2) examining its individual difference predictors—are achieved in three phases comprising seven studies (Fig. 1; for participant information see Table 1).

**Table 1:** Sample size and background information for all participants who completed a study, and for those participants included in analyses (Studies 1–7)

Study no.	Sample no.	Sample size	Country	Age (years)		Gender				Employment status			Use of robots at work			
				Mean	s.d.	Female	Male	Other	UD^a^	Employed	Unemployed	UD^a^	Don’t know	No	Yes	UD^a^
All participants																
1	1	266	UK	49.496	13.598	132	133	1	0	175	91	0	3	161	11	0
						49.62%	50.00%	0.38%	0%	65.79%	34.21%	0%	1.71%	92.00%	6.29%	0%
1	2	100	US	36.510	10.566	42	58	0	0	94	6	0	2	90	2	0
						42.00%	58.00%	0%	0%	94.00%	6.00%	0%	2.13%	95.74%	2.13%	0%
2	−	70	US	36.257	10.270	31	39	0	0	64	6	0	1	55	8	0
						44.29%	55.71%	0%	0%	91.43%	8.57%	0%	1.56%	85.94%	12.50%	0%
3	−	350	US	40.693	12.194	193	153	1	3	325	24	1	5	279	41	0
						55.14%	43.71%	0.29%	0.86%	92.86%	6.86%	0.29%	1.54%	85.85%	12.62%	0%
4	1	1,668	UK	47.932	16.611	852	812	4	0	1,043	624	1	13	955	75	0
						51.08%	48.68%	0.24%	0%	62.53%	37.41%	0.06%	1.25%	91.56%	7.19%	0%
4	2	1,808	US	48.004	16.772	976	830	2	0	1,053	754	1	14	871	168	0
						53.98%	45.91%	0.11%	0%	58.24%	41.70%	0.06%	1.33%	82.72%	15.95%	0%
5	1	1,200	UK	46.648	16.616	590	601	6	3	753	447	0	14	690	49	0
						49.17%	50.08%	0.50%	0.25%	62.75%	37.25%	0%	1.86%	91.63%	6.51%	0%
5	2	1,219	US	46.656	16.914	616	598	5	0	712	506	1	12	639	61	0
						50.53%	49.06%	0.41%	0%	58.41%	41.51%	0.08%	1.69%	89.75%	8.57%	0%
6	−	2,505	US	47.405	17.262	1,299	1,186	15	5	1,537	964	4	19	1,210	307	1
						51.86%	47.35%	0.60%	0.20%	61.36%	38.48%	0.16%	1.24%	78.72%	19.97%	0.07%
7	−	1,116	US	42.910	13.535	552	555	9	0	843	273	0	22	754	66	1
						49.46%	49.73%	0.81%	0%	75.54%	24.46%	0%	2.61%	89.44%	7.83%	0.12%
Participants included in analyses																
1	1	224	UK	50.344	13.262	121	102	1	0	145	79	0	3	136	6	0
						54.02%	45.54%	0.45%	0%	64.73%	35.27%	0%	2.07%	93.79%	4.14%	0%
1	2	95	US	36.621	10.729	39	56	0	0	91	4	0	2	88	1	0
						41.05%	58.95%	0%	0%	95.79%	4.21%	0%	2.20%	96.70%	1.10%	0%
2	–	67	US	35.657	9.634	31	36	0	0	61	6	0	1	52	8	0
						46.27%	53.73%	0%	0%	91.04%	8.96%	0%	1.64%	85.25%	13.11%	0%
3	–	334	US	40.826	12.154	184	147	1	2	311	22	1	5	270	36	0
						55.09%	44.01%	0.30%	0.60%	93.11%	6.59%	0.30%	1.61%	86.82%	11.58%	0%
4	1	1,528	UK	48.328	16.515	790	734	4	0	944	583	1	12	874	58	0
						51.70%	48.04%	0.26%	0%	61.78%	38.15%	0.07%	1.27%	92.58%	6.14%	0%
4	2	1,537	US	49.465	16.563	861	674	2	0	870	667	0	11	745	114	0
						56.02%	43.85%	0.13%	0%	56.60%	43.40%	0%	1.26%	85.63%	13.10%	0%
5	1	1,107	UK	47.112	16.583	544	555	6	2	691	416	0	10	639	42	0
						49.14%	50.14%	0.54%	0.18%	62.42%	37.58%	0%	1.45%	92.47%	6.08%	0%
5	2	1,108	US	47.100	16.947	563	540	5	0	651	456	1	12	591	48	0
						50.81%	48.74%	0.45%	0%	58.75%	41.16%	0.09%	1.84%	90.78%	7.37%	0%
6	–	2,203	US	47.947	17.493	1,164	1,021	14	4	1,316	883	4	16	1,064	235	1
						52.84%	46.35%	0.64%	0.18%	59.74%	40.08%	0.18%	1.22%	80.85%	17.86%	0.08%
7	–	1,071	US	42.846	13.450	535	527	9	0	808	263	0	22	721	64	1
						49.95%	49.21%	0.84%	0%	75.44%	24.56%	0%	2.72%	89.23%	7.92%	0.12%

All studies were administered via Qualtrics. In Studies 1 (Sample 1), 4 (Samples 1 and 2), 5 (Samples 1 and 2) and 6, participants were recruited via Pureprofile; in Studies 1 (Sample 2), 2 and 3, participants were recruited via Amazon Mechanical Turk; in Study 7, participants were recruited via Prolific.

Samples for Studies 4–6 were recruited to be reasonably representative of the UK/US populations in terms of age, gender and geographical region, whereas for Study 1 (Sample 1) the focus was on gender only. Supplementary Tables 1 and 2 contain more comprehensive breakdowns of these variables, the criteria that were used to guide representative sampling and additional demographic characteristics.

^a^UD, undisclosed: for gender, this category comprises participants who either selected the option ‘choose not to disclose’ or whose data were missing; for other variables, this category comprises participants whose data were missing. For employment status, the category ‘employed’ comprises participants who were either self-employed or working for an employer whereas ‘unemployed’ refers to participants who were not working for themselves or someone else.

Phase 1 consisted of two studies that undertook an in-depth examination of the construct of robots that was necessary to build the taxonomy. In Study 1 we developed an all-encompassing general definition of robots. In Study 2 we used this definition to identify all domains of human activity in which robots operate.

Phase 2 consisted of three studies aimed at creating the taxonomy. In Study 3 we sampled a comprehensive content space of people’s psychological processes involving robots across the domains identified in Phase 1 to develop items assessing each process. In Study 4 we determined the main dimensions of these processes using exploratory factor analyses (EFAs^104–106^). In Study 5 we further confirmed these dimensions using exploratory structural equation modelling (ESEM^107,108^) and developed the psychological responses to robots (PRR) scale that can assess psychological processes towards any robot.

Phase 3 consisted of two studies that focused on determining the most important individual difference predictors of the psychological responses and testing the mechanisms behind these relationships. In Study 6 we used machine learning^109,110^ to identify the key predictors of the main dimensions of the PRR scale. In Study 7 we probed the mechanisms behind these predictors.

All in all, to achieve our research objectives, as stimuli we used representations (that is, images and descriptions) of robots (Supplementary Table 7) from 28 exhaustive domains of human activity in which robots operate (Table 2). This comprehensive approach allowed us to minimize the chance that our findings are driven by idiosyncrasies of a sample that is small in size and/or variety of robot types, which could compromise replicability^111,112^. Despite the wide variety of our stimulus sample, it is unclear to what degree this sample is representative of the general population of robots because (1) there are no established recommendations on what variables would need to be measured to accurately define this population, (2) the type of data used to quantify general characteristics of human populations is not available for robots and (3) the field of robotics is rapidly evolving. Therefore, in the context of our research we use the term ‘robot/s’ in reference to our specific stimulus sample and we do not imply that our insights extend to the general population (that is, all physical robots).

**Table 2:** Definition of robots developed from the clusters of their characteristics generated by participants (Study 1), and robot domains grounded in this definition (Study 2), with example participant items analysed using indictive content analysis to develop the domains

Definition part	Definition	Robot clusters that informed the definition^a^
1	A robot is a non-living entity that primarily functions as helping and/or substituting humans in some capacity by performing physical and/or intellectual tasks that range from simple, routine to complex ones.	1, 4, 5
2	Robots are characterized by different degrees of autonomy: sometimes they only follow commands that have been pre-programmed, but sometimes they are artificially intelligent and thus are able to learn from the environment and adapt to it.	1
3	Although robots at times require maintenance and repair, they have potential to work tirelessly over long periods of time as they do not have life commitments (e.g., family) and/or wellbeing considerations as humans do (e.g., time off, sick leave).	1
4	Humans can perceive robots as having positive attributes (e.g., clever, consistent, cute, efficient, flexible, friendly, reliable, robust, safe, supportive). However, humans also can have negative perceptions about robots, such as seeing them as cold, creepy, emotionless, lacking conscience, soulless, threatening, etc., generally attributing negative qualities to robots as a result of their nonhuman nature.	2, 4
5	A robot typically consists of software (i.e., the code or programme on which it runs) and different materials and components used to produce it (e.g., metal, wires, sensors, microchips, etc.). Although robots can take the form and/or have characteristics of humans, they can appear as an animal or any non-living object.	1, 3
Domain no.	Domain	Example items
1	Health and human care and wellbeing (e.g., medical, surgical, fitness, lab diagnostics, elderly, disability, infant/child, and personal care)	Elder care in home; at the doctor or hospital; exercise
2	Social and companionship	Companionship; social life
3	Sex (this domain was added based on ref. ^17^ and was not generated from participants’ responses)	−
4	Animal care (e.g., walking pets)	Animal care; walking pets
5	Security and surveillance	Public safety; security
6	Policing and military	Policing robots; warfare
7	Education, libraries, and knowledge/information management and gathering	Library; studying
8	Research and exploration within science, technology, engineering, and mathematics (STEM) (e.g., ocean exploration, supercomputing, IT innovation, space discovery)	Research/exploration
9	Communication tools and channels	Chatbots; communications
10	Leisure, recreation, and travel	Travel; watercraft
11	Culture/entertainment, gaming, toys, and other amusement	Concerts; entertainment
12	Workplace domain (i.e., to aid or replace human effort)	Work product generation
13	Dangerous and/or risky work	Do dangerous work
14	Inspection, repair and/or improvement of products, engines, equipment, technology, and/or infrastructure (e.g., buildings, bridges, roads, power supplies, nuclear reactors, pipes, gas mains)	Repairs; auto maintenance
15	Agriculture (e.g., harvesting, farms)	Agriculture; farms
16	Household chores/tasks and domestic help/assistance (i.e., inside and outside of the home)	Home; house chores
17	Industry	Factory/factories
18	Hospitality and food service (i.e., hotels, conventions, restaurants, bars, and other lodging, space, food and/or drink provider) and related customer service and support	Eating out; hospitality
19	Banking/financial services and related customer service and support	ATM; bank; business
20	Retail and commerce and related customer service and support	Retail; self-checkout
21	Construction	Construction; demolition
22	Manufacturing	Manufacturing facilities
23	Mining	Mining
24	Warehouses and fulfilment centres	Warehouse work
25	Public services (e.g., road work and other shared public good)	Roadwork
26	Transportation (i.e., land, water, and/or sky) of goods, people, and other living entities, transport equipment, and delivery/courier/shipping services	Drone; transport
27	Airports	Airports
28	Art (this domain was added based on ref. ^18^ and was not generated from participants’ responses)	−

Our aim was to develop domains that are narrow rather than broad, which means that some overlap between them may be present. This approach was aligned with our objective to establish a comprehensive content space of all robots to decrease the probability of using a biased stimulus sample^111,112^ when developing the taxonomy of psychological responses to representations of robots. For that reason, it was more optimal to lean towards having too many rather than too few domains, to reduce the chance of failing to cover the content space of all robots in detail and omitting important types of robots.

^a^Cluster 1, characteristics conveying the degree of robot–human similarity; Cluster 2, positive characteristics; Cluster 3, characteristics conveying robots’ composition; Cluster 4, negative characteristics; and Cluster 5, characteristics conveying robots’ ability to perform various tasks (Supplementary Table 3).

## Results

In this section we briefly present the results (for a detailed description see Supplementary Results).

### Phase 1: mapping a comprehensive content space of robots

Phase 1 aimed to establish a comprehensive content space that encompasses a wide range of robots by identifying all domains of human activity in which robots operate, to ensure that our taxonomy developed in Phase 2 is not biased towards only a few robot types^111,112^.

The first step in this endeavour was to devise a general definition of robots in Study 1, because robot definitions are typically proposed by experts^6,21,22,113^ and it is less well known whether these reflect how people more broadly perceive robots. Because any robot definition is essentially a set of characteristics that describe robots (for example, made of mechanical parts, autonomous^6,22,113^), to develop a general definition we first recruited Sample 1 and asked them to generate robot characteristics. Using this approach, 277 characteristics were identified (Supplementary Table 3). We then recruited Sample 2 and asked them to group these characteristics into common categories. Using hierarchical cluster analysis^114–116^, the following main clusters of robot characteristics were identified: (1) characteristics conveying the degree of robot–human similarity; (2) positive characteristics; (3) characteristics conveying robots’ composition; (4) negative characteristics; and (5) characteristics conveying robots’ ability to perform various tasks (Supplementary Table 3).

The general definition of robots that we subsequently developed by linking the themes of each cluster is available in Table 2. It is important to emphasize that we did not form the definition by always translating an individual cluster theme into a separate part, because the definition was more succinct and coherent if certain themes were combined in the same parts.

In Study 2 we used this robot definition to identify a comprehensive list of domains in which robots operate. Participants were presented with the definition and asked to generate all such domains they could think of. To develop an extensive inventory of domains, we analysed their responses using inductive content analysis^117–121^. Additionally, to ensure we did not miss any domains that participants were unable to identify, we consulted various other resources (for example, articles from the literature review of this paper and classifications detailed in Methods). The final list of domains, accompanied by the example items generated by participants, is available in Table 2.

### Phase 2: creating the taxonomy of psychological processes

To develop the taxonomy, it was first necessary to identify a comprehensive range of psychological processes involving robots in Study 3. We instructed participants to write about any feelings, thoughts and behaviours they could think of concerning robots from the domains developed in Study 2 (Table 2)—each participant was randomly allocated to one of five domains. Participants were not provided with specific robot examples for a given domain, because we expected that reliance on their own reflections and experiences would cover a broader spectrum of robots and therefore increase the diversity of psychological processes reported (for a similar methodological approach see ref. ^82^). Table 3 contains the final list of psychological processes derived from participants’ responses using iterative categorization^122^.

**Table 3:** Summary of key findings (Studies 3 and 4): psychological processes, items corresponding to each process and the output of EFAs performed on the items across two participant samples

Item no.	Psychological process	Item	Sample 1 (UK)			Sample 2 (US)		
			P	N	C	P	N	C
61	Companionship	This robot would make a good companion.	0.853			0.864		
84	Enjoyment	**I associate this robot with enjoyment**.	0.819			0.815		
85	Humour	This robot is humorous.	0.806			0.711		
35	Attachment	**I would feel attached to this robot**.	0.793			0.864		
26	Treating the robot like a human	I would treat this robot as if it were a human.	0.779			0.814		
34	Intimacy	I would be able to connect on an intimate level with this robot (e.g., share feelings, be in close contact, hug or hold, etc.).	0.775			0.842		
60	Comfort	This robot is comforting.	0.772			0.792		
65	Friendliness	This robot is friendly.	0.763			0.764		
116	Empathy	**This robot is empathetic**.	0.753			0.751		
88	Thoughtfulness	**This robot is thoughtful**.	0.745			0.793		
122	Entertainment	This robot is entertaining.	0.724			0.700		
123	Play	I would like to play with this robot.	0.715			0.742		
62	Interaction	I would want to interact with this robot.	0.693			0.663		
40	Engagement	**I would like to engage with this robot**.	0.692			0.689		
32	Happiness	**This robot makes me feel happy**.	0.681			0.806		
41	Motivation	This robot motivates me.	0.681			0.796		
27	Perceiving the robot as human-like	**This robot is like a human**.	0.675			0.743		
63	Communication	**I would find it easy to communicate with this robot**.	0.642			0.685		
67	Wellbeing	This robot promotes wellbeing.	0.635			0.685		
24	Anthropomorphism	I can see human traits in this robot.	0.625			0.681		
77	Pleasantness	I find this robot pleasant.	0.623			0.689		
149	Robot rights	**I think this robot should have rights**.	0.609			0.711		
95	Relaxation	This robot makes me feel relaxed.	0.608			0.742		
66	Care	**This robot provides care**.	0.600			0.675		
132	Self-improvement	**This robot helps me to improve myself**.	0.597			0.773		
14	Excitement	This robot makes me feel excited.	0.590			0.734		
125	Creativity	**This robot is creative**.	0.573			0.615		
89	Learning from robots	I could learn from this robot.	0.567			0.648		
107	Empowerment	**I feel empowered by this robot**.	0.545			0.691		
47	Pride	I feel proud about this robot.	0.541		0.343	0.691		
64	Social support	This robot provides support to me.	0.534			0.633		
129	Competition	I would want to compete with this robot.	0.532	0.420		0.496	0.471	
96	Gratitude	**This robot makes me feel grateful**.	0.526			0.683		
99	Gaining knowledge about robot	I would want to learn more about this robot.	0.504			0.552		
46	Hope	This robot makes me feel hopeful.	0.501		0.371	0.669		
57	Safety	This robot makes me feel safe.	0.499			0.641		
42	Admiration	I admire this robot.	0.495		0.333	0.704		
83	Surprise	This robot surprises me.	0.495			0.531		
39	Interest	I would be interested in this robot.	0.486			0.572		
17	Awareness	**This robot has awareness**.	0.482			0.576		
143	Liberation	This robot makes me feel liberated or free.	0.481		0.324	0.686		
68	Cooperation	This robot and I could cooperate.	0.457			0.490		0.330
98	Information search	This robot provides me information.	0.456			0.479		
86	Artificial intelligence	This robot is intelligent.	0.421			0.511		
9	Need fulfilment	This robot fulfils my needs.	0.409		0.383	0.659		
146	Testing the robot	I would experiment with or test this robot to see what it can do.	0.402			0.399		
71	Openness	I would be open to this robot.	0.390	−0.348		0.450		0.323
87	Learning	This robot can learn.	0.382			0.437		
130	Winning	I want to beat or outperform this robot.	0.378	0.481			0.590	
100	Being knowledgeable about robot	I am knowledgeable about this robot.	0.364			0.483		
82	Positive affect	I feel positive about this robot.	0.359	−0.424	0.366	0.519		0.342
45	Uniqueness	This robot is unique.	0.354			0.335		
18	Ignoring	I would ignore this robot.	−0.324	0.352		−0.379	0.435	
19	Monotony	This robot deals with monotonous and repetitive tasks.	−0.362		0.623			0.559
38	Indifference	I am indifferent toward this robot.	−0.388			−0.344		
29	Instrumentality	This robot is just a means to an end.	−0.435		0.329	−0.412		0.374
28	Objectification	This robot is merely an object.	−0.464			−0.554		
37	Emotionless (human)	I feel no emotions toward this robot.	−0.533			−0.573		
23	Not human	This robot does not feel or respond like humans.	−0.547			−0.614		0.402
36	Emotionless (robot)	This robot is emotionless.	−0.715		0.384	−0.662		0.473
13	Anxiety	**This robot makes me feel anxious**.		0.806			0.693	
59	Threat	**I feel threatened by this robot**.		0.804			0.755	
119	Self-doubt	This robot makes me feel insecure (or doubt myself).		0.793			0.722	
49	Being upset	This robot upsets me.		0.772			0.746	
12	Stress	This robot makes me feel stressed.		0.769			0.731	
102	Fear	I am afraid of this robot.		0.763			0.723	
78	Unpleasantness	This robot makes me feel unpleasant.		0.745			0.749	
25	Dehumanization	**I would feel dehumanized when interacting with this robot**.		0.742			0.708	
50	Anger	This robot angers me.		0.727			0.753	
103	Creepiness	This robot is creepy.		0.720			0.690	
114	Freedom restriction	**This robot restricts or limits me**.		0.718			0.698	
30	Sadness	This robot makes me feel sad.		0.714			0.717	
58	Danger	This robot is dangerous.		0.710			0.672	
111	Boycott	I would ban the use of this robot.		0.701			0.722	
72	Negative affect	**I feel negative toward this robot**.		0.686			0.699	
117	Insignificance	This robot can make humans feel insignificant or not needed.		0.685	0.441		0.589	0.354
140	Disconnection	This robot disconnects humans from one another.		0.675			0.630	
135	Confusion	**This robot makes me feel confused**.		0.671			0.710	
136	People judging the use of robots	I think using this robot is wrong.		0.670			0.727	
33	Loneliness	This robot makes me feel lonely.		0.669			0.671	
115	Societal issues	**This robot can have negative social implications**.		0.661			0.614	
81	Dissatisfaction	This robot brings me dissatisfaction.		0.660			0.697	
92	Being gross	This robot is gross.		0.660			0.682	
148	Existential questioning	This robot makes me question life and existence.		0.659		0.352	0.631	
113	Immorality	**This robot is immoral**.		0.657			0.657	
126	Privacy	**This robot violates privacy (e.g., is too intrusive or invasive)**.		0.656			0.693	
104	Humans lacking control	**I would lack or lose control when using or interacting with this robot**.		0.655			0.650	
76	Abnormal	**This robot is abnormal**.		0.645			0.610	
91	Disgust	**This robot is disgusting**.		0.641			0.695	
48	Damage reputation	**This robot could damage my reputation**.		0.637			0.671	
79	Hate	I would hate dealing with this robot.		0.636			0.651	
147	Human being tired of robot	This robot makes me feel tired or exhausted.		0.631			0.688	
11	Avoidance	I would avoid this robot.		0.630			0.604	
52	Protection of self	**I would want to protect myself when interacting with this robot**.		0.612			0.585	
134	Mixed feelings	I would have mixed feelings toward this robot.		0.607			0.628	
118	Replacement	This robot can make humans feel replaced.		0.600	0.501		0.533	0.418
74	Shyness	I would feel shy around this robot.		0.599			0.643	
31	Guilt	This robot makes me feel guilty.		0.594			0.638	
145	Embarrassment	**I would feel embarrassed or ashamed if I had to interact with this robot**.		0.579			0.719	
142	Robots contribute to human degeneration	**This robot can contribute to human degeneration (e.g., make people become lazy, use less of their mental and physical capacity, etc.)**.		0.570			0.559	
1	Impatience	I would feel impatient when interacting with this robot.		0.559			0.647	
105	Unpredictability	This robot is unpredictable.		0.558			0.549	
51	Redundancy	This robot will make human jobs redundant.		0.536	0.535		0.490	0.380
21	Robot damage	I would be inclined to harm or damage this robot.		0.535		0.320	0.633	
112	Unethical activities	This robot could be used for unethical activities.		0.528			0.478	
73	Disappointment	This robot is disappointing.		0.525	−0.342		0.663	
16	Caution	I would be cautious or careful with this robot.		0.499			0.381	
22	Verbal abuse of robots	I would likely be verbally abusive toward this robot.		0.483			0.632	
139	Dependence (on robots or technology)	This robot creates dependence in humans.		0.447	0.388		0.416	
120	Human interaction substitute	This robot substitutes human interaction.		0.429			0.336	
138	Uselessness	This robot is useless.		0.405	−0.564		0.600	−0.348
131	Social comparison	I compare whether this robot is better than humans.		0.345	0.400		0.368	
133	Boredom	This robot is boring.		0.343			0.478	
3	Inefficiency	This robot is inefficient in what it does.		0.330	−0.410		0.484	
144	Authentic self	I can be my authentic self around this robot.		−0.373				0.374
10	Confidence	I would feel confident in this robot.		−0.375	0.466	0.412		0.426
80	Satisfaction	I am satisfied with this robot.		−0.379	0.403	0.483		0.371
69	Coexistence	I could coexist with this robot.		−0.381				
121	Trust	I would trust this robot.		−0.393	0.342	0.523		
70	Acceptance	I would be accepting of this robot.		−0.491	0.333	0.429	−0.343	0.372
4	Performance	**This robot can effectively achieve a certain result or a specified outcome**.			0.734			0.669
5	Usefulness	This robot is useful.			0.703			0.603
6	Help	This robot is helpful.			0.662			0.592
7	Accuracy	**This robot is accurate in what it does**.			0.642			0.570
2	Complexity	**This robot can do complex tasks**.			0.641			0.484
8	Financial costs	**This robot reduces costs**.			0.632			0.545
54	Speed	**This robot is fast at what it does**.			0.605			0.527
93	Future orientation	I think this robot is the future.			0.603			0.488
94	Progress	I associate this robot with progress.			0.583			0.517
109	Social good	This robot is a benefit to society.			0.545	0.424		0.400
128	Time freedom	This robot frees up my time to do other things.			0.531	0.375		0.417
55	Level of advancement	**This robot is advanced**.			0.516			0.526
127	Easier life	This robot makes my life easier.			0.516	0.478		0.392
43	Being impressed	This robot impresses me.			0.510	0.412		0.409
110	Corporate social responsibility (CSR)	Any benefits gained from this robot should be shared with or passed onto society.			0.504			0.428
141	Robots augment human capabilities	This robot can augment human capabilities.			0.471			0.424
20	Endurance	This robot has endurance (e.g., never tires, runs nonstop, etc.).			0.462			0.580
101	Monitoring	I would monitor this robot to make sure it functions properly.			0.426			0.389
15	Human alertness	I would feel alert with this robot.			0.398			0.397
97	Bias	This robot is not biased.			0.387			0.440
53	Robot superiority	This robot is superior to humans.			0.363	0.353		
44	Novelty	This robot is novel.						
56	Human superiority	Whenever I am given a choice, I will choose a human over this robot.						
75	Unusualness	This robot is unusual.						
90	Cleanliness	I would find this robot sanitary.						0.360
106	Dominance	I am dominant over this robot.						
108	Humans having control	I would have control over this robot.						
124	Autonomy	This robot is autonomous.						
137	Objectivity	This robot is objective.						
		Variance explained (%)	15.995	17.891	10.422	20.736	17.224	8.586
		Eigenvalues	23.832	26.658	15.529	30.897	25.663	12.793
		P	−			−		
		N	−0.192	−		−0.192	−	
		C	0.471	−0.417	−	0.470	−0.245	−

P, N and C refer to the dimensions (that is, factors) that comprise positive, negative and competence-related psychological processes, respectively, regarding robots. Values under each factor correspond to standardized factor loadings; only loadings with absolute values ≥0.320 are reported for clarity. The psychological processes and corresponding items are ordered according to item loadings on the three factors, while item no. corresponds to the number they were assigned when they were created. Items in bold were those selected for the PRR scale tested in Study 5 (Table 4). Coefficients for factors P, N and C at the bottom of the table denote correlations between factors. All items were scored on a seven-point Likert scale (1, strongly disagree; 7, strongly agree).

In Study 4 we then created items for each of these processes (Table 3) and asked participants to answer the items about an example of a robot (Supplementary Table 7) from one of the 28 domains (Table 2) to which they were randomly allocated. To develop the taxonomy from participants’ responses we used maximum-likelihood EFAs^104^ with Kaiser^123^ normalized promax rotation^106,124^. EFAs were appropriate because the Kaiser–Meyer–Olkin measure of sampling adequacy was 0.983 and 0.984 for Samples 1 and 2, respectively, and Bartlett’s test of sphericity was significant (for both samples, *P* < 0.001)^125^.

To select the most appropriate factor solution we used the following procedure. We first consulted parallel analysis^126,127^, very simple structure^128^, Velicer map^129^, optimal coordinates^130^, acceleration factor^130^, Kaiser rule^131^ and visual inspection of scree plots^132^, which indicated that extraction of between one and 19 factors (Sample 1) and between two and 18 factors (Sample 2) could be optimal. Next, we evaluated the largest factor solutions (that is, 19 factors for Sample 1 and 18 for Sample 2) against several statistical and semantic benchmarks. If the benchmarks were not met we decreased the number of factors by one and evaluated these new solutions. This procedure was continued until the benchmarks were met. Concerning statistical benchmarks, a factor solution was required to produce only valid factors—those that have at least three items with standardized loadings ≥0.5 and cross-loadings of <0.32 (refs. ^105,125,133,134^). Semantically, a solution was required to make sense conceptually by having factors that are coherent and easy to interpret^135,136^.

For Samples 1 and 2, three-factor solutions emerged as the most optimal. These met the statistical criteria and had semantically coherent factors that denoted positive, negative and competence-related psychological processes (Table 3). Therefore, the taxonomy was labelled the positive–negative–competence (PNC) model of psychological processes regarding robots. None of the larger factor solutions met the statistical criteria.

We aimed to further validate the PNC model by confirming its dimensions and thereby developing the PRR scale that measures them. To do this, in Study 4 we selected a representative subset of PNC items (bold items in Table 3) and subjected them to ESEM^107^ using the maximum-likelihood with robust standard errors (MLR) estimator^137,138^ and target rotation with all cross-loadings as targets of zero^139,140^. For both samples, fit indices showed good to excellent fit (that is, SRMR < 0.05, CFI > 0.90, RMSEA < 0.06 (refs. ^141–143^)): Sample 1, *χ*^2^(558) = 1,953.820, *P* <0.001, SRMR = 0.026, CFI = 0.939, RMSEA = 0.041, 90% confidence interval (CI) [0.039, 0.043]; Sample 2: *χ*^2^(558) = 1,850.880, *P* <0.001, SRMR = 0.025, CFI = 0.944, RMSEA = 0.039, 90% CI [0.037, 0.041].

Subsequently, in Study 5 we recruited two additional samples and asked participants to answer these items about one of the two robot examples (Supplementary Table 7) from one of the 28 domains to which participants were randomly allocated. The ESEM models for both samples had a good to excellent fit (Table 4). Moreover, items previously classified under a specific dimension (that is, positive, negative or competence) by EFAs in Study 4 (Table 3) had the highest loadings for this dimension whereas the cross-loadings were <0.32. To ensure that the model comprising the three dimensions was the most appropriate we tested several alternative models, which were all rejected due to poor fit (Supplementary Results).

**Table 4:** ESEMs of the PRR scale (Study 5)

Item no.	Sample 1 (UK)			Sample 2 (US)		
	P	N	C	P	N	C
116	0.801			0.756		
132	0.760			0.746		
88	0.758			0.769		
35	0.742			0.756		
84	0.740			0.789		
32	0.682			0.729		
40	0.674			0.588		
149	0.672			0.654		
27	0.658			0.662		
96	0.655			0.626		
107	0.644			0.638		
66	0.587			0.604		
63	0.561			0.536		
125	0.557			0.523		
17	0.546			0.496		
59		0.799			0.816	
113		0.756			0.656	
25		0.750			0.787	
114		0.746			0.724	
13		0.733			0.785	
48		0.723			0.709	
145		0.719			0.695	
135		0.715			0.713	
126		0.709			0.698	
76		0.694			0.658	
115		0.685			0.682	
72		0.680			0.760	
104		0.675			0.708	
91		0.673			0.701	
52		0.653			0.687	
142		0.595			0.622	
7			0.762			0.644
4			0.701			0.606
54			0.681			0.660
2			0.643			0.659
55			0.617			0.619
8			0.483			0.514
Factor						
P	−			−		
N	−0.239	−		−0.183	−	
C	0.386	−0.431	−	0.423	−0.352	−
Model fit						
Sample 1, *χ*^2^(558) = 1,918.764, *P* < 0.001, SRMR = 0.028, CFI = 0.927, RMSEA = 0.047, 90% CI [0.045, 0.049]						
Sample 2, *χ*^2^(558) = 1,839.997, *P* < 0.001, SRMR = 0.029, CFI = 0.927, RMSEA = 0.046, 90% CI [0.043, 0.048]						

Values under each factor correspond to standardized factor loadings; only loadings ≥0.32 are reported for clarity. The items to which the numbers (no.) correspond can be seen in Table 3. Coefficients for factors P, N and C at the bottom of the table (above model fit) denote the standardized loadings of the factors on each other. All factors also yielded good to excellent Cronbach’s *α*-values (Sample 1, positive, *α* = 0.927; negative, *α* = 0.943; competence, *α* = 0.818; Sample 2, positive, *α* = 0.923; negative, *α* = 0.943; competence, *α* = 0.802).

To show that our model has equivalent factor structure, loadings and intercepts regardless of participants’ country, robot examples used and several key demographic characteristics, we tested configural, metric and scalar measurement invariance^144–146^. As shown in Table 5, measurement invariance was demonstrated in all cases given that the configural model demonstrated good to excellent fit (SRMR < 0.05, CFI > 0.90, RMSEA < 0.06 (refs. ^141–143^); changes in SRMR, CFI and RMSEA were, respectively, ≤0.030, 0.010 and 0.015 for the metric model and ≤0.015, 0.010 and 0.015 for the scalar model^144^. Since we could not analyse measurement invariance for participants who did versus did not use robots at work in Study 5 because the number of those who did was insufficient (Table 1), we tested this in Study 6 where sample sizes were larger. In Study 6 we also computed measurement invariance for additional participant characteristics assessed in that study (educational attainment, income, being liberal versus conservative, ethnic identity and relationship status). Measurement invariance was demonstrated in all cases (Supplementary Table 10).

**Table 5:** Measurement invariance tests of the PRR scale for country: United Kingdom versus United States; robot example: A versus B; gender: female versus male; age: below median versus median and above; and employment status: employed versus unemployed (Study 5)

Invariance model	SRMR	ΔSRMR	CFI	ΔCFI	RMSEA	ΔRMSEA
Sample 1 (UK) versus Sample 2 (US)						
Configural	0.028	−	0.927	−	0.046	−
Metric	0.033	0.005	0.926	0.001	0.045	0.001
Scalar	0.035	0.002	0.923	0.003	0.045	<0.001
Robot example A versus B (Sample 1)						
Configural	0.031	−	0.924	−	0.048	−
Metric	0.038	0.007	0.925	0.001	0.046	0.002
Scalar	0.038	<0.001	0.924	0.001	0.046	<0.001
Robot example A versus B (Sample 2)						
Configural	0.032	−	0.927	−	0.046	−
Metric	0.040	0.008	0.925	0.002	0.045	0.001
Scalar	0.040	<0.001	0.924	0.001	0.044	0.001
Gender: female versus male (Sample 1)						
Configural	0.031	−	0.926	−	0.048	−
Metric	0.041	0.010	0.924	0.002	0.047	0.001
Scalar	0.042	0.001	0.920	0.004	0.047	<0.001
Gender: female versus male (Sample 2)						
Configural	0.032	−	0.927	−	0.046	−
Metric	0.041	0.009	0.926	0.001	0.044	0.002
Scalar	0.041	<0.001	0.923	0.003	0.045	0.001
Age: <48 (median) versus ≥48 years (Sample 1)						
Configural	0.031	−	0.925	−	0.048	−
Metric	0.039	0.008	0.924	0.001	0.046	0.002
Scalar	0.040	0.001	0.919	0.005	0.047	0.001
Age: <45 (median) versus ≥45 years (Sample 2)						
Configural	0.033	−	0.921	−	0.048	−
Metric	0.041	0.008	0.919	0.002	0.047	0.001
Scalar	0.043	0.002	0.915	0.004	0.047	<0.001
Employment status: employed versus unemployed (Sample 1)						
Configural	0.031	−	0.926	−	0.048	−
Metric	0.039	0.008	0.924	0.002	0.047	0.001
Scalar	0.039	<0.001	0.923	0.001	0.046	0.001
Employment status: employed versus unemployed (Sample 2)						
Configural	0.032	−	0.925	−	0.047	−
Metric	0.039	0.007	0.924	0.001	0.045	0.002
Scalar	0.039	<0.001	0.923	0.001	0.045	<0.001

The symbol Δ refers to the absolute value of a change in fit indices for an invariance model relative to the previous (that is, metric minus configural; scalar minus metric). For robot example, ‘A’ indicates that the robot example for the domain to which participants from Study 5 were randomly allocated belonged to one of the two stimulus sets used in the present research, while ‘B’ indicates that the robot example belonged to the other stimulus set (Supplementary Table 7). For gender, very few participants identified themselves as ‘other’ or did not disclose any information (Table 1), and they were therefore randomly classified as either ‘female’ or ‘male’ so they could be used in invariance testing. For employment status the category ‘employed’ includes those participants who were self-employed or working for an employer. For use of robots at work we could not analyse measurement invariance because of the insufficient number of participants who used robots at work (Table 1). However, for Study 6, in which sample sizes were larger (Table 1), we tested measurement invariance for this variable and for additional participant characteristics assessed in that study (educational attainment, income, political orientation: liberal versus conservative, ethnic identity and relationship status). Measurement invariance was met in all cases (Supplementary Table 10).

Overall, the structure of the PNC model and its validity across different subgroups of participants were confirmed.

### Phase 3: examining individual difference predictors

In Study 6, to identify the main predictors of the PNC model we followed the analytic strategy described in Methods. We first computed 11 common machine learning models (for example, linear least squares, lasso^109,110^) for the positive, negative and competence dimensions separately. The key predictors in each model were 79 personality measures that were found to be conceptually or theoretically relevant to the PNC dimensions. We selected these measures by examining several comprehensive psychological scale databases (for example, Database of Individual Differences Survey Tools^147^). All measures and their justifications are available in Supplementary Table 11.

We then identified the most predictive models, which were the same across all PNC dimensions: conditional random forest (r.m.s.e._Positive_ = 0.919; r.m.s.e._Negative_ = 0.988; r.m.s.e._Competence_ = 0.778), linear least squares (r.m.s.e._Positive_ = 0.929; r.m.s.e._Negative_ = 1.000; r.m.s.e._Competence_ = 0.795), ridge (r.m.s.e._Positive_ = 0.921; r.m.s.e._Negative_ = 0.994; r.m.s.e._Competence_ = 0.787), lasso (r.m.s.e._Positive_ = 0.921; r.m.s.e._Negative_ = 0.993; r.m.s.e._Competence_ = 0.784), elastic net (r.m.s.e._Positive_ = 0.921; r.m.s.e._Negative_ = 0.993; r.m.s.e._Competence_ = 0.784) and random forest (r.m.s.e._Positive_ = 0.925; r.m.s.e._Negative_ = 0.995; r.m.s.e._Competence_ = 0.781).

Subsequently we determined all individual differences that were among the top 30 predictors across these six models and that were also statistically significant in the linear least-squares model after applying the false-discovery rate^148^ correction (Supplementary Tables 12–15). Several variables met these criteria and were therefore deemed the main individual difference predictors of PNC dimensions. For the positive dimension these were general risk propensity (GRP^149^), anthropomorphism (IDAQ^150^) and parental expectations (FMPS_PE^151^); for the negative dimension these were trait negative affect (PANAS_TNA^152^), psychopathy (SD3_P^153^), anthropomorphism (IDAQ^150^) and expressive suppression (ERQ_ES^154^); and for the competence dimension these were approach temperament (ATQ_AP^155^) and security-societal (PVQ5X_SS^156^). According to the most interpretable model (that is, the linear least squares) these most predictive individual differences were positively associated with the corresponding PNC dimensions.

In Study 7, to replicate the findings we measured the most predictive individual differences in wave 1 and used linear regressions to show that they significantly predicted PNC dimensions in wave 2 (Table 6), consistent with Study 6. Furthermore, we examined various potential mediators of the relationship between each predictor and a PNC dimension using parallel mediation analyses^157^ percentile-bootstrapped with 10,000 samples (for mediators and mediated effects see Table 7; the rationale behind each mediator and detailed mediation analyses are available in Supplementary Table 17 and Supplementary Results, respectively).

**Table 6:** Main individual difference predictors of the positive, negative and competence dimensions (Study 7)

Variable	b	s.e. b	99% CI	*t*	*P*	*f*^2^
DV, positive dimension						
Model 1: GRP positively predicts DV						
(constant)	2.938	0.086	2.717–3.159	34.329	<0.001	1.102
GRP	0.145	0.035	0.054–0.236	4.099	<0.001	0.016
Model 2: IDAQ positively predicts DV						
(constant)	2.791	0.068	2.615–2.967	40.888	<0.001	1.564
IDAQ	0.178	0.023	0.120–0.236	7.868	<0.001	0.058
Model 3: FMPS_PE positively predicts DV						
(constant)	2.786	0.107	2.511–3.061	26.137	<0.001	0.639
FMPS_PE	0.158	0.034	0.071–0.245	4.684	<0.001	0.021
DV, negative dimension						
Model 4: PANAS_TNA positively predicts DV						
(constant)	2.090	0.078	1.888–2.292	26.734	<0.001	0.669
PANAS_TNA	0.124	0.047	0.003–0.245	2.634	0.009	0.006
Model 5: IDAQ positively predicts DV						
(constant)	2.133	0.060	1.979–2.287	35.714	<0.001	1.193
IDAQ	0.056	0.020	0.005–0.107	2.840	0.005	0.008
Model 6: SD3_P positively predicts DV						
(constant)	1.707	0.098	1.456–1.959	17.496	<0.001	0.286
SD3_P	0.296	0.048	0.172–0.420	6.152	<0.001	0.035
Model 7: ERQ_ES positively predicts DV						
(constant)	2.041	0.083	1.827–2.255	24.586	<0.001	0.565
ERQ_ES	0.064	0.021	0.010–0.117	3.085	0.002	0.009
DV, competence dimension						
Model 8: ATQ_AP positively predicts DV						
(constant)	4.470	0.151	4.079–4.860	29.527	<0.001	0.816
ATQ_AP	0.157	0.029	0.081–0.233	5.345	<0.001	0.027
Model 9: PVQ5X_SS positively predicts DV						
(constant)	4.859	0.106	4.586–5.133	45.797	<0.001	1.962
PVQ5X_SS	0.097	0.024	0.034–0.160	3.962	<0.001	0.015

DV, dependent variable. Model 1, *R*^2^ = 0.015; Model 2, *R*^2^ = 0.055; Model 3, *R*^2^ = 0.020; Model 4, *R*^2^ = 0.006; Model 5, *R*^2^ = 0.007; Model 6, *R*^2^ = 0.034; Model 7, *R*^2^ = 0.009; Model 8, *R*^2^ = 0.026; and Model 9, *R*^2^ = 0.014. All models had 1,069 residual degrees of freedom. In all models we used *t*-tests (two-sided) to assess the significance of the coefficients, with the significance criterion being *P* < 0.010 based on the Benjamini–Yekutieli correction^216,217^ for multiple comparisons. The table contains raw *P* values that are statistically significant if they meet this benchmark; therefore, all nine predictors reached statistical significance. *f*^2^ denotes Cohen’s *f*^2^ effect size^218^. GRP and FMPS_PE were measured on a 1–5 scale (1, strongly disagree; 5, strongly agree); IDAQ was measured on a 0–10 scale (0, not at all; 10, very much); PANAS_TNA was measured on a 1–5 scale (1, strongly disagree; 5, strongly agree); SD3_P was measured on a 1–5 scale (1, disagree strongly; 5, agree strongly); ERQ_ES and ATQ_AP were measured on a 1–7 scale (1, strongly disagree; 7, strongly agree); and PVQ5X_SS was measured on a 1–6 scale (1, not like me at all; 6, very much like me).

**Table 7:** All variables tested as mediators and their mediated effects (in parentheses), listed under the relevant individual difference predictors of the positive, negative and competence dimensions (Study 7)

DV, positive dimension
Mediators tested for predictor GRP
GRP_M1 (ab = 0.001, 99% CI_bootstrapped_ [−0.006, 0.010], ab_%_ = 0.007). I think robots do not pose a risk to me.
GRP_M2 (ab < 0.001, 99% CI_bootstrapped_ [−0.016, 0.016], ab_%_ = 0.002). I think robots do not pose a risk to society.
**GRP_M3 (ab** **=** **0.057, 99% CI**_**bootstrapped**_ **[0.029, 0.093], ab**_**%**_ **=** **0.393).** I think robot adoption has its risks, but these risks are what make robots appealing.
**GRP_M4 (ab** **=** **0.013, 99% CI**_**bootstrapped**_ **[0.001, 0.032], ab**_**%**_ **=** **0.090).** I am curious to see how robots will change the world.
GRP_M5 (ab = −0.003, 99% CI_bootstrapped_ [−0.018, 0.009], ab_%_ = −0.021). I think robot adoption has its risks, but the potential rewards are high.
GRP_M6 (ab = −0.001, 99% CI_bootstrapped_ [−0.010, 0.006], ab_%_ = −0.007). The benefits of robots outweigh their risks.
GRP_M7 (ab = 0.020, 99% CI_bootstrapped_ [−0.004, 0.048], ab_%_ = 0.138). (1) I feel that technology helps me to align with my ideal self; (2) I feel that technology helps me to succeed in my endeavours.^a^
Mediators tested for predictor IDAQ^b^
IDAQ_M1 (ab = 0.004, 99% CI_bootstrapped_ [−0.009, 0.019], ab_%_ = 0.022). When I interact with a non-human entity (e.g., robots, machines, nature, animals), I can experience strong emotions that I would normally experience toward human beings.
**IDAQ_M2 (ab** **=** **0.021, 99% CI**_**bootstrapped**_ **[0.008, 0.039], ab**_**%**_ **=** **0.118).** Interacting with non-human entities (e.g., robots, machines, nature, animals) helps me fulfil the need to experience strong emotions regularly.
**IDAQ_M3 (ab** **=** **0.035, 99% CI**_**bootstrapped**_ **[0.016, 0.060], ab**_**%**_ **=** **0.197).** When I see a non-human entity (e.g., robots, machines, nature, animals) that has human characteristics, I experience positive feelings.
IDAQ_M4 (ab = −0.001, 99% CI_bootstrapped_ [−0.007, 0.004], ab_%_ = −0.006). When I see a non-human entity (e.g., robots, machines, nature, animals) that has human characteristics, I experience negative feelings.
Mediators tested for predictor FMPS_PE
**FMPS_PE_M1 (ab** **=** **0.031, 99% CI**_**bootstrapped**_ **[0.009, 0.058], ab**_**%**_ **=** **0.196).** I value robots because they are closer to perfection than humans.
**FMPS_PE_M2 (ab** **=** **0.027, 99% CI**_**bootstrapped**_ **[0.006, 0.055], ab**_**%**_ **=** **0.171).** I value robots because I believe they can help me fulfil my own high expectations.
FMPS_PE_M3 (ab = 0.011, 99% CI_bootstrapped_ [−0.024, 0.048], ab_%_ = 0.070). I value robots because I believe they can help me fulfil my parents’ high expectations.
FMPS_PE_M4 (ab = 0.009, 99% CI_bootstrapped_ [−0.013, 0.032], ab_%_ = 0.057). I value robots because their superiority over humans allows me to become superior over others.
FMPS_PE_M5 (ab = 0.026, 99% CI_bootstrapped_ [−0.012, 0.065], ab_%_ = 0.165). I value robots because I believe they help me better cope with my parents’ high expectations of me.
**FMPS_PE_M6 (ab** **=** **0.033, 99% CI**_**bootstrapped**_ **[0.011, 0.062], ab**_**%**_ **=** **0.209).** I value robots because I believe they help me better cope with my own high expectations of myself.
**DV, negative dimension**
Mediators tested for predictor PANAS_TNA
**Activated Displeasure—12-PAC_AD (ab** **=** **0.231, 99% CI**_**bootstrapped**_ **[0.029, 0.450], ab**_**%**_ **=** **1.863)**; Deactivated Displeasure—12-PAC_DD (ab = −0.019, 99% CI_bootstrapped_ [−0.180, 0.147], ab_%_ = −0.153); Displeasure—12-PAC_D (ab = −0.030, 99% CI_bootstrapped_ [−0.228, 0.173], ab_%_ = −0.242); Unpleasant Activation—12-PAC_UA (ab = −0.073, 99% CI_bootstrapped_ [−0.231, 0.081], ab_%_ = −0.589); Unpleasant Deactivation—12-PAC_UD (ab = 0.033, 99% CI_bootstrapped_ [−0.054, 0.130], ab_%_ = 0.266)^c^. Measured using 12-point affect circumplex (12-PAC)^158^.
Mediators tested for predictor IDAQ^b^
IDAQ_M1 (ab = 0.001, 99% CI_bootstrapped_ [−0.011, 0.014], ab_%_ = 0.018); IDAQ_M2 (ab = 0.008, 99% CI_bootstrapped_ [−0.002, 0.022], ab_%_ = 0.143); IDAQ_M3 (ab = −0.011, 99% CI [−0.026, −0.001], ab_%_ = −0.196); IDAQ_M4 (ab = −0.005, 99% CI_bootstrapped_ [−0.021, 0.011], ab_%_ = −0.089)^d^. Same as for ‘DV, positive dimension: IDAQ’.
Mediators tested for predictor SD3_P
SD3_P_M1 (ab = −0.011, 99% CI_bootstrapped_ [−0.075, 0.050], ab_%_ = −0.037). I tend to have negative feelings toward other people.
**SD3_P_M2 (ab** **=** **0.122, 99% CI**_**bootstrapped**_ **[0.070, 0.183], ab**_**%**_ **=** **0.412).** I tend to have negative feelings toward other people’s creations and inventions.
**SD3_P_M3 (ab** **=** **0.022, 99% CI**_**bootstrapped**_ **[0.005, 0.050], ab**_**%**_ **=** **0.074).** Using technologies that I am not proficient in makes me feel inferior.
SD3_P_M4 (ab = −0.008, 99% CI_bootstrapped_ [−0.036, 0.020], ab_%_ = −0.027). Technology can expose me for who I am.
**12-PAC_AD (ab** **=** **0.064, 99% CI**_**bootstrapped**_ **[0.010, 0.136], ab**_**%**_ **=** **0.216)**; 12-PAC_DD (ab = −0.005, 99% CI_bootstrapped_ [−0.061, 0.051], ab_%_ = −0.017); 12-PAC_D (ab = −0.028, 99% CI_bootstrapped_ [−0.098, 0.037], ab_%_ = −0.095); 12-PAC_UA (ab = −0.017, 99% CI_bootstrapped_ [−0.067, 0.022], ab_%_ = −0.057); 12-PAC_UD (ab = −0.003, 99% CI_bootstrapped_ [−0.037, 0.029], ab_%_ = −0.010).^c^ Same as for ‘DV, negative dimension: PANAS_TNA’ (ref. ^158^).
Mediators tested for predictor ERQ_ES
ERQ_ES_M1 (ab = 0.007, 99% CI_bootstrapped_ [−0.010, 0.029], ab_%_ = 0.109). At the moment, I feel mentally exhausted.
ERQ_ES_M2 (ab = 0.007, 99% CI_bootstrapped_ [−0.012, 0.027], ab_%_ = 0.109). At the moment, I feel emotionally exhausted.
12-PAC_AD (ab = 0.017, 99% CI_bootstrapped_ [−0.002, 0.043], ab_%_ = 0.266); 12-PAC_DD (ab = −0.005, 99% CI_bootstrapped_ [−0.026, 0.013], ab_%_ = −0.078); 12-PAC_D (ab = −0.008, 99% CI_bootstrapped_ [−0.037, 0.014], ab_%_ = −0.125); 12-PAC_UA (ab = −0.007, 99% CI_bootstrapped_ [−0.023, 0.003], ab_%_ = −0.109); 12-PAC_UD (ab = −0.001, 99% CI_bootstrapped_ [−0.013, 0.009], ab_%_ = −0.016).^c^ Same as for ‘DV, negative dimension: PANAS_TNA’ (ref. ^158^).

DV, competence dimension
Mediators tested for predictor ATQ_AP
ATQ_AP_M1 (ab = 0.008, 99% CI_bootstrapped_ [−0.004, 0.026], ab_%_ = 0.051). I value robots that can help me perform better than others.
ATQ_AP_M2 (ab = 0.015, 99% CI_bootstrapped_ [−0.007, 0.042], ab_%_ = 0.096). I value robots that can help me become better at a task, goal, or skill that I want to accomplish or master.
ATQ_AP_M3 (ab = −0.010, 99% CI_bootstrapped_ [−0.029, 0.005], ab_%_ = −0.064). When evaluating other people, it is important to me how good they are at what they do.
ATQ_AP_M4 (ab = 0.012, 99% CI_bootstrapped_ [−0.001, 0.030], ab_%_ = 0.076). When evaluating robots, it is important to me how good they are at what they do.
**ATQ_AP_M5 (ab** **=** **0.020, 99% CI**_**bootstrapped**_ **[0.002, 0.042], ab**_**%**_ **=** **0.127).** I highly value exceptional skills and competencies.
ATQ_AP_M6 (ab = 0.003, 99% CI_bootstrapped_ [−0.021, 0.027], ab_%_ = 0.019). When I see a human that can accomplish something challenging, I react strongly to it.
ATQ_AP_M7 (ab = −0.006, 99% CI_bootstrapped_ [−0.032, 0.020], ab_%_ = −0.038). When I see a robot that can accomplish something challenging, I react strongly to it.
ATQ_AP_M8 (ab = 0.011, 99% CI_bootstrapped_ [−0.012, 0.037], ab_%_ = 0.070). When I see the potential for robots to improve human life, I get excited.
ATQ_AP_M9 (ab = 0.017, 99% CI_bootstrapped_ [−0.010, 0.047], ab_%_ = 0.108). When I encounter robots or other inventions that can better my life, I react strongly to it.
ATQ_AP_M10 (ab = 0.018, 99% CI_bootstrapped_ [−0.002, 0.043], ab_%_ = 0.115). I am thrilled when seeing robots helping society to achieve tasks that are often difficult to accomplish.
Mediators tested for predictor PVQ5X_SS
PVQ5X_SS_M1 (ab = 0.009, 99% CI_bootstrapped_ [−0.005, 0.027], ab_%_ = 0.093). I think advanced technology (e.g., robots, machines, devices) can make the country more powerful.
PVQ5X_SS_M2 (ab = 0.016, 99% CI_bootstrapped_ [−0.008, 0.042], ab_%_ = 0.165). With effective use of advanced technology (e.g., robots, machines, devices), the country maintains its strength to defend its citizens.
PVQ5X_SS_M3 (ab = 0.004, 99% CI_bootstrapped_ [−0.003, 0.015], ab_%_ = 0.041). I think advanced technology (e.g., robots, machines, devices) can create order and stability.
**PVQ5X_SS_M4 (ab** **=** **0.015, 99% CI**_**bootstrapped**_ **[<0.001, 0.036], ab**_**%**_ **=** **0.155).** Advanced technology (e.g., robots, machines, devices) is a reflection of how powerful our society is.
PVQ5X_SS_M5 (ab = 0.007, 99% CI_bootstrapped_ [−0.001, 0.019], ab_%_ = 0.072). Being surrounded by advanced technology (e.g., robots, machines, devices) that is effective at what it does makes me feel safe.

For each mediator we first present its name, followed by its mediated effect (ab) in parentheses.

Mediated effects are presented in raw units. For example, for GRP_M3 (ab = 0.057, 99% CI [0.029, 0.093], ab_%_ = 0.393) the mediated effect ab indicates that for one unit increase in GRP as a predictor, the positive dimension increased by 0.057 units, which is the effect that can be accounted for by the mediator (GRP_M3). For an easier understanding of the magnitude of each mediated effect, ab_%_ is also reported and indicates the percentage of the total effect between a predictor and DV (that is, coefficients b in Table 6) explained by the mediator. In some cases ab_%_ can exceed 1 (that is, 100%), which means that the effect travelling through the mediator is larger than the total effect itself. A mediated effect is significant only if its 99% CI_bootstrapped_ does not contain 0 (ref. ^157^). Some mediated effects (ab and ab_%_) are negative; this means they are in the opposite direction to the effect between a predictor and DV, and therefore do not explain their relationship. All mediators that successfully explained a portion of the relationship between a predictor and DV (that is, mediated effects that are positive and whose 99% CI_bootstrapped_ does not contain 0) are presented in bold typeface.

^a^The two items for GRP_M7 were averaged into a composite score.

^b^For IDAQ as a predictor we used the same mediators for the positive and negative dimensions, considering that we wanted to ensure that any potential differences between the mechanisms for these two dimensions are not a consequence of different mediators being used in the mediation models.

^c^The five 12-PAC mediators capture state affect because they were assessed in relation to how people currently felt.

^d^Although the mediated effect of IDAQ_M3 was significant, the direction of this effect was negative (ab = −0.011) and thus opposite to the positive direction of the relationship between IDAQ and the negative domain (Table 6). Therefore, the mediator failed to explain this relationship.

To aid the interpretation of the mechanisms, below we summarize the mediated effects from Table 7 that successfully explain a portion of the relationship between the key individual differences and PNC dimensions.

For the positive dimension, GRP^149^ was a positive predictor because people scoring higher on this trait valued the risks associated with robot adoption (GRP_M3) and were curious to see how robots would change the world (GRP_M4). Moreover, IDAQ^150^ was a positive predictor because people scoring higher on this trait generally felt positive towards inanimate entities with human features (IDAQ_M3), and because interaction with such entities helped them fulfil the need to experience strong emotions regularly (IDAQ_M2). FMPS_PE^151^ was also a positive predictor due its association with valuing robots because they were closer to perfection than humans (FMPS_PE_M1), and also because they could help humans fulfil their own high expectations (FMPS_PE_M2) and could help humans cope with their own high expectations of themselves (FMPS_PE_M6).

For the negative dimension, PANAS_TNA^152^ was a positive predictor because people scoring high on this trait were more likely to be in a state of activated displeasure (for example, feeling scared and upset; 12-PAC_AD^158^). Furthermore, SD3_P^153^ was a positive predictor because people scoring high on this trait were also more likely to be in the state of activated displeasure (12-PAC_AD^158^), had negative feelings towards other people’s inventions (SD3_P_M2) and felt inferior towards technologies in which they were not proficient (SD3_P_M3). For ERQ_ES^154^ and IDAQ^150^, we did not manage to explain the mechanism behind their relationship with the negative dimension.

For the competence dimension, ATQ_AP^155^ was a positive predictor because people scoring high on this trait were more likely to value exceptional skills and competencies (ATQ_AP_M5). PVQ5X_SS^156^ was also a positive predictor because it was associated with people linking advanced technology (for example, robots and machines) with how powerful society is (PVQ5X_SS_M4).

## Discussion

In this section we first discuss (1) our findings and their contributions in relation to previous research to achieve inductive integration^77^ and then (2) the main limitations (for a detailed discussion see Supplementary Discussion).

Starting with Phase 1, we first discuss the robot definition (Table 2) and then the domains (Table 2). Regarding the definition, ours and that of IEEE^22^ both conceptualize robots as devices or entities that can perform different tasks (Part 1, Table 2), emphasize that robots can have different degrees of autonomy (Part 2, Table 2) and include robots’ composition (Part 5, Table 2). However, the two definitions also have unique elements: ours includes robots’ durability (Part 3, Table 2) and positive/negative attributes (Part 4, Table 2) whereas the IEEE definition includes robots’ capability to form robotic systems. Overall, although our definition is somewhat more nuanced, both definitions are remarkably aligned, which indicates that experts and lay individuals perceive robots similarly.

Regarding the domains in which robots operate we have identified 28 (Table 2), which is more than professional organizations usually propose (for example, the IEEE lists 18 domains on their website, https://robots.ieee.org/learn/types-of-robots/). However, this is not surprising because our list was intentionally nuanced to enable the identification of a comprehensive sample of robots, and we hope that other scholars will adopt it in their research for this purpose. It is important to emphasize that, despite the meticulous procedure used to develop the list, it is possible that (1) we failed to identify more niche domains and (2) the number of domains might increase as technology advances.

Continuing with Phase 2, we first compare the psychological processes of the PNC model (Table 3) against those reported in previous research and then discuss the model (Tables 3 and 4) more specifically. In general, participants evoked the processes identified in the literature reviewed in the Introduction, including positive feelings such as happiness^10,33^ (Item 32); negative feelings such as anxiety^27^ (Item 13); performance^48^ and usefulness^19^ (Items 4 and 5); anthropomorphism^36,159^ (Items 24 and 27); and various approach^66^ (Items 22 and 40) or avoidance^26^ (Items 11 and 52) behaviours (Table 3). Importantly, participants also described many infrequent or previously unidentified processes. For example, they indicated that robots contribute to human degeneration (Item 142); lead to existential questioning (Item 148); make people feel dehumanized (Item 25); help humans self-improve (Item 132); and restrict freedom (Item 114).

One of the main contributions of our research is showing that these seemingly highly diverse psychological processes fall under three dimensions: positive (P), negative (N) and competence (C) (Tables 3 and 4). In general, previous research on human–robot relationships and interactions has focused on studying and measuring specific psychological reactions to robots (for example, safety, anthropomorphism, animacy, intelligence, likeability and various social attributes^159,160^) but did not attempt to identify all these reactions and investigate them under an all-encompassing construct of psychological processes. In that regard, the PNC model can be seen as an integrative framework that links and organizes an exhaustive list of psychological processes, both those that researchers have already studied separately and the less common ones generated by our participants. We believe that our model moves the field forward, not only through this integration but also by enabling researchers to systematically study psychological processes regarding robots by (1) using the PNC as a guide to inform the design of future research on these processes and (2) employing the PRR scale to measure them.

One of the most interesting insights spawned by the PNC model stems from comparing it with the stereotype content model (SCM^161,162^). According to the SCM, people form impressions of other humans along two dimensions: warmth (that is, positive and negative social characteristics) and competence (that is, a person’s ability to successfully accomplish tasks). Although our model is broader than the SCM because it comprises all psychological processes rather than only social and intellectual characteristics, the competence dimensions from the two models are thematically comparable whereas the positive and negative attributes from the SCM’s warmth dimension are broadly aligned with our positive and negative dimensions. These comparisons suggest that (1) people use similar criteria when forming impressions of robots and humans and (2) robots’ similarity to humans does not play a role in this regard, because many of our stimuli depicted non-humanoid robots (Supplementary Table 7).

Ending with Phase 3, we discuss our findings on individual difference predictors (Tables 6 and 7) in relation to the previous relevant literature. In this respect, researchers found that extraversion, openness and anthropomorphism predicted positive responses to robots^163–166^; the need for cognition predicted lower negative attitudes towards robots^167^; and animal reminder disgust, neuroticism and religiosity predicted experiencing robots as eerie^168^. Among these, our research corroborated only the positive relationship between anthropomorphism and positive responses (Table 6).

We also went beyond previous research by discovering many relationships not easily anticipated by theory. For example, although we had a sound rationale behind each predictor (Supplementary Table 11) it would have been difficult to foresee psychopathy as the most robust predictor of the negative dimension (Table 6)^153^. We also did not expect that one of the main mechanisms behind negative robot perceptions would be negative feelings towards other people’s creations and the state of activated displeasure, which mediated the relationship between psychopathy and the negative PNC dimension (Table 7). Therefore, using a data-driven approach allowed us to generate unexpected insights, thus diversifying the body of knowledge on psychological reactions to robots^79,81,98^.

There are several limitations to this research. First, the stimuli were not physical robots but their depictions. These stimuli hold ecological validity because people often interact with robots indirectly (for example, via social media or various websites), and many psychological processes may therefore be shaped in this manner. Nonetheless, previous research showed that direct interaction with robots impacts people’s experiences^27,169,170^. Therefore, based on the present findings it is not known whether our taxonomy applies to the physical counterparts of the robots depicted by our stimuli, and investigating this is currently unachievable because many of these robots are inaccessible for in-person research due to their size, cost, limited production or potential use as weapons (for example, industrial and military robots). However, this research may be possible in the future if such robots become more accessible.

Second, participants were from Western, educated, industrialized, rich and democratic^171^ countries (United Kingdom and United States). Because our research proposed and investigated a construct (that is, psychological processes regarding robots) from scratch, our priority was to establish its foundations. Combining the investigation of cultural differences with this agenda using equally meticulous methods would have exceeded the scope of a single article. Nevertheless, because measurement invariance analyses showed that the PNC model applies to individuals regardless of their income, age, education, use of robots at work, political orientation, ethnic identity and relationship status, it is plausible that the model would generalize to countries that differ from the United Kingdom or United States on these population characteristics. Conducting an in-depth examination of this question will be a crucial step as this research topic progresses.

Third, we recruited online participants who are inherently more confident with technology. Whereas this might have influenced the findings, alternative modes of recruitment (for example, laboratory) would have yielded smaller and less representative participant samples^172–176^. Furthermore, to reduce the chance of technological proficiency biasing the findings, all machine learning models controlled for a variable indicative of technological proficiency (that is, previous frequency of interaction with robots; Supplementary Tables 11 and 12).

Finally, rapid technological development might make robots with an embodiment similar to humans able to perform and simulate all human activities, thereby substantially changing how people perceive robots. However, since our comparison of the PNC model and SCM^161,162^ indicates that people form impressions of robots and humans in a similar manner, it is unlikely that robots becoming more like humans will have a notable impact on the structure of our model. Even if it does, the PNC can be updated via the same methodological procedures we used.

## Methods

This research complies with the ethics policy and procedures of the London School of Economics and Political Science (LSE), and has also been approved by its Research Ethics Committee (no. 20810). Informed consent was obtained from all participants and they were compensated for their participation. Table 1 summarizes key participant information. In Studies 4–6, participants were recruited to be reasonably representative of the UK/US populations for age, gender and geographical region, and in Study 1 (Sample 1) for gender only. More comprehensive breakdowns of participant information and the criteria used for representative sampling are available in Supplementary Tables 1 and 2.

To be included in analyses, participants had to pass seriousness checks^177^, instructed-response items (for example, please respond with ‘somewhat disagree’)^178–180^, understanding checks in which they identified the main research topic (that is, robots) amongst dummy topics (for example, animals or art) and completely automated public Turing tests to tell computers and humans apart (CAPTCHAs), used to safeguard against bots^181^. The number of these quality checks varied per study. For seriousness checks: Study 1, two (one per sample); Study 2, one; Study 3, one; Study 4, two (one per sample); Study 5, two (one per sample); Study 6, one; and Study 7, two (one per wave). For instructed-response items: Study 1, six (two in Sample 1 and four in Sample 2); Studies 2 and 3, none; Study 4, eight (four per sample); Study 5, four (two per sample); Study 6, three; and Study 7, three (two in wave 1 and one in wave 2). For understanding checks: Study 1, two (one per sample); Study 2, one; Study 3, one; Study 4, two (one per sample); Study 5, two (one per sample); Study 6, one; and Study 7, none. For CAPTCHA: Study 1, one (in Sample 2); Study 2, one; Study 3, one; Study 4, two (one per sample); Study 5, two (one per sample); Study 6, one; and Study 7, two (one per wave).

In Studies 4–7, which were quantitative, we employed pairwise deletion for missing data because various simulations showed that this does not bias the type of analyses we used when missing data are infrequent (≤5%)**—**even in smaller participant samples (for example, 240)**—**and larger samples are generally more robust to missing data^182,183^. In our analyses, the percentage of participants with missing data never exceeded 1.95.

The analyses using machine learning models (Study 6) did not rely on distributional assumptions due to cross-validation^184^, and neither did the mediation analyses (Study 7) due to bootstrapped confidence intervals used to test mediated effects^157^. All other quantitative analyses assumed a normal distribution of data. Because formal normality tests are sensitive to small deviations that do not bias findings^134^, we assumed variables to be normal if they had skewness between −2 and 2 and kurtosis between −7 and 7 (refs. ^185–187^). All the required variables met these criteria (Supplementary Tables 18–23). Given the large sample sizes we used, even severe deviations from normality would not compromise the validity of statistical inferences^157,188,189^.

Next, we succinctly describe the methods of the studies in each phase (for a more comprehensive description, see Supplementary Methods). Study 7 was preregistered on 12 December 2021 via the Open Science Framework (OSF) and can be accessed using this link: https://osf.io/nejvm?view_only = 79b6eeee42e24cb2a977927712bdcdd2. There were no deviations from the preregistered protocol. Data and analysis codes for all studies are also publicly available via the OSF using the following link: https://osf.io/2ntdy/?view_only = 2cacc7b1cf2141cf8c343f3ee28dab1d

### Phase 1: mapping a comprehensive content space of robots

#### Study 1

Sample size. To determine Sample 1 size we relied on previous work showing that, in qualitative research, samples having 30–50 participants tend to reach the point of data saturation, which means that the addition of further participants produces little new information^190–195^. We recruited a considerably larger sample (266; Table 1) to ensure that the study detected all important robot characteristics because the robot definition we wanted to develop was essential for all subsequent studies. For Sample 2 we recruited 100 participants (Table 1), which is comparable to other studies using hierarchical clustering^196,197^ given the lack of guidelines on optimal sample sizes for this technique (for additional insights based on simulations, see Supplementary Methods).

Procedure. In Sample 1, participants first answered the consent form after which they were presented with three items that elicited robot characteristics. In the following order, they were asked to: (1) state the first thing that comes to mind when they think about a robot; (2) define in their own words what a robot is; and (3) list as many characteristics they associate with robots that they could think of. At the end we assessed participant information, including gender, age, employment status and use of robots at work (Table 1). In Sample 2, after answering the consent form, participants were exposed to 277 robot characteristics produced by Sample 1 (Supplementary Table 3) and were asked to sort them into groups based on similarity. In this regard, participants were provided with up to 60 empty boxes representing different groups into which they could drag the characteristics they perceived as being similar. At the end, participant information was assessed as for Sample 1.

Analytic approach. We first extracted robot characteristics generated by Sample 1 participants for the three questions described in the Study 1 procedure and then rephrased those that were stated vaguely (for example, ‘appearance of thought’) into a more precise formulation (for example, ‘appears to think on its own’). Next, we deleted all characteristics that were identical and therefore redundant. However, we included many items that were overlapping or similar (for example, ‘performs actions’ and ‘performs certain actions’) to ensure that the potential content space of robot characteristics was sampled in detail (for the final list of 277 characteristics see Supplementary Table 3). The characteristics, as sorted into categories by Sample 2 participants, were subjected to hierarchical cluster analysis for categorical data^114–116^: a dissimilarity matrix was computed using Gower’s distance^198,199^, clusters were produced using Ward’s linkage method^200,201^ and the optimal number of clusters was determined via the mean silhouette width approach using the partitioning around medoids algorithm^114,202,203^. The five clusters that emerged were then arranged into the robot definition (Table 2).

#### Study 2

Sample size. To determine sample size we followed the same guidelines as for Study 1 (Sample 1) that considered the point of data saturation in qualitative research.

Procedure. After completing the consent form, participants were presented with the robot definition developed in Study 1. They were then asked to think about and list any domains that came to mind in which humans can encounter and/or interact with robots. It was explained that, by ‘domains’, we mean any area of human life and human activity in which people encounter, interact with, use, are helped by and/or are substituted by robots. At the end, participant information was assessed as in Study 1 (Table 1).

Analytic approach. To identify the domains we performed an inductive qualitative content analysis on participants’ responses^117–121^: we first created a list of all domain items identified by participants (see Supplementary Results, subsection ‘Additional analysis output’) and then arranged these items into common categories that correspond to the domains of robot use. The first author created the initial list of categories from the domain items. The list was revised by the remaining authors and, eventually, it was consolidated by all three authors. To ensure that no important domains had been omitted we also consulted the classification of robots proposed by IEEE (https://robots.ieee.org/learn/types-of-robots/), the list of industries and sectors endorsed by the International Labor Organization (https://www.ilo.org/global/industries-and-sectors/lang--en/index.htm) and the articles from our literature review.

### Phase 2: creating the taxonomy of psychological processes

#### Study 3

Sample size. To determine sample size we followed the same guidelines as for Study 1 (Sample 1) and Study 2. Because Study 3 aimed to identify a comprehensive range of psychological processes towards robots, which was a crucial step of our research, we recruited a substantially larger sample than required (350; Table 1) to ensure that even highly infrequent processes were detected.

Procedure. Participants first completed the consent form and were then randomly allocated to five out of the 28 domains we developed (Table 2). After reading the definition of robots (Table 2), we prompted them to think about robots from the allocated domains by writing about interactions they had with such robots, or else about interactions they could imagine or were exposed to via media. To assess participants’ psychological processes, we then asked them to list and describe feelings they had experienced (for affective responses), thoughts they had (for cognitive responses) and actions they engaged in (for behavioural responses) when they interacted with any robots they could think of from each domain, or to write about feelings, thoughts and actions they could conceive in case they had never interacted with these robots. At the end, participant information was assessed as in Studies 1 and 2 (Table 1).

Analytic approach. We implemented iterative categorization^122^. This qualitative analysis involved first splitting participants’ responses to questions assessing their psychological processes into key points (that is, separate issues or thoughts—for example, ‘I think this will be the future’)—and then grouping these points into themes based on similarity. Out of 334 participants who were included in analyses (Table 1), only four produced merely meaningless responses that could not be analysed and the remaining 330 generated 10,332 valid key points (approximately 31 per participant) that were analysed.

#### Study 4

Sample size. Because power analyses are difficult to implement for EFAs before any parameters are known, to determine the sizes of Samples 1 and 2 we consulted various resources that estimated the optimal sample size for EFAs (for a more comprehensive description see Supplementary Methods). Because the size of 1,500 met all the estimates, we recruited the samples required to reach this number after accounting for exclusions (Table 1).

Procedure. The procedure for both samples was identical. After answering the consent form, participants were randomly allocated to a domain (Table 2) and received a specific example of a robot from that domain (Supplementary Table 7) that included an image and description approximately eight lines long. For the sex domain, two robot examples were created (one male and one female) and participants assigned to this domain were randomly allocated to one. Participants were then asked to answer 149 items (Table 3), presented in a randomized order, about the robot in question. At the end, participant information was assessed as in Studies 1, 2 and 3 (Table 1).

Analytic approach. For both samples we planned several steps to determine the optimal factor structure. First, the Kaiser–Meyer–Olkin measure of sampling adequacy and Bartlett’s test of sphericity were required to show that our data are suitable for EFAs^125^. Second, to determine the preliminary number of factors for examining in EFAs, we used parallel analysis^126,127,204^, very simple structure^128^, Velicer map^129^, optimal coordinates^130^, acceleration factor^130^, Kaiser rule^131^ and visual inspection of scree plots^132^. This was advisable because consulting several criteria allows understanding of the range within which lies the optimal number of factors potentially^82,135,136,205,206^. Next, we aimed to evaluate the largest factor solution identified in the previous step against several statistical benchmarks using maximum-likelihood EFAs^104,105^ with Kaiser-normalized^123^ promax rotation^106,124^. Namely, the factor solution was required to produce only valid factors (that is, those that have at least three items with loadings ≥0.5 and cross-loadings <0.32) to be accepted^105,125,133,134^. If these criteria were not met, we aimed to decrease the number of factors by one and evaluate the new solution—this procedure would continue until a satisfying solution was identified. Finally, the accepted factor structure also had to have factors that are coherent and easy to interpret^135,136^. Importantly, this approach to selecting the best structure is not only statistically and semantically viable but has precedent in previous taxonomic research^82,205^.

#### Study 5

Sample size. To determine sample size we used Monte Carlo simulations^207^ based on the data from Samples 1 and 2 (Study 4). Details are available in Supplementary Methods.

Procedure. The procedure for both samples was identical. After answering the consent form, participants were randomly allocated to one robot example. The randomization procedure was the same as in Study 4 except that there were two (rather than one) possible robot examples per domain (Supplementary Table 7). The sex domain had four examples—two male and two female robots. The descriptions of robots were also consistent with Study 4. Participants were then asked to answer the 37 selected items (Table 4), presented in a randomized order, about the robot in question. At the end, participant information was assessed as in Studies 1, 2, 3 and 4 (Table 1).

Analytic approach. The maximum-likelihood with robust standard errors estimator^137,138^ was implemented using ESEM^82,107,108,208^ to test model fit. Target rotation with all cross-loadings specified as targets of zero was chosen^139,140^. The following fit criteria were used^141–143^: SRMR < 0.05, excellent fit; SRMR = 0.05–0.08, good fit; SRMR > 0.08, poor fit; CFI > 0.95, excellent fit; CFI = 0.90–0.95, good fit; CFI < 0.90, poor fit; RMSEA < 0.06, excellent fit; RMSEA = 0.06–0.10, good fit; and RMSEA > 0.10, poor fit. For testing of configural measurement invariance the same fit criteria were used. For metric invariance, changes in SRMR, CFI and RMSEA were required to be ≤0.030, 0.010 and 0.015, respectively, and, for scalar invariance, ≤0.015, 0.010 and 0.015, respectively^144,146^.

### Phase 3: examining individual difference predictors

#### Study 6

Sample size. For machine learning algorithms combined with cross-validation there are no straightforward guidelines for compution of power analyses. Simulations showed that, for the tenfold cross-validations we were planning to use, a sample of 2,000 leads to high generalizability (that is, a likelihood that the results will apply to other samples from the same population) without inflating the time taken to run the models^209^. Therefore, we aimed to recruit a sample that would have approximately 2,200 participants after accounting for exclusions, in case of any additional missing data.

Procedure. After answering the consent form, participants were randomly allocated to one robot example as in Study 5 and asked to answer the PRR scale items (Table 4) presented in a randomized order. They then completed measures that assessed the 79 individual differences we tested as predictors (Supplementary Table 11), ranging from general personality traits, such as BIG 5 (ref. ^210^) or approach temperament^155^, to more specific ones, such as psychopathy^153^. We also measured covariates for inclusion in the models alongside the individual differences (that is, familiarity with the robot, frequency of interaction, descriptive norms, injunctive norms, age, income and political orientation; Supplementary Table 11). Finally participant information was assessed as in the previous studies, with the addition of education level, ethnic identity and relationship status (Supplementary Table 1).

Analytic approach. We implemented a rigorous multistep procedure to select the most predictive individual differences. Using the caret package^109,110^ in R, we computed the following 11 machine learning models for each PNC dimension separately: linear least squares, ridge, lasso, elastic net, *k*-nearest neighbours, regression trees, conditional inference trees, random forest, conditional random forest, neural networks and neural networks with a principal component step. For each model, tenfold cross-validation^184,211–214^ was implemented and all 79 individual differences plus covariates were used as predictors.

The most predictive models were selected using root-mean-square error (r.m.s.e.)^109,110,184^. For each PNC dimension, the model with the highest r.m.s.e. was identified and the remaining models were compared with it using paired-samples *t*-tests (Bonferroni corrected *α* of 0.00167 was used as the significance criterion). Ultimately, the model with the highest r.m.s.e. and those not significantly different from it were identified as the most predictive models. For each of these models we first identified the 30 most important predictors using the VarImp function in R^110^ and then identified individual differences that appeared in the top 30 across all models.

Based on the linear least-squares model—which is in essence a linear regression algorithm combined with cross-validation and thus outputs *P* values—we retained only the most important individual differences identified in the previous step that were also statistically significant after applying false-discovery rate correction^148^. We used this approach because in Study 7 we aimed to replicate the selected predictors using linear regressions; therefore, we wanted to further minimize the likelihood that these predictors are false positives.

#### Study 7

Sample size. Because this study tested the key predictors identified in Study 6, sample size was estimated using power analyses^215^ based on the parameters from that study (Supplementary Methods).

Procedure. The study consisted of two waves. In wave 1, participants first completed the consent form and were then presented with, in a randomized order, the measures assessing the most predictive individual differences identified in Study 7 (Table 6). Finally participant information was assessed as in Studies 1–5. Approximately 4 days after completing wave 1, participants were invited to participate in wave 2. They first completed the consent form and were then presented with the items measuring the mediators (Table 7) in a randomized order. Subsequently, they were randomly allocated to a robot example as in Studies 5 and 6 and asked to answer the PRR scale items (Table 4), presented in a randomized order.

Analytic approach. To test whether the key individual differences predicted the relevant PNC dimensions we used linear regressions, one per predictor (Table 6). Furthermore, to identify the most important mediators we used the Process package (Model 4 (ref. ^157^)) to perform parallel mediation analyses (that is, with all potential mediators analysed together for the relevant predictor; Table 7), percentile-bootstrapped with 10,000 samples. In line with the Benjamini–Yekutieli correction^216,217^, the significance criterion was 0.01 for the regression analyses whereas for the mediated effects we used 99% confidence intervals that are the equivalent of this criterion.

### Reporting summary

Further information on research design is available in the Nature Portfolio Reporting Summary linked to this article.

### Supplementary information

Supplementary Information Supplementary Notes, Methods, Results, Discussion, Tables 1–23 and References.

Reporting Summary

Peer Review File

## Data Availability

The data that support the findings from all studies, as well as the materials used, are publicly available via the OSF: https://osf.io/2ntdy/?view_only = 2cacc7b1cf2141cf8c343f3ee28dab1d), except for the stimuli used in Studies 4–7, which can be obtained from the corresponding author on request.

## Code availability

The codes for all the analyses for the studies conducted are publicly available via the OSF using the following link: https://osf.io/2ntdy/?view_only = 2cacc7b1cf2141cf8c343f3ee28dab1d.
