# Supplementary material for: The positive–negative–competence (PNC) model of psychological responses to representations of robots
Source: Nat Hum Behav. 2023 Oct 2;7(11):1933–54. doi: 10.1038/s41562-023-01705-7 (PMC10663151; doi:10.1038/s41562-023-01705-7)
Supplement: Supplementary file 1 — Supplementary Notes, Methods, Results, Discussion, Tables 1–23 and References. [file 41562_2023_1705_MOESM1_ESM.pdf]

nature human behaviour

Article

https://doi.org/10.1038/s41562-023-01705-7

The positive–negative–competence (PNC) model of psychological responses to representations of robots

In the format provided by the authors and unedited

Contents

Supplementary Notes 4
Extended Overview of Previous Research on Psychological Processes Regarding Robots 4
Supplementary Methods 6
Extended Methods for Studies 1-7 6
Phase One: Mapping a Comprehensive Content Space of Robots From All Domains of Human Activity 6
Study 1 6
Study 2 7
Phase Two: Developing the Taxonomy (i.e., Key Dimensions) of Psychological Processes 8
Study 3 8
Study 4 9
Study 5 12
Phase Three: Determining Main Individual Difference Predictors and Their Mechanisms 14
Study 6 14
Study 7 15
Supplementary Results 17
Extended Results for Studies 1-7 17
Phase One: Mapping a Comprehensive Content Space of Robots From All Domains of Human Activity 17
Study 1 17
Study 2 18
Phase Two: Developing the Taxonomy (i.e., Key Dimensions) of Psychological Processes 19
Study 3 19
Study 4 19
Study 5 20
Phase Three: Determining Main Individual Difference Predictors and Their Mechanisms 21
Study 6 21
Study 7 22
Additional Analysis Output 24
Hierarchical Cluster Analysis (Study 1, Sample 2) 24
Participants' Items Categorized into Robot Domains (Study 2) 28
False Discovery Rate (FDR) Correction by Benjamini and Hochberg[170] Performed on the P-Values from the Linear Least Squares Model Presented in Supplementary Table 13 (Study 6) 37

False Discovery Rate (FDR) correction by Benjamini and Hochberg[170] Performed on the P-Values from the Linear Least Squares Model Presented in Supplementary Table 14 (Study 6) 39

False Discovery Rate (FDR) correction by Benjamini and Hochberg[170] Performed on the P-Values from the Linear Least Squares Model Presented in Supplementary Table 15 (Study 6) 41

Full Output of All Mediation Analyses (Study 7) 43

Supplementary Discussion 75

Supplementary Tables 82

Supplementary Tables 1 and 2: Comprehensive Participant Information 82

Supplementary Table 3: Robot Characteristics Generated by Sample 1 and Arranged into the Clusters Using Hierarchical Clustering on Sample 2 (Study 1) 83

Supplementary Table 4: Definition of Robots Developed from the Clusters of Their Characteristics (Study 1) 86

Supplementary Table 5: Robot Domains and Example Items for Each Domain (Study 2) 87

Supplementary Table 6: Summary of Key Findings (Studies 3 and 4): Psychological Processes, Items Corresponding to Each Process, and the Output of Exploratory Factor Analyses (EFAs) Performed on the Items across Two Participant Samples 89

Supplementary Table 7: Robot Examples Used as Stimuli (Studies 4-7) 94

Supplementary Table 8: Exploratory Structural Equation Models (ESEMs) of the Psychological Responses to Robots (PRR) Scale (Study 5) 99

Supplementary Table 9: Measurement Invariance Tests of the Psychological Responses to Robots (PRR) Scale for Country: UK vs. US; Robot Example: A vs. B; Gender: Female vs. Male; Age: Below Median vs. Median and Above; and Employment Status: Employed vs. Unemployed (Study 5). 101

Supplementary Table 10: Additional Measurement Invariance Tests of the Psychological Responses to Robots (PRR) Scale for Educational Attainment: Secondary or Below vs. Higher Education; Income: Below Median vs. Median and Above; Political Orientation: Liberal vs. Conservative; Ethnic Identity: White vs. Another Identity; In a Relationship: No vs. Yes; and Use of Robots at Work: No vs. Yes (Study 6). 103

Supplementary Table 11: All Predictors (Bold Typeface), the Measures Used to Assess Them (Light Typeface), and Their Justification (Study 6) 104

Supplementary Table 12: The Most Predictive Machine Learning Models and Their 30 Most Important Predictors (Study 6) 112

Supplementary Table 13: Coefficients for the Liner Least Squares Model Computed for the Positive Dimension (Study 6) 115

Supplementary Table 14: Coefficients for the Liner Least Squares Model Computed for the Negative Dimension (Study 6) 118

Supplementary Table 15: Coefficients for the Liner Least Squares Model Computed for the Competence Dimension (Study 6) 121

Supplementary Table 16: Main Individual Difference Predictors of the Positive, Negative, and Competence Dimensions (Study 7) 124

Supplementary Table 17: All Variables that Were Tested as Mediators, Their Mediated Effects (in Parentheses), and Their Justification, Listed Under the Relevant Individual Difference Predictors of the Positive, Negative, and Competence Dimensions (Study 7)........ 126

Supplementary Table 18: Skewness and Kurtosis for Items Assessing Psychological Processes Regarding Robots That Were Used in Statistical Analyses in Study 4 (Sample 1) 136

Supplementary Table 19: Skewness and Kurtosis for Items Assessing Psychological Processes Regarding Robots That Were Used in Statistical Analyses in Study 4 (Sample 2) 140

Supplementary Table 20: Skewness and Kurtosis for the Psychological Responses to Robots (PRR) Scale Items Used in Statistical Analyses in Study 5 (Sample 1)........ 144

Supplementary Table 21: Skewness and Kurtosis for the Psychological Responses to Robots (PRR) Scale Items Used in Statistical Analyses in Study 5 (Sample 2)........ 145

Supplementary Table 22: Skewness and Kurtosis for the Psychological Responses to Robots (PRR) Scale Items Used in Measurement Invariance Testing (Study 6)........ 146

Supplementary Table 23: Skewness and Kurtosis for the Items Used to Examine the Relationships Between the Most Predictive Individual Differences and the Positive-Negative-Competence (PNC) Dimensions (Study 7) 147

Supplementary References........ 148

Supplementary Notes

Extended Overview of Previous Research on Psychological Processes Regarding Robots

In this review, we classify previous research according to three broad categories of psychological processes: 1) affective, which refer to experienced feelings toward robots; 2) cognitive, which refer to thoughts about robots; and 3) behavioral, which refer to actions undertaken toward them. It is also important to specify how we define robots in this context, since their definition is often confined to various specific types (e.g., autonomous, teleoperated, social[1–6]), and describing them as an overarching category can be less straightforward [5–7]. We adopt a general definition proposed by the Institute of Electrical and Electronics Engineers (IEEE[8]), according to which robots are devices that can act in the physical world to accomplish different tasks and are made of mechanical and electronic parts. These devices can be autonomous or, in some cases, subordinated to humans or software agents that act on behalf of humans. They can also form groups (i.e., robotic systems) in which they cooperate to accomplish collective goals (e.g., car manufacturing).

Affective Responses

Previous research has documented a wide range of feelings that people can experience toward robots[3]. Regarding negative feelings, fear and anxiety are frequently encountered in the literature, and they are typically experienced in relation to robots taking people's jobs[9–13]. Individuals can also find robots creepy if they are designed to be human-like but look unnatural and inconsistent with human appearance[14]. In relation to positive feelings, individuals can experience happiness, amazement, amusement, enjoyment, pleasure, or warmth [3,11,12,15–20]. In this context, an important emotion that deserves attention is empathy, as individuals can feel it toward robots even if they are not living beings[3,21–24]. For example, when people observed an image of scissors cutting a robotic hand, a neurological response indicative of empathy was activated[21]. However, research indicates that people do not experience the same levels of empathy for all robots and tend to empathize more strongly with human-looking than inanimate-looking robots[22].

A unique type of emotional experience that deserves a discussion outside of the positive-negative dichotomy is linked to intimate relationships with robots, including friendships and romantic relationships[3]. For example, individuals can get emotionally attached to robots, and even feel attracted to and in love with them[25–30]. Whereas these romantic feelings are perceived by many as taboo, they are not as uncommon as one may think and are becoming increasingly frequent in contemporary society[28]. Nevertheless, it is unclear whether they are driven by loneliness and difficulty connecting with others or by different factors.

Cognitive Responses

People's thoughts about robots can be organized into several themes. One of the main themes is the level of competence robots display in relation to tasks they are specialized in[5,17,31,32]. For example, robots are often seen as efficient and accurate in what they do, and as more physically endurant than humans [5,33,34]. Because of their competence, individuals sometimes perceive robots as beneficial to society[35–38]. However, people can also dwell on robots taking over their jobs, given the intelligence and superiority of robots in characteristics such as speed, precision, and computational power[3,10]. A theme related to competence and frequently evoked in the literature is usefulness. Individuals can consider robots helpful and appreciate their efficiency and effectiveness in accomplishing various tasks (e.g., household chores, planning, carrying heavy load[39–42]). Nevertheless, robots can also be evaluated negatively on any of these qualities and thought of as incompetent, inefficient, or useless[43,44].

Another theme that is important for understanding how people think of robots is anthropomorphism—i.e., ascribing human characteristics to non-living entities[45,46]. For example, people may think of robots as sentient beings that have feelings and personality[47–50]. In that respect, they may also perceive robots as having autonomy, agency, or awareness[51–53], which has led to extensive debates on whether robots should have rights[54–58]. Nonetheless, people can also think of robots as not being comparable to humans and see them as cold, inanimate, and soulless, thus lacking any human qualities[59,60].

Since robots can be judged through the lens of human characteristics, another important theme that may preoccupy people's thoughts is whether robots can be trusted in their capacities as companions, caregivers, tutors, co-workers, and various other roles they assume[61–63]. A related subject is privacy, given that robots' use of personal data is one of the key considerations that may determine how people perceive them and ultimately engage with them[64–66].

Behavioral Responses

Behaviors that have been observed in relation to robots can be classified as either approach (e.g., engaging with robots in some way) or avoidance (e.g., moving physically away from them)[15,67–69]. Common approach behaviors involve communication, cooperation, or some other form of interaction, such as playing and requesting information[3,15,70,71]. More negative approach behaviors have also been documented, including several instances where people were physically and verbally abusing robots[72–74]. In contrast to approach, avoidance behaviors such as escaping, running away, or hiding from robots are not frequently mentioned in the literature and may typically occur in an industrial setup where there is a risk of robots injuring humans[75,76].

Supplementary Methods

Extended Methods for Studies 1-7

Phase One: Mapping a Comprehensive Content Space of Robots From All Domains of Human Activity

Study 1

Participants and Exclusion Criteria

Participants were recruited via Pureprofile (Sample 1) and Amazon Mechanical Turk (MTurk; Sample 2). We used online recruitment platforms because they generally contain more diverse and attentive participants than typical university research pools[77–80].

Sample 1. We initially recruited 266 participants, and 224 remained (Supplementary Table 1) after the exclusion criteria were applied. Participants were excluded if they did not pass a seriousness check[81], two instructed-response items[82–84], and an understanding check in which they were asked to identify the main topic of the study (i.e., robots) amongst a range of dummy topics (e.g., animals, cartoon characters, art).

Sample 2. We initially recruited 100 participants, and 95 remained (Supplementary Table 1) after the exclusion criteria were applied. Similar to Sample 1, participants were excluded if they did not pass a seriousness check[81], four instructed-response items[82–84], and an understanding check in which they were asked to identify the main task of the study (i.e., sorting robot characteristics based on their similarity) amongst a series of dummy alternatives (e.g., sorting different movies based on their similarity). We also included a CAPTCHA item at the end to stop any potential bots from completing the study[85].

Sample Size Rationale

Sample 1. There are no commonly accepted sample size criteria concerning qualitative research approaches in which participants are asked to generate certain information (e.g., listing robot characteristics) because data types and study goals vary widely[86]. However, it has been demonstrated that sample sizes larger than 30-50 (and sometimes even fewer) participants tend to reach the point of data saturation, which implies that adding new participants beyond this number produces very little new information[87–92]. We decided to recruit a considerably larger sample (266, Supplementary Table 1) to maximize the chances that no robot characteristics were potentially missed, since the robot definition we wanted to develop was essential for all subsequent studies.

Sample 2. Understanding power regarding hierarchical clustering as the core analysis implemented on Sample 2 is still in its infancy, especially when dealing with categorical variables as in our case[93]. For this reason, articles that employ the analysis generally do not provide a sample size justification[94,95]. Nevertheless, based on recent simulations, the most important determinant of power seems to be the number of observations per cluster, with 20 observations yielding sufficient power to detect a cluster[93]. In the present research, observations per cluster correspond to the number of robot characteristics per cluster. We thus estimated our research would be sufficiently powered to detect any plausible cluster structure because: a) we detected many robot characteristics (277; Supplementary Table 3); and b) based on previous research, we did not expect there would be many clusters (for example, in the IEEE definition[8], robots are defined using four main characteristics). Overall, considering the lack of guidelines on how many participants to test, we recruited a sample of 100 (Supplementary Table 1), which is comparable to other studies using hierarchical clustering[95,96].

Procedure

Sample 1. Participants first answered the consent form, after which they were presented with three items that elicited robot characteristics. In the following order, they were asked to: 1) state the first thing that comes to mind when they think about a robot; 2) define in their own words what a robot is; and 3) list as many characteristics they associate with robots as they could think of. We decided to use all three questions to elicit robot characteristics rather than only direct question 3 because we assumed that prompting participants in several different ways to think about robots would make it more likely that they would recall a larger number of different characteristics.

Then, all participants answered the understanding check (see the "Participants and Exclusion Criteria" section above). Thereafter, we exposed them to a dummy scale (approach and avoidance temperament questionnaire[97]) in which we embedded two instructed-response items (e.g., Please select "Disagree")[82–84] to further identify participants who were inattentive during the study. In addition, we measured their demographics (i.e., age and gender), as well as their employment status and use of robots at work (Supplementary Table 1). Finally, all participants answered the seriousness check[81].

Sample 2. After answering the consent form, participants were exposed to 277 different robot characteristics generated by Sample 1 (Supplementary Table 3) and were asked to sort them into groups based on similarity. In this regard, participants were provided with up to 60 empty boxes representing different groups into which they could drag the characteristics they perceived as similar. Four instructed-response items (e.g., please insert this item into Group 20)[82–84] were also embedded amongst the robot characteristics to reveal participants who responded inattentively. Thereafter, we measured their demographics (i.e., age and gender), employment status, and use of robots at work (Supplementary Table 1). At the end, all participants had to answer the seriousness check[81] and complete a CAPTCHA item[85].

Analytic Approach

We first aimed to extract all robot characteristics from the responses Sample 1 participants generated regarding the three questions described in the "Procedure" section above, and then rephrase those that were stated vaguely into a more precise formulation. Next, we planned to delete all characteristics that were identical and therefore redundant. However, we aimed to keep many characteristics that were not directly identical but were overlapping or similar to ensure that the possible content space of robot characteristics was sampled in detail. Then, we planned to subject the characteristics, as sorted into categories by Sample 2 participants, to a hierarchical cluster analysis for categorical data[98–100]. We aimed to compute the dissimilarity matrix using Gower's distance[101,102], generate the clusters using Ward's linkage method[103,104], and determine the optimal number of clusters via the mean silhouette width approach using the PAM algorithm[98,105,106]. The final step was to arrange the clusters that emerged into the robot definition (for the definition that was eventually developed using this procedure, see Supplementary Table 4).

Study 2

Participants and Exclusion Criteria

Similar to Study 1 (Sample 1), we initially recruited 70 participants via MTurk, and 67 of them remained after the exclusion criteria were applied (Supplementary Table 1). Participants were excluded if they did not pass a seriousness check[81] and an understanding check in which they were asked to identify the main task of the study amongst a series of dummy alternatives, similar to the previous study. A CAPTCHA item was also administered at the end to stop bots from completing the survey[85].

Determining Sample Size

When determining the sample size for Study 2, we followed similar guidelines as for Study 1 (Sample 1). Namely, given that several studies showed that sample sizes larger than 30-50 participants tend to result in data saturation in qualitative studies[87–92], we decided to recruit 70 participants (Supplementary Table 1) to account for potential exclusions and obtain at least 50 valid participants.

Procedure

After completing the consent form, participants were presented with the robot definition developed in Study 1. Upon reading it, they were asked to take a moment and think about different domains in which humans can encounter and/or interact with robots. It was explained that, by "domains," we mean any area of human life and human activity in which people encounter, interact with, use, are helped by, and/or are substituted by robots. They were provided with 15 blank fields in which they could enter their domain ideas. If they filled in all 15 blanks, they were given the opportunity to enter 10 additional domains. Then, participants answered the understanding check (see the "Participants and Exclusion Criteria" section), and we measured their demographics (i.e., age and gender), employment status, and use of robots at work (Supplementary Table 1). At the end, all participants had to answer the seriousness check[81] and complete a CAPTCHA item[85].

Analytic Approach

To identify the domains, we aimed to perform an inductive qualitative content analysis[86,107–110] on participants' responses by first creating a list of all domain items that participants identified and then arranging these items into common categories that correspond to the domains of robot use. To ensure no important domains are omitted, we also planned to consult the classification of robots proposed by the IEEE (https://robots.ieee.org/learn/types-of-robots/), the list of industries and sectors endorsed by the International Labor Organization (ILO; https://www.ilo.org/global/industries-and-sectors/lang--en/index.htm), and the papers from our literature review (see the "Supplementary Notes" section).

Phase Two: Developing the Taxonomy (i.e., Key Dimensions) of Psychological Processes

Study 3

Participants and Exclusion Criteria

We initially recruited 350 participants via MTurk, and 334 remained (Supplementary Table 1) after the exclusion criteria were applied. Participants were excluded if they did not pass a seriousness check[81] and an understanding check in which they were asked to identify the main task of the study amongst a series of dummy alternatives, similar to the previous studies. A CAPTCHA item was also administered at the end to stop bots from completing the study[85].

Determining Sample Size

We decided to recruit 350 participants to uncover an extensive range of psychological processes that would underpin the taxonomy developed in the subsequent studies. This number both greatly exceeds the sample size of 30-50 required for data saturation and is substantially larger than typical sample sizes in qualitative research[87–92,111].

Procedure

After completing the consent form and reading the instructions, each participant was randomly allocated to five of the 28 domains developed in Study 2 (Supplementary Table 5). For each domain, they first read the definition of robots created in Study 1 and then answered four questions that prompted them to engage in thinking about robots from this domain. In the following order,

participants were asked: 1) whether they personally had any interactions with robots from the domain in question; 2) whether they had been exposed to any examples of interactions between humans and robots in this domain (e.g., through the media or some other informational sources); 3) whether they were able to imagine interactions between a robot from the domain and themselves or someone else; and 4) to list at least three (but no more than five) examples of interactions between humans and robots in the domain.

To assess participants' psychological processes, we then asked them to list and describe feelings they experienced (for affective responses), thoughts they had (for cognitive responses), and actions they engaged in (for behavioral responses) when they interacted with any robots they could think of from each domain, or to write about feelings, thoughts, and actions they could conceive in case they had never interacted with these robots. Participants were not provided with specific robot examples for a given domain because we expected that relying on their own reflections and experiences would cover a broader spectrum of robots and therefore increase the diversity of psychological processes reported (for a similar methodological approach, see ref.[94]).

After responding to the questions regarding all five domains, participants answered the understanding check (see the "Participants and Exclusion Criteria" section). Then, we measured their demographics (i.e., age and gender), employment status, and use of robots at work (Supplementary Table 1). At the end, participants had to answer the seriousness[81] check and complete a CAPTCHA item[85].

Analytic Approach

To analyze the data, we aimed to use iterative categorization[112]. This qualitative analysis involves splitting participants' responses to questions assessing their psychological processes into key points (i.e., separate issues or thoughts, e.g., "I think this will be the future"), and then grouping these points into themes based on similarity.

Study 4

Item Development

To establish a representative item for each of the 149 psychological processes, we first developed several items that were based on the responses generated for each process in Study 3 (Supplementary Table 6). All authors then independently selected the most appropriate item, and in cases where no majority was reached, the final choice was achieved through discussion. All 149 items (Supplementary Table 6) were scored using a 7-point Likert scale from "1=Strongly disagree" to "7=Strongly agree" because this response type has desirable psychometric properties[113,114]. Overall, there are more items that capture cognitive and affective psychological processes than behaviors (Supplementary Table 6) because participants mostly evoked behaviors that were very specific to a robot domain in question (e.g., vacuuming the floor), and there were fewer examples that apply across all robots (e.g., avoiding a robot).

All items were phrased in the same direction, and none were reverse-worded. The latter items have previously been used to tackle acquiescence bias, but research has shown that they can produce methodological issues such as deceased internal consistency, factor structure contamination, etc[115–118]. In addition, they do not effectively reduce acquiescence bias; a more successful approach is to detect and exclude participants who respond carelessly[119]. For that reason, we implemented several attention checks to identify careless responders and exclude their data from analyses (for details, see the "Participants and Exclusion Criteria" section below).

Participants and Exclusion Criteria

Participants in both samples were recruited via Pureprofile. For Samples 1 and 2, 1668 and 1808 participants, respectively, completed the study, and 1528 and 1538 remained (Supplementary Table 1) after the exclusion criteria were applied. Identical checks were used in each sample. Four instructed-response items (e.g., Please respond with "Strongly agree" for this item)[82–84] were embedded amongst the questions measuring psychological processes. Participants who failed to correctly answer one of these checks were immediately screened out by Pureprofile, so they are not included in the participant count under "All Participants" in Supplementary Tables 1 and 2, which includes all participants who completed the study. From the participants who completed the study, we then excluded those who did not pass a seriousness check[81] and an understanding check in which they were asked to identify the main task of the study amongst a series of dummy alternatives, similar to Studies 1-3. A CAPTCHA item was administered at the end to stop bots from completing the survey[85].

Determining Sample Size

We consulted several resources to determine the number of participants to test for each sample because there is no consensus regarding sample size requirements for exploratory factor analysis (EFA[120–124]). First, a few resources posit that the ratio of the number of participants to the number of items should be at least 10:1[124–128]. Second, some studies estimated that, if the ratio of the number of items to the number of factors is larger than 10:3, recruiting approximately 400 participants leads to high power, even under low communalities[123]. Third, it has been proposed that a sample size larger than 300 is sufficient for a wide range of factor solutions[129,130].

We therefore concluded that it is necessary to recruit a sample of 1500 participants to include in statistical analyses after accounting for potential exclusions and missing data. Importantly, this number is consistent with the sample-to-item ratio criterion (10:1)[124–128], considering that we used 149 items in total. Moreover, the sample size meets the benchmark of >300[130], and the rule by MacCallum et al.[123] because we had no theoretical reason to believe that the item-to-factor ratio would be larger than 10:3 (which would correspond to more than 44 factors being extracted). The total number of participants who took part in the study in each sample (Supplementary Table 1) corresponds to the number that Pureprofile required to meet our sample size demands.

Procedure

After answering the consent form and reading the instructions, all participants were randomly allocated to a domain and received a specific example of a robot from that domain (Stimulus Set A in Supplementary Table 7). For the Sex domain, two robot examples were created (one male and one female), and participants assigned to this domain were therefore randomly allocated to one of the two examples (Stimulus Set A in Supplementary Table 7). For consistency, the descriptions of robots across all domains contained an image and eight lines of text. Participants were then asked to answer 149 items about the robot in question, presented in a randomized order, as well as the four instructed-response items[82–84]. Subsequently, they responded to the understanding check (see the "Participants and Exclusion Criteria" section), and we measured their demographics (i.e., age and gender), employment status, and use of robots at work (Supplementary Table 1). At the end, participants had to answer the seriousness check[81] and complete a CAPTCHA item[85].

Analytic Approach

For both samples, the choice of the best factor solution was based on a) statistical criteria; b) semantic criteria (i.e., whether the factors are easy to interpret conceptually); and c) precedents in previous taxonomic research.

In terms of statistical criteria, the first step was to show that our data for Samples 1 and 2 are suitable for EFAs by computing the Kaiser-Meyer-Olkin (KMO) measure of sampling adequacy and Bartlett's test of sphericity[131]. In this regard, to further proceed with EFAs, the KMO values had to be higher than 0.9, and Bartlett's test had to be statistically significant at $p < .05$[131].

The second step was to use the following analyses/criteria to determine the preliminary number of factors to examine in EFAs: parallel analysis[132,133], very simple structure[134], Velicer map[135], optimal coordinates[136], acceleration factor[136], Kaiser rule[137], and visual inspection of scree plots[138]. This is advisable because no single statistical criterion is best for selecting the most optimal factor solution[126–128], and consulting many different criteria allows understanding the range within which this solution potentially lies. In practice, especially when it comes to analyzing complex taxonomies, some criteria (e.g., parallel analysis) can greatly overestimate the optimal number of factors, whereas some criteria (e.g., very simple structure) may underestimate it, and the factor solutions that researchers eventually select lie somewhere in between[94,139].

The goal of the third step was to more stringently evaluate a range of different factor solutions to identify the one that is statistically most optimal. Statistical techniques used for this purpose were maximum likelihood (ML) EFAs[140] with an oblique rotation—promax[141,142] with Kaiser normalization[143]. This rotation was selected because it tends to produce a clean factor structure and is thus compatible with taxonomic research that involves many items and can suffer from interpretability issues[94,141]. For an optimal factor solution, we expected all factors to be valid (i.e., clearly defined and have little noise), as reflected in a sufficient number of items with high loadings and low cross-loadings[120,131]. More specifically, we expected all factors to meet the following benchmarks: 1) they should have at least 3 items with standardized loadings of .5 or higher; and 2) these items should not have cross-loadings of 0.32 or higher[120,131,144,145]. If a factor solution does not meet these benchmarks, it likely has too many factors, and fewer factors are therefore typically more appropriate[120,131]. In line with this rationale, our plan was to first evaluate, against the benchmarks, the factor solution estimated by the analysis from step 2 that indicated retaining the largest number of factors (in taxonomic research, this is typically parallel analysis[94]). If this solution was not satisfactory, we aimed to decrease the number of factors by one and evaluate this new factor solution. We planned to continue this procedure until we identified a factor solution that met the benchmarks. This solution was deemed the statistically most optimal one.

In terms of semantic criteria, it was important that the statistically most optimal solution also makes sense on a conceptual level by having factors that are easy to interpret and are coherent. Indeed, the number of factors to determine is not only a statistical but also a subjective decision based on factor interpretability, and it is recommended that researchers avoid selecting factor structures that are flawed semantically, even if they are statistically sound[126–128].

Finally, the statistical and semantic criteria that we used to identify the best factor solution were also guided by precedents in previous taxonomic research. Although we could not identify previous research on robots that is methodologically similar to the present research when it comes to determining the best factor solution, we did identify conceptually comparable studies from a different domain. These studies focused on developing two different taxonomies of psychological situation characteristics: CAPTION[94] and DIAMONDS[139]. In each case, participants were asked to rate a range of different situations on many characteristics (e.g., malicious, sentimental, enjoyable, humorous), similar to how our participants were asked to rate a range of different robots in relation to various psychological processes (e.g., empathy, creativity, anxiety). Importantly, the procedure to select the best factor structure (see Study 2 for CAPTION and Study 1 for DIAMONDS) was similar to our Study 4. For example, the researchers first computed several analyses to determine the optimal number of factors (e.g., parallel analysis, optimal coordinates), which resulted in a wide range of factors being recommended,

comparable to our results: 5-22 factors for CAPTION, and 3-17 for DIAMONDS. However, when EFAs with promax rotation were conducted to explore these factor structures, factors with only weak loadings and many high cross-loadings were identified. The researchers therefore started analyzing other structures with fever factors until they identified the ones that did not have any weak factors and that also made sense semantically: 7 factors were retained for CAPTION and 8 for DIAMONDS.

Study 5

Item Selection

To select a subset of items to validate the Positive-Negative-Competence (PNC) model from the pool of items evaluated in Study 4 (Supplementary Table 6), we relied on several statistical and conceptual criteria. Statistically, we focused on items that had cross-loadings smaller than .32[145,146], and whose average factor loading across Samples 1 and 2 (Study 4) was higher than .50 (e.g., if a factor had loadings of .675 and .743, its average loading was .709)[120,147–149].

Conceptually, for both the positive and negative dimensions, we aimed to select a diverse set of items that tap into both affective (e.g., “I associate this robot with enjoyment” and “This robot makes me feel anxious”) and cognitive (e.g., “I think this robot should have rights” and “This robot violates privacy (e.g., is too intrusive or invasive)”) psychological processes. In this context, we avoided selecting items that are direct opposites (e.g., “I find this robot pleasant” and “This robot makes me feel unpleasant”). We also included at least one behavioral item for both the positive (e.g., “I would like to engage with this robot”) and negative (e.g., “I would want to protect myself when interacting with this robot”) dimensions, considering that behavioral items are rare because most behaviors that participants generated in Study 3 could apply only to specific robots and were therefore not used in item creation. Finally, for the competence dimension, our main conceptual criterion was to select items that comprise diverse aspects of cognitive processes about competence because this domain generally did not contain affective and behavioral items.

The items we selected can be seen in Supplementary Table 6, where they are emphasized in bold. Before making a final decision about the items, we probed whether the selected ones would produce an acceptable fit by implementing the same exploratory structural equation models (ESEMs) we were planning to use in Study 5 (see the “Supplementary Results” section for this study). For both samples, fit indices showed good to excellent fit (Sample 1 – UK: $\chi^2(558) = 1953.820$, $p = <.001$, SRMR = .026, CFI = .939, RMSEA = .041, 90% CI [.039, .043]; Sample 2 – US: $\chi^2(558) = 1850.880$, $p = <.001$, SRMR = .025, CFI = .944, RMSEA = .039, 90% CI [.037, .041]. Therefore, the selected items were used for confirmatory analyses in Study 5.

Participants and Exclusion Criteria

Participants in both samples were recruited via Pureprofile. For Samples 1 and 2, 1200 and 1219 participants, respectively, completed the study, and 1107 and 1108 remained (Supplementary Table 1) after the exclusion criteria were applied. Identical checks were used in each sample. Two instructed-response items (e.g., Please respond with “Somewhat disagree” for this item)[82–84] were embedded amongst the items testing psychological processes. Participants who failed to correctly answer one of these checks were immediately screened out by Pureprofile, so they are not included in the participant count under “All Participants” in Supplementary Tables 1 and 2, which includes all participants who completed the study. From the participants who completed the study, we then excluded those who did not pass a seriousness check[81] and the understanding check identical to the one used in Study 4. A CAPTCHA item was administered at the end to stop bots from completing the survey[85].

Determining Sample Size

To determine the number of participants to test, we used Monte Carlo simulations[150] based on the data from Samples 1 and 2 (Study 4). In Mplus[151], we first applied to these data the ESEM models we were planning to use in Study 5 (see the "Supplementary Results" section for this study), and then utilized the parameters from these analyses to run the simulations. As our initial aim was to recruit a sample that would result in roughly 1100 participants included in analyses after accounting for exclusions and missing data, we examined whether this sample size was sufficiently powered to confirm the factor structure of the PNC model. The simulations showed that, for both models, the loadings of the selected items (Supplementary Table 6) on their corresponding factors had parameter biases lower than 10%, standard error biases lower than 5%, and coverage between 0.91 and 0.98, in line with the recommendations[150]. Importantly, the power to replicate each factor loading was 1.00, thus indicating that the sample size of 1100 would lead to highly powered research. The total number of participants who took part in the study in each sample (Supplementary Table 1) corresponds to the number that Pureprofile required to meet our sample size demands.

Procedure

After answering the consent form and reading the instructions, participants were randomly allocated to one robot example. The randomization procedure involved first randomly assigning a participant to one stimulus set (i.e., A or B; Supplementary Table 7), and then to one of the 28 domains from that stimulus set, for which they received the corresponding robot example (Supplementary Table 7). If participants were assigned to the Sex domain, they were randomly allocated to either the male or female robot example from that domain (Supplementary Table 7), in line with Study 4. The descriptions of robots were also consistent with the previous study and contained an image and eight lines of text. Participants were then asked to answer the 37 selected items (Supplementary Tables 6 and 8) in relation to the robot in question, presented in a randomized order, as well as the two instructed-response items[82–84]. Subsequently, they responded to the understanding check (see the "Participants and Exclusion Criteria" section), and we measured their demographics (i.e., age and gender), employment status, and use of robots at work (Supplementary Table 1). At the end, participants answered the seriousness check[81] and had to complete a CAPTCHA item[85].

Analytic Approach

To compute the models and assess their fit, we aimed to implement the MLR maximum likelihood estimator with robust standard errors[151,152] using exploratory structural equation modelling (ESEM[153]). This analytic technique was selected because it combines the best features of confirmatory factor analysis (CFA), structural equation modelling (SEM), and EFA: it allows confirming a priori factors while bypassing several assumptions of CFA that are not attainable for complex factor structures typical in taxonomic research (e.g., the requirement of zero cross-loadings)[94,154,155]. For ESEM models, target rotation with all cross-loadings specified as targets of zero was chosen[156,157]. The following fit criteria were used[158–160]: SRMR < .05 = excellent fit, SRMR between .05 and .08 = good fit, and SRMR > .08 = poor fit; CFI > .95 = excellent fit, CFI between .90 and .95 = good fit, and CFI < .90 = poor fit; RMSEA < .06 = excellent fit, RMSEA between .06 and .10 = good fit, and RMSEA > .10 = poor fit. For testing configural measurement invariance, the same fit criteria were used. For metric invariance, changes in SRMR, CFI, and RMSEA had to be ≤ .030, .010, and .015 respectively, and for scalar invariance, the changes had to be ≤ .015, .010, and .015 respectively[161].

Phase Three: Determining Main Individual Difference Predictors and Their Mechanisms

Study 6

Participants and Exclusion Criteria

Two thousand five hundred and five participants recruited via Pureprofile completed the study, and 2203 remained (Supplementary Table 1) after the exclusion criteria were applied. Concerning the exclusions, participants who failed to correctly answer one of the three instructed-response items[82–84] embedded amongst the Psychological Responses to Robots (PRR) scale and other measures (Supplementary Tables 8 and 11) were immediately screened out by Pureprofile, so they are not included in the participant count under “All Participants” in Supplementary Tables 1 and 2, which includes all participants who completed the study. From the participants who completed the study, we then excluded those who did not pass a seriousness check[81] and the understanding check identical as in Studies 4 and 5. A CAPTCHA item was administered at the end to stop bots from completing the survey[85].

Determining Sample Size

There are no clear guidelines concerning the use of machine learning algorithms combined with cross-validation regarding sample size and power. In a series of simulations, Song, Tang, and Wee[162] showed that, for the 10-fold cross-validations that we were planning to use, a sample size of 2000 leads to high generalizability (i.e., likelihood that the results will apply to other samples from the same population) without inflating the time taken to run the models. We therefore aimed to recruit a sample that would result in roughly 2200 participants after applying the exclusion criteria, in case of any additional missing data. The total number of participants who took part in the study (Supplementary Table 1) corresponds to the number that Pureprofile required to meet our sample size demands.

Procedure

After answering the consent form and reading the instructions, participants were randomly allocated to one robot example (Supplementary Table 7) using the same randomization procedure as in Study 5. The descriptions of robots were also consistent with the previous study and contained an image and eight lines of text. Participants were then asked to answer the 37 PRR items (Supplementary Table 8) concerning the allocated robot, presented in a randomized order, as well as one instructed-response item[82–84]. Subsequently, they responded to the understanding check (see the “Participants and Exclusion Criteria” section), and then completed the measures assessing four covariates (i.e., familiarity, frequency of interaction, descriptive norms, and injunctive norms; Supplementary Table 11) and all 79 individual differences (Supplementary Table 11) whose order was randomized. Two instructed-response items were embedded among the measures. Finally, we assessed participants’ gender, the remaining covariates (i.e., age, income, and political orientation; Supplementary Table 11), and some additional participant information (i.e., educational attainment, ethnic identity, relationship status, employment status, and use of robots at work; Supplementary Table 11). At the end, participants answered the seriousness check[81] and had to complete a CAPTCHA item to finish the study[85].

Analytic Approach

To select the most predictive individual differences, we developed a meticulous procedure that consisted of several steps. Using the caret package[163,164] in R, we first aimed to compute the following 11 machine learning models for the positive, negative, and cognitive dimensions separately: linear least squares, regularized regressions (i.e., ridge, lasso, and elastic net), k-nearest neighbors, regression trees, conditional inference trees, random forest, conditional random forest, neural networks, and neural networks with a principal component step. To train the models, 10-fold cross-validation was selected[165–

[169]. In each model, the aim was to use all variables from Supplementary Table 11 as predictors, and a PNC dimension assessed via the PRR scale (Supplementary Table 8) as the dependent variable.

Then, for each dependent variable, we planned to identify the most predictive model by using the root-mean-square error (RMSE) as the criterion[163–165]. We would then compare this model to all other computed models by using paired samples t-tests to identify any models that were not significantly different (i.e., worse) from the most predictive model. As the significance criterion for the t-test, we used a conservative Bonferroni corrected p-value of .00167 (i.e., α = .05 divided by 30 comparisons) to avoid rejecting any models that were not convincingly different from the one that had the best fit. For each of the selected models (i.e., the most predictive one and the models that were not different from it), we planned to identify the 30 most important predictors by using the VarImp function in R[164], and then select those predictors that appeared among the top 30 across all the selected models.

Finally, we aimed to undertake an additional step to ensure the robustness of all the predictors that remained. The VarImp function orders predictors according to importance, but it does not provide meaningful output regarding whether and which ones are likely to be false positives (type I error). Therefore, based on the linear least squares model, which is in essence a linear regression algorithm combined with cross-validation and thus outputs statistical significance values, we decided to retain only those individual differences that reach the final stage and are also statistically significant after applying the false discovery rate (FDR[170]) correction. In this context, we preferred FDR over Bonferroni to avoid the increased risk of type II error[171], given the large number of significance tests in each model (i.e., 86; see Supplementary Tables 13-15, and pp.37-42). Overall, this final step was important because in Study 7 we were planning to use linear regression-based mediation analyses[172] to probe the mechanism behind the individual differences and PNC dimensions. Therefore, we wanted to minimize the chances that any retained predictors would not replicate in that setup.

Study 7

Participants and Exclusion Criteria

One thousand one hundred and sixteen participants recruited via Prolific completed the study, and 1071 remained (Supplementary Table 1) after the exclusion criteria were applied. Participants were excluded if they did not accurately answer three instructed-response items[82–84], two in Wave 1 and one in Wave 2, and two seriousness checks[81] administered at the end of each wave. Participants also had to complete a CAPTCHA item at the end of both waves to finish the survey[85].

Determining Sample Size

Considering that the analyses we were aiming to compute in the present study to test the relationships between the main individual difference predictors and the PNC dimensions (Supplementary Table 16) involved the variables that were also tested in Study 6, we relied on the data from that study to estimate the sample size for Study 7. Namey, our objective was to first compute the same nine linear regression models we were planning to use in the present study (Supplementary Table 16) on the data from Study 6 to identify which one of the relationships between the PNC dimensions and the main individual difference predictors produced the smallest effect size, and then compute the sample size necessary to replicate this effect assuming a high power of 0.99 and a significance level of 0.01. This level was determined using the Benjamini-Yekutieli (B-Y[173,174]) adjustment for multiple tests that was calculated based on the 66 significance tests we aimed to compute (i.e., 9 tests probing the relationships between the key individual differences and the PNC dimensions, see Supplementary Table 16; and 57 tests of mediated effects, see Supplementary Table 17). Overall, the smallest effect size was associated

with the relationship between ERQ_ES (Supplementary Table 11) and the negative dimension (Cohen's f^2 of 0.03100701). An a priori power analysis in G* Power[175] showed that 779 participants should be tested to replicate this effect. To ensure this sample size is obtained despite the expected attrition between Waves 1 and 2 and the exclusion criteria, we decided to recruit 1200 participants for Wave 1 (for further rationale, see the preregistration using this link: https://osf.io/nejvm?view_only=79b6eeee42e24cb2a977927712bdcdd2). Overall, this number led to more than enough participants being included in analyses (Supplementary Table 1). Sensitivity power calculations concerning a relationship between an individual difference predictor and a PNC dimension based on linear regressions further showed that, with 1071 participants who were used in statistical analyses (Supplementary Table 1) and assuming the aforementioned significance level of 0.01, the study had a power of 0.99 to capture Cohen's f^2 0.0225081, a power of 0.95 to capture Cohen's f^2 of 0.0166850, and a power of 0.90 to capture Cohen's f^2 of 0.0139362[175]. Therefore, the study was highly powered to capture small effects concerning the relationships between individual difference predictors and PNC dimensions[176].

Procedure

The study consisted of two waves. In Wave 1, participants first completed the consent form and were then presented, in a randomized order, with the measures assessing the key individual differences: GRP, IDAQ, FMPS_PE, PANAS_TNA, SD3_P, ERQ_ES, PVQ5X_SS, and ATQ_AP (for details regarding these variables, see Supplementary Table 11). Two instructed-response items[82–84] were embedded within these measures. Subsequently, we assessed age and gender, and collected information about participants' employment status and use of robots at work (Supplementary Table 1). Finally, they were asked to answer the seriousness check[81] and CAPTCHA[85].

Approximately four days after completing wave 1, participants were invited to participate in wave 2. They first completed the consent form and were then presented with the items measuring the mediators (Supplementary Table 17) in a randomized order. Subsequently, they were randomly allocated to one robot example (Supplementary Table 7) using the same randomization procedure as in Studies 5 and 6. The descriptions of robots were also consistent with the previous studies and contained an image and eight lines of text. Participants were then asked to answer the 37 PRR scale items (Supplementary Table 8) concerning the allocated robot, presented in a randomized order, as well as one instructed-response item[82–84]. Finally, they responded to the seriousness check[81] and a CAPTCHA item[85].

Analytic Approach

To test whether the key individual differences predicted the relevant PNC dimensions, we aimed to use linear regressions, one per predictor (Supplementary Table 16). Moreover, to identify the most important mediators, we aimed to use the Process package (Model 4[172]) to conduct parallel mediation analyses (i.e., with all potential mediators analyzed together for the relevant predictor; Supplementary Table 17), percentile-bootstrapped with 10,000 samples. In line with the Benjamini-Yekutieli (B-Y) correction[173,174], the significance criterion was .01 for the regression analyses, whereas for the mediated effects we used 99% confidence intervals as the equivalent of this criterion.

Supplementary Results

Extended Results for Studies 1-7

Phase One: Mapping a Comprehensive Content Space of Robots From All Domains of Human Activity

Study 1

Computing and Interpreting the Clusters of Robot Characteristics

In line with the analytic strategy (see the "Supplementary Methods" section for Study 1), our initial objective was to extract all unique (i.e., non-redundant) robot characteristics from participants' answers in Sample 1. We first identified all the characteristics they mentioned in response to each of the three questions we asked about robots (i.e., writing down the first thing that comes to their mind when thinking about robots; defining what a robot is; and listing robot characteristics) (note. Because some participants who were excluded from analyses due to not passing the check items—see the "Participants and Exclusion Criteria" section for Study 1, Sample 1—gave meaningful responses in relation to these questions, we also considered these responses when extracting the unique robot characteristics). We then rephrased all items that were stated vaguely (e.g., "appearance of thought") into a more precise formulation (e.g., "appears to think on its own"). Next, we deleted all characteristics that were directly synonymous and therefore redundant. However, in the final list of characteristics, we purposely included many items that were overlapping or similar (e.g., "performs actions" and "performs certain actions") to ensure that the list comprehensively covers a large portion of the possible space of robot characteristics. This final list can be seen in Supplementary Table 3 (see also Supplementary Figure 1), whereas participants' raw responses can be accessed via OSF (see https://osf.io/2ntdy/?view_only=2cacc7b1cf2141cf8c343f3ee28dab1d).

The robot characteristics, as sorted into common categories by Sample 2 participants, were then subjected to a hierarchical cluster analysis for categorical data[98–100]. A dissimilarity matrix was first computed using Gower's distance[101,102]. Clusters were then produced using Ward's linkage method[103,104], and the optimal number of clusters was determined using the mean silhouette width approach implemented via the PAM algorithm[98,105,106]. The clusters and the robot characteristics per cluster are presented in Supplementary Table 3, whereas the dendrogram produced by the cluster analysis is available in Supplementary Figure 1.

As seen in Supplementary Table 3, Cluster 1 is the largest and most complex one. It broadly deals with the theme of robots' degree of similarity to humans in terms of the level of agency (e.g., being autonomous, self-sufficient, artificially intelligent, and able to do things on its own versus being controllable and remotely controlled), appearance (e.g., human-like and humanoid versus animal-like and artificial), durability (e.g., doesn't need a pension or sickness benefit, doesn't get tired), and the ability to pursue human duties and tasks (e.g., human substitute, helps to build cars, replicates human actions). Cluster 2 comprises positive robot characteristics that indicate robots are competent at what they do and can be used to the benefit of humans (e.g., quick, rational, precise, competent, intelligent, mobile, exciting, and accurate). Cluster 3 captures characteristics that encompass the constitution and makeup of robots (comprised of hardware, comprised of software, man-made, has sensors, has wheels, and has wires). Cluster 4 deals with the theme of negative robot characteristics that tend to indicate the non-human nature of robots and danger for humans (e.g., soulless, awkward movements, bland, boring, creepy, dangerous, error prone, emotionless). Finally, Cluster 5 refers to a robot's ability to perform some kind of tasks (e.g., performs complex actions, performs boring tasks, performs multiple tasks, performs household tasks).

Using the Clusters to Construct a General Definition of Robots

We developed the definition (Supplementary Table 4) by linking the themes of each of the five clusters obtained via hierarchical cluster analysis (Supplementary Table 3). It is important to emphasize that we did not form the definition by always translating an individual cluster theme into a separate part of the definition, given that from a narrative perspective, the definition had a better flow if certain elements were combined in the same parts. Therefore, Part 1 of the definition was informed by Clusters 1, 4, and 5 because it emphasized robots' non-living nature (Cluster 4) as well as their ability to substitute and help humans (Cluster 1) by performing various types of tasks (Cluster 5). Part 2 was informed by Cluster 1 through the emphasis on robots' degree of agency. Part 3 was also grounded in Cluster 1 because it conveyed the difference between humans and robots in terms of durability. Part 4 was informed by Clusters 2 and 4 because it conveyed that people can perceive robots positively (Cluster 2), but they can also attribute negative characteristics to robots, generally because of their non-human nature (Cluster 4). This implies that robots can be perceived negatively in cases where people attribute a lack of humanness to them, but it does not mean that robots who are very different from humans are always seen negatively. Indeed, robots can have many different appearances and abilities, and robots who are less (vs. more) like humans might sometimes be more accepted by users[177,178]. Finally, Part 5 combined Clusters 1 and 3 because it described the makeup of robots (Cluster 3) and their potential degree of similarity to humans in terms of appearance (Cluster 1).

Study 2

To identify the domains, we performed an inductive qualitative content analysis on participants' responses[86,107–110], in line with the analytic strategy (see the "Supplementary Methods" section for Study 2). More precisely, we first created a list of all domain items that participants identified (see pp.28-36). Then, we started arranging these items into common categories that corresponded to the domains of robot use. The first author created the initial list of categories from the domain items. Then, the list was revised by the second and third authors, and eventually it was consolidated into the final version by all three authors.

Subsequently, to ensure our list covers all important domains, we consulted the classification of robots proposed by the IEEE (https://robots.ieee.org/learn/types-of-robots/), the list of industries and sectors endorsed by the ILO (https://www.ilo.org/global/industries-and-sectors/lang--en/index.htm), and all the references we cited in the literature review (see the "Supplementary Notes" section). Although the domains we developed did not directly correspond to the IEEE and ILO categories, our domains did cover the content proposed by these resources, and they also went beyond. However, after consulting the articles from our literature review, we decided to add two new domains that the list derived from participants' responses was missing: sex robots[28,179] and art[2,4,180]. The final list of 28 domains, as well as the representative items our participants generated, can be seen in Supplementary Table 5, whereas all participants' items are available on pp.28-36. As our aim was to develop domains that are narrow rather than broad, some overlap between them may exist. This approach was necessary for our objective of establishing a comprehensive content space of all robots to avoid using a biased stimulus sample[181,182] when developing the taxonomy of psychological responses to representations of robots. In that respect, it was more optimal to lean toward having too many rather than too few domains to reduce the chance of failing to cover the content space of all robots in detail and omitting important types of robots.

Phase Two: Developing the Taxonomy (i.e., Key Dimensions) of Psychological Processes

Study 3

To develop a comprehensive list of psychological processes, we implemented iterative categorization using the procedure described by Neale[112], as indicated in the analytic strategy (see the "Supplementary Methods" section for Study 3). Data for each participant regarding the key questions that prompted them to write about these processes (i.e., listing and describing feelings they experienced, thoughts they had, and actions they engaged in regarding a robot from the domain in question; see the "Supplementary Methods" section for Study 3) were first transferred from the original dataset into the coding file and assigned individual codes. Then, we separated the response of each participant into key points (i.e., separate issues or thoughts, e.g., "I think this will be the future"). Irrelevant points, including those that were meaningless or that merely repeated the name of the domain in question, were labelled in red and excluded from further analyses. Out of 334 participants who were included in analyses (Supplementary Table 1), only 4 produced merely meaningless responses that could not be analyzed, and the remaining 330 participants generated 10332 valid key points (approximately 31 per participant) that were analyzed.

Once all responses were rearranged into key points, we started grouping these points into themes based on similarity in a different file. Eventually, all key points were allocated to various themes that corresponded to separate psychological processes. Some of these processes were rare and identified by only a few participants (e.g., embarrassment, disappointment), whereas others were common (e.g., trust, safety). However, we did not take their frequency of occurrence into account because our objective was to capture their variety rather than their prevalence, since the latter may be relative and subject to various contextual factors. Overall, 149 psychological processes were identified (Supplementary Table 6; all original coding files can be accessed via OSF using the following link: https://osf.io/2ntdy/?view_only=2cacc7b1cf2141cf8c343f3ee28dab1d). We omitted an additional category that was identified (i.e., sex/intimate intercourse) because participants evoked it only in relation to sex robots and it would not be suitable to assess it in relation to many different types of robots in subsequent studies.

Study 4

In line with the analytic strategy (see the "Supplementary Methods" section for Study 4), we first computed the Kaiser-Meyer-Olkin (KMO) measure of sampling adequacy and Bartlett's test of sphericity to ensure the data were suitable for EFAs. For Samples 1 and 2, KMO values were .983 and .984, respectively, and Bartlett's test was significant, χ^2 (11026) = 163884.191, $p < .001$, and χ^2 (11026) = 169896.554, $p < .001$. The results therefore provided convincing evidence regarding the suitability of our data for EFAs[131].

Second, to determine the preliminary number of factors for examining in EFAs, we consulted parallel analysis[132,133], very simple structure[134], Velicer map[135], optimal coordinates[136], acceleration factor[136], Kaiser rule[137], and visual inspection of scree plots[138], which indicated that extracting anywhere between 1-19 factors (Sample 1) and 2-18 factors (Sample 2) could be optimal. Next, we evaluated the largest factor solutions (i.e., 19 factors for Sample 1 and 18 for Sample 2) against several statistical and semantic benchmarks. If the benchmarks were not met, we decreased the number of factors by one and evaluated these new solutions. This procedure was continued until the benchmarks were met. Concerning statistical benchmarks, a factor solution had to produce only valid factors: those that have at least 3 items with standardized loadings ≥ .5 and cross-loadings < .32[120,131,144,145]. Semantically, a solution had to make sense conceptually by having factors that are coherent and easy to interpret[127,128].

For both Samples 1 and 2, three-factor solutions (Supplementary Table 6) were identified as the most optimal ones, and none of the larger factor solutions met the statistical criteria indicated above. As can be seen in Supplementary Table 6, beyond meeting the statistical criteria, the three-factor solutions had semantically coherent factors that denoted positive, negative, and competence-related psychological processes. Hence, the taxonomy was labelled the Positive-Negative-Competence (PNC) model of psychological processes regarding robots. The Positive and Negative factors explained the largest proportion of variance for each sample, whereas Competence was somewhat weaker and contained fewer items (Supplementary Table 6). Most items across the two samples had similar loadings and belonged to the same factors (Supplementary Table 6), thus indicating that both samples produced approximately identical insights regarding the structure of psychological responses to robots.

Study 5

Exploratory Structural Equation Modelling (ESEM)

Fitting the Models. In line with the analytic approach (see the "Supplementary Methods" section for Study 5), we fit the models using Mplus and implemented the MLR maximum likelihood estimator with robust standard errors[151–153]. Target rotation with all cross-loadings specified as targets of zero was used[156,157]. As shown in Supplementary Table 8; ESEM models for both samples met the criteria for good to excellent fit[158–160]. Furthermore, items that were previously classified under a specific dimension (Positive, Negative, or Competence) by EFAs in Study 4 (Supplementary Table 6) had the highest loadings for this dimension, whereas the cross-loadings with other factors were in all cases smaller than .32[120,146]. All dimensions also yielded good to excellent Cronbach's α values (Sample 1—Positive: α = .927; Negative: α = .943; Competence: α = .818; Sample 2—Positive: α = .923; Negative: α = .943; Competence: α = .802). Overall, Study 5 confirmed the structure of the PNC model as measured via the PRR scale.

Testing Alternative Factor Structures. To ensure that the model comprising three dimensions (Supplementary Table 8) is the most appropriate one, we tested several other factor structures. First, we wanted to ensure that the positive and competence dimensions were not redundant, considering that they both comprised positive evaluations of robots and loaded positively onto each other (Supplementary Table 8). We therefore computed an ESEM model in which these two dimensions were treated as the same factor, whereas the negative dimension was a separate factor. This model, however, produced a poor fit on some of the indices and was therefore rejected (Sample 1 – UK: $\chi^2(593)$ = 3245.221, p = <.001, SRMR = .052, CFI = .859, RMSEA = .064, 90% CI [.061, .066]; Sample 2 – US: $\chi^2(593)$ = 2987.629, p = <.001, SRMR = .051, CFI = .864, RMSEA = .060, 90% CI [.058, .063]).

Second, considering that all three PNC dimensions (Supplementary Table 8) in their essence deal with evaluations of robots, we wanted to eliminate the possibility that they comprise a single general factor rather than separate dimensions. To test this, we computed a bifactor ESEM model[183,184], which had a good to excellent fit in both samples (Sample 1 – UK: $\chi^2(524)$ = 1591.732, p = <.001, SRMR = .024, CFI = .943, RMSEA = .043, 90% CI [.041, .045]; Sample 2 – US: $\chi^2(524)$ = 1476.231, p = <.001, SRMR = .024, CFI = .946, RMSEA = .041, 90% CI [.038, .043]). However, for both samples, the explained common variance (ECV) was substantially below the cut-off value of .70 that would indicate the existence of a general factor ($ECV_{Sample\ 1}$ = .386; $ECV_{Sample\ 2}$ = .337)[185–188]. Overall, evidence did not support the existence of other plausible dimensionalities of our taxonomy.

Measurement Invariance

To show that the PNC model has equivalent factor structure, loadings, and intercepts regardless of participants' country, robot examples used, and several key participant characteristics, we tested configural, metric, and scalar measurement invariance[161,189,190]. All ESEM models used to test invariance were computed using Mplus and implemented the MLR estimator and the target rotation with all cross-loadings as targets of zero[151–153].

As shown in Supplementary Table 9, measurement invariance was demonstrated in all cases, given that the configural model demonstrated good to excellent fit (SRMR < .05, CFI > .90, RMSEA < .06[158–160]), and changes in SRMR, CFI, and RMSEA were ≤ .030, .010, and .015 respectively for the metric model, and ≤ .015, .010, and .015 for the scalar model[161]. Because we could not analyse measurement invariance for participants who did vs. did not use robots at work in Study 5, since the number of those who did not was insufficient (Supplementary Table 1), we tested this for Study 6 where the sample sizes were larger. For Study 6, we also computed measurement invariance for additional participant characteristics assessed in that study (educational attainment; income; political orientation—liberal vs. conservative; ethnic identity; and being in a relationship; Supplementary Table 1). Measurement invariance was demonstrated in all these cases (Supplementary Table 10).

Phase Three: Determining Main Individual Difference Predictors and Their Mechanisms

Study 6

All analyses were computed in line with the analytic strategy (see the "Supplementary Methods" section for Study 6); Supplementary Table 12 summarizes findings for the most predictive machine learning models and their 30 most important predictors. As can be seen from the table, in each of the three domains, the most predictive models were conditional random forest ($RMSE_{Positive}$ = 0.919; $RMSE_{Negative}$ = 0.988; $RMSE_{Competence}$ = 0.778), linear least squares ($RMSE_{Positive}$ = 0.929; $RMSE_{Negative}$ = 1.000; $RMSE_{Competence}$ = 0.795), ridge ($RMSE_{Positive}$ = 0.921; $RMSE_{Negative}$ = 0.994; $RMSE_{Competence}$ = 0.787), lasso ($RMSE_{Positive}$ = 0.921; $RMSE_{Negative}$ = 0.993; $RMSE_{Competence}$ = 0.784), elastic net ($RMSE_{Positive}$ = 0.921; $RMSE_{Negative}$ = 0.993; $RMSE_{Competence}$ = 0.784), and random forest ($RMSE_{Positive}$ = 0.925; $RMSE_{Negative}$ = 0.995; $RMSE_{Competence}$ = 0.781). Conditional random forest was therefore the most predictive model across all the domains but did not significantly differ from the other five models (all *p*s ≥ .008; for the significance criterion, we used a conservative Bonferroni corrected p-value of .00167, as indicated in the analytic approach in the "Supplementary Methods" section for Study 6).

The most predictive individual differences for the positive dimension were general risk propensity (GRP[191]), anthropomorphism (IDAQ[46]), and parental expectations (FMPS_PE[192]); for the negative dimension, they were trait negative affect (PANAS_TNA[193]), psychopathy (SD3_P[194]), anthropomorphism (IDAQ[46]), and expressive suppression (ERQ_ES[195]); and for the competence dimension, they were approach temperament (ATQ_AP[97]) and security-societal (PVQ5X_SS[196]) (Supplementary Table 12). These individual differences were among the 30 most important predictors based on the VarImp function[164] (Supplementary Table 12), and they also remained statistically significant in the linear least squares models after applying the FDR correction[170] (the coefficients and significance values for these models are available in Supplementary Tables 13-15, whereas the FDR corrections can be seen on pp.37-42). As shown in Supplementary Table 12, several covariates also passed this stringent test and were therefore robust as predictors. However, these variables are not the focus of our analysis because they were included in the models to ensure the key results for the individual differences are not confounded.

To further aid the interpretability of the findings, here we report the direction of the linear relationships between the most predictive individual differences and the PNC dimensions as identified by the linear least squares model, given that the output of this model is more intuitive and easily interpretable

compared to the others. General risk propensity (GRP[191]), anthropomorphism (IDAQ[46]), and parental expectations (FMPS_PE[192]) positively predicted people's positive psychological processes regarding robots. Moreover, trait negative affect (PANAS_TNA[193]), psychopathy (SD3_P[194]), anthropomorphism (IDAQ[46]), and expressive suppression (ERQ_ES[195]) positively predicted the negative psychological processes. Finally, approach temperament (ATQ_AP[97]) and security societal (PVQ5X_SS[196]) positively predicted the competence-related processes.

Study 7

Testing the Relationships between Individual Differences and PNC Dimensions

Each of the nine relationships between the key individual differences as predictors and the corresponding PNC dimensions as dependent variables was tested using a linear regression, in line with the analytic approach (see the "Supplementary Methods" section for Study 7) and preregistration (https://osf.io/nejvm?view_only=79b6eeee42e24cb2a977927712bdcdd2). The significance criterion was .01, in line with the B-Y correction[173,174]. As indicated in Supplementary Table 16, the predictor that yielded the highest effect size for the positive dimension was anthropomorphism (Cohen's f^2 = 0.058). For the negative dimension, it was psychopathy (Cohen's f^2 = 0.035). Finally, for the competence dimension, it was approach temperament (Cohen's f^2 = 0.027).

Testing Mediated Effects

To identify the most important mediators, we used the Process package (Model 4[172]) to conduct parallel mediation analyses (i.e., with all potential mediators included in the analyses together), percentile-bootstrapped with 10,000 samples. Parallel mediations were used because they allow identifying mediators that produce the largest effects while accounting for correlations between all tested mediators[172]. Considering that the mediation analyses generate bootstrapped confidence intervals rather than p-values, we used the 99% confidence intervals for all mediation analyses, which corresponds to the significance criterion of 0.01 established via the B-Y correction[173,174]. All mediators and the corresponding mediated effects are available in Supplementary Table 17, whereas the full output of all mediation analyses is presented on pp.43-74. To further aid interpretation of the mechanisms, here we summarize the mediated effects from Supplementary Table 17 that successfully explained a portion of the relationship between the key individual differences and PNC dimensions.

For the positive dimension, GRP[191] was a positive predictor because people higher on this trait valued the risks associated with robot adoption (GRP_M3) and were curious to see how robots would change the world (GRP_M4). Moreover, IDAQ[46] was a positive predictor because people higher on this trait generally felt positive toward inanimate entities with human features (IDAQ_M3), and because interacting with such entities helped them fulfil the need to experience strong emotions regularly (IDAQ_M2). Finally, FMPS_PE[192] was a positive predictor due to being associated with valuing robots because they were closer to perfection than humans (FMPS_PE_M1), because they could help humans fulfill their own high expectations (FMPS_PE_M2), and because they could help humans cope with their own high expectations of themselves (FMPS_PE_M6).

Concerning the negative dimension, PANAS_TNA[193] was a positive predictor because people high on this trait were more likely to be in a state of activated displeasure (e.g., feeling scared and upset; 12-PAC_AD[197]). Moreover, SD3_P was a positive predictor because people high on this trait were also more likely to be in the state of activated displeasure (12-PAC_AD[197]), and they tended to have negative feelings toward other people's inventions (SD3_P_M2) and felt inferior toward technologies they were

not proficient in (SD3_P_M3). For ERQ_ES[195] and IDAQ[46], we did not manage to explain the mechanism behind their relationship with the negative dimension.

Finally, concerning the competence dimension, ATQ_AP[97] was a positive predictor because people high on this trait were more likely to value exceptional skills and competencies (ATQ_AP_M5). Moreover, PVQ5X_SS[196] was a positive predictor because it was associated with people linking advanced technology (e.g., robots, machines) with how powerful society is (PVQ5X_SS_M4).

Additional Analysis Output

Hierarchical Cluster Analysis (Study 1, Sample 2)

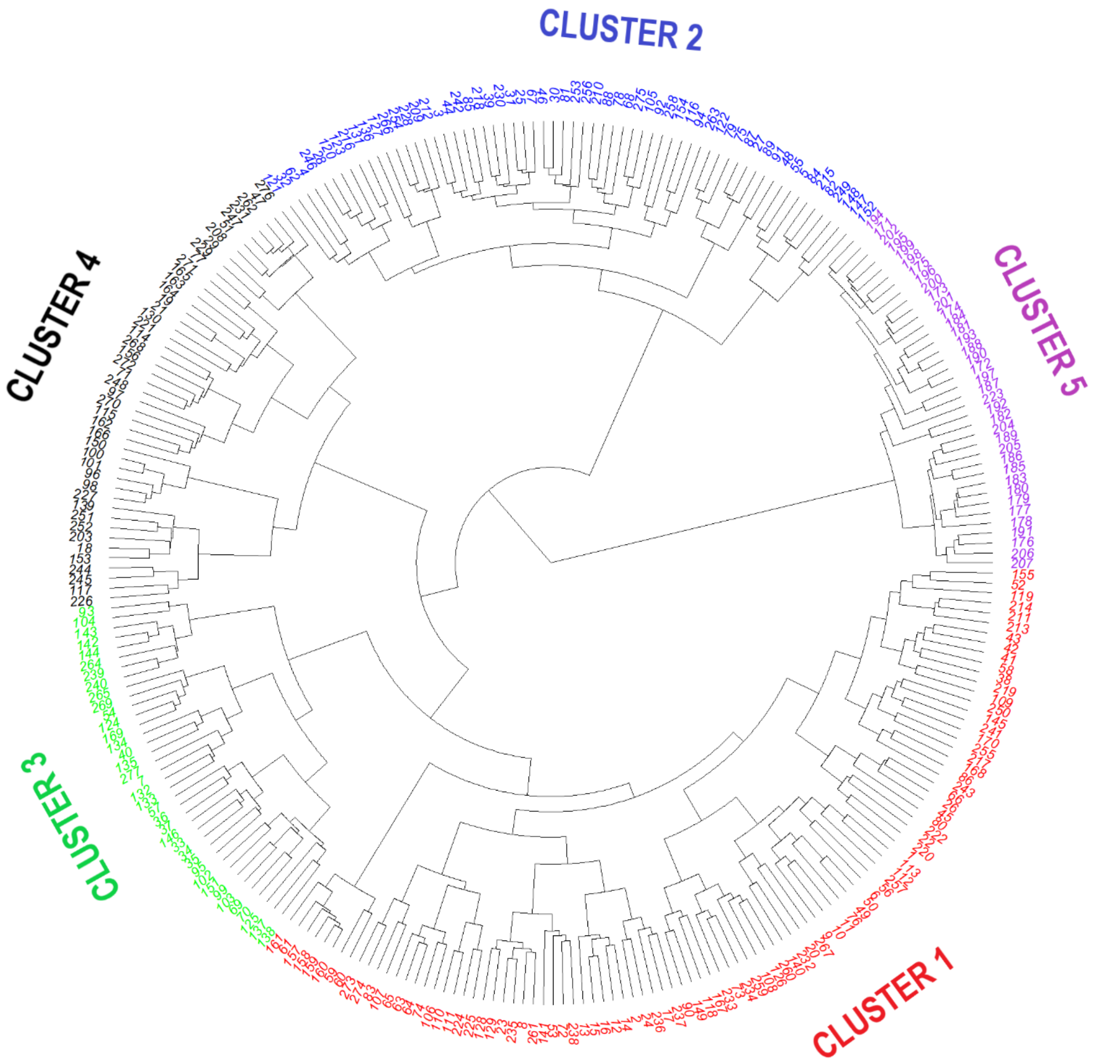

Supplementary Figure 1. Polar Dendrogram for Hierarchical Cluster Analysis (Study 1). Robot characteristics to which the numbers in the dendrogram correspond can be seen on the next page.

1. ability to add parts
2. able to move things on its own
3. accurate
4. adaptable
5. agile
6. animal-like
7. apparatus
8. appears to think on its own
9. artificial
10. artificial intelligence
11. artificial life form
12. automated
13. automated movement
14. automatic
15. automation
16. automaton
17. autonomous
18. awkward movements
19. bland
20. boredom relief
21. boring
22. can be 'scrapped' once no longer needed
23. can learn
24. can produce somethings without having to wait for humans
25. capable
26. cheap
27. cheerful
28. clever
29. cold
30. committed
31. competent
32. complex
33. comprised of hardware
34. comprised of software
35. computer
36. computer driven
37. computerised
38. conditioned
39. consistent
40. constructed
41. control
42. controllable
43. controlled
44. correct
45. cost efficient
46. credible
47. creepy
48. cute
49. cyberman
50. cyborg
51. dangerous
52. dependant on power supply
53. developmental
54. device
55. dexterous
56. different
57. digital
58. directed
59. doesn't get bored
60. doesn't get tired
61. doesn't need a pension or sickness benefit
62. dynamic
63. eases everyday life problems
64. eases everyday tasks
65. eases human tasks
66. economic
67. efficient
68. effortless
69. electric
70. electronic
71. emotionless
72. enabling senses
73. engineered
74. enhances human interaction
75. entertaining
76. entity
77. error prone
78. essential
79. exciting
80. expensive
81. faithful
82. fast
83. fixes things
84. flexible
85. focused
86. follows order
87. friendly
88. functional
89. funny
90. futuristic
91. great-looking
92. handy
93. hard
94. hard-working
95. has computing power
96. has digital voice
97. has no memories

98. has sci-fi voice (slow, chunky, shiny, computerised)
99. has sensors
100. has strange voice
101. has weird voice
102. has wheels
103. has wires
104. heavy
105. helpful
106. helps humans
107. helps to build cars
108. hi-tech
109. human operator
110. human substitute
111. human task substitute
112. human-like
113. humanoid
114. impersonal
115. inanimate
116. inexhaustible
117. inflexible
118. innovative
119. insular
120. intelligent
121. interactive
122. interesting
123. intuitive
124. item
125. jointed
126. latest technology
127. leading
128. learns
129. learns certain actions
130. limited
131. logical
132. machine
133. machinery
134. man-made
135. manufactured
136. masculine
137. mechanical capability
138. mechanical limbs
139. mechanical movements
140. media reference (e.g., books, tv shows, films)
141. memory control
142. metal-like
143. metal-made
144. metallic
145. methodical
146. micro-chip
147. mobile
148. mobility
149. modern
150. monotone
151. motorised
152. moves
153. moves weirdly
154. multipurpose
155. needs default settings
156. no conscience
157. no holidays
158. no meals
159. no sick leave - within reason (maintenance / repair)
160. no sickness
161. no strikes
162. non-human
163. non-humorous
164. non-intelligent
165. non-interactive
166. non-living
167. novel
168. obedient
169. object
170. passive
171. performs actions
172. performs boring tasks
173. performs certain actions
174. performs certain functions
175. performs certain tasks
176. performs certain tasks independently
177. performs complex actions
178. performs complex movements
179. performs complex tasks
180. performs complicated tasks
181. performs everyday tasks
182. performs hard work over long periods of time
183. performs high quality tasks
184. performs household tasks
185. performs human actions
186. performs human tasks
187. performs human tasks more efficiently
188. performs manual tasks
189. performs many tasks
190. performs monotonous tasks
191. performs multiple tasks
192. performs non-stop

193. performs physical tasks
194. performs predetermined actions
195. performs predetermined tasks
196. performs repetitive tasks
197. performs repetitive tasks efficiently
198. performs routine tasks
199. performs set tasks
200. performs specific actions
201. performs specific tasks
202. performs specifically designed tasks
203. performs stiff movements
204. performs tasks
205. performs tasks electronically
206. performs tasks independently
207. performs tasks without human help
208. potentially dangerous
209. powerful
210. practical
211. pre-programmed
212. precise
213. programmable
214. programmed
215. quick
216. rational
217. regimented
218. reliable
219. remotely controlled
220. repairable
221. repetitive
222. replacement
223. replicates everyday tasks
224. replicates human actions
225. replicates human movement
226. rigid
227. robotic voice
228. robust
229. rude
230. safe
231. scary
232. sci-fi
233. scientific
234. scientifically engineered
235. self-learning
236. self-sufficient
237. semi-autonomous
238. sensing
239. shiny
240. silver
241. simple
242. skilful
243. slave
244. slow
245. slow-moving
246. smart
247. sometimes scary
248. soulless
249. speedy
250. standard
251. stiff
252. stiff movement
253. straightforward
254. strong
255. structured
256. supporting
257. talks
258. task-oriented
259. technical
260. technology
261. thinks
262. threatening
263. time-saving
264. tinny
265. tool
266. tough
267. toy
268. unaware
269. unit
270. unreal
271. unreliable
272. unthoughtful
273. used in factories
274. used in production lines
275. useful
276. worrying
277. mechanical

Participants' Items Categorized into Robot Domains (Study 2)

Note. The items are in the original shape as written by participants and therefore in some cases contain grammatical and spelling errors.

1. **Health and human care and wellbeing (e.g., medical, surgical, fitness, lab diagnostics, elderly, disability, infant/child, and personal care)**

- Assisted living for elderly patients
- Elder care in home
- Disability assistance
- Take care of babies
- Personal care
- At the doctor or hospital
- Dentist
- Dr. office
- Healthcare
- Hospitals
- In hospitals, surgeries
- Making health decisions
- Medical care
- In hospitals cleaning bots
- Medical world
- Medicine
- Care of youth
- Lab diagnostics
- Nurse
- Surgery
- Exercise
- Fitness
- Sleeping
- Helping motivate people to exercise and encourage them while exercising
- "Babysitting" by watching children sleep and alerting parents in the other room if they have woken (works well for infants and toddlers, not older kids)

2. **Social and companionship**

- Social media
- Use as a companion
- Companionship
- Social life

3. **Sex** (**Note.** This domain was added based on Bendel[2] and was not generated from participants' items)

4. **Animal care (e.g., walking pets)**

- Animal care
- Walking pets

- Walking the dog

5. **Security and surveillance**

- Home monitoring
- Night watchmen
- Public safety
- Security

6. **Policing and military**

- Swat team alternatives/aids
- Warfare
- Battle bots
- Bomb detonator
- Police training
- Policing robots
- Protection/military/law enforcement
- During an emergency rescue situation
- Military
- Military and field work
- Military applications
- Search and rescue
- Law enforcement
- Parking lot attendant/"police"

7. **Education, libraries, and knowledge/information management and gathering**

- Ask for directions
- Child education
- Computer and technology assistance
- Educational (school)
- Finding information
- Information
- Information kiosks
- Asking about the weather
- Library
- Navigational resources
- Studying
- Study for them
- University/universities
- Internet
- Online

8. **Research and exploration within science, technology, engineering, and mathematics (stem) (e.g., ocean exploration, supercomputing, IT innovation, space discovery)**

- Research
- Research/exploration
- Science

- Space exploration
- Technology
- Working with space exploration like the Mars rover
- Google's super computers
- Computation
- Computers/computer programs
- Exploration
- Space station arm
- Electronic control
- Academic labs
- Programming
- Programming data
- Resource collection
- IT
- Anazlying
- Do big calculations

9. Communication tools and channels

- Answering service
- Calling
- Calling a business
- Calling into a government building dealing with automatic system
- Chat rooms online
- Chatbots
- Communications
- Contacting company over siping orders
- Customer service
- Customer service desk
- Cell phone
- Phone
- Smart phone
- Using our phone
- Through AI on phones
- Interacting with tele-routing botsautomatic
- Online customer support chat
- Phone customer support chat
- Telephone call

10. Leisure, recreation, and travel

- Travel agency
- Travel
- Traveling
- Watercraft
- Recreation

11. Culture/entertainment, gaming, toys, and other amusement

- Amusement park
- Animatronics such as those at Chuck E' Cheese's
- Casino
- Child entertainment
- Computer games
- Concerts
- Disney World's Hall of Presidents
- Entertainment
- Gaming
- In a theme park
- In an arcade
- Jukebox
- Kids toys
- Movie theater
- Museums
- Play video games
- Sporting events
- Sports venues
- Theatre
- Tour guides
- Toys
- Various attractions at theme parks

12. Workplace domain (i.e., to aid or replace human effort)

- Robots that process documents
- Work product generation (collation of documentation for example)
- At work
- While working
- Work
- Workplace
- Work in place of humans
- Working long shifts
- Robots can hold jobs

13. Dangerous and/or risky work

- Do dangerous work

14. Inspection, repair, and/or improvement of products, engines, equipment, technology, and/or infrastructure (e.g., buildings, bridges, roads, power supplies, nuclear reactors, pipes, gas mains)

- Repairing items
- Repairs
- Auto repair
- Auto maintenance

15. Agriculture (e.g., harvesting, farms)

- Agriculture

- Farms
- Field harvesting

16. Household chores/tasks and domestic help/assistance (i.e., inside and outside of the home)

- Assistant
- Automatic vacuum
- Cleaner/cleaning
- Cleaning at home or in an office
- Cleaning robots such as roomba
- Cleaning service
- Cleaning the house
- Cleanliness of home
- Domestic
- Gauge the temperature and adjust it
- Giving out house orders for the smart house
- Heat the oven
- Home
- Home (cleaning, etc.)
- Home cleaning
- Home cleaning robots
- Home maintenance
- Home repair
- Home services
- House chores
- House cleaning
- Household chores
- Janitor/housekeeping
- Kitchen
- Lawn mower
- Do yard work for them
- Landscaping
- Lawnmower robot (like a Roomba for the lawn)
- Maid
- Personal helper
- Helping
- Providing assistance
- Roomba
- Smart assistant
- Writing for them
- Set an appointment
- Day to day tasks
- Smart home features like Nest and alarms
- Talking to Siri or Alexa
- Using their home assistant
- Car washes
- Vacuum

- Vacuum robots
- Vacuum the floor
- While controlling things in their home

17. Industry
- Factory/factories
- Factory assembly
- Factory floor
- Lifting heavy items
- Welding like car factories
- Working in factories; assemblers
- Working in factories; pipe threaders
- Working in factories; spot welders

18. Hospitality and food service (i.e., hotels, conventions, restaurants, bars, and other lodging, space, food and/or drink provider) and related customer service and support
- Bartenders in restaurants/bar
- Bartending
- Bell hop
- Checking you in for a service, such as a hotel or an airport
- Convention center
- Cooking
- Dining
- Eating out
- Fast food restaurants
- Food preparation
- Food service
- Food service and going out
- Front desk associate at hotels
- Hospitality
- Hotel desk
- Hotel service
- Hotels
- In a restaurant/diner
- Meal prep
- Ordering at a fast food restaurant
- Ordering food
- Ordering from fast food
- Preparing/serving food
- Reception
- Restaurants
- Restaurant service
- Service
- Service situations
- Robots that make food
- Servers in restaurants

- Service (hotel, food, etc)
- Serving food and drinks
- Cleaning crew at hotels/other places
- Car rental
- Some food is prepared by robots in certain restaurants
- Use it at a fast food chain to take orders
- Use it at a restaurant as a waiter or waitress.
- While cooking

19. Banking/financial services and related customer service and support

- Talking to you bank teller
- Accuracy in money handling
- ATM
- Bank
- Bank teller
- Banking
- Business
- Checkout counters
- Do their banking needs for them
- Financial institutions

20. Retail and commerce and related customer service and support

- Buying groceries
- Getting groceries
- Going to a store and shopping for you
- Grocery store
- Kiosks
- Online ordering
- Online shopping
- Ordering food at a kiosk at a restaurant
- Retail
- Self checkout at the grocery store
- Self-checkout
- Selling items such as clothing or other goods
- Shopping
- Specialty stores
- Store
- In a store (cleaning, checking out, etc.)
- Going to the store
- Supermarket
- Swiping a credit card
- Telemarketing
- Vending machine
- Walmart, self check out
- While out shopping
- While shopping

- Back room department store
- Department stores
- Checking out in a grocery store
- At the supermarket to pick up spills and check people out

21. Construction
- Building construction
- Construction
- Construction sites
- Demolition

22. Manufacturing
- Manufactoring in factories
- Manufacturing
- Manufacturing facilities
- Technician

23. Mining
- Mining

24. Warehouses and fulfilment centers
- Counting items in inventory like Walmart
- Use it at a supermarket to stock goods overnight.
- Warehouse work
- Warehouses
- Warehouses, e.g., amazon fullfillment centers
- Warehousing
- Sorting things in a warehouse
- Stocking
- Storage rooms

25. Public services (e.g., road work and other shared public good)
- Roadwork

26. Transportation (i.e., land, water, and/or sky) of goods, people, and other living entities, transport equipment, and delivery/courier/shipping services
- Air travel
- Aircraft
- Automated transportation
- Autonomous driving
- Car/cars
- Certain vehicles have robot aspects
- City bus
- Dmv
- Drive for them
- Drone delivery

- Flying in an airplane
- Garaging parking
- Subway
- Courier services
- Delivery services
- Driving
- Drones
- Helping with delivery
- Mail like amazon or usps
- On the road
- Public transportations
- Robot delivery service
- Self-driving cars and trucks
- Shipping
- Some delivery bots in offices and hotels
- Walking down the street
- Bus station
- Transport
- Transportation
- Transportation hubs
- While driving

27. Airports

- Airports
- Use it at an airport to help people or give information.

28. Art (**Note.** This domain was added based on Herath and Kroos[4] and was not generated from participants' items)

False Discovery Rate (FDR) Correction by Benjamini and Hochberg[170] Performed on the P-Values from the Linear Least Squares Model Presented in Supplementary Table 13 (Study 6)

Note. As can be seen in the analysis output below (see the yellow highlighted text), any p-values (i.e., column "p") for which the number in the "test" column is 1 remained significant after the FDR correction was applied.

p	i	q	m	crit	test
.985	86	.05	86	.050000	0
.967	85	.05	86	.049419	0
.908	84	.05	86	.048837	0
.905	83	.05	86	.048256	0
.888	82	.05	86	.047674	0
.884	81	.05	86	.047093	0
.871	80	.05	86	.046512	0
.869	79	.05	86	.045930	0
.839	78	.05	86	.045349	0
.830	77	.05	86	.044767	0
.766	76	.05	86	.044186	0
.716	75	.05	86	.043605	0
.692	74	.05	86	.043023	0
.690	73	.05	86	.042442	0
.676	72	.05	86	.041860	0
.670	71	.05	86	.041279	0
.592	70	.05	86	.040698	0
.568	69	.05	86	.040116	0
.542	68	.05	86	.039535	0
.530	67	.05	86	.038953	0
.524	66	.05	86	.038372	0
.523	65	.05	86	.037791	0
.521	64	.05	86	.037209	0
.489	63	.05	86	.036628	0
.462	62	.05	86	.036047	0
.451	61	.05	86	.035465	0
.414	60	.05	86	.034884	0
.400	59	.05	86	.034302	0
.399	58	.05	86	.033721	0
.388	57	.05	86	.033140	0
.387	56	.05	86	.032558	0
.346	55	.05	86	.031977	0
.329	54	.05	86	.031395	0
.317	53	.05	86	.030814	0
.290	52	.05	86	.030233	0
.282	51	.05	86	.029651	0
.275	50	.05	86	.029070	0
.273	49	.05	86	.028488	0
.259	48	.05	86	.027907	0
.255	47	.05	86	.027326	0
.255	46	.05	86	.026744	0
.247	45	.05	86	.026163	0
.243	44	.05	86	.025581	0
.226	43	.05	86	.025000	0
.208	42	.05	86	.024419	0
.202	41	.05	86	.023837	0

.181	40	.05	86	.023256	0
.177	39	.05	86	.022674	0
.161	38	.05	86	.022093	0
.158	37	.05	86	.021512	0
.130	36	.05	86	.020930	0
.127	35	.05	86	.020349	0
.117	34	.05	86	.019767	0
.107	33	.05	86	.019186	0
.105	32	.05	86	.018605	0
.088	31	.05	86	.018023	0
.087	30	.05	86	.017442	0
.072	29	.05	86	.016860	0
.060	28	.05	86	.016279	0
.057	27	.05	86	.015698	0
.054	26	.05	86	.015116	0
.053	25	.05	86	.014535	0
.052	24	.05	86	.013953	0
.039	23	.05	86	.013372	0
.034	22	.05	86	.012791	0
.033	21	.05	86	.012209	0
.028	20	.05	86	.011628	0
.023	19	.05	86	.011047	0
.020	18	.05	86	.010465	0
.018	17	.05	86	.009884	0
.015	16	.05	86	.009302	0
.015	15	.05	86	.008721	0
.011	14	.05	86	.008140	0
.011	13	.05	86	.007558	0
.007	12	.05	86	.006977	1
.004	11	.05	86	.006395	1
.004	10	.05	86	.005814	1
.004	9	.05	86	.005233	1
.002	8	.05	86	.004651	1
.001	7	.05	86	.004070	1
.001	6	.05	86	.003488	1
<.001	5	.05	86	.002907	1
<.001	4	.05	86	.002326	1
<.001	3	.05	86	.001744	1
<.001	2	.05	86	.001163	1
<.001	1	.05	86	.000581	1

Number of cases read: 86   Number of cases listed: 86

False Discovery Rate (FDR) correction by Benjamini and Hochberg[170] Performed on the P-Values from the Linear Least Squares Model Presented in Supplementary Table 14 (Study 6)

Note. As can be seen in the analysis output below (see the yellow highlighted text), any p-values (i.e., column "p") for which the number in the "test" column is 1 remained significant after the FDR correction was applied.

p	i	q	m	crit	test
.995	86	.05	86	.050000	0
.992	85	.05	86	.049419	0
.992	84	.05	86	.048837	0
.992	83	.05	86	.048256	0
.932	82	.05	86	.047674	0
.887	81	.05	86	.047093	0
.883	80	.05	86	.046512	0
.876	79	.05	86	.045930	0
.870	78	.05	86	.045349	0
.866	77	.05	86	.044767	0
.864	76	.05	86	.044186	0
.849	75	.05	86	.043605	0
.831	74	.05	86	.043023	0
.813	73	.05	86	.042442	0
.796	72	.05	86	.041860	0
.779	71	.05	86	.041279	0
.712	70	.05	86	.040698	0
.659	69	.05	86	.040116	0
.631	68	.05	86	.039535	0
.583	67	.05	86	.038953	0
.583	66	.05	86	.038372	0
.547	65	.05	86	.037791	0
.527	64	.05	86	.037209	0
.520	63	.05	86	.036628	0
.517	62	.05	86	.036047	0
.516	61	.05	86	.035465	0
.494	60	.05	86	.034884	0
.492	59	.05	86	.034302	0
.477	58	.05	86	.033721	0
.471	57	.05	86	.033140	0
.468	56	.05	86	.032558	0
.430	55	.05	86	.031977	0
.423	54	.05	86	.031395	0
.409	53	.05	86	.030814	0
.403	52	.05	86	.030233	0
.394	51	.05	86	.029651	0
.355	50	.05	86	.029070	0
.354	49	.05	86	.028488	0
.349	48	.05	86	.027907	0
.344	47	.05	86	.027326	0
.313	46	.05	86	.026744	0
.289	45	.05	86	.026163	0
.288	44	.05	86	.025581	0
.286	43	.05	86	.025000	0
.285	42	.05	86	.024419	0
.239	41	.05	86	.023837	0

.221	40	.05	86	.023256	0
.221	39	.05	86	.022674	0
.212	38	.05	86	.022093	0
.207	37	.05	86	.021512	0
.201	36	.05	86	.020930	0
.191	35	.05	86	.020349	0
.149	34	.05	86	.019767	0
.138	33	.05	86	.019186	0
.135	32	.05	86	.018605	0
.129	31	.05	86	.018023	0
.125	30	.05	86	.017442	0
.125	29	.05	86	.016860	0
.125	28	.05	86	.016279	0
.106	27	.05	86	.015698	0
.104	26	.05	86	.015116	0
.099	25	.05	86	.014535	0
.094	24	.05	86	.013953	0
.086	23	.05	86	.013372	0
.085	22	.05	86	.012791	0
.083	21	.05	86	.012209	0
.082	20	.05	86	.011628	0
.077	19	.05	86	.011047	0
.058	18	.05	86	.010465	0
.047	17	.05	86	.009884	0
.038	16	.05	86	.009302	0
.033	15	.05	86	.008721	0
.030	14	.05	86	.008140	0
.024	13	.05	86	.007558	0
.014	12	.05	86	.006977	0
.010	11	.05	86	.006395	0
.007	10	.05	86	.005814	0
.005	9	.05	86	.005233	1
.003	8	.05	86	.004651	1
.002	7	.05	86	.004070	1
.001	6	.05	86	.003488	1
<.001	5	.05	86	.002907	1
<.001	4	.05	86	.002326	1
<.001	3	.05	86	.001744	1
<.001	2	.05	86	.001163	1
<.001	1	.05	86	.000581	1

Number of cases read: 86   Number of cases listed: 86

False Discovery Rate (FDR) correction by Benjamini and Hochberg[170] Performed on the P-Values from the Linear Least Squares Model Presented in Supplementary Table 15 (Study 6)

Note. As can be seen in the analysis output below (see the yellow highlighted text), any p-values (i.e., column "p") for which the number in the "test" column is 1 remained significant after the FDR correction was applied.

p	i	q	m	crit	test
.991	86	.05	86	.050000	0
.960	85	.05	86	.049419	0
.959	84	.05	86	.048837	0
.946	83	.05	86	.048256	0
.939	82	.05	86	.047674	0
.922	81	.05	86	.047093	0
.908	80	.05	86	.046512	0
.879	79	.05	86	.045930	0
.877	78	.05	86	.045349	0
.873	77	.05	86	.044767	0
.869	76	.05	86	.044186	0
.860	75	.05	86	.043605	0
.808	74	.05	86	.043023	0
.803	73	.05	86	.042442	0
.801	72	.05	86	.041860	0
.800	71	.05	86	.041279	0
.794	70	.05	86	.040698	0
.785	69	.05	86	.040116	0
.775	68	.05	86	.039535	0
.761	67	.05	86	.038953	0
.744	66	.05	86	.038372	0
.736	65	.05	86	.037791	0
.722	64	.05	86	.037209	0
.694	63	.05	86	.036628	0
.691	62	.05	86	.036047	0
.680	61	.05	86	.035465	0
.621	60	.05	86	.034884	0
.602	59	.05	86	.034302	0
.566	58	.05	86	.033721	0
.544	57	.05	86	.033140	0
.540	56	.05	86	.032558	0
.540	55	.05	86	.031977	0
.530	54	.05	86	.031395	0
.510	53	.05	86	.030814	0
.503	52	.05	86	.030233	0
.486	51	.05	86	.029651	0
.485	50	.05	86	.029070	0
.444	49	.05	86	.028488	0
.415	48	.05	86	.027907	0
.408	47	.05	86	.027326	0
.374	46	.05	86	.026744	0
.370	45	.05	86	.026163	0
.369	44	.05	86	.025581	0
.364	43	.05	86	.025000	0
.331	42	.05	86	.024419	0
.322	41	.05	86	.023837	0

.318	40	.05	86	.023256	0
.304	39	.05	86	.022674	0
.294	38	.05	86	.022093	0
.287	37	.05	86	.021512	0
.285	36	.05	86	.020930	0
.280	35	.05	86	.020349	0
.279	34	.05	86	.019767	0
.234	33	.05	86	.019186	0
.227	32	.05	86	.018605	0
.201	31	.05	86	.018023	0
.198	30	.05	86	.017442	0
.196	29	.05	86	.016860	0
.193	28	.05	86	.016279	0
.193	27	.05	86	.015698	0
.184	26	.05	86	.015116	0
.160	25	.05	86	.014535	0
.147	24	.05	86	.013953	0
.140	23	.05	86	.013372	0
.134	22	.05	86	.012791	0
.110	21	.05	86	.012209	0
.098	20	.05	86	.011628	0
.090	19	.05	86	.011047	0
.077	18	.05	86	.010465	0
.066	17	.05	86	.009884	0
.047	16	.05	86	.009302	0
.043	15	.05	86	.008721	0
.034	14	.05	86	.008140	0
.028	13	.05	86	.007558	0
.021	12	.05	86	.006977	0
.019	11	.05	86	.006395	0
.012	10	.05	86	.005814	0
.011	9	.05	86	.005233	0
.008	8	.05	86	.004651	0
.005	7	.05	86	.004070	0
.005	6	.05	86	.003488	0
.002	5	.05	86	.002907	1
.002	4	.05	86	.002326	1
<.001	3	.05	86	.001744	1
<.001	2	.05	86	.001163	1
<.001	1	.05	86	.000581	1

Number of cases read: 86 Number of cases listed: 86

Full Output of All Mediation Analyses (Study 7)

Positive Dimension: Predictor—GRP[191]

Note. Yellow highlighted text in the analysis output below shows significant mediated effects. "PROCESS Procedure for SPSS Version 3.4.1" in the analysis output refers to the version of the Process[172] package used to compute the analysis, whereas the version of SPSS[198] used to run the package was 23.

```
Run MATRIX procedure:

**************** PROCESS Procedure for SPSS Version 3.4.1 ****************

          Written by Andrew F. Hayes, Ph.D.       www.afhayes.com
    Documentation available in Hayes (2018). www.guilford.com/p/hayes3

**************************************************************************
Model  : 4
    Y  : Positive
    X  : GRP
   M1  : GRP_M1
   M2  : GRP_M2
   M3  : GRP_M3
   M4  : GRP_M4
   M5  : GRP_M5
   M6  : GRP_M6
   M7  : GRP_M7

Sample
Size:  1071

**************************************************************************
OUTCOME VARIABLE:
 GRP_M1

Model Summary
              R         R-sq          MSE            F          df1          df2            p
      .02472165     .00061116   2.49673196     .65372972   1.00000000  1069.00000000     .41896225

Model
                  coeff           se            t            p         LLCI         ULCI
constant     4.55785843     .12141376  37.53988500     .00000000   4.24455795   4.87115890
GRP          -.04044500     .05002253   -.80853554     .41896225   -.16952497    .08863497

**************************************************************************

OUTCOME VARIABLE:
```

GRP_M2

Model Summary

R	R-sq	MSE	F	df1	df2	p
.00171794	.00000295	2.69648841	.00315497	1.00000000	1069.00000000	.95521756

Model

	coeff	se	t	p	LLCI	ULCI
constant	3.92907146	.12617729	31.13929067	.00000000	3.60347897	4.25466395
GRP	.00291996	.05198512	.05616914	.95521756	-.13122433	.13706424

****

OUTCOME VARIABLE:

GRP_M3

Model Summary

R	R-sq	MSE	F	df1	df2	p
.18472369	.03412284	2.12716218	37.76599929	1.00000000	1069.00000000	.00000000

Model

	coeff	se	t	p	LLCI	ULCI
constant	2.95166117	.11206816	26.33808941	.00000000	2.66247641	3.24084592
GRP	.28374651	.04617214	6.14540473	.00000000	.16460223	.40289079

****

OUTCOME VARIABLE:

GRP_M4

Model Summary

R	R-sq	MSE	F	df1	df2	p
.10157437	.01031735	2.13451109	11.14422826	1.00000000	1069.00000000	.00087200

Model

	coeff	se	t	p	LLCI	ULCI
constant	4.95306355	.11226158	44.12073719	.00000000	4.66337969	5.24274742
GRP	.15440236	.04625183	3.33829721	.00087200	.03505245	.27375227

****

OUTCOME VARIABLE:

GRP_M5

Model Summary

R	R-sq	MSE	F	df1	df2	p
.09599937	.00921588	1.76937462	9.94341282	1.00000000	1069.00000000	.00165924

Model

	coeff	se	t	p	LLCI	ULCI
constant	4.76497207	.10220963	46.61959866	.00000000	4.50122661	5.02871753
GRP	.13278755	.04211043	3.15331775	.00165924	.02412428	.24145083

****

OUTCOME VARIABLE:
GRP_M6

Model Summary

R	R-sq	MSE	F	df1	df2	p
.02928629	.00085769	1.98837435	.91765437	1.00000000	1069.00000000	.33830821

Model

	coeff	se	t	p	LLCI	ULCI
constant	4.69841723	.10835052	43.36312484	.00000000	4.41882559	4.97800886
GRP	.04276302	.04464048	.95794278	.33830821	-.07242889	.15795493

****
OUTCOME VARIABLE:
GRP_M7

Model Summary

R	R-sq	MSE	F	df1	df2	p
.07011657	.00491633	1.43103047	5.28152590	1.00000000	1069.00000000	.02174519

Model

	coeff	se	t	p	LLCI	ULCI
constant	4.56621424	.09191923	49.67637657	.00000000	4.32902251	4.80340597
GRP	.08703299	.03787077	2.29815707	.02174519	-.01069014	.18475611

****
OUTCOME VARIABLE:
Positive

Model Summary

R	R-sq	MSE	F	df1	df2	p
.51757064	.26787936	.92843642	48.57257606	8.00000000	1062.00000000	.00000000

Model

	coeff	se	t	p	LLCI	ULCI
constant	.80589107	.15300632	5.26704428	.00000017	.41106333	1.20071880
GRP	.05737087	.03121156	1.83812883	.06632259	-.02316953	.13791126
GRP_M1	-.01759820	.02824211	-.62311905	.53334008	-.09047603	.05527963
GRP_M2	.09053071	.02744201	3.29898184	.00100258	.01971750	.16134392
GRP_M3	.20220124	.02246626	9.00021976	.00000000	.14422781	.26017467
GRP_M4	.08403334	.02780321	3.02243241	.00256765	.01228806	.15577861
GRP_M5	-.02392861	.03050188	-.78449622	.43292395	-.10263769	.05478048
GRP_M6	-.01636600	.03275139	-.49970401	.61738702	-.10087987	.06814787
GRP_M7	.22649444	.03187856	7.10491436	.00000000	.14423287	.30875601

****************** DIRECT AND INDIRECT EFFECTS OF X ON Y *****************

Direct effect of X on Y

Effect	se	t	p	LLCI	ULCI
.05737087	.03121156	1.83812883	.06632259	-.02316953	.13791126

```
Indirect effect(s) of X on Y:
              Effect       BootSE     BootLLCI     BootULCI
TOTAL      .08716015    .02053662    .03522201    .14200561
GRP_M1     .00071176    .00235164   -.00599949    .00982639
GRP_M2     .00026435    .00528757   -.01552914    .01557147
GRP_M3     .05737390    .01224818    .02922639    .09268108
GRP_M4     .01297495    .00612556    .00086354    .03191612
GRP_M5    -.00317742    .00463946   -.01772598    .00917248
GRP_M6    -.00069986    .00243458   -.01002859    .00631420
GRP_M7     .01971249    .00985734   -.00404113    .04847412

*********************** ANALYSIS NOTES AND ERRORS ************************

Level of confidence for all confidence intervals in output:
  99.0000

Number of bootstrap samples for percentile bootstrap confidence intervals:
  10000

NOTE: Variables names longer than eight characters can produce incorrect output.
      Shorter variable names are recommended.

------ END MATRIX -----
```

Positive Dimension: Predictor—IDAQ[46]

Note. Yellow highlighted text in the analysis output below shows significant mediated effects. "PROCESS Procedure for SPSS Version 3.4.1" in the analysis output refers to the version of the Process[172] package used to compute the analysis, whereas the version of SPSS[198] used to run the package was 23.

```
Run MATRIX procedure:

**************** PROCESS Procedure for SPSS Version 3.4.1 ****************

          Written by Andrew F. Hayes, Ph.D.       www.afhayes.com
    Documentation available in Hayes (2018). www.guilford.com/p/hayes3

**************************************************************************
Model  : 4
    Y  : Positive
    X  : IDAQ
   M1  : IDAQ_M1
   M2  : IDAQ_M2
   M3  : IDAQ_M3
   M4  : IDAQ_M4

Sample
Size:  1071

**************************************************************************
OUTCOME VARIABLE:
 IDAQ_M1

Model Summary
              R         R-sq          MSE            F          df1          df2            p
      .16515477      .02727610     2.77811175    29.97576937     1.00000000  1069.00000000      .00000005

Model
                 coeff           se            t            p         LLCI         ULCI
constant     2.89315879      .10425720    27.75020701      .00000000     2.62412972     3.16218786
IDAQ          .18905569      .03453064     5.47501318      .00000005      .09995158      .27815980

**************************************************************************
OUTCOME VARIABLE:
 IDAQ_M2

Model Summary
              R         R-sq          MSE            F          df1          df2            p
      .15536281      .02413760     2.62780565    26.44132833     1.00000000  1069.00000000      .00000032
```

```
Model
                coeff          se           t           p        LLCI        ULCI
constant   2.47408825   .10139763 24.39986245   .00000000  2.21243811  2.73573839
IDAQ        .17269032   .03358353  5.14211322   .00000032   .08603015   .25935048

**************************************************************************
OUTCOME VARIABLE:
 IDAQ_M3

Model Summary
          R        R-sq         MSE           F         df1         df2           p
   .14611527   .02134967  2.04551005 23.32068794  1.00000000 1069.00000000   .00000157

Model
                coeff          se           t           p        LLCI        ULCI
constant   3.58382287   .08946064 40.06033165   .00000000  3.35297538  3.81467036
IDAQ        .14308734   .02962992  4.82914982   .00000157   .06662921   .21954548

**************************************************************************
OUTCOME VARIABLE:
 IDAQ_M4

Model Summary
          R        R-sq         MSE           F         df1         df2           p
   .02478106   .00061410  2.30220689   .65687740  1.00000000 1069.00000000   .41784490

Model
                coeff          se           t           p        LLCI        ULCI
constant   3.11473717   .09490812 32.81844830   .00000000  2.86983280  3.35964153
IDAQ       -.02547675   .03143416  -.81047973   .41784490  -.10659061   .05563711

**************************************************************************
OUTCOME VARIABLE:
 Positive

Model Summary
          R        R-sq         MSE           F         df1         df2           p
   .47863912   .22909540   .97486631 63.29878029  5.00000000 1065.00000000   .00000000

Model
                coeff          se           t           p        LLCI        ULCI
constant   1.42679932   .14605754  9.76874821   .00000000  1.04990459  1.80369404
IDAQ        .11863217   .02085922  5.68727605   .00000002   .06480590   .17245843
IDAQ_M1     .02027045   .02271790   .89226758   .37245108  -.03835204   .07889293
IDAQ_M2     .12329054   .02239582  5.50507023   .00000005   .06549917   .18108191
IDAQ_M3     .24546617   .02712807  9.04841897   .00000000   .17546343   .31546890
IDAQ_M4     .03877720   .02238621  1.73219098   .08352909  -.01898939   .09654378

****************** DIRECT AND INDIRECT EFFECTS OF X ON Y *****************

Direct effect of X on Y
```

Effect	se	t	p	LLCI	ULCI
.11863217	.02085922	5.68727605	.00000002	.06480590	.17245843

Indirect effect(s) of X on Y:

	Effect	BootSE	BootLLCI	BootULCI
TOTAL	.05925851	.01077660	.03279534	.08831712
IDAQ_M1	.00383224	.00520635	-.00949311	.01871139
IDAQ_M2	.02129108	.00603569	.00802882	.03937993
IDAQ_M3	.03512310	.00845757	.01567182	.05975113
IDAQ_M4	-.00098792	.00165385	-.00666157	.00359096

********************** ANALYSIS NOTES AND ERRORS ************************

Level of confidence for all confidence intervals in output:
99.0000

Number of bootstrap samples for percentile bootstrap confidence intervals:
10000

NOTE: Variables names longer than eight characters can produce incorrect output.
Shorter variable names are recommended.

------ END MATRIX -----

Positive Dimension: Predictor—FMPS_PE[192]

Note. Yellow highlighted text in the analysis output below shows significant mediated effects. "PROCESS Procedure for SPSS Version 3.4.1" in the analysis output refers to the version of the Process[172] package used to compute the analysis, whereas the version of SPSS[198] used to run the package was 23.

```
Run MATRIX procedure:

**************** PROCESS Procedure for SPSS Version 3.4.1 ****************

          Written by Andrew F. Hayes, Ph.D.       www.afhayes.com
    Documentation available in Hayes (2018). www.guilford.com/p/hayes3

**************************************************************************
Model  : 4
    Y  : Positive
    X  : FMPS_PE
   M1  : FMPS_PE_M1
   M2  : FMPS_PE_M2
   M3  : FMPS_PE_M3
   M4  : FMPS_PE_M4
   M5  : FMPS_PE_M5
   M6  : FMPS_PE_M6

Sample
Size:  1071

**************************************************************************
OUTCOME VARIABLE:
 FMPS_PE_M1

Model Summary
              R         R-sq          MSE            F          df1          df2            p
      .12526223    .01569063   2.41129441  17.04065855   1.00000000 1069.00000000    .00003944

Model
                  coeff           se            t            p         LLCI         ULCI
constant     2.12195987    .14898470  14.24280394    .00000000   1.73751433   2.50640541
FMPS_PE       .19489979    .04721372   4.12803325    .00003944    .07306780    .31673179

**************************************************************************
OUTCOME VARIABLE:
 FMPS_PE_M2

Model Summary
              R         R-sq          MSE            F          df1          df2            p
      .17851573    .03186787   2.62242186  35.18811892   1.00000000 1069.00000000    .00000000
```

```
Model
                 coeff         se          t          p       LLCI       ULCI
constant    2.94987195   .15537024 18.98608129   .00000000 2.54894893 3.35079497
FMPS_PE      .29207365   .04923731  5.93195743   .00000000  .16501989  .41912741

**************************************************************************
OUTCOME VARIABLE:
 FMPS_PE_M3

Model Summary
          R       R-sq        MSE           F        df1          df2          p
   .22491438  .05058648 2.06704977 56.95826150 1.00000000 1069.00000000  .00000000

Model
                 coeff         se          t          p       LLCI       ULCI
constant    1.42492704   .13794057 10.33000691   .00000000 1.06898017 1.78087391
FMPS_PE      .32991108   .04371380  7.54706973   .00000000  .21711040  .44271176

**************************************************************************
OUTCOME VARIABLE:
 FMPS_PE_M4

Model Summary
          R       R-sq        MSE           F        df1          df2          p
   .18178952  .03304743 1.68434011 36.53509366 1.00000000 1069.00000000  .00000000

Model
                 coeff         se          t          p       LLCI       ULCI
constant    1.43594580   .12451780 11.53205222   .00000000 1.11463553 1.75725607
FMPS_PE      .23851356   .03946008  6.04442666   .00000000  .13668933  .34033779

**************************************************************************
OUTCOME VARIABLE:
 FMPS_PE_M5

Model Summary
          R       R-sq        MSE           F        df1          df2          p
   .24788443  .06144669 1.85742231 69.98698108 1.00000000 1069.00000000  .00000000

Model
                 coeff         se          t          p       LLCI       ULCI
constant    1.25997347   .13075909  9.63583870   .00000000  .92255796 1.59738898
FMPS_PE      .34666263   .04143796  8.36582220   .00000000  .23973460  .45359066

**************************************************************************
OUTCOME VARIABLE:
 FMPS_PE_M6

Model Summary
          R       R-sq        MSE           F        df1          df2          p
   .16263249  .02644933 2.61583065 29.04248293 1.00000000 1069.00000000  .00000009
```

```
Model
                 coeff          se           t           p        LLCI        ULCI
constant    2.38469569   .15517486 15.36779664   .00000000  1.98427683  2.78511455
FMPS_PE      .26501152   .04917540  5.38910781   .00000009   .13811753   .39190551

**************************************************************************
OUTCOME VARIABLE:
 Positive

Model Summary
              R       R-sq        MSE          F        df1         df2           p
      .56855665  .32325666  .85740253 72.53685538 7.00000000 1063.00000000   .00000000

Model
                 coeff          se           t           p        LLCI        ULCI
constant    1.68555546   .10494481 16.06135108   .00000000  1.41474932  1.95636161
FMPS_PE     .02187274   .02919126   .74929086   .45384769  -.05345420   .09719969
FMPS_PE_M1  .15970107   .02252991  7.08840301   .00000000   .10156349   .21783865
FMPS_PE_M2  .09152060   .02367460  3.86577219   .00011745   .03042919   .15261201
FMPS_PE_M3  .03310566   .03564575   .92874084   .35323425  -.05887685   .12508818
FMPS_PE_M4  .03767777   .02801601  1.34486555   .17895552  -.03461650   .10997205
FMPS_PE_M5  .07359453   .03792513  1.94052158   .05258047  -.02426984   .17145890
FMPS_PE_M6  .12489764   .02609782  4.78574909   .00000194   .05755318   .19224209

****************** DIRECT AND INDIRECT EFFECTS OF X ON Y *****************

Direct effect of X on Y
         Effect          se           t           p        LLCI        ULCI
      .02187274   .02919126   .74929086   .45384769  -.05345420   .09719969

Indirect effect(s) of X on Y:
              Effect      BootSE    BootLLCI    BootULCI
TOTAL      .13637683   .02212768   .07974857   .19284411
FMPS_PE_M1 .03112571   .00942825   .00920780   .05840153
FMPS_PE_M2 .02673075   .00956097   .00554038   .05462248
FMPS_PE_M3 .01092193   .01325889  -.02382911   .04759972
FMPS_PE_M4 .00898666   .00823893  -.01272503   .03215770
FMPS_PE_M5 .02551247   .01468907  -.01171772   .06478711
FMPS_PE_M6 .03309931   .01002370   .01085256   .06224937

********************** ANALYSIS NOTES AND ERRORS ************************

Level of confidence for all confidence intervals in output:
  99.0000

Number of bootstrap samples for percentile bootstrap confidence intervals:
  10000

NOTE: Variables names longer than eight characters can produce incorrect output.
      Shorter variable names are recommended.
------ END MATRIX -----
```

Negative Dimension: Predictor—PANAS_TNA[193]

Note. Yellow highlighted text in the analysis output below shows significant mediated effects. "PROCESS Procedure for SPSS Version 3.4.1" in the analysis output refers to the version of the Process[172] package used to compute the analysis, whereas the version of SPSS[198] used to run the package was 23.

```
Run MATRIX procedure:

**************** PROCESS Procedure for SPSS Version 3.4.1 ****************

          Written by Andrew F. Hayes, Ph.D.       www.afhayes.com
    Documentation available in Hayes (2018). www.guilford.com/p/hayes3

**************************************************************************
Model  : 4
    Y  : Negative
    X  : PANAS_TNA
   M1  : 12-PAC_UA
   M2  : 12-PAC_AD
   M3  : 12-PAC_D
   M4  : 12-PAC_DD
   M5  : 12-PAC_UD

Sample
Size:  1071

**************************************************************************
OUTCOME VARIABLE:
 12-PAC_UA

Model Summary
              R         R-sq          MSE            F          df1          df2            p
      .59741200    .35690110    .25287286  593.26377314   1.00000000 1069.00000000    .00000000

Model
                  coeff           se            t            p         LLCI         ULCI
constant      .45674730    .04115061  11.09940567    .00000000    .35056077    .56293383
PANAS_TNA     .60290830    .02475297  24.35700665    .00000000    .53903483    .66678177

**************************************************************************

OUTCOME VARIABLE:
 12-PAC_AD

Model Summary
              R         R-sq          MSE            F          df1          df2            p
      .61311581    .37591099    .14669969  643.89669363   1.00000000 1069.00000000    .00000000
```

Model

	coeff	se	t	p	LLCI	ULCI
constant	.50832174	.03134294	16.21806062	.00000000	.42744327	.58920021
PANAS_TNA	.47840856	.01885345	25.37511958	.00000000	.42975843	.52705869

****
OUTCOME VARIABLE:
12-PAC_D

Model Summary

R	R-sq	MSE	F	df1	df2	p
.60844214	.37020183	.35246525	628.36918664	1.00000000	1069.00000000	.00000000

Model

	coeff	se	t	p	LLCI	ULCI
constant	.31912648	.04858289	6.56870142	.00000000	.19376143	.44449152
PANAS_TNA	.73255770	.02922365	25.06729317	.00000000	.65714794	.80796745

****
OUTCOME VARIABLE:
12-PAC_DD

Model Summary

R	R-sq	MSE	F	df1	df2	p
.56519092	.31944078	.46087399	501.76704445	1.00000000	1069.00000000	.00000000

Model

	coeff	se	t	p	LLCI	ULCI
constant	.33111747	.05555412	5.96026872	.00000000	.18776360	.47447135
PANAS_TNA	.74854585	.03341699	22.40015724	.00000000	.66231543	.83477627

****
OUTCOME VARIABLE:
12-PAC_UD

Model Summary

R	R-sq	MSE	F	df1	df2	p
.53782752	.28925844	.32510297	435.06288887	1.00000000	1069.00000000	.00000000

Model

	coeff	se	t	p	LLCI	ULCI
constant	.72680286	.04665902	15.57689894	.00000000	.60640223	.84720350
PANAS_TNA	.58541349	.02806640	20.85816121	.00000000	.51298993	.65783705

****
OUTCOME VARIABLE:
Negative

Model Summary

R	R-sq	MSE	F	df1	df2	p
.16192091	.02621838	.89872088	4.77457434	6.00000000	1064.00000000	.00008172

```
Model
                   coeff          se           t           p        LLCI        ULCI
constant      1.88002461   .09376665 20.05003527   .00000000  1.63806370  2.12198551
PANAS_TNA     -.01847915   .06300644  -.29328992   .76935775  -.18106464   .14410633
12-PAC_UA     -.12108288   .08659594 -1.39825117   .16232903  -.34454008   .10237432
12-PAC_AD      .48277289   .13802707  3.49766811   .00048873   .12659981   .83894597
12-PAC_D      -.04109024   .09941155  -.41333463   .67944475  -.29761758   .21543711
12-PAC_DD     -.02509244   .07985586  -.31422163   .75341431  -.23115713   .18097226
12-PAC_UD      .05681277   .06060295   .93745872   .34873539  -.09957061   .21319614

****************** DIRECT AND INDIRECT EFFECTS OF X ON Y *****************

Direct effect of X on Y
         Effect          se           t           p        LLCI        ULCI
     -.01847915   .06300644  -.29328992   .76935775  -.18106464   .14410633

Indirect effect(s) of X on Y:
                  Effect      BootSE    BootLLCI    BootULCI
TOTAL          .14233596   .05064161   .02172503   .28481341
12-PAC_UA     -.07300187   .06034289  -.23116276   .08091350
12-PAC_AD      .23096268   .08072038   .02867596   .44990980
12-PAC_D      -.03010097   .07541773  -.22768831   .17316983
12-PAC_DD     -.01878284   .06265410  -.17971851   .14651952
12-PAC_UD      .03325896   .03453402  -.05359545   .12971733

*********************** ANALYSIS NOTES AND ERRORS ************************

Level of confidence for all confidence intervals in output:
  99.0000

Number of bootstrap samples for percentile bootstrap confidence intervals:
  10000
NOTE: Variables names longer than eight characters can produce incorrect output.
      Shorter variable names are recommended.
------ END MATRIX -----
```

Negative Dimension: Predictor—IDAQ[46]

Note. Yellow highlighted text in the analysis output below shows significant mediated effects. Although the mediated effect for IDAQ_M3 was significant (ab = -0.011, 99% CI = [-0.026, -0.001], ab% = -0.196), the direction of this effect was negative and therefore opposite to the positive direction of the relationship between IDAQ and the negative domain. Therefore, IDAQ_M3 did not explain this relationship. This so-called "inconsistent mediation" (MacKinnon et al.[199], p.602) may be a statistical artefact or simply indicate that, although higher anthropomorphism directly predicted higher negative scores, it indirectly decreased them through IDAQ_M3 because this mediator was linked to positive responses to robots.
"PROCESS Procedure for SPSS Version 3.4.1" in the analysis output refers to the version of the Process[172] package used to compute the analysis, whereas the version of SPSS[198] used to run the package was 23.

```
Run MATRIX procedure:

**************** PROCESS Procedure for SPSS Version 3.4.1 ****************

          Written by Andrew F. Hayes, Ph.D.       www.afhayes.com
    Documentation available in Hayes (2018). www.guilford.com/p/hayes3

**************************************************************************
Model  : 4
    Y  : Negative
    X  : IDAQ
   M1  : IDAQ_M1
   M2  : IDAQ_M2
   M3  : IDAQ_M3
   M4  : IDAQ_M4

Sample
Size:  1071

**************************************************************************
OUTCOME VARIABLE:
 IDAQ_M1

Model Summary
              R         R-sq          MSE            F          df1          df2            p
      .16515477      .02727610   2.77811175  29.97576937   1.00000000  1069.00000000      .00000005

Model
                 coeff           se            t            p         LLCI         ULCI
constant    2.89315879    .10425720  27.75020701    .00000000   2.62412972   3.16218786
IDAQ         .18905569    .03453064   5.47501318    .00000005    .09995158    .27815980

**************************************************************************
OUTCOME VARIABLE:
 IDAQ_M2
```

```
Model Summary
          R       R-sq        MSE          F        df1        df2          p
     .15536281  .02413760  2.62780565  26.44132833  1.00000000  1069.00000000  .00000032


Model
              coeff         se          t          p       LLCI       ULCI
constant   2.47408825  .10139763  24.39986245  .00000000  2.21243811  2.73573839
IDAQ        .17269032  .03358353   5.14211322  .00000032   .08603015   .25935048

**************************************************************************
OUTCOME VARIABLE:
 IDAQ_M3

Model Summary
          R       R-sq        MSE          F        df1        df2          p
     .14611527  .02134967  2.04551005  23.32068794  1.00000000  1069.00000000  .00000157

Model
              coeff         se          t          p       LLCI       ULCI
constant   3.58382287  .08946064  40.06033165  .00000000  3.35297538  3.81467036
IDAQ        .14308734  .02962992   4.82914982  .00000157   .06662921   .21954548

**************************************************************************
OUTCOME VARIABLE:
 IDAQ_M4

Model Summary
          R       R-sq        MSE          F        df1        df2          p
     .02478106  .00061410  2.30220689   .65687740  1.00000000  1069.00000000  .41784490

Model
              coeff         se          t          p       LLCI       ULCI
constant   3.11473717  .09490812  32.81844830  .00000000  2.86983280  3.35964153
IDAQ       -.02547675  .03143416   -.81047973  .41784490  -.10659061   .05563711

**************************************************************************
OUTCOME VARIABLE:
 Negative

Model Summary
          R       R-sq        MSE          F        df1        df2          p
     .35366340  .12507780   .80672351  30.45021774  5.00000000  1065.00000000  .00000000

Model
              coeff         se          t          p       LLCI       ULCI
constant   1.72707111  .13286598  12.99859518  .00000000  1.38421659  2.06992563
IDAQ        .06285464  .01897527   3.31245005  .00095591   .01388983   .11181945
IDAQ_M1     .00457131  .02066607    .22119854  .82498025  -.04875654   .05789916
```

```
IDAQ_M2       .04894791      .02037308     2.40257785      .01645029     -.00362388      .10151971
IDAQ_M3      -.07992610      .02467793    -3.23876814      .00123757     -.14360636     -.01624584
IDAQ_M4       .17917054      .02036435     8.79824701      .00000000      .12662129      .23171979

****************** DIRECT AND INDIRECT EFFECTS OF X ON Y *****************

Direct effect of X on Y
         Effect             se              t              p           LLCI           ULCI
      .06285464      .01897527     3.31245005      .00095591      .01388983      .11181945

Indirect effect(s) of X on Y:
                 Effect         BootSE       BootLLCI       BootULCI
TOTAL        -.00668403      .00838132     -.02852472      .01524616
IDAQ_M1       .00086423      .00450691     -.01091545      .01377881
IDAQ_M2       .00845283      .00439349     -.00190190      .02153304
IDAQ_M3      -.01143641      .00461475     -.02567079     -.00141210
IDAQ_M4      -.00456468      .00619951     -.02113868      .01137460

********************** ANALYSIS NOTES AND ERRORS ************************

Level of confidence for all confidence intervals in output:
  99.0000

Number of bootstrap samples for percentile bootstrap confidence intervals:
  10000

NOTE: Variables names longer than eight characters can produce incorrect output.
      Shorter variable names are recommended.

------ END MATRIX -----
```

Negative Dimension: Predictor— SD3_P[194]

Note. Yellow highlighted text in the analysis output below shows significant mediated effects. "PROCESS Procedure for SPSS Version 3.4.1" in the analysis output refers to the version of the Process[172] package used to compute the analysis, whereas the version of SPSS[198] used to run the package was 23.

```
Run MATRIX procedure:

**************** PROCESS Procedure for SPSS Version 3.4.1 ****************

          Written by Andrew F. Hayes, Ph.D.       www.afhayes.com
    Documentation available in Hayes (2018). www.guilford.com/p/hayes3

**************************************************************************
Model  : 4
    Y  : Negative
    X  : SD3_P
   M1  : 12-PAC_UA
   M2  : 12-PAC_AD
   M3  : 12-PAC_D
   M4  : 12-PAC_DD
   M5  : 12-PAC_UD
   M6  : SD3_P_M1
   M7  : SD3_P_M2
   M8  : SD3_P_M3
   M9  : SD3_P_M4

Sample
Size:  1071

**************************************************************************
OUTCOME VARIABLE:
 12-PAC_UA

Model Summary
              R          R-sq           MSE             F           df1           df2             p
      .15108448      .02282652      .38423429    24.97156398     1.00000000  1069.00000000      .00000068

Model
                  coeff            se             t             p          LLCI          ULCI
constant     1.07991098      .06422039    16.81570356      .00000000      .91419436     1.24562761
SD3_P         .15814543      .03164709     4.99715559      .00000068      .07648213      .23980873

**************************************************************************
OUTCOME VARIABLE:
 12-PAC_AD

Model Summary
```

```
              R          R-sq          MSE             F          df1          df2            p
      .17661157     .03119165     .22773016    34.41740526    1.00000000  1069.00000000     .00000001

Model
                 coeff          se            t            p         LLCI         ULCI
constant      .96897672     .04944076   19.59874290     .00000000     .84139799    1.09655546
SD3_P         .14293383     .02436385    5.86663492     .00000001     .08006446     .20580321

**************************************************************************
OUTCOME VARIABLE:
 12-PAC_D

Model Summary
              R          R-sq          MSE             F          df1          df2            p
      .20668919     .04272042     .53573955    47.70615811    1.00000000  1069.00000000     .00000000

Model
                 coeff          se            t            p         LLCI         ULCI
constant      .94841064     .07583186   12.50675686     .00000000     .75273135    1.14408993
SD3_P         .25810700     .03736909    6.90696446     .00000000     .16167843     .35453557

**************************************************************************
OUTCOME VARIABLE:
 12-PAC_DD

Model Summary
              R          R-sq          MSE             F          df1          df2            p
      .18585048     .03454040     .65380823    38.24467629    1.00000000  1069.00000000     .00000000

Model
                 coeff          se            t            p         LLCI         ULCI
constant      .99050644     .08377222   11.82380499     .00000000     .77433754    1.20667534
SD3_P         .25529740     .04128202    6.18422803     .00000000     .14877178     .36182302

**************************************************************************
OUTCOME VARIABLE:
 12-PAC_UD

Model Summary
              R          R-sq          MSE             F          df1          df2            p
      .19104868     .03649960     .44071834    40.49616516    1.00000000  1069.00000000     .00000000

Model
                 coeff          se            t            p         LLCI         ULCI
constant     1.21141373     .06877893   17.61315161     .00000000    1.03393407    1.38889339
SD3_P         .21568663     .03389349    6.36365973     .00000000     .12822664     .30314662

**************************************************************************
OUTCOME VARIABLE:
 SD3_P_M1
```

```
Model Summary
          R       R-sq        MSE          F        df1        df2          p
      .39472854  .15581062  1.96237297  197.30354399  1.00000000  1069.00000000  .00000000

Model
              coeff         se          t          p       LLCI       ULCI
constant   .92229416   .14513282   6.35482837   .00000000   .54778815  1.29680017
SD3_P     1.00460174   .07151983  14.04647799   .00000000   .82004937  1.18915412

**************************************************************************
OUTCOME VARIABLE:
 SD3_P_M2

Model Summary
          R       R-sq        MSE          F        df1        df2          p
      .31710879  .10055799   .93299141  119.51464027  1.00000000  1069.00000000  .00000000

Model
              coeff         se          t          p       LLCI       ULCI
constant  1.03027861   .10007225  10.29534738   .00000000   .77204852  1.28850870
SD3_P      .53911955   .04931449  10.93227516   .00000000   .41186665   .66637246

**************************************************************************
OUTCOME VARIABLE:
 SD3_P_M3

Model Summary
          R       R-sq        MSE          F        df1        df2          p
      .11470624  .01315752  2.63960295  14.25292405  1.00000000  1069.00000000  .00016861

Model
              coeff         se          t          p       LLCI       ULCI
constant  2.76908028   .16832327  16.45096537   .00000000  2.33473278  3.20342777
SD3_P      .31315330   .08294783   3.77530450   .00016861   .09911171   .52719489

**************************************************************************
OUTCOME VARIABLE:
 SD3_P_M4

Model Summary
          R       R-sq        MSE          F        df1        df2          p
      .21234953  .04509232  2.21203819  50.47995054  1.00000000  1069.00000000  .00000000

Model
              coeff         se          t          p       LLCI       ULCI
constant  2.39742134   .15408883  15.55869622   .00000000  1.99980491  2.79503778
SD3_P      .53950004   .07593326   7.10492439   .00000000   .34355910   .73544097

**************************************************************************
OUTCOME VARIABLE:
 Negative
```

Model Summary

R	R-sq	MSE	F	df1	df2	p
.34954751	.12218346	.81321013	14.75416149	10.00000000	1060.00000000	.00000000

Model

	coeff	se	t	p	LLCI	ULCI
constant	1.14500088	.12497945	9.16151306	.00000000	.82249445	1.46750730
SD3_P	.16004971	.05176959	3.09157762	.00204296	.02645954	.29363988
12-PAC_UA	-.10782382	.08142624	-1.32418999	.18572541	-.31794226	.10229462
12-PAC_AD	.44641951	.13195836	3.38303306	.00074311	.10590417	.78693484
12-PAC_D	-.10724711	.09519319	-1.12662590	.26015567	-.35289081	.13839659
12-PAC_DD	-.01896504	.07603483	-.24942565	.80307987	-.21517107	.17724099
12-PAC_UD	-.01409205	.05742559	-.24539672	.80619677	-.16227738	.13409327
SD3_P_M1	-.01118589	.02167661	-.51603482	.60593771	-.06712186	.04475009
SD3_P_M2	.22560780	.03032543	7.43955907	.00000000	.14735378	.30386182
SD3_P_M3	.07152245	.01818155	3.93379375	.00008906	.02460542	.11843949
SD3_P_M4	-.01517276	.01910989	-.79397391	.42738833	-.06448536	.03413985

****************** DIRECT AND INDIRECT EFFECTS OF X ON Y *****************

Direct effect of X on Y

Effect	se	t	p	LLCI	ULCI
.16004971	.05176959	3.09157762	.00204296	.02645954	.29363988

Indirect effect(s) of X on Y:

	Effect	BootSE	BootLLCI	BootULCI
TOTAL	.13579819	.03072140	.06049766	.22066899
12-PAC_UA	-.01705184	.01648580	-.06674429	.02246363
12-PAC_AD	.06380845	.02496629	.01009341	.13601906
12-PAC_D	-.02768123	.02564615	-.09753965	.03730109
12-PAC_DD	-.00484172	.02102095	-.06124391	.05105656
12-PAC_UD	-.00303947	.01217732	-.03689754	.02909597
SD3_P_M1	-.01123736	.02417816	-.07464725	.05025854
SD3_P_M2	.12162958	.02221857	.06961185	.18348038
SD3_P_M3	.02239749	.00876196	.00463457	.04968999
SD3_P_M4	-.00818570	.01054741	-.03598672	.01977285

********************** ANALYSIS NOTES AND ERRORS ************************

Level of confidence for all confidence intervals in output:
  99.0000

Number of bootstrap samples for percentile bootstrap confidence intervals:
  10000

NOTE: Variables names longer than eight characters can produce incorrect output.
      Shorter variable names are recommended.

------ END MATRIX -----

Negative Dimension: Predictor—ERQ_ES[195]

Note. "PROCESS Procedure for SPSS Version 3.4.1" in the analysis output below refers to the version of the Process[172] package used to compute the analysis, whereas the version of SPSS[198] used to run the package was 23.

```
Run MATRIX procedure:

**************** PROCESS Procedure for SPSS Version 3.4.1 ****************

          Written by Andrew F. Hayes, Ph.D.       www.afhayes.com
    Documentation available in Hayes (2018). www.guilford.com/p/hayes3

**************************************************************************
Model  : 4
    Y  : Negative
    X  : ERQ_ES
   M1  : 12-PAC_UA
   M2  : 12-PAC_AD
   M3  : 12-PAC_D
   M4  : 12-PAC_DD
   M5  : 12-PAC_UD
   M6  : ERQ_ES_M1
   M7  : ERQ_ES_M2

Sample
Size:  1071

**************************************************************************
OUTCOME VARIABLE:
 12-PAC_UA

Model Summary
              R         R-sq          MSE            F          df1          df2            p
      .09035760      .00816450      .38999954     8.79969159     1.00000000  1069.00000000      .00307981

Model
                 coeff           se            t            p         LLCI         ULCI
constant     1.23563768      .05433608    22.74064987      .00000000     1.09542689     1.37584847
ERQ_ES        .04000546      .01348607     2.96642741      .00307981      .00520550      .07480541

**************************************************************************
OUTCOME VARIABLE:
 12-PAC_AD

Model Summary
              R         R-sq          MSE            F          df1          df2            p
      .08465751      .00716689      .23337747     7.71671380     1.00000000  1069.00000000      .00556693

Model
```

	coeff	se	t	p	LLCI	ULCI
constant	1.13680052	.04203254	27.04572205	.00000000	1.02833821	1.24526283
ERQ_ES	.02898005	.01043237	2.77789737	.00556693	.00205999	.05590011

****
OUTCOME VARIABLE:
12-PAC_D

Model Summary

R	R-sq	MSE	F	df1	df2	p
.16491470	.02719686	.54442728	29.88625201	1.00000000	1069.00000000	.00000006

Model

	coeff	se	t	p	LLCI	ULCI
constant	1.12027173	.06419869	17.45007165	.00000000	.95461109	1.28593237
ERQ_ES	.08710822	.01593395	5.46683199	.00000006	.04599168	.12822476

****
OUTCOME VARIABLE:
12-PAC_DD

Model Summary

R	R-sq	MSE	F	df1	df2	p
.13784674	.01900172	.66433100	20.70629688	1.00000000	1069.00000000	.00000597

Model

	coeff	se	t	p	LLCI	ULCI
constant	1.18338234	.07091669	16.68693603	.00000000	1.00038632	1.36637836
ERQ_ES	.08009344	.01760134	4.55041722	.00000597	.03467430	.12551258

****
OUTCOME VARIABLE:
12-PAC_UD

Model Summary

R	R-sq	MSE	F	df1	df2	p
.12412123	.01540608	.45036681	16.72679240	1.00000000	1069.00000000	.00004641

Model

	coeff	se	t	p	LLCI	ULCI
constant	1.40603480	.05839013	24.08000993	.00000000	1.25536280	1.55670681
ERQ_ES	.05927110	.01449228	4.08984014	.00004641	.02187470	.09666750

****
OUTCOME VARIABLE:
ERQ_ES_M1

Model Summary

R	R-sq	MSE	F	df1	df2	p
.15704229	.02466228	3.26679385	27.03061338	1.00000000	1069.00000000	.00000024

```
Model
                 coeff         se          t          p       LLCI       ULCI
constant    2.19712379   .15725957  13.97131999   .00000000  1.79132547  2.60292211
ERQ_ES       .20292814   .03903142   5.19909736   .00000024   .10221005   .30364623

**************************************************************************
OUTCOME VARIABLE:
 ERQ_ES_M2

Model Summary
          R       R-sq        MSE          F        df1        df2          p
   .16293409   .02654752  3.16064627  29.15324324  1.00000000  1069.00000000  .00000008

Model
                 coeff         se          t          p       LLCI       ULCI
constant    2.10315971   .15468356  13.59653000   .00000000  1.70400861  2.50231081
ERQ_ES       .20729310   .03839206   5.39937434   .00000008   .10822483   .30636137

**************************************************************************
OUTCOME VARIABLE:
 Negative

Model Summary
          R       R-sq        MSE          F        df1        df2          p
   .20469555   .04190027   .88591303   5.80551304  8.00000000  1062.00000000  .00000029

Model
                 coeff         se          t          p       LLCI       ULCI
constant    1.66622741   .11377587  14.64482211   .00000000  1.37263255  1.95982228
ERQ_ES       .05421591   .02078893   2.60792275   .00923717   .00057078   .10786104
12-PAC_UA   -.18081666   .08612385  -2.09949583   .03600874  -.40305639   .04142307
12-PAC_AD    .57405713   .13869654   4.13894334   .00003765   .21615530   .93195896
12-PAC_D    -.08937041   .09891065   -.90354686   .36644063  -.34460607   .16586525
12-PAC_DD   -.06486868   .08064351   -.80438811   .42135289  -.27296659   .14322922
12-PAC_UD   -.01684078   .06356410   -.26494168   .79110580  -.18086582   .14718426
ERQ_ES_M1    .03651087   .03416354   1.06870841   .28544393  -.05164702   .12466876
ERQ_ES_M2    .03145289   .03491387    .90087101   .36786115  -.05864119   .12154697

****************** DIRECT AND INDIRECT EFFECTS OF X ON Y *****************

Direct effect of X on Y
     Effect         se          t          p       LLCI       ULCI
   .05421591   .02078893  2.60792275   .00923717   .00057078   .10786104

Indirect effect(s) of X on Y:
               Effect     BootSE   BootLLCI   BootULCI
TOTAL        .00935298   .00645436  -.00709196   .02616254
12-PAC_UA   -.00723365   .00491374  -.02346680   .00279294
12-PAC_AD    .01663620   .00850497  -.00215038   .04295595
12-PAC_D    -.00778490   .00959443  -.03674917   .01380745
12-PAC_DD   -.00519556   .00723553  -.02614445   .01340841
```

```
12-PAC_UD    -.00099817      .00381388     -.01284658      .00946659
ERQ_ES_M1     .00740908      .00712607     -.00995539      .02896013
ERQ_ES_M2     .00651997      .00700624     -.01154980      .02694814

********************** ANALYSIS NOTES AND ERRORS ************************

Level of confidence for all confidence intervals in output:
  99.0000

Number of bootstrap samples for percentile bootstrap confidence intervals:
  10000

NOTE: Variables names longer than eight characters can produce incorrect output.
      Shorter variable names are recommended.

------ END MATRIX -----
```

Competence Dimension: Predictor—ATQ_AP[97]

Note. Yellow highlighted text in the analysis output below shows significant mediated effects. "PROCESS Procedure for SPSS Version 3.4.1" in the analysis output refers to the version of the Process[172] package used to compute the analysis, whereas the version of SPSS[198] used to run the package was 23.

```
Run MATRIX procedure:

**************** PROCESS Procedure for SPSS Version 3.4.1 ****************

          Written by Andrew F. Hayes, Ph.D.       www.afhayes.com
    Documentation available in Hayes (2018). www.guilford.com/p/hayes3

**************************************************************************
Model  : 4
    Y  : Competen
    X  : ATQ_AP
   M1  : ATQ_AP_M1
   M2  : ATQ_AP_M2
   M3  : ATQ_AP_M3
   M4  : ATQ_AP_M4
   M5  : ATQ_AP_M5
   M6  : ATQ_AP_M6
   M7  : ATQ_AP_M7
   M8  : ATQ_AP_M8
   M9  : ATQ_AP_M9
   M10 : ATQ_AP_M10

Sample
Size:  1071

**************************************************************************
OUTCOME VARIABLE:
 ATQ_AP_M1

Model Summary
            R          R-sq           MSE             F           df1           df2             p
      .12379354      .01532484     2.35685633    16.63721629     1.00000000  1069.00000000      .00004861

Model
                  coeff            se             t             p          LLCI          ULCI
constant     3.40711352      .24983400    13.63750912      .00000000     2.76243276     4.05179428
ATQ_AP        .19776431      .04848502     4.07887439      .00004861      .07265179      .32287682

**************************************************************************
OUTCOME VARIABLE:
 ATQ_AP_M2
```

```
Model Summary
          R       R-sq        MSE          F        df1        df2          p
     .16297173  .02655978  1.95183054  29.16707954  1.00000000 1069.00000000  .00000008

Model
              coeff         se          t          p       LLCI       ULCI
constant   3.90227911  .22735581  17.16375391  .00000000  3.31560191  4.48895631
ATQ_AP      .23829150  .04412270   5.40065547  .00000008   .12443568   .35214732

**************************************************************************
OUTCOME VARIABLE:
 ATQ_AP_M3

Model Summary
          R       R-sq        MSE          F        df1        df2          p
     .18054956  .03259814  2.13327801  36.02165474  1.00000000 1069.00000000  .00000000

Model
              coeff         se          t          p       LLCI       ULCI
constant   3.07873714  .23768880  12.95280680  .00000000  2.46539630  3.69207799
ATQ_AP      .27685131  .04612801   6.00180429  .00000000   .15782090   .39588171

**************************************************************************
OUTCOME VARIABLE:
 ATQ_AP_M4

Model Summary
          R       R-sq        MSE          F        df1        df2          p
     .13399771  .01795539  1.49403006  19.54524935  1.00000000 1069.00000000  .00001083

Model
              coeff         se          t          p       LLCI       ULCI
constant   4.73008134  .19891369  23.77956714  .00000000  4.21679722  5.24336545
ATQ_AP      .17066376  .03860297   4.42100094  .00001083   .07105125   .27027626

**************************************************************************
OUTCOME VARIABLE:
 ATQ_AP_M5

Model Summary
          R       R-sq        MSE          F        df1        df2          p
     .21948170  .04817222  1.10104681  54.10232736  1.00000000 1069.00000000  .00000000

Model
              coeff         se          t          p       LLCI       ULCI
constant   4.45259666  .17076068  26.07506918  .00000000  4.01195957  4.89323375
ATQ_AP      .24375408  .03313934   7.35542843  .00000000   .15824011   .32926805

**************************************************************************
OUTCOME VARIABLE:
 ATQ_AP_M6
```

Model Summary

R	R-sq	MSE	F	df1	df2	p
.25995715	.06757772	1.78304477	77.47625024	1.00000000	1069.00000000	.00000000

Model

	coeff	se	t	p	LLCI	ULCI
constant	3.01206112	.21730320	13.86109882	.00000000	2.45132403	3.57279820
ATQ_AP	.37119870	.04217180	8.80205943	.00000000	.26237705	.48002035

****

OUTCOME VARIABLE:

ATQ_AP_M7

Model Summary

R	R-sq	MSE	F	df1	df2	p
.21547475	.04642937	2.01700230	52.04962355	1.00000000	1069.00000000	.00000000

Model

	coeff	se	t	p	LLCI	ULCI
constant	2.42945696	.23112035	10.51165303	.00000000	1.83306558	3.02584833
ATQ_AP	.32359590	.04485328	7.21454250	.00000000	.20785486	.43933694

****

OUTCOME VARIABLE:

ATQ_AP_M8

Model Summary

R	R-sq	MSE	F	df1	df2	p
.19223867	.03695571	2.08644246	41.02163305	1.00000000	1069.00000000	.00000000

Model

	coeff	se	t	p	LLCI	ULCI
constant	3.30087537	.23506513	14.04238659	.00000000	2.69430476	3.90744598
ATQ_AP	.29218015	.04561884	6.40481327	.00000000	.17446363	.40989666

****

OUTCOME VARIABLE:

ATQ_AP_M9

Model Summary

R	R-sq	MSE	F	df1	df2	p
.24959701	.06229867	1.91025383	71.02184059	1.00000000	1069.00000000	.00000000

Model

	coeff	se	t	p	LLCI	ULCI
constant	2.30907102	.22492127	10.26612989	.00000000	1.72867597	2.88946606
ATQ_AP	.36785997	.04365023	8.42744567	.00000000	.25522331	.48049662

****

OUTCOME VARIABLE:

ATQ_AP_M10

Model Summary

R	R-sq	MSE	F	df1	df2	p
.17072451	.02914686	1.96897398	32.09341360	1.00000000	1069.00000000	.00000002

Model

	coeff	se	t	p	LLCI	ULCI
constant	3.79753136	.22835209	16.63015837	.00000000	3.20828332	4.38677939
ATQ_AP	.25105505	.04431605	5.66510491	.00000002	.13670031	.36540979

****

OUTCOME VARIABLE:

Competen

Model Summary

R	R-sq	MSE	F	df1	df2	p
.40996133	.16806829	.74603990	19.44918426	11.00000000	1059.00000000	.00000000

Model

	coeff	se	t	p	LLCI	ULCI
constant	3.03994978	.19442131	15.63588788	.00000000	2.53824948	3.54165008
ATQ_AP	.07012019	.02901182	2.41695256	.01581922	-.00474423	.14498461
ATQ_AP_M1	.04209756	.02252337	1.86906161	.06189010	-.01602354	.10021867
ATQ_AP_M2	.06274950	.02731411	2.29732906	.02179434	-.00773402	.13323302
ATQ_AP_M3	-.03650471	.01989669	-1.83471308	.06682860	-.08784772	.01483829
ATQ_AP_M4	.06803384	.02614943	2.60173338	.00940481	.00055576	.13551193
ATQ_AP_M5	.08041815	.02882532	2.78984406	.00536796	.00603499	.15480131
ATQ_AP_M6	.00675757	.02327278	.29036353	.77159499	-.05329738	.06681251
ATQ_AP_M7	-.01748190	.02700069	-.64746129	.51747377	-.08715665	.05219284
ATQ_AP_M8	.03593808	.02924904	1.22869261	.21946005	-.03953849	.11141466
ATQ_AP_M9	.04742823	.02707928	1.75145843	.08015645	-.02244930	.11730576
ATQ_AP_M10	.07052701	.02874176	2.45381675	.01429509	-.00364052	.14469454

****************** DIRECT AND INDIRECT EFFECTS OF X ON Y *****************

Direct effect of X on Y

Effect	se	t	p	LLCI	ULCI
.07012019	.02901182	2.41695256	.01581922	-.00474423	.14498461

Indirect effect(s) of X on Y:

	Effect	BootSE	BootLLCI	BootULCI
TOTAL	.08688968	.01740951	.04423602	.13458620
ATQ_AP_M1	.00832540	.00551019	-.00405888	.02561141
ATQ_AP_M2	.01495267	.00910301	-.00671639	.04178807
ATQ_AP_M3	-.01010638	.00629142	-.02893679	.00510500
ATQ_AP_M4	.01161091	.00594998	-.00124722	.02998890
ATQ_AP_M5	.01960225	.00777975	.00150024	.04234337
ATQ_AP_M6	.00250840	.00926320	-.02096943	.02747026
ATQ_AP_M7	-.00565707	.00932303	-.03187199	.01967900
ATQ_AP_M8	.01050039	.00893237	-.01150208	.03681757

```
ATQ_AP_M9    .01744695      .01077462     -.01009407      .04651602
ATQ_AP_M10   .01770616      .00853025     -.00161006      .04280441

********************** ANALYSIS NOTES AND ERRORS ************************

Level of confidence for all confidence intervals in output:
  99.0000

Number of bootstrap samples for percentile bootstrap confidence intervals:
  10000

NOTE: Variables names longer than eight characters can produce incorrect output.
      Shorter variable names are recommended.

------ END MATRIX -----
```

Competence Dimension: Predictor—PVQ5X[196]

Note. Yellow highlighted text in the analysis output below shows significant mediated effects. "PROCESS Procedure for SPSS Version 3.4.1" in the analysis output refers to the version of the Process[172] package used to compute the analysis, whereas the version of SPSS[198] used to run the package was 23.

```
Run MATRIX procedure:

**************** PROCESS Procedure for SPSS Version 3.4.1 ****************

          Written by Andrew F. Hayes, Ph.D.       www.afhayes.com
    Documentation available in Hayes (2018). www.guilford.com/p/hayes3

**************************************************************************
Model  : 4
    Y  : Competen
    X  : PVQ5X_SS
   M1  : PVQ5X_SS_M1
   M2  : PVQ5X_SS_M2
   M3  : PVQ5X_SS_M3
   M4  : PVQ5X_SS_M4
   M5  : PVQ5X_SS_M5

Sample
Size:  1071

**************************************************************************
OUTCOME VARIABLE:
 PVQ5X_SS_M1

Model Summary
              R         R-sq          MSE            F          df1          df2            p
      .13905664     .01933675   1.74610974  21.07857660   1.00000000 1069.00000000    .00000493

Model
                  coeff           se            t            p         LLCI         ULCI
constant     4.54383340    .14984570  30.32341553    .00000000   4.15716610   4.93050070
PVQ5X_SS      .15835912    .03449232   4.59114110    .00000493    .06935387    .24736436

**************************************************************************
OUTCOME VARIABLE:
 PVQ5X_SS_M2

Model Summary
              R         R-sq          MSE            F          df1          df2            p
      .27388059     .07501058   1.70954256  86.68888864   1.00000000 1069.00000000    .00000000

Model
```

```
                 coeff          se          t          p       LLCI       ULCI
constant    3.53986318   .14826836 23.87470437  .00000000 3.15726611 3.92246025
PVQ5X_SS     .31776667   .03412924  9.31068680  .00000000  .22969834  .40583501

**************************************************************************
OUTCOME VARIABLE:
 PVQ5X_SS_M3

Model Summary
              R       R-sq        MSE          F        df1           df2          p
      .05165490  .00266823 2.13745747 2.85996726 1.00000000 1069.00000000  .09110083

Model
                 coeff          se          t          p       LLCI       ULCI
constant    4.13989397   .16578959 24.97077165  .00000000 3.71208448 4.56770346
PVQ5X_SS     .06453806   .03816238  1.69114377  .09110083 -.03393752  .16301365

**************************************************************************
OUTCOME VARIABLE:
 PVQ5X_SS_M4

Model Summary
              R       R-sq        MSE           F        df1           df2          p
      .16040304  .02572914 1.97652321 28.23080129 1.00000000 1069.00000000  .00000013

Model
                 coeff          se          t          p       LLCI       ULCI
constant    4.06474433   .15942612 25.49610035  .00000000 3.65335537 4.47613329
PVQ5X_SS     .19498411   .03669760  5.31326654  .00000013  .10028830  .28967993

**************************************************************************
OUTCOME VARIABLE:
 PVQ5X_SS_M5

Model Summary
              R       R-sq        MSE          F        df1           df2          p
      .08474444  .00718162 2.12989689 7.73268587 1.00000000 1069.00000000  .00551825

Model
                 coeff          se          t          p       LLCI       ULCI
constant    3.79677841   .16549612 22.94179778  .00000000 3.36972621 4.22383062
PVQ5X_SS     .10593297   .03809482  2.78077073  .00551825  .00763170  .20423424

**************************************************************************
OUTCOME VARIABLE:
 Competen

Model Summary
              R       R-sq        MSE           F        df1           df2          p
      .38459640  .14791439  .76052228 30.78347057 6.00000000 1064.00000000  .00000000
```

```
Model
                  coeff           se            t            p         LLCI         ULCI
constant     3.59212418     .14112864   25.45283693     .00000000   3.22794764   3.95630071
PVQ5X_SS      .04559828     .02380644    1.91537600     .05571240   -.01583324    .10702980
PVQ5X_SS_M1   .05836698     .02893715    2.01702559     .04394362   -.01630413    .13303809
PVQ5X_SS_M2   .04904453     .02722207    1.80164553     .07188418   -.02120089    .11928995
PVQ5X_SS_M3   .06235976     .02344139    2.66024106     .00792591    .00187022    .12284930
PVQ5X_SS_M4   .07862275     .02516987    3.12368492     .00183430    .01367295    .14357256
PVQ5X_SS_M5   .06602434     .02347129    2.81298326     .00499874    .00545766    .12659101

****************** DIRECT AND INDIRECT EFFECTS OF X ON Y *****************

Direct effect of X on Y
         Effect           se            t            p         LLCI         ULCI
      .04559828    .02380644   1.91537600    .05571240   -.01583324    .10702980

Indirect effect(s) of X on Y:
               Effect       BootSE     BootLLCI     BootULCI
TOTAL       .05117658    .01247999    .02091959    .08550622
PVQ5X_SS_M1 .00924294    .00579254   -.00467447    .02700283
PVQ5X_SS_M2 .01558472    .00956343   -.00786205    .04166870
PVQ5X_SS_M3 .00402458    .00322391   -.00289861    .01471461
PVQ5X_SS_M4 .01533019    .00675087    .00045237    .03570380
PVQ5X_SS_M5 .00699415    .00395466   -.00062979    .01945527

*********************** ANALYSIS NOTES AND ERRORS ************************

Level of confidence for all confidence intervals in output:
  99.0000

Number of bootstrap samples for percentile bootstrap confidence intervals:
  10000

NOTE: Variables names longer than eight characters can produce incorrect output.
      Shorter variable names are recommended.

------ END MATRIX -----
```

Supplementary Discussion

In this research, we adopted a meticulous data driven approach combining various qualitative (e.g., iterative categorization[112]) and quantitative analyses (e.g., factor-analytic techniques[153,200], machine learning[163,164]) to undertake a comprehensive investigation of psychological processes regarding an extensive sample of stimuli depicting robots across 28 domains of human activity in which they operate (e.g., healthcare, education, industry; Supplementary Table 7). The starting point of our investigation was measuring how people characterize robots (Supplementary Table 3) to develop an all-encompassing definition (Supplementary Table 4) in Study 1. This definition was then used in Study 2 to identify all domains of human activity where robots can be found (Supplementary Table 5). The definition and domains allowed us to elicit a comprehensive content space of affective, cognitive, and behavioral (i.e., psychological) processes in Study 3 (Supplementary Table 6), and to ultimately organize these processes into a taxonomy in Studies 4 and 5 (Supplementary Tables 6 and 8). We labelled this taxonomy the Positive-Negative-Competence (PNC) model because the EFA[140] (Supplementary Table 6) and ESEM[153] analyses (Supplementary Table 8) showed that the psychological processes fall under these three dimensions. To gain a deeper understanding of the PNC model, in Study 6 (Supplementary Table 12) we identified the main individual difference predictors of its positive (i.e., general risk propensity[191], anthropomorphism[46], and parental expectations[192]), negative (i.e., trait negative affect[193], anthropomorphism[46], psychopathy[194], and expressive suppression[195]), and competence (i.e., approach temperament[97], security-societal[196]) dimensions. Finally, in Study 7, we probed the key mediators behind these predictors (Supplementary Table 17).

Overall, the main contribution of the present research is laying the foundations to investigate psychological processes in response to robots from the perspective of psychological and behavioral science. This was achieved in three ways. First, by developing the PNC model, we transformed these psychological processes into a valid construct. Second, by creating the PRR scale (Supplementary Table 8), we established a tool that can be used to measure them and therefore investigate what shapes them and how they are linked to various psychological constructs. Third, by comprehensively investigating the individual difference predictors of the positive, negative, and competence dimensions, we clarified how our model is linked to individual difference constructs that psychologists typically study and how it can be understood from the perspective of these constructs. In the next section, we undertake a comprehensive inductive integration[201] to more stringently assess how the present research has moved the field forward by examining it in relation to previous relevant work.

Inductive Integration

Phase One: Robot Definition and Domains

The robot definition we developed (Supplementary Table 4) shares several elements with the widely used IEEE definition[8]. They both conceptualize robots as devices or entities that can perform different tasks (Part 1, Supplementary Table 4), and they emphasize that robots can have different degrees of autonomy (Part 2, Supplementary Table 4). Moreover, they refer to robots' composition (Part 5, Supplementary Table 4). Our definition contains two elements that are not mentioned by the IEEE—durability (Part 3, Supplementary Table 4) and positive/negative attributes (Part 4, Supplementary Table 4)—whereas the IEEE definition has one element we did not cover—robots' capability to form robotic systems. Another common definition, proposed by the International Organization for Standardization (ISO[202]), is relatively brief and contains two elements that are also part of our definition: robots as devices that can perform tasks (Part 1, Supplementary Table 4) and have different degrees of autonomy (Part 2, Supplementary Table 4). Overall, although our definition is somewhat more nuanced, it is remarkably aligned with the two official definitions, which indicates that experts and lay individuals

perceive robots similarly. Importantly, the two elements that all three definitions share refer to robots as devices or entities that perform different tasks (Part 1, Supplementary Table 4) and can vary in their degree of autonomy (Part 2, Supplementary Table 4). It is therefore possible to speculate that these two characteristics—performing tasks and a degree of autonomy—might be fundamental characteristics of robots because they have emerged in both expert and non-expert settings.

When it comes to robot types, we have identified 28 domains in which robots operate (Supplementary Table 5), which is more than professional organisations usually propose (e.g., the IEEE lists 18 domains on their website, https://robots.ieee.org/learn/types-of-robots/). However, this is not surprising because our list was intentionally nuanced to enable the identification of a comprehensive sample of robots, and we hope that other scholars will adopt it in their research for this purpose. It is important to emphasize that, despite the meticulous procedure used to develop the list, it is possible that (a) we failed to identify more niche domains and (b) the number of domains might increase as technology advances.

Phase Two: The PNC Model of Psychological Responses to Robots

We first compare the psychological processes we uncovered (Supplementary Table 6) against the ones reported in previous research and then examine the PNC model more specifically. In Study 3, participants generally evoked all the processes we could identify in the literature (for an overview, see the “Supplementary Notes” section). For example, they referred to anthropomorphism (Items 24, 27, and 37, Supplementary Table 6); negative feelings such as anxiety, fear, and creepiness (Items 13, 102, and 103, Supplementary Table 6); various positive feelings (Items 32, 77, 80, 82, and 84, Supplementary Table 6); empathy (Item 116, Supplementary Table 6); emotional attachment to robots (Item 35, Supplementary Table 6); competence (Items 2, 4, and 7, Supplementary Table 6); robots being beneficial to society (Item 109, Supplementary Table 6); robots taking over human jobs (Item 51, Supplementary Table 6); robots being helpful (Item 6, Supplementary Table 6); trust (Item 121, Supplementary Table 6); privacy (Item 126, Supplementary Table 6); and various approach (Items 22 and 40, Supplementary Table 6) or avoidance (Items 11 and 52, Supplementary Table 6) behaviors. However, our participants also went beyond and described numerous psychological processes that are rarely or never encountered in the literature. For example, they indicated that robots contribute to human degeneration (Item 142, Supplementary Table 6); lead to existential questioning (Item 148, Supplementary Table 6); can damage reputation (Item 48, Supplementary Table 6); lead to disconnection among humans (Item 140, Supplementary Table 6); restrict freedom (Item 114, Supplementary Table 6); make people feel dehumanized and insignificant (Items 25 and 117, Supplementary Table 6); and can create dependence (Item 139, Supplementary Table 6).

One of the most important contributions of the present research is showing that these seemingly highly diverse psychological processes fall under three dimensions: positive (P), negative (N), and competence (C; Supplementary Tables 6 and 8). In general, previous research on human-robot relationships and interactions generally focused on studying and measuring specific psychological reactions to robots (e.g., safety, anthropomorphism, animacy, intelligence, likeability, various social attributes[203,204]) but did not attempt to identify all these reactions and investigate them under an all-encompassing construct of psychological processes. In that regard, the PNC model can be seen as an integrative framework that links and organizes an exhaustive list of psychological processes, both those that researchers have already studied separately and the less common ones that our participants generated. We believe that our model moves the field forward not only through this integration but also by enabling researchers to systematically study psychological processes regarding robots in three ways. First, our taxonomy could help them navigate these processes and identify the ones that may be linked. Second, the taxonomy could help them position their research within the appropriate PNC dimension and recognize other

processes potentially worth considering in their studies. Third, the PRR scale we developed (Supplementary Table 8) could be used to investigate which causal influences shape psychological processes in response to robots because it can measure these processes and thus capture how various experimental manipulations affect them. Ultimately, we hope these three advancements will lead to theoretical progress by making the research field more organized and easier to navigate, increasing the knowledge of which psychological processes are linked and why, and advancing the understanding of causal influences on these processes.

There are various insights that can be inferred from the PNC model regarding different psychological processes and their relationships. For example, feelings of creepiness or eeriness regarding a robot have traditionally been explained using the construct of the uncanny valley, according to which robots that closely resemble humans but fail to achieve a realistic human-like appearance evoke these feelings[3,205,206]. However, this construct has been criticized, and various studies have failed to support it[207,208]. In line with these studies, the present research did not yield evidence that would support the uncanny valley as an explanation of creepiness. Indeed, in our Study 4 (Supplementary Table 6), creepiness was classified in the same domain as characteristics that convey the non-human nature of robots, such as “not human” or “emotionless”, but also in the same domain as characteristics that convey the possibility that robots are destroying our basic humanness and our society (e.g., “societal issues,” “robots contribute to human degeneration”). These insights are inconsistent with the uncanny valley in two ways. First, they indicate that robots may be perceived as creepy when they have non-human characteristics rather than when they resemble humans but fail to fully achieve a realistic human-like appearance. Second, they indicate that creepiness is not linked only to robots’ appearance but also to the consequences that robots may have for humans and wider society. This is only one example of how the PNC model can be used to critically examine different constructs related to robots and inspire various ideas and observations to deepen our understanding of the psychological processes these constructs tackle.

One of the more interesting insights stemming from our model arises if we compare it to studies conducted outside the domain of technology. As part of their stereotype content model (SCM), Fiske, Cuddy, and Glick[209] showed that people form impressions of other humans alongside two dimensions: warmth (i.e., positive and negative social characteristics) and competence (i.e., a person’s ability to successfully accomplish tasks). Although our model is broader than the SCM because it comprises all psychological processes rather than only social and intellectual characteristics, the competence dimensions from the two models are thematically comparable, whereas the positive and negative attributes from the SCM’s warmth dimension are broadly aligned with our positive and negative dimensions.[210] These comparisons suggest that people use similar criteria when forming impressions of robots and humans, and that robots’ similarity to humans does not play a role in this regard since many of our stimuli depicted non-humanoid robots (Supplementary Table 7).

Interestingly, this insight has important implications for one of the most widely used frameworks regarding human relationship with technology—the media equation[211–213]. According to this framework, people interact with robots, computers, and other devices like they interact with humans and treat them as social actors. However, it remains unknown why exactly this is the case, and several researchers have speculated on potential explanations (see ref.[214]). The alignment between our PNC model and the SCM[209,210] indicates that people may treat robots as social actors because, on a fundamental cognitive level, they form impressions of robots and humans by considering the same criteria (i.e., by assessing whether robots/humans have any positive and/or negative qualities, and whether they are competent in what they do). In other words, upon encountering a robot, humans may form an impression of this robot that is organized along these criteria and contains various attributes that can be applied to both

robots and humans (e.g., being friendly or unfriendly, being more or less thoughtful, being more or less knowledgeable, etc.). If subsequent communication with the robot is guided by these attributes, it will naturally contain elements that apply to both humans and robots, thus creating the impression that people interact with robots like they interact with humans. Investigating this in more depth may lead to interesting insights about cognitive mechanisms that underpin the media equation.

Phase Three: Individual Difference Predictors and Mediators

Only a few studies that investigate the link between individual differences and psychological responses to robots have been conducted. In sum, this research found that extraversion and openness predicted positive responses to robots[215–217]; dispositional anthropomorphism, which was not measured directly but via its personality correlates (i.e., need for cognition, desire for control, and chronic loneliness), predicted positive attitudes toward service robots[218]; need for cognition itself also predicted less negative attitudes toward robots[219]; and animal reminder disgust, neuroticism, and religiosity predicted experiencing robots as eerie[220]. From these effects, the only one that the present research replicated was the positive relationship between anthropomorphism (measured directly via IDAQ[46] rather than via its personality correlates) and positive psychological processes regarding robots (see Supplementary Table 16), which is also aligned with extensive theorizing on anthropomorphism[45].

Although we cannot state that the remaining relationships between individual differences and the PNC dimensions our research identified were fully unexpected (Supplementary Tables 12 and 16), considering that we had a rationale behind each individual difference predictor (Supplementary Table 11), they were certainly not the most intuitive ones. For example, it could not have been anticipated that psychopathy[194] would be the most robust predictor of the negative dimension (Supplementary Table 16). Equally, we did not expect that security-societal[196] and approach temperament[97] would be the most robust predictors of the competence dimension, or that parental expectations[192] and general risk propensity[191] would be the most robust predictors of the positive dimension. Therefore, in line with the notion by Jack et al.[221], using a data-driven approach allowed us to generate unexpected insights, thus diversifying knowledge about the psychology behind human experiences of robots.

In addition to determining the most robust individual difference predictors, we made a major advancement in understanding the mechanisms, considering that previous studies generally did not delve into this sphere. Regarding the negative dimension, one overarching observation is that the mediators identified (Supplementary Table 17) are broadly linked to negative feelings stemming from one's ego being undermined. For example, a portion of the relationship between psychopathy[194] and the negative dimension was explained by the negative feelings toward other people's inventions and by the feelings of inferiority in relation to technology. Moreover, activated displeasure[197] (i.e., feeling upset, fearful, guilty, and ashamed) was a significant mediator for both psychopathy[194] and trait negative affect[193]. Concerning the positive dimension, the identified mediators (Supplementary Table 17) were somewhat more complex, and several themes emerged. One theme that underpins the relationship between general risk propensity[191] and this dimension involves finding the risks associated with robot adoption appealing. The second theme that relates to parental expectations[192] involves valuing robots because they are closer to perfection than humans and are helpful to participants in attaining their own high expectations. The third theme that concerns anthropomorphism[46] involves experiencing positive and strong emotions regarding robots. Finally, in relation to the competence dimension, the main theme that emerged across the identified mediators (Supplementary Table 17) was liking exceptional competencies and linking them to various broader consequences (e.g., how powerful society is).

Limitations

There are several limitations to this research. First, our stimuli were not physical robots but their depictions. This approach allowed us to sample a comprehensive range of robots across 28 exhaustive domains of human activity (Supplementary Table 7) and decrease the chance that our findings are driven by idiosyncrasies of a sample that is small in size and/or variety of robot types, which could compromise replicability[181,182]. Our stimuli also hold ecological validity in relation to how people typically encounter robots. For instance, among the 28 domains of human activity examined in this research (Supplementary Table 5), individuals may have direct exposure to robots in only a few of these domains (e.g., household, education, public services). Even within these domains, people may own only one or a few robots and directly interact with them (e.g., robotic pets, social robots, robotic vacuum cleaners). Conversely, it is plausible that people encounter most other robots and form opinions about them via social media or news websites in the form of visuals and descriptions. In this context, it is important to explain why we chose to depict robots via images accompanied with written descriptions rather than via other forms of visuals and descriptions (e.g., videos accompanied with narration). We did this to minimize the number of potential confounding effects associated with the stimuli. For example, whereas different videos covering the robots in question could have different durations and contain narrations of different lengths and styles, images allowed us to avoid the duration as a potential confound, and to equalize the length and styles of robot descriptions, thus also avoiding these elements as confounds.

However, despite the ecological validity of our stimuli and the advantages they have over other visuals such as videos, they did not involve actual physical robots, and various studies have shown that direct contact with robots can influence people's psychological responses[13,222,223]. In this respect, direct contact with the robots from our stimuli could potentially change the structure of our taxonomy (e.g., resulting in a model that has fewer or more factors than the PNC and includes different psychological processes). Alternatively, direct contact may not alter the taxonomy's structure but simply change how robots are evaluated on the PNC dimensions (e.g., as more or less positive, negative, or competent). These questions could be resolved by measuring psychological processes regarding all physical counterparts of the robots from our stimuli (Supplementary Table 7) and comparing them to the psychological processes uncovered in our research. Whereas this is currently not possible because of various practical reasons (e.g., inaccessibility of the robots from our stimuli due to their size, cost, limited production, potential use as weapons, etc.), it might change in the future as various robots, including the more specialized ones, become more widely available. At that point, it will be possible to expand the PNC model to physical robots by adopting the methodology used in the present research.

The second limitation is associated with qualitative research analyses, which are inevitably informed by the researcher's perspective or approach to interpretation, and replicability is typically not the aim[224]. Nevertheless, we took a few measures to overcome these weaknesses in the qualitative studies we conducted (i.e., Studies 1-3). To be precise, our sample sizes (Supplementary Table 1) were more than sufficient to reach data saturation[87–92], and coding of the data was done in a way that resulted in categories that to some degree overlap (e.g., Supplementary Tables 3 and 5) to ensure that no information is omitted. Most importantly, the advantages of combining qualitative and quantitative approaches considerably outweigh the limitations[224]. For example, joining the two approaches can lead to both a deeper understanding of the phenomena in question and more profound theoretical insights than quantitative research in isolation. In that regard, our discussion of inductive integration reveals the richness of insights that would not have been possible without using qualitative research methods.

Third, our participants were from WEIRD (i.e., Western, educated, industrialized, rich and democratic[225]) countries (US and UK). Since our research proposed and investigated a construct (i.e., psychological processes regarding representations of robots) from scratch, our priority was to establish its foundations. Combining the investigation of cultural differences with this agenda using equally

meticulous methods would have exceeded the scope of a single article. Nevertheless, as measurement invariance analyses showed that the PNC model applies to individuals regardless of their income, age, educational attainment, use of robots at work, political orientation, ethnic identity, employment status, and relationship status (Supplementary Tables 9-10), it is plausible that the model would generalize to countries different from the UK or US on these population characteristics. In other words, whether countries are rich or not, have liberal or conservative governments, have populations that differ in educational attainment and ethnic diversity, etc., might not matter for the structure of the PNC model. Overall, investigating whether these evidence-based inferences are accurate and generally examining the cultural generalizability of the PNC model will be a long-term project that may span a range of different studies and publications. Ideally, the first step would be sampling countries that are representative of the entire possible cultural space. For example, if Hofstede dimensions[226] are used to define cultural space, then countries across the spectrum of each of these dimensions (i.e., power distance, individualism vs. collectivism, masculinity vs. femininity, uncertainty avoidance, long vs. short-term orientation, and indulgence vs. restraint) would need to be selected. Second, for each of these countries, the construct of psychological processes regarding robots would ideally be tested using the rigorous methods employed in the present research, by first developing the robot definition and domains from participants' responses, then mapping a comprehensive content space of psychological processes regarding robots, followed by organizing these processes into a taxonomy, and finally determining their individual difference predictors and the mechanisms behind these predictors. Then, the countries would be compared to each other to understand whether any differences exist. Finally, if any differences are detected, it would be necessary to understand which variables explain them. For example, did these differences arise due to cultural variables (e.g., individualism vs. collectivism[226]) or some other influences (e.g., social, economic, environmental, political, etc.)? Conducting this type of in-depth examination of the generalizability of psychological processes regarding robots will be a crucial step as the research topic progresses.

Fourth, we employed mediation analyses without experimentally manipulating the predictors (i.e., individual differences) or the mediators, which means that strict causal claims regarding the mechanisms cannot be made[227,228]. However, personality traits generally cannot be manipulated, barring a major life event (e.g., long-term unemployment[229]), and the adopted approach is valuable in understanding the mechanisms because we followed several practices that are necessary to minimize the weaknesses of mediation in personality psychology[230]. First, in Study 7, the order of variables was aligned with the following temporal sequence: Individual Difference → Mediator → PRR scale. Indeed, we first measured the individual differences in wave 1, whereas the mediators were measured at the beginning of wave 2, before participants saw any robot examples, followed by the PRR scale (Supplementary Table 8). Second, we made sure that all mediators (Supplementary Table 17) were more specific in relation to a particular situation or aspect of dealing with a robot compared to the individual differences (Supplementary Table 11) that were more general and represented a higher level of participants' views and behavior. In that regard, it is plausible that these general patterns of feeling, thinking, and acting were reflected in the more specific instances captured by the mediators, which in turn played a role in even more specific psychological processes regarding the robots in question.

Fifth, we recruited online participants who are inherently more confident with technology. Whereas this might have influenced the findings, alternative modes of recruitment (e.g., participant pool of the university research lab) were suboptimal because they would have resulted in smaller and less representative samples (e.g., university students and staff members plus some volunteers who live in the proximity of the university), which would have by default compromised the power and generalizability of the findings[77–80,231]. Moreover, it is not a given that these modes of recruitment would have resulted in participants who have lower confidence with technology, considering that research

participation is often advertised online and participants such as students tend to use technology for their studies. However, it is important to point out that various analyses we conducted make it less plausible that technological proficiency biased the findings. For example, one of the covariates we used in the machine learning models was a variable indicative of technological proficiency involving robots (i.e., people's previous frequency of interaction with robots; Supplementary Tables 11-12).

Sixth, effect sizes for some of the key individual difference predictors we established were small (Supplementary Table 16). Considering that these predictors were initially identified amongst numerous individual differences using a meticulous procedure that relied on various machine learning models (for the description of the analytic approach, see the "Supplementary Methods" section for Study 6), this finding indicates that effect sizes for the relationships between individual differences and psychological processes regarding robots are small to medium at best. Therefore, no single individual difference may be dominant in shaping these processes, and various individual differences, including the most predictive ones we detected, may operate together in shaping them. Nevertheless, it is important to emphasize that recent research has found that traditional effect size classifications (in case of Cohen's f^2, .02 = small, .15 = medium, and .35 = large[176]) are inflated, considering that average effect sizes in personality psychology range from small to medium[232], and that it would be more appropriate to refer to traditionally medium effects as large[233]. In that respect, our effect sizes are aligned with a large body of personality psychology and comparable to the predictive power of individual differences in other contexts.

Finally, rapid technological development might make robots with a similar embodiment to humans able to perform and simulate all human activities, therefore significantly changing how people perceive robots. However, since our comparison of the PNC model and SCM[209,210] indicates that people form impressions of robots and humans in a similar manner, it is unlikely that robots becoming more like humans will have a significant impact on the structure of our model. Even if it does, the PNC can be updated via the same methodological procedures we used.

Supplementary Tables

Supplementary Tables 1 and 2: Comprehensive Participant Information

These tables contain comprehensive background information for participants from the present research. Supplementary Table 1 contains information that was collected in all studies (i.e., age, gender, employment status, and use of robots at work), including a breakdown of age categories, in combination with additional variables that were collected only in Study 6 (educational attainment, political orientation, relationship status, ethnic identity, and income). Supplementary Table 2 contains information about the regions in which participants lived (this information was collected only for participants from Studies 4-7). For studies 4-6, we requested from the recruiter to obtain samples that are representative in terms of age, gender, and geographical region, whereas for Study 1 (Sample 1), we aimed for representativeness in terms of gender. The rows "Representative UK" and "Representative US" contain the criteria that the recruiter used for the representative samples; these criteria were informed by Census (UK: England and Wales [www.ons.gov.uk/census], Scotland [www.scotlandscensus.gov.uk/], and Northern Ireland [www.nisra.gov.uk/statistics/census]), and by Census Bureau (US [www.census.gov]). We were informed by the recruiter there would be some discrepancies between these criteria and the demographic data we obtain due to some participants not updating the information in their database, and due to the quotas in their system registering participation only after a participant has finished the study, which means that for some categories more (or fewer) participants would be allowed to take part in the study than required for that category. However, despite these discrepancies, the samples would be reasonably representative of the general population for the variables in question. As can be seen from the tables, this was generally the case. Gender (Supplementary Table 1) in Studies 4-6 and Study 1 (Sample 1) was relatively evenly distributed, in line with the nationally representative criteria, and no gender was severely underrepresented. Concerning age (Supplementary Table 1), there were some discrepancies between specific categories and the representative criteria. However, the pattern of age distribution we obtained was similar to the one in the representative criteria in the sense that there were fewer participants in the youngest age bracket (18-24), whereas the other brackets tended to be somewhat more aligned. Similarly, for the region (Supplementary Table 2), there were some discrepancies between the obtained data and the representative criteria, but the patterns were reasonably aligned. For example, for the UK samples, there was generally a close correspondence between the obtained data and the representative categories. For the US samples, a substantially larger number of participants came from the South compared to the other regions, similar to the representative criteria (38%), whereas the other regions were more comparable amongst themselves. Therefore, the variables in question were reasonably representative of the target population.

Supplementary Table 1: Comprehensive Participant Information: Age, Gender, Employment Status, and Use of Robots at Work (Studies 1-7); and Educational Attainment, Political Orientation, Relationship Status, Ethnic Identity, and Income (Study 6)

Study	Sample N°	Sample Size	Country	Age		Age Brackets						Gender				Employment Status		Use of Robots at Work			Educational Attainment		Political Orientation			In a Relationship		Ethnic Identity		Income (in $)	
				M	SD	18-24	25-34	35-44	45-54	55-64	65+	Female	Male	Other	UD [a]	Employed	Unemployed	Don't know	No	Yes	Secondary or below	Higher education	Conservative	Liberal	Moderate	No	Yes	White	Another	M	SD
Representative UK				-	-	13.00%	17.00%	18.00%	17.45%	14.24%	20.31%	50.64%	49.36%	-	-	-	-	-	-	-	-	-	-	-	-	-	-	-	-	-	-
Representative US				-	-	13.00%	16.00%	17.00%	17.00%	17.00%	20.00%	51.00%	49.00%	-	-	-	-	-	-	-	-	-	-	-	-	-	-	-	-	-	-
All Participants																															
1	1	266	UK	49.496	13.598	3	45	57	61	58	42	132	133	1	0	175	91	3	161	11	-	-	-	-	-	-	-	-	-	-	-
						1.13%	16.92%	21.43%	22.93%	21.80%	15.79%	49.62%	50.00%	0.38%	0.00%	65.79%	34.21%	1.71%	92.00%	6.29%	-	-	-	-	-	-	-	-	-		
1	2	100	US	36.510	10.566	9	46	26	8	10	1	42	58	0	0	94	6	2	90	2	-	-	-	-	-	-	-	-	-	-	-
						9.00%	46.00%	26.00%	8.00%	10.00%	1.00%	42.00%	58.00%	0.00%	0.00%	94.00%	6.00%	2.13%	95.74%	2.13%	-	-	-	-	-	-	-	-	-		
2	-	70	US	36.257	10.270	4	34	18	10	2	2	31	39	0	0	64	6	1	55	8	-	-	-	-	-	-	-	-	-	-	-
						5.71%	48.57%	25.71%	14.29%	2.86%	2.86%	44.29%	55.71%	0.00%	0.00%	91.43%	8.57%	1.56%	85.94%	12.50%	-	-	-	-	-	-	-	-	-		
3	-	350	US	40.693	12.194	15	116	104	57	39	18	193	153	1	3	325	24	5	279	41	-	-	-	-	-	-	-	-	-	-	-
						4.29%	33.14%	29.71%	16.29%	11.14%	5.14%	55.14%	43.71%	0.29%	0.86%	92.86%	6.86%	1.54%	85.85%	12.62%	-	-	-	-	-	-	-	-	-		
4	1	1668	UK	47.932	16.611	157	274	313	297	275	352	852	812	4	0	1043	624	13	955	75	-	-	-	-	-	-	-	-	-	-	-
						9.41%	16.43%	18.76%	17.81%	16.49%	21.10%	51.08%	48.68%	0.24%	0.00%	62.53%	37.41%	1.25%	91.56%	7.19%	-	-	-	-	-	-	-	-	-		
4	2	1808	US	48.004	16.772	134	343	355	238	360	378	976	830	2	0	1053	754	14	871	168	-	-	-	-	-	-	-	-	-	-	-
						7.41%	18.97%	19.63%	13.16%	19.91%	20.91%	53.98%	45.91%	0.11%	0.00%	58.24%	41.70%	1.33%	82.72%	15.95%	-	-	-	-	-	-	-	-	-		
5	1	1200	UK	46.648	16.616	140	217	200	217	194	232	590	601	6	3	753	447	14	690	49	-	-	-	-	-	-	-	-	-	-	-
						11.67%	18.08%	16.67%	18.08%	16.17%	19.33%	49.17%	50.08%	0.50%	0.25%	62.75%	37.25%	1.86%	91.63%	6.51%	-	-	-	-	-	-	-	-	-		
5	2	1219	US	46.656	16.914	115	236	250	171	213	234	616	598	5	0	712	506	12	639	61	-	-	-	-	-	-	-	-	-	-	-
						9.43%	19.36%	20.51%	14.03%	17.47%	19.20%	50.53%	49.06%	0.41%	0.00%	58.41%	41.51%	1.69%	89.75%	8.57%	-	-	-	-	-	-	-	-	-		
6	-	2505	US	47.405	17.262	188	482	616	262	385	568	1299	1186	15	5	1537	964	19	1210	307	785	1717	729	827	940	840	1661	2123	358	70.924	46.773
						7.50%	19.24%	24.59%	10.46%	15.37%	22.67%	51.86%	47.35%	0.60%	0.20%	61.36%	38.48%	1.24%	78.72%	19.97%	31.34%	68.54%	29.10%	33.01%	37.52%	33.53%	66.31%	84.75%	14.29%		
7	-	1116	US	42.910	13.535	68	288	292	223	165	80	552	555	9	0	843	273	22	754	66	-	-	-	-	-	-	-	-	-	-	-
						6.09%	25.81%	26.16%	19.98%	14.78%	7.17%	49.46%	49.73%	0.81%	0.00%	75.54%	24.46%	2.61%	89.44%	7.83%	-	-	-	-	-	-	-	-	-		
Participants Included in Analyses																															
1	1	224	UK	50.344	13.262	3	29	48	57	51	36	121	102	1	0	145	79	3	136	6	-	-	-	-	-	-	-	-	-	-	-
						1.34%	12.95%	21.43%	25.45%	22.77%	16.07%	54.02%	45.54%	0.45%	0.00%	64.73%	35.27%	2.07%	93.79%	4.14%	-	-	-	-	-	-	-	-	-		
1	2	95	US	36.621	10.729	9	43	25	7	10	1	39	56	0	0	91	4	2	88	1	-	-	-	-	-	-	-	-	-	-	-
						9.47%	45.26%	26.32%	7.37%	10.53%	1.05%	41.05%	58.95%	0.00%	0.00%	95.79%	4.21%	2.20%	96.70%	1.10%	-	-	-	-	-	-	-	-	-		
2	-	67	US	35.657	9.634	4	33	18	9	2	1	31	36	0	0	61	6	1	52	8	-	-	-	-	-	-	-	-	-	-	-
						5.97%	49.25%	26.87%	13.43%	2.99%	1.49%	46.27%	53.73%	0.00%	0.00%	91.04%	8.96%	1.64%	85.25%	13.11%	-	-	-	-	-	-	-	-	-		
3	-	334	US	40.826	12.154	14	108	102	55	36	18	184	147	1	2	311	22	5	270	36	-	-	-	-	-	-	-	-	-	-	-
						4.19%	32.34%	30.54%	16.47%	10.78%	5.39%	55.09%	44.01%	0.30%	0.60%	93.11%	6.59%	1.61%	86.82%	11.58%	-	-	-	-	-	-	-	-	-		
4	1	1528	UK	48.328	16.515	141	235	284	281	259	328	790	734	4	0	944	583	12	874	58	-	-	-	-	-	-	-	-	-	-	-
						9.23%	15.38%	18.59%	18.39%	16.95%	21.47%	51.70%	48.04%	0.26%	0.00%	61.78%	38.15%	1.27%	92.58%	6.14%	-	-	-	-	-	-	-	-	-		
4	2	1537	US	49.465	16.563	99	253	289	212	333	351	861	674	2	0	870	667	11	745	114	-	-	-	-	-	-	-	-	-	-	-
						6.44%	16.46%	18.80%	13.79%	21.67%	22.84%	56.02%	43.85%	0.13%	0.00%	56.60%	43.40%	1.26%	85.63%	13.10%	-	-	-	-	-	-	-	-	-		
5	1	1107	UK	47.112	16.583	124	191	183	204	185	220	544	555	6	2	691	416	10	639	42	-	-	-	-	-	-	-	-	-	-	-
						11.20%	17.25%	16.53%	18.43%	16.71%	19.87%	49.14%	50.14%	0.54%	0.18%	62.42%	37.58%	1.45%	92.47%	6.08%	-	-	-	-	-	-	-	-	-		
5	2	1108	US	47.100	16.947	102	204	225	160	192	225	563	540	5	0	651	456	12	591	48	-	-	-	-	-	-	-	-	-	-	-
						9.21%	18.41%	20.31%	14.44%	17.33%	20.31%	50.81%	48.74%	0.45%	0.00%	58.75%	41.16%	1.84%	90.78%	7.37%	-	-	-	-	-	-	-	-	-		
6	-	2203	US	47.947	17.493	178	407	505	226	360	523	1164	1021	14	4	1316	883	16	1064	235	702	1498	639	731	825	761	1438	1876	304	68.855	46.046
						8.08%	18.47%	22.92%	10.26%	16.34%	23.74%	52.84%	46.35%	0.64%	0.18%	59.74%	40.08%	1.22%	80.85%	17.86%	31.87%	67.00%	29.01%	33.18%	37.45%	34.54%	65.27%	85.16%	13.80%		
7	-	1071	US	42.846	13.450	66	276	279	216	160	74	535	527	9	0	808	263	22	721	64	-	-	-	-	-	-	-	-	-	-	-
						6.16%	25.77%	26.05%	20.17%	14.94%	6.91%	49.95%	49.21%	0.84%	0.00%	75.44%	24.56%	2.72%	89.23%	7.92%	-	-	-	-	-	-	-	-	-		

Note. All studies were administered via Qualtrics. In Studies 1 (Sample 1), 4 (Samples 1 and 2), 5 (Samples 1 and 2), and 6, participants were recruited via Pureprofile. In Studies 1 (Sample 2), 2, and 3, participants were recruited via Amazon Mechanical Turk (MTurk). In Study 7, participants were recruited via Prolific.
For Employment Status, "employed" comprises participants who were either self-employed or working for an employer, whereas "unemployed" refers to participants who were not working for themselves or someone else. For Educational Attainment, "secondary or below" comprises participants who had either no formal qualifications or secondary education, whereas "higher education" comprises participants who had an undergraduate degree, graduate degree, doctoral degree, or professional qualification. For Political Orientation, "conservative" comprises participants who identified themselves as extremely conservative, conservative, or slightly conservative, whereas "liberal" comprises participants who identified themselves as extremely libera, liberal, or slightly liberal, and "moderate" refers to participants who identified themselves as moderate/middle of the road. For In a Relationship, "no" comprises participants who were single, divorced, or widowed, whereas "yes" comprises participants who were in a relationship, married, or in civil partnership. For Ethnic Identity, "white" comprises participants who identified themselves as white (American or other), whereas "Another" comprises participants who identified themselves as black (African American or other), Asian or Asian American, Hispanic or Latino, or mixed. Regarding Ethnic Identity, participants who did not select any of these options and identified themselves as "other" are not reported in the table because their number was minor (0.84% of all participants and 0.91% of participants included in analyses), and because for these participants, in many cases, clear ethnic identity was not expressed.
[a] For Gender, "UD" stands for undisclosed; this category comprises participants who either did not want to disclose their gender or whose data were missing. In some instances, for Age Brackets, Employment Status, Educational Attainment, Political Orientation, In a Relationship, and Ethnic Identity, the number of participants from their subordinate categories does not add up to the number in the column Sample Size: this is because of missing data (or, in the case of Ethnic Identity, because of missing data in combination with the data of the very few participants who identified themselves as other and did not select any of the offered options for this variable). Similarly, for Use of Robots at Work, in some instances the number of participants from the subordinate categories does not add up to the number that corresponds to Employed Participants under Employment Status; this is because of missing data. In all cases, the amount of missing data was low and did not exceed 0.36%.

Supplementary Table 2: Comprehensive Participant Information: Breakdown of the UK/US Regions in which Participants Lived (Studies 4-7)

Study	Sample N°	Sample Size	Country	UK Regions													US Regions				
				East	East Midlands	London	North East	Northern Ireland	North West	Scotland	South East	South West	Wales	West Midlands	Yorkshire and the Humber	Other	Midwest	Northeast	South	West	Other
Representative UK				9.29%	7.17%	13.13%	4.07%	2.85%	11.08%	8.31%	13.72%	8.39%	4.81%	8.85%	8.33%	-	-	-	-	-	-
Representative US				-	-	-	-	-	-	-	-	-	-	-	-	-	20.90%	17.30%	38.00%	23.80%	-
All Participants																					
4	1	1668	UK	137	92	215	69	51	182	152	270	136	75	150	135	4	-	-	-	-	-
				8.21%	5.52%	12.89%	4.14%	3.06%	10.91%	9.11%	16.19%	8.15%	4.50%	8.99%	8.09%	0.24%	-	-	-	-	-
4	2	1808	US	-	-	-	-	-	-	-	-	-	-	-	-	-	376	384	684	354	9
				-	-	-	-	-	-	-	-	-	-	-	-	-	20.80%	21.24%	37.83%	19.58%	0.50%
5	1	1200	UK	96	82	162	48	34	123	107	153	86	70	135	103	1	-	-	-	-	-
				8.00%	6.83%	13.50%	4.00%	2.83%	10.25%	8.92%	12.75%	7.17%	5.83%	11.25%	8.58%	0.08%	-	-	-	-	-
5	2	1219	US	-	-	-	-	-	-	-	-	-	-	-	-	-	278	254	454	230	3
				-	-	-	-	-	-	-	-	-	-	-	-	-	22.81%	20.84%	37.24%	18.87%	0.25%
6	-	2505	US	-	-	-	-	-	-	-	-	-	-	-	-	-	538	586	985	387	7
				-	-	-	-	-	-	-	-	-	-	-	-	-	21.48%	23.39%	39.32%	15.45%	0.28%
7	-	1116	US	-	-	-	-	-	-	-	-	-	-	-	-	-	250	282	435	149	0
				-	-	-	-	-	-	-	-	-	-	-	-	-	22.40%	25.27%	38.98%	13.35%	0.00%
Participants Included in Analyses																					
4	1	1528	UK	129	86	189	65	47	166	139	248	129	68	134	124	4	-	-	-	-	-
				8.44%	5.63%	12.37%	4.25%	3.08%	10.86%	9.10%	16.23%	8.44%	4.45%	8.77%	8.12%	0.26%	-	-	-	-	-
4	2	1537	US	-	-	-	-	-	-	-	-	-	-	-	-	-	317	324	602	289	4
				-	-	-	-	-	-	-	-	-	-	-	-	-	20.62%	21.08%	39.17%	18.80%	0.26%
5	1	1107	UK	87	76	146	46	30	114	96	145	84	66	122	94	1	-	-	-	-	-
				7.86%	6.87%	13.19%	4.16%	2.71%	10.30%	8.67%	13.10%	7.59%	5.96%	11.02%	8.49%	0.09%	-	-	-	-	-
5	2	1108	US	-	-	-	-	-	-	-	-	-	-	-	-	-	257	235	406	208	2
				-	-	-	-	-	-	-	-	-	-	-	-	-	23.19%	21.21%	36.64%	18.77%	0.18%
6	-	2203	US	-	-	-	-	-	-	-	-	-	-	-	-	-	486	501	877	334	4
				-	-	-	-	-	-	-	-	-	-	-	-	-	22.06%	22.74%	39.81%	15.16%	0.18%
7	-	1071	US	-	-	-	-	-	-	-	-	-	-	-	-	-	241	268	417	145	0
				-	-	-	-	-	-	-	-	-	-	-	-	-	22.50%	25.02%	38.94%	13.54%	0.00%

Note. All studies were administered via Qualtrics. In Studies 4 (Samples 1 and 2), 5 (Samples 1 and 2), and 6, participants were recruited via Pureprofile. In Study 7, participants were recruited via Prolific. If sample sizes across UK or US regions do not add up to the number in the column Sample Size, this is because of missing data. The amount of missing data was low (≤ 0.08%).

Supplementary Table 3: Robot Characteristics Generated by Sample 1 and Arranged into the Clusters Using Hierarchical Clustering on Sample 2 (Study 1)

Cluster 1: Degree of Robot-Human Similarity	Cluster 2: Positive Characteristics	Cluster 3: Robots' Composition	Cluster 4: Negative Characteristics	Cluster 5: Ability to Perform Various Tasks
▪ ability to add parts	▪ accurate	▪ apparatus	▪ awkward movements	▪ performs actions
▪ able to move things on its own	▪ adaptable	▪ comprised of hardware	▪ bland	▪ performs boring tasks
▪ animal-like	▪ agile	▪ comprised of software	▪ boring	▪ performs certain actions
▪ appears to think on its own	▪ capable	▪ computer	▪ cold	▪ performs certain functions
▪ artificial	▪ cheerful	▪ computer driven	▪ creepy	▪ performs certain tasks
▪ artificial intelligence	▪ clever	▪ computerized	▪ dangerous	▪ performs certain tasks independently
▪ artificial life form	▪ committed	▪ constructed	▪ emotionless	▪ performs complex actions
▪ automated	▪ competent	▪ device	▪ error prone	▪ performs complex movements
▪ automated movement	▪ complex	▪ digital	▪ has digital voice	▪ performs complex tasks
▪ automatic	▪ consistent	▪ electric	▪ has no memories	▪ performs complicated tasks
▪ automation	▪ correct	▪ electronic	▪ has sci-fi voice (slow, chunky, shiny, computerized)	▪ performs everyday tasks
▪ automaton	▪ credible	▪ hard	▪ has strange voice	▪ performs hard work over long periods of time
▪ autonomous	▪ cute	▪ has computing power	▪ has weird voice	▪ performs high quality tasks
▪ boredom relief	▪ dexterous	▪ has sensors	▪ impersonal	▪ performs household tasks
▪ can be 'scrapped' once no longer needed	▪ dynamic	▪ has wheels	▪ inanimate	▪ performs human actions
▪ can learn	▪ efficient	▪ has wires	▪ inflexible	▪ performs human tasks
▪ can produce somethings without having to wait for humans	▪ effortless	▪ heavy	▪ limited	▪ performs human tasks more efficiently
▪ cheap	▪ entertaining	▪ item	▪ mechanical movements	▪ performs manual tasks
▪ conditioned	▪ essential	▪ jointed	▪ monotone	▪ performs many tasks
▪ control	▪ exciting	▪ machine	▪ moves weirdly	▪ performs monotonous tasks
▪ controllable	▪ faithful	▪ machinery	▪ no conscience	▪ performs multiple tasks
▪ controlled	▪ fast	▪ man-made	▪ non-human	▪ performs non-stop
▪ cost efficient	▪ flexible	▪ manufactured	▪ non-humorous	▪ performs physical tasks
▪ cyberman	▪ focused	▪ mechanical capability	▪ non-intelligent	▪ performs predetermined actions
▪ cyborg	▪ friendly	▪ mechanical limbs	▪ non-interactive	▪ performs predetermined tasks
▪ udependent on power supply	▪ functional	▪ metal-like	▪ non-living	▪ performs repetitive tasks
▪ developmental	▪ funny	▪ metal-made	▪ performs stiff movements	▪ performs repetitive tasks efficiently
▪ different	▪ great-looking	▪ metallic	▪ potentially dangerous	▪ performs routine tasks

- directed
- doesn't get bored
- doesn't get tired
- doesn't need a pension or sickness benefit
- eases everyday life problems
- eases everyday tasks
- eases human tasks
- economic
- enabling senses
- engineered
- enhances human interaction
- entity
- expensive
- fixes things
- follows order
- futuristic
- helps humans
- helps to build cars
- hi-tech
- human operator
- human substitute
- human task substitute
- human-like
- humanoid
- innovative
- insular
- latest technology
- learns
- learns certain actions
- media reference (e.g., books, tv shows, films)
- memory control
- methodical
- modern
- needs default settings
- no holidays
- no meals
- no sick leave - within reason (maintenance / repair)
- no sickness

- handy
- hard-working
- helpful
- inexhaustible
- intelligent
- interactive
- interesting
- intuitive
- leading
- logical
- masculine
- mobile
- mobility
- moves
- multipurpose
- powerful
- practical
- precise
- quick
- rational
- reliable
- robust
- safe
- skillful
- smart
- speedy
- straightforward
- strong
- supporting
- task-oriented
- time-saving
- tough
- useful

- micro-chip
- motorized
- object
- shiny
- silver
- tinny
- tool
- unit
- mechanical

- repetitive
- rigid
- robotic voice
- rude
- scary
- slow
- slow-moving
- sometimes scary
- soulless
- stiff
- stiff movement
- threatening
- unaware
- unreal
- unreliable
- unthoughtful
- worrying

- performs set tasks
- performs specific actions
- performs specific tasks
- performs specifically designed tasks
- performs tasks
- performs tasks electronically
- performs tasks independently
- performs tasks without human help
- replicates everyday tasks

- no strikes
- novel
- obedient
- passive
- pre-programmed
- programmable
- programmed
- regimented
- remotely controlled
- repairable
- replacement
- replicates human actions
- replicates human movement
- sci-fi
- scientific
- scientifically engineered
- self-learning
- self-sufficient
- semi-autonomous
- sensing
- simple
- slave
- standard
- structured
- talks
- technical
- technology
- thinks
- toy
- used in factories
- used in production lines

Supplementary Table 4: Definition of Robots Developed from the Clusters of Their Characteristics (Study 1)

	Definition	Robot Clusters that Informed the Definition [a]
Part 1	A robot is a non-living entity that primarily functions as helping and/or substituting humans in some capacity by performing physical and/or intellectual tasks that range from simple, routine to complex ones.	1, 4, & 5
Part 2	Robots are characterized by different degrees of autonomy: sometimes they only follow commands that have been pre-programmed, but sometimes they are artificially intelligent and thus are able to learn from the environment and adapt to it.	1
Part 3	Although robots at times require maintenance and repair, they have potential to work tirelessly over long periods of time as they do not have life commitments (e.g., family) and/or wellbeing considerations as humans do (e.g., time off, sick leave).	1
Part 4	Humans can perceive robots as having positive attributes (e.g., clever, consistent, cute, efficient, flexible, friendly, reliable, robust, safe, supportive). However, humans also can have negative perceptions about robots, such as seeing them as cold, creepy, emotionless, lacking conscience, soulless, threatening, etc., generally attributing negative qualities to robots as a result of their nonhuman nature.	2 & 4
Part 5	A robot typically consists of software (i.e., the code or program on which it runs) and different materials and components used to produce it (e.g., metal, wires, sensors, micro-chips, etc.). Although robots can take the form and/or have characteristics of humans, they can appear as an animal or any non-living object.	1 & 3

[a] Cluster 1—Characteristics Conveying the Degree of Robot-Human Similarity; Cluster 2—Positive Characteristics; Cluster 3—Characteristics Conveying Robots' Composition; Cluster 4—Negative Characteristics; Cluster 5—Characteristics Conveying Robots' Ability to Perform Various Tasks (for details, see Supplementary Table 3).

Supplementary Table 5: Robot Domains and Example Items for Each Domain (Study 2)

Domain	Example Items
1. Health and human care and wellbeing (e.g., medical, surgical, fitness, lab diagnostics, elderly, disability, infant/child, and personal care)	▪ elder care in home; disability assistance; at the doctor or hospital; in hospitals, surgeries; lab diagnostics; exercise
2. Social and companionship	▪ social media; companionship; social life
3. Sex	▪ Note. This domain was added based on ref.[2] and was not generated from participants' items
4. Animal care (e.g., walking pets)	▪ animal care; walking pets; walking the dog
5. Security and surveillance	▪ home monitoring; night watchmen; public safety; security
6. Policing and military	▪ swat team alternatives/aids; warfare; battle bots; policing robots; protection/military/law enforcement; military applications; search and rescue
7. Education, libraries, and knowledge/information management and gathering	▪ ask for directions; child education; educational (school); finding information; library; studying
8. Research and exploration within science, technology, engineering, and mathematics (STEM) (e.g., ocean exploration, supercomputing, IT innovation, space discovery)	▪ research/exploration; science; space exploration; technology; computation; academic labs; programming; IT; analyzing
9. Communication tools and channels	▪ answering service; calling; chatbots; communications; customer service desk; smart phone; phone customer support chat
10. Leisure, recreation, and travel	▪ travel agency; travel; watercraft
11. Culture/entertainment, gaming, toys, and other amusement	▪ amusement park; casino; computer games; concerts; entertainment; gaming; in a theme park; movie theater
12. Workplace domain (i.e., to aid or replace human effort)	▪ robots that process documents; work product generation (collation of documentation for example); work
13. Dangerous and/or risky work	▪ do dangerous work
14. Inspection, repair, and/or improvement of products, engines, equipment, technology, and/or infrastructure (e.g., buildings, bridges, roads, power supplies, nuclear reactors, pipes, gas mains)	▪ repairing items; repairs; auto repair; auto maintenance
15. Agriculture (e.g., harvesting, farms)	▪ agriculture; farms; field harvesting

16. Household chores/tasks and domestic help/assistance (i.e., inside and outside of the home)	▪ automatic vacuum; cleaner/cleaning; domestic; giving out house orders for the smart house; home; house chores; personal helper; helping; providing assistance
17. Industry	▪ factory/factories; lifting heavy items; welding like car factories; working in factories; assemblers
18. Hospitality and food service (i.e., hotels, conventions, restaurants, bars, and other lodging, space, food and/or drink provider) and related customer service and support	▪ bartending; convention center; eating out; food preparation; food service; hospitality; reception; restaurants; service
19. Banking/financial services and related customer service and support	▪ talking to you bank teller; accuracy in money handling; ATM; bank; business; financial institutions
20. Retail and commerce and related customer service and support	▪ getting groceries; going to a store and shopping for you; grocery store; online shopping; ordering food at a kiosk at a restaurant; retail; self-checkout
21. Construction	▪ building construction; construction; construction sites; demolition
22. Manufacturing	▪ manufacturing in factories; manufacturing; manufacturing facilities
23. Mining	▪ mining
24. Warehouses and fulfilment centers	▪ counting items in inventory like Walmart; use it at a supermarket to stock goods overnight; warehouse work
25. Public services (e.g., road work and other shared public good)	▪ roadwork
26. Transportation (i.e., land, water, and/or sky) of goods, people, and other living entities, transport equipment, and delivery/courier/shipping services	▪ air travel; automated transportation; autonomous driving; drone delivery; courier services; public transportations; transport
27. Airports	▪ airports; use it at an airport to help people or give information
28. Art	▪ Note. This domain was added based on ref.[4] and was not generated from participants' items.

Note. Our aim was to develop domains that are narrow rather than broad, which means that some overlap between them may be present. This approach was aligned with our objective to establish a comprehensive content space of all robots to decrease the probability of using a biased stimulus sample[181,182] when developing the taxonomy of psychological responses to representations of robots. For that reason, it was more optimal to lean toward having too many rather than too few domains to reduce the chance of failing to cover the content space of all robots in detail and omitting important types of robots.

Supplementary Table 6: Summary of Key Findings (Studies 3 and 4): Psychological Processes, Items Corresponding to Each Process, and the Output of Exploratory Factor Analyses (EFAs) Performed on the Items across Two Participant Samples

№	Psychological Process	Item	Sample 1 (UK)			Sample 2 (US)		
			P	N	C	P	N	C
61	Companionship	This robot would make a good companion.	0.853			0.864		
84	Enjoyment	**I associate this robot with enjoyment.**	0.819			0.815		
85	Humor	This robot is humorous.	0.806			0.711		
35	Attachment	**I would feel attached to this robot.**	0.793			0.864		
26	Treating the robot like a human	I would treat this robot as if it were a human.	0.779			0.814		
34	Intimacy	I would be able to connect on an intimate level with this robot (e.g., share feelings, be in close contact, hug or hold, etc.).	0.775			0.842		
60	Comfort	This robot is comforting.	0.772			0.792		
65	Friendliness	This robot is friendly.	0.763			0.764		
116	Empathy	**This robot is empathetic**.	0.753			0.751		
88	Thoughtfulness	**This robot is thoughtful.**	0.745			0.793		
122	Entertainment	This robot is entertaining.	0.724			0.700		
123	Play	I would like to play with this robot.	0.715			0.742		
62	Interaction	I would want to interact with this robot.	0.693			0.663		
40	Engagement	**I would like to engage with this robot.**	0.692			0.689		
32	Happiness	**This robot makes me feel happy.**	0.681			0.806		
41	Motivation	This robot motivates me.	0.681			0.796		
27	Perceiving the robot as human-like	**This robot is like a human.**	0.675			0.743		
63	Communication	**I would find it easy to communicate with this robot.**	0.642			0.685		
67	Wellbeing	This robot promotes wellbeing.	0.635			0.685		
24	Anthropomorphism	I can see human traits in this robot.	0.625			0.681		
77	Pleasantness	I find this robot pleasant.	0.623			0.689		
149	Robot rights	**I think this robot should have rights.**	0.609			0.711		
95	Relaxation	This robot makes me feel relaxed.	0.608			0.742		
66	Care	**This robot provides care.**	0.600			0.675		
132	Self-improvement	**This robot helps me to improve myself.**	0.597			0.773		
14	Excitement	This robot makes me feel excited.	0.590			0.734		
125	Creativity	**This robot is creative.**	0.573			0.615		

89	Learning from robots	I could learn from this robot.	0.567			0.648		
107	Empowerment	**I feel empowered by this robot.**	0.545			0.691		
47	Pride	I feel proud about this robot.	0.541		0.343	0.691		
64	Social Support	This robot provides support to me.	0.534			0.633		
129	Competition	I would want to compete with this robot.	0.532	0.420		0.496	0.471	
96	Gratitude	**This robot makes me feel grateful.**	0.526			0.683		
99	Gaining knowledge about robot	I would want to learn more about this robot.	0.504			0.552		
46	Hope	This robot makes me feel hopeful.	0.501		0.371	0.669		
57	Safety	This robot makes me feel safe.	0.499			0.641		
42	Admiration	I admire this robot.	0.495		0.333	0.704		
83	Surprise	This robot surprises me.	0.495			0.531		
39	Interest	I would be interested in this robot.	0.486			0.572		
17	Awareness	**This robot has awareness.**	0.482			0.576		
143	Liberation	This robot makes me feel liberated or free.	0.481		0.324	0.686		
68	Cooperation	This robot and I could cooperate.	0.457			0.490		0.330
98	Information search	This robot provides me information.	0.456			0.479		
86	Artificial intelligence	This robot is intelligent.	0.421			0.511		
9	Need fulfilment	This robot fulfils my needs.	0.409		0.383	0.659		
146	Testing the robot	I would experiment with or test this robot to see what it can do.	0.402			0.399		
71	Openness	I would be open to this robot.	0.390	-0.348		0.450		0.323
87	Learning	This robot can learn.	0.382			0.437		
130	Winning	I want to beat or outperform this robot.	0.378	0.481			0.590	
100	Being knowledgeable about robot	I am knowledgeable about this robot.	0.364			0.483		
82	Positive affect	I feel positive about this robot.	0.359	-0.424	0.366	0.519		0.342
45	Uniqueness	This robot is unique.	0.354			0.335		
18	Ignoring	I would ignore this robot.	-0.324	0.352		-0.379	0.435	
19	Monotony	This robot deals with monotonous and repetitive tasks.	-0.362		0.623			0.559
38	Indifference	I am indifferent toward this robot.	-0.388			-0.344		
29	Instrumentality	This robot is just a means to an end.	-0.435		0.329	-0.412		0.374
28	Objectification	This robot is merely an object.	-0.464			-0.554		
37	Emotionless (human)	I feel no emotions toward this robot.	-0.533			-0.573		
23	Not human	This robot does not feel or respond like humans.	-0.547			-0.614		0.402
36	Emotionless (robot)	This robot is emotionless.	-0.715		0.384	-0.662		0.473
13	Anxiety	**This robot makes me feel anxious.**		0.806			0.693	
59	Threat	**I feel threatened by this robot.**		0.804			0.755	

119	Self-doubt	This robot makes me feel insecure (or doubt myself).	0.793			0.722	
49	Being upset	This robot upsets me.	0.772			0.746	
12	Stress	This robot makes me feel stressed.	0.769			0.731	
102	Fear	I am afraid of this robot.	0.763			0.723	
78	Unpleasantness	This robot makes me feel unpleasant.	0.745			0.749	
25	Dehumanization	**I would feel dehumanized when interacting with this robot.**	0.742			0.708	
50	Anger	This robot angers me.	0.727			0.753	
103	Creepiness	This robot is creepy.	0.72			0.690	
114	Freedom restriction	**This robot restricts or limits me.**	0.718			0.698	
30	Sadness	This robot makes me feel sad.	0.714			0.717	
58	Danger	This robot is dangerous.	0.710			0.672	
111	Boycott	I would ban the use of this robot.	0.701			0.722	
72	Negative affect	**I feel negative toward this robot.**	0.686			0.699	
117	Insignificance	This robot can make humans feel insignificant or not needed.	0.685	0.441		0.589	0.354
140	Disconnection	This robot disconnects humans from one another.	0.675			0.630	
135	Confusion	**This robot makes me feel confused.**	0.671			0.710	
136	People judging the use of robots	I think using this robot is wrong.	0.670			0.727	
33	Loneliness	This robot makes me feel lonely.	0.669			0.671	
115	Societal issues	**This robot can have negative social implications.**	0.661			0.614	
81	Dissatisfaction	This robot brings me dissatisfaction.	0.660			0.697	
92	Being gross	This robot is gross.	0.660			0.682	
148	Existential questioning	This robot makes me question life and existence.	0.659		0.352	0.631	
113	Immorality	**This robot is immoral.**	0.657			0.657	
126	Privacy	**This robot violates privacy (e.g., is too intrusive or invasive).**	0.656			0.693	
104	Humans lacking control	**I would lack or lose control when using or interacting with this robot.**	0.655			0.650	
76	Abnormal	**This robot is abnormal.**	0.645			0.610	
91	Disgust	**This robot is disgusting.**	0.641			0.695	
48	Damage reputation	**This robot could damage my reputation.**	0.637			0.671	
79	Hate	I would hate dealing with this robot.	0.636			0.651	
147	Human being tired of robot	This robot makes me feel tired or exhausted.	0.631			0.688	
11	Avoidance	I would avoid this robot.	0.630			0.604	
52	Protection of self	**I would want to protect myself when interacting with this robot.**	0.612			0.585	
134	Mixed feelings	I would have mixed feelings toward this robot.	0.607			0.628	
118	Replacement	This robot can make humans feel replaced.	0.600	0.501		0.533	0.418

74	Shyness	I would feel shy around this robot.	0.599			0.643	
31	Guilt	This robot makes me feel guilty.	0.594			0.638	
145	Embarrassment	**I would feel embarrassed or ashamed if I had to interact with this robot.**	0.579			0.719	
142	Robots contribute to human degeneration	**This robot can contribute to human degeneration (e.g., make people become lazy, use less of their mental and physical capacity, etc.).**	0.570			0.559	
1	Impatience	I would feel impatient when interacting with this robot.	0.559			0.647	
105	Unpredictability	This robot is unpredictable.	0.558			0.549	
51	Redundancy	This robot will make human jobs redundant.	0.536	0.535		0.490	0.380
21	Robot damage	I would be inclined to harm or damage this robot.	0.535		0.320	0.633	
112	Unethical activities	This robot could be used for unethical activities.	0.528			0.478	
73	Disappointment	This robot is disappointing.	0.525	-0.342		0.663	
16	Caution	I would be cautious or careful with this robot.	0.499			0.381	
22	Verbal abuse of robots	I would likely be verbally abusive toward this robot.	0.483			0.632	
139	Dependence (on robots or technology)	This robot creates dependence in humans.	0.447	0.388		0.416	
120	Human interaction substitute	This robot substitutes human interaction.	0.429			0.336	
138	Uselessness	This robot is useless.	0.405	-0.564		0.600	-0.348
131	Social comparison	I compare whether this robot is better than humans.	0.345	0.400		0.368	
133	Boredom	This robot is boring.	0.343			0.478	
3	Inefficiency	This robot is inefficient in what it does.	0.330	-0.410		0.484	
144	Authentic self	I can be my authentic self around this robot.	-0.373				0.374
10	Confidence	I would feel confident in this robot.	-0.375	0.466	0.412		0.426
80	Satisfaction	I am satisfied with this robot.	-0.379	0.403	0.483		0.371
69	Coexistence	I could coexist with this robot.	-0.381				
121	Trust	I would trust this robot.	-0.393	0.342	0.523		
70	Acceptance	I would be accepting of this robot.	-0.491	0.333	0.429	-0.343	0.372
4	Performance	**This robot can effectively achieve a certain result or a specified outcome.**		0.734			0.669
5	Usefulness	This robot is useful.		0.703			0.603
6	Help	This robot is helpful.		0.662			0.592
7	Accuracy	**This robot is accurate in what it does.**		0.642			0.570
2	Complexity	**This robot can do complex tasks.**		0.641			0.484
8	Financial costs	**This robot reduces costs.**		0.632			0.545
54	Speed	**This robot is fast at what it does.**		0.605			0.527
93	Future orientation	I think this robot is the future.		0.603			0.488

94	Progress	I associate this robot with progress.			0.583			0.517
109	Social good	This robot is a benefit to society.			0.545	0.424		0.400
128	Time freedom	This robot frees up my time to do other things.			0.531	0.375		0.417
55	Level of advancement	**This robot is advanced.**			0.516			0.526
127	Easier life	This robot makes my life easier.			0.516	0.478		0.392
43	Being impressed	This robot impresses me.			0.510	0.412		0.409
110	Corporate social responsibility (CSR)	Any benefits gained from this robot should be shared with or passed onto society.			0.504			0.428
141	Robots augment human capabilities	This robot can augment human capabilities.			0.471			0.424
20	Endurance	This robot has endurance (e.g., never tires, runs nonstop, etc.).			0.462			0.580
101	Monitoring	I would monitor this robot to make sure it functions properly.			0.426			0.389
15	Human alertness	I would feel alert with this robot.			0.398			0.397
97	Bias	This robot is not biased.			0.387			0.440
53	Robot superiority	This robot is superior to humans.			0.363	0.353		
44	Novelty	This robot is novel.						
56	Human superiority	Whenever I am given a choice, I will choose a human over this robot.						
75	Unusualness	This robot is unusual.						
90	Cleanliness	I would find this robot sanitary.						0.360
106	Dominance	I am dominant over this robot.						
108	Humans having control	I would have control over this robot.						
124	Autonomy	This robot is autonomous.						
137	Objectivity	This robot is objective.						
		Variance Explained (%)	15.995	17.891	10.422	20.736	17.224	8.586
		Eigenvalues	23.832	26.658	15.529	30.897	25.663	12.793
		P	-			-		
		N	-0.192	-		-0.192	-	
		C	0.471	-0.417	-	0.470	-0.245	-

Note. Labels P, N, and C refer to the dimensions (i.e., factors) that comprise Positive, Negative, and Competence-related psychological processes regarding robots. Values under each factor correspond to standardized factor loadings; only loadings with absolute values ≥ 0.320 are reported for clarity. The psychological processes and the corresponding items are ordered according to the item loadings on the three factors, whereas Nº corresponds to the number they were assigned when they were created. The items in bold were the ones selected for the PRR scale that was tested in Study 5 (see Supplementary Table 8). The coefficients for factors P-N-C at the bottom of the table denote correlations between the factors. All items were scored on a 7-point Likert scale (1=Strongly disagree, 7=Strongly agree).

Supplementary Table 7: Robot Examples Used as Stimuli (Studies 4-7)

This table lists robots that were depicted in stimuli used in Studies 4-7. There were two sets of stimuli: A and B. In Study 4, only Stimulus Set A was used, whereas in Studies 5-7 both stimulus sets were used. In all these studies (i.e., 4-7), participants were randomly allocated to one robot example. Each stimulus consisted of an image of a robot and a description of this robot, approximately eight lines long, presented below the image. The stimuli are not displayed in Supplementary Information for copyright reasons, but they are available from the corresponding author upon request.

Domain	Robot
Stimulus Set A: Robot Examples Used as Stimuli in Studies 4-7	
1. Health and human care and wellbeing (e.g., medical, surgical, fitness, lab diagnostics, elderly, disability, infant/child, and personal care)	▪ Da Vinci Surgical System: a robot designed to facilitate surgery using a minimally invasive approach.
2. Social and companionship	▪ Buddy: the first emotional companion robot.
3. Sex	▪ Tanya (female robot): an AI driven, life-size robotic doll system for human pleasure and companionship.<br>▪ Johnny (male robot): an AI driven, life-size robotic doll system for human pleasure and companionship.
4. Animal care (e.g., walking pets)	▪ CleverPet: the world's first game robot created for dogs.
5. Security and surveillance	▪ Knightscope K5: a security robot that can detect, report, and deter crime.
6. Policing and military	▪ AVATAR: a tactical robot used by the special weapons and tactics (SWAT) teams to inspect dangerous situations.
7. Education, libraries, and knowledge/information management and gathering	▪ NAO: a small humanoid tutor robot for children.
8. Research and exploration within science, technology, engineering, and mathematics (STEM) (e.g., ocean exploration, supercomputing, IT innovation, space discovery)	▪ Adam: a robot scientist that can autonomously perform experiments, test hypotheses, and interpret findings.
9. Communication tools and channels	▪ Alexa[a]: a virtual assistant AI technology typically used in Echo smart speakers.
10. Leisure, recreation, and travel	▪ Travelmate: the first fully autonomous suitcase.
11. Culture/entertainment, gaming, toys, and other amusement	▪ Aibo: a robotic dog that has the capability to interact with its human owner in many of the same ways a living pet would.
12. Workplace domain (i.e., to aid or replace human effort)	▪ Robo-C: an android that can serve in a business capacity.

13. Dangerous and/or risky work	▪ The Guardian XO Full-body Exoskeleton: a robot that allows the operator to effortlessly and securely lift and control objects weighing up to 200 lbs. (90 kg).
14. Inspection, repair, and/or improvement of products, engines, equipment, technology, and/or infrastructure (e.g., buildings, bridges, roads, power supplies, nuclear reactors, pipes, gas mains)	▪ Spot: a highly maneuverable mobile robot capable of navigating diverse terrains with exceptional agility, offering the potential to automate repetitive inspection tasks.
15. Agriculture (e.g., harvesting, farms)	▪ AVO: an autonomous robot that can be used to weed row crops, meadows, and intercropping cultures.
16. Household chores/tasks and domestic help/assistance (i.e., inside and outside of the home)	▪ Dyson 360 Heurist: a robot vacuum cleaner.
17. Industry	▪ KR AGILUS: a robot that can operate at high working speeds and carry out various industry-specific functions (e.g., gluing, moving, machining).
18. Hospitality and food service (i.e., hotels, conventions, restaurants, bars, and other lodging, space, food and/or drink provider) and related customer service and support	▪ Connie: a robot concierge developed for the hospitality industry.
19. Banking/financial services and related customer service and support	▪ Pepper: a semi-humanoid robot that can engage with people through conversations and its touch screen, and it offers services such as click and collect, payment, etc.
20. Retail and commerce and related customer service and support	▪ DAL-e (i.e., "Drive you, Assist you, Link with you"-experience): a customer service robot designed to communicate autonomously with individuals and assist them.
21. Construction	▪ Jaibot: a semi-autonomous drilling robot that can work for up to 8 hours between charges.
22. Manufacturing	▪ FANUC R-2000: a manufacturing robot that can handle various grippers or welding guns at speed and is suited to a large range of applications (e.g., manufacturing cars).
23. Mining	▪ Komatsu 980E: an autonomous self-driving mining truck that carries ore.
24. Warehouses and fulfilment centers	▪ Drive Unit: a mobile robot designed to carry heavy shelves of inventory on its back to prepare them for shipping or for storage.

25. Public services (e.g., road work and other shared public good)	▪ BrainOS: an autonomous floor care robot that can be used for both hard floor (e.g., retail, grocery) and soft floor care (e.g., cleaning carpeted areas within commercial office buildings).
26. Transportation (i.e., land, water, and/or sky) of goods, people, and other living entities, transport equipment, and delivery/courier/shipping services	▪ Waymo Driver: autonomous driving technology that operates vehicles without the need for human drivers.
27. Airports	▪ TaxiBot: a robot used for airplane taxiing without the need for engines to be running.
28. Art	▪ Shimon: a robotic marimba player that can be a member of any band or orchestra.

Stimulus Set B: Robot Examples Used as Stimuli in Studies 5-7

1. Health and human care and wellbeing (e.g., medical, surgical, fitness, lab diagnostics, elderly, disability, infant/child, and personal care)	▪ Xenex: an automated and portable germ-zapping robot that is used to disinfect entire hospital rooms using pulsed, full-spectrum UV rays that kill a range of infectious bacteria.
2. Social and companionship	▪ LOVOT: an advanced companion robot that can move autonomously, recognize individuals, and provide hugs.
3. Sex	▪ Willow (female robot): an AI driven, life-size robotic doll for human pleasure and companionship.<br>▪ Lucas (male robot): an AI driven, life-size robotic doll for human pleasure and companionship.
4. Animal care (e.g., walking pets)	▪ Rocky: a companion and playmate robot for pets who was designed to take care of any pet when their owner is not at home.
5. Security and surveillance	▪ Picard Surveillance Robot: a robot that can detect unauthorized activities, monitor areas in real-time, and provide actionable insights to ensure maximum security.
6. Policing and military	▪ Battlefield Extraction-Assist Robot (BEAR): a robot used to transport wounded soldiers to safety with no risk to human life.
7. Education, libraries, and knowledge/information management and gathering	▪ Tega: a robot designed for early education with the focus on various aspects of literacy (e.g., vocabulary, storytelling).
8. Research and exploration within science, technology, engineering, and mathematics (STEM) (e.g., ocean exploration, supercomputing, IT innovation, space discovery)	▪ Robonaut: a research and exploration robot that can be used for several purposes, from working on the International Space Station to exploring different planets.
9. Communication tools and channels	▪ BOCCO emo: a communication robot that allows interacting with family members through voice and text messages.

10. Leisure, recreation, and travel	▪ Challau: a tourism robot platform that allows people to virtually explore various tourist spots.
11. Culture/entertainment, gaming, toys, and other amusement	▪ KUKA Entertainment Passenger Robot: a robot developed for carrying passengers for robot-based amusement rides or simulators.
12. Workplace domain (i.e., to aid or replace human effort)	▪ Tengai: a recruiter and interview robot with the purpose to undertake job interviews and assessments.
13. Dangerous and/or risky work	▪ SHOAL: a robotic fish that can monitor and search for pollution in ports and various areas of the sea.
14. Inspection, repair, and/or improvement of products, engines, equipment, technology, and/or infrastructure (e.g., buildings, bridges, roads, power supplies, nuclear reactors, pipes, gas mains)	▪ Sensabot: a robot that can inspect various oil and gas field equipment.
15. Agriculture (e.g., harvesting, farms)	▪ Spot Robotic Shepherd: an agile mobile robot that can scan and inspect the landscape and safely herd livestock.
16. Household chores/tasks and domestic help/assistance (i.e., inside and outside of the home)	▪ Aido: an easy-to-use, family-friendly robot that can help with household chores, handle schedules, play with kids etc.
17. Industry	▪ Pick: a robotic system with the capability to accurately identify and grasp objects that can be used in various industrial settings.
18. Hospitality and food service (i.e., hotels, conventions, restaurants, bars, and other lodging, space, food and/or drink provider) and related customer service and support	▪ Flippy: a mechanical arm robot that can pull raw patties from a stack and place them on the grill, flip burgers, or fry 80 baskets of food an hour.
19. Banking/financial services and related customer service and support	▪ NAO Customer-facing Robot: a humanoid robot for financial institutions, typically used for customer interactions in a bank's reception area.
20. Retail and commerce and related customer service and support	▪ Mim: a robot that provides customer service by meeting and greeting customers in key areas and directing them to products, departments, and other facilities.
21. Construction	▪ AX-162: a robotic excavator that can perform a variety of tasks like digging foundations, trenching, and loading trucks.
22. Manufacturing	▪ Sawyer: a collaborative robot that can help out with manufacturing tasks and work alongside humans.
23. Mining	▪ Gemini-Scout Mine Rescue Robot: a robot that helps rescue trapped miners.

24. Warehouses and fulfilment centers	▪ Stretch: a robot created to move boxes in warehouses and distribution centers.
25. Public services (e.g., road work and other shared public good)	▪ Sanbot: an AI robot that offers various professional services for the public service (e.g., customs, police stations, tax bureaus, courthouses, libraries, etc.).
26. Transportation (i.e., land, water, and/or sky) of goods, people, and other living entities, transport equipment, and delivery/courier/shipping services	▪ R2: a fully autonomous, on-road vehicle designed to transport local goods.
27. Airports	▪ Spencer: a mobile robot created to aid passengers through airport terminals.
28. Art	▪ Ai-Da: the world's first ultra-realistic AI robot artist.

Note. [a] Although Alexa can change its physical surroundings by switching on and off or controlling smart home devices such as lightbulbs, it does not move and may therefore not be fully aligned with one of the elements of the IEEE robot definition[8] (i.e., "acting in the physical world in order to accomplish one or more tasks"). Nevertheless, the main reason why we included this device among the stimuli was because our research generally revolved around participants' perceptions, conceptualizations, and experiences of robots, and Alexa was spontaneously mentioned by participants as an example of a robot in 35 instances in Study 3 (raw analysis with participants' responses can be accessed in the file "Study 3 - Iterative Analysis File" that can be found in the folder "Study 3 -> Iterative Categorization" using the following link: https://osf.io/nejvm?view_only=79b6eeee42e24cb2a977927712bdcdd2).

Considering that participants saw Alexa as a robot, and that our research tackled psychological processes regarding robots from participants' perspective, we found it appropriate to use the image and description of Alexa as one of the stimuli. Based on other research, it seems common for people to use the word robot in reference to Alexa. For example, in a study by Liu and Yao[234] (p.4), participants said the following when talking to or referring to Alexa: "Why are you going to the movies you are a robot" or "Alexa is a robot." Moreover, in a study by Fortunati, Edwards, Edwards, Manganelli, and de Luca[235] (p.6) a participant made the following observation regarding Alexa: "males treat it more as a person than a robot and females use it more like a robot than a human." In that regard, our research is consistent with other studies when it comes to Alexa being perceived as a robot.

Importantly, given that we used two sets of stimuli (i.e., Stimulus Sets A and B from this table), the second one of which did not include Alexa, and that measurement invariance analyses (Supplementary Table 9) showed that the taxonomy we developed had equivalent factor structure, loadings, and intercepts for these two stimuli sets, evidence indicates that participants experience Alexa like other stimuli that depict robots. In other words, it does not seem that Alexa confounded the findings regarding psychological processes in response to robots.

Supplementary Table 8: Exploratory Structural Equation Models (ESEMs) of the Psychological Responses to Robots (PRR) Scale (Study 5)

№	Psychological Process	Item	Sample 1 (UK)			Sample 2 (US)		
			P	N	C	P	N	C
116	Empathy	This robot is empathetic.	0.801			0.756		
132	Self-improvement	This robot helps me to improve myself.	0.760			0.746		
88	Thoughtfulness	This robot is thoughtful.	0.758			0.769		
35	Attachment	I would feel attached to this robot.	0.742			0.756		
84	Enjoyment	I associate this robot with enjoyment.	0.740			0.789		
32	Happiness	This robot makes me feel happy.	0.682			0.729		
40	Engagement	I would like to engage with this robot.	0.674			0.588		
149	Robot rights	I think this robot should have rights.	0.672			0.654		
27	Perceiving the robot as human-like	This robot is like a human.	0.658			0.662		
96	Gratitude	This robot makes me feel grateful.	0.655			0.626		
107	Empowerment	I feel empowered by this robot.	0.644			0.638		
66	Care	This robot provides care.	0.587			0.604		
63	Communication	I would find it easy to communicate with this robot.	0.561			0.536		
125	Creativity	This robot is creative.	0.557			0.523		
17	Awareness	This robot has awareness.	0.546			0.496		
59	Threat	I feel threatened by this robot.		0.799			0.816	
113	Immorality	This robot is immoral.		0.756			0.656	
25	Dehumanization	I would feel dehumanized when interacting with this robot.		0.750			0.787	
114	Freedom restriction	This robot restricts or limits me.		0.746			0.724	
13	Anxiety	This robot makes me feel anxious.		0.733			0.785	
48	Damage reputation	This robot could damage my reputation.		0.723			0.709	
145	Embarrassment	I would feel embarrassed or ashamed if I had to interact with this robot.		0.719			0.695	
135	Confusion	This robot makes me feel confused.		0.715			0.713	
126	Privacy	This robot violates privacy (e.g., is too intrusive or invasive).		0.709			0.698	
76	Abnormal	This robot is abnormal.		0.694			0.658	
115	Societal issues	This robot can have negative social implications.		0.685			0.682	
72	Negative affect	I feel negative toward this robot.		0.680			0.760	

104	Humans lacking control	I would lack or lose control when using or interacting with this robot.		0.675			0.708	
91	Disgust	This robot is disgusting.		0.673			0.701	
52	Protection of self	I would want to protect myself when interacting with this robot.		0.653			0.687	
142	Robots contribute to human degeneration	This robot can contribute to human degeneration (e.g., make people become lazy, use less of their mental and physical capacity, etc.).		0.595			0.622	
7	Accuracy	This robot is accurate in what it does.			0.762			0.644
4	Performance	This robot can effectively achieve a certain result or a specified outcome.			0.701			0.606
54	Speed	This robot is fast at what it does.			0.681			0.660
2	Complexity	This robot can do complex tasks.			0.643			0.659
55	Level of advancement	This robot is advanced.			0.617			0.619
8	Financial costs	This robot reduces costs.			0.483			0.514
Factor								
P			-			-		
N			-0.239	-		-0.183	-	
C			0.386	-0.431	-	0.423	-0.352	-

Model Fit

Sample 1: $\chi^2(558) = 1918.764$, $p < .001$, SRMR = .028, CFI = .927, RMSEA = .047, 90% CI [.045, .049]

Sample 2: $\chi^2(558) = 1839.997$, $p < .001$, SRMR = .029, CFI = .927, RMSEA = .046, 90% CI [.043, .048]

Note. Labels P, N, and C refer to the factors that comprise Positive, Negative, and Competence-related psychological processes regarding robots. Values under each factor correspond to standardized factor loadings; only loadings ≥.32 are reported for clarity. The coefficients for factors P-N-C at the bottom of the table (above Model Fit) denote the standardized loadings of the factors on each other. All factors also yielded good to excellent Cronbach's α values (Sample 1—Positive: α = .927; Negative: α = .943; Competence: α = .818; Sample 2—Positive: α = .923; Negative: α = .943; Competence: α = .802).

Supplementary Table 9: Measurement Invariance Tests of the Psychological Responses to Robots (PRR) Scale for Country: UK vs. US; Robot Example: A vs. B; Gender: Female vs. Male; Age: Below Median vs. Median and Above; and Employment Status: Employed vs. Unemployed (Study 5).

Invariance Model	SRMR	ΔSRMR	CFI	ΔCFI	RMSEA	ΔRMSEA
Sample 1 (UK) vs. Sample 2 (US)						
Configural	.028	-	.927	-	.046	-
Metric	.033	.005	.926	.001	.045	.001
Scalar	.035	.002	.923	.003	.045	<.001
Robot Example A vs. B (Sample 1)						
Configural	.031	-	.924	-	.048	-
Metric	.038	.007	.925	.001	.046	.002
Scalar	.038	<.001	.924	.001	.046	<.001
Robot Example A vs. B (Sample 2)						
Configural	.032	-	.927	-	.046	-
Metric	.040	.008	.925	.002	.045	.001
Scalar	.040	<.001	.924	.001	.044	.001
Gender: Female vs. Male (Sample 1)						
Configural	.031	-	.926	-	.048	-
Metric	.041	.010	.924	.002	.047	.001
Scalar	.042	.001	.920	.004	.047	<.001
Gender: Female vs. Male (Sample 2)						
Configural	.032	-	.927	-	.046	-
Metric	.041	.009	.926	.001	.044	.002
Scalar	.041	<.001	.923	.003	.045	.001
Age: Below 48 (median) vs. 48 and above (Sample 1)						
Configural	.031	-	.925	-	.048	-
Metric	.039	.008	.924	.001	.046	.002
Scalar	.040	.001	.919	.005	.047	.001
Age: Below 45 (median) vs. 45 and above (Sample 2)						
Configural	.033	-	.921	-	.048	-
Metric	.041	.008	.919	.002	.047	.001
Scalar	.043	.002	.915	.004	.047	<.001
Employment Status: Employed vs. Unemployed (Sample 1)						
Configural	.031	-	.926	-	.048	-
Metric	.039	.008	.924	.002	.047	.001
Scalar	.039	<.001	.923	.001	.046	.001
Employment Status: Employed vs. Unemployed (Sample 2)						
Configural	.032	-	.925	-	.047	-
Metric	.039	.007	.924	.001	.045	.002
Scalar	.039	<.001	.923	.001	.045	<.001

Note. Sign Δ refers to the absolute value of a change in fit indices for an Invariance Model relative to the previous one (i.e., metric minus configural; scalar minus metric). For Robot Example, "A" indicates that the robot example for the domain to which participants from Study 5 were randomly allocated belonged to one of the two stimulus sets used in the present research, whereas "B" indicates that the robot example belonged to the other stimulus set (Supplementary Table 7). For Gender, very few participants identified themselves as "other" or did not disclose any information (Supplementary Table 1) and were therefore randomly classified as either "female" or "male" so they could be used in invariance testing. For Employment Status, the category "employed" includes participants who were self-employed or working for an employer. For Use of Robots at Work, we could not analyse measurement invariance because of the insufficient number of participants who used robots at work (Supplementary Table 1). However, for Study 6, in which sample sizes were larger (Supplementary Table 1), we tested measurement invariance for this variable and for additional participant characteristics assessed in that study (educational attainment, income, being liberal vs. conservative, ethnic identity, and relationship status). Measurement invariance was met in all cases (Supplementary Table 10).

Supplementary Table 10: Additional Measurement Invariance Tests of the Psychological Responses to Robots (PRR) Scale for Educational Attainment: Secondary or Below vs. Higher Education; Income: Below Median vs. Median and Above; Political Orientation: Liberal vs. Conservative; Ethnic Identity: White vs. Another Identity; In a Relationship: No vs. Yes; and Use of Robots at Work: No vs. Yes (Study 6).

Invariance Model	SRMR	ΔSRMR	CFI	ΔCFI	RMSEA	ΔRMSEA
Educational Attainment: Secondary or Below vs. Higher Education						
Configural	.026	-	.937	-	.045	-
Metric	.031	.005	.936	.001	.043	.002
Scalar	.033	.002	.933	.003	.044	.001
Income: Below $59k (Median) vs. $59k and Above						
Configural	.027	-	.936	-	.045	-
Metric	.038	.011	.931	.005	.045	<.001
Scalar	.038	<.001	.928	.003	.045	<.001
Political Orientation: Liberal vs. Conservative						
Configural	.027	-	.934	-	.047	-
Metric	.036	.009	.931	.003	.046	.001
Scalar	.037	.001	.929	.002	.046	<.001
Ethnic Identity: White vs. Another Identity						
Configural	.026	-	.936	-	.046	-
Metric	.031	.005	.935	.001	.044	.002
Scalar	.032	.001	.933	.002	.044	<.001
In a Relationship: No vs. Yes						
Configural	.026	-	.937	-	.045	-
Metric	.032	.006	.936	.001	.043	.002
Scalar	.033	.001	.934	.002	.043	<.001
Use of Robots at Work: No vs. Yes						
Configural	.028	-	.937	-	.046	-
Metric	.037	.009	.936	.001	.044	.002
Scalar	.038	.001	.934	.002	.044	<.001

Note. Sign Δ refers to the absolute value of a change in fit indices for an Invariance Model relative to the previous one (i.e., metric minus configural; scalar minus metric). For Educational Attainment, "secondary or below" comprises participants who had either no formal qualification or secondary education, whereas "higher education" comprises participants who had an undergraduate degree, graduate degree, doctoral degree, or professional qualification. For Political Orientation, "conservative" comprises participants who identified themselves as extremely conservative, conservative, or slightly conservative, whereas "liberal" comprises participants who identified themselves as extremely libera, liberal, or slightly liberal. For Ethnic Identity, "white" comprises participants who identified themselves as white (American or other), whereas "another Identity" comprises participants who identified themselves as black (African American or other), Asian or Asian American, Hispanic or Latino, or mixed. For In a Relationship, "no" comprises participants who were single, divorced, or widowed, whereas "yes" comprises participants who were in a relationship, married, or in civil partnership.

Supplementary Table 11: All Predictors (Bold Typeface), the Measures Used to Assess Them (Light Typeface), and Their Justification (Study 6)

	Predictor	Justification
		Individual Differences
1-4.	**Affiliation (Exclusion Concern) (FSMI_AEC), Affiliation (Independence) (FSMI_AI), Kincare (Family) (FSMI_KF), Status (FSMI_S)**: Fundamental Social Motives Inventory (FSMI[236]), 24 items, 1-7 scale (1=Strongly disagree, 7=Strongly agree).	Considering that people sometimes attribute human traits, emotions, or intentions to non-living entities such as robots[46,203], we measured several fundamental social motives because we thought these motives may also be important for how people judge robots. In particular, we selected FSMI_AEC and FSMI_AI because these social motives to some degree tap into isolation, dependency on others, and loneliness (versus independence and self-sufficiency), and loneliness has been identified as an important component of human relationships with robots[237–239]. Moreover, we selected FSMI_KF because this social motive captures how important people find caring for their family and spending time with them, and we speculated that individuals who are not caring in this regard may not show much affection toward robots either. Finally, we selected FSMI_S because we expected that people who value status and being ranked above others may react positively to robots, considering that robots are obedient and cannot challenge their status.
5-9.	**Agreeableness (BFI_A), Conscientiousness (BFI_C), Extraversion (BFI_E), Neuroticism (BFI_N), Openness to Experience (BFI_O)**: Short Big Five Inventory (BFI-S-15[240]), 15 items, 1-7 scale (1=Strongly disagree, 7=Strongly agree).	Considering that the Big Five are core personality traits, we found it important to examine their association with the PRR scale. Previous research also found that some of the Big Five traits (i.e., extraversion and openness) predicted positive responses to robots[215–217], thus indicating potential relevance of the Big Five to psychological processes regarding robots.

10-13.	**Anger (BPAQ_A), Hostility (BPAQ_H), Physical Aggression (BPAQ_PA), Verbal Aggression (BPAQ_VA)**: Buss-Perry Aggression Questionnaire (BPAQ[241]), 29 items, 1-5 scale (1=Extremely uncharacteristic of me, 5=Extremely characteristic of me).	Anger toward robots, and their verbal or physical harm and abuse have been identified among the negative psychological processes in both the present and previous research[72–74]. We therefore speculated that the dimensions of BPAQ that capture anger and hostility, as well as verbal and physical aggression, may predict people's psychological reactions to robots.
14.	**Animal Reminder Disgust (DSR_ARD)**: Animal Reminder Disgust Sub-Scale of the Disgust Scale-Revised (DS-R[242]), 8 items, True (1)/False (0) and Not (0)/Slightly (0.5)/Very (1) scales.	We measured DSR_ARD because it was found to predict negative psychological processes (i.e., feelings of eeriness[220]) regarding robots.
15.	**Anthropomorphism (IDAQ)**: Individual Differences in Anthropomorphism Questionnaire (IDAQ[46]), 15 items, 0-10 scale (0=Not at all, 10=Very much).	Considering that anthropomorphism has been a highly important construct in relation to various types of robots[203], and that it was associated with positive attitudes toward service robots[218], we expected it may play an important role as a predictor of psychological processes regarding robots.
16-17.	**Approach Temperament (ATQ_AP), Avoidance Temperament (ATQ_AV)**: Approach Avoidance Temperament Questionnaire (ATQ[97]), 12 items, 1-7 scale (1=Strongly disagree, 7=Strongly agree).	We measured ATQ_AP and ATQ_AV both because they are among the fundamental dimensions of personality[97,243], and because they have critical roles in people's creative and innovative performance[244], so we expected they may also play a role in how people react to technological innovations such as robots.
18-23.	**Autonomy Frustration (BPNSFS_AF), Autonomy Satisfaction (BPNSFS_AS), Competence Frustration (BPNSFS_CF), Competence Satisfaction (BPNSFS_CS), Relatedness Frustration (BPNSFS_RF), Relatedness Satisfaction (BPNSFS_RS)**: Basic Psychological Need Satisfaction and Frustration Scale (BPNSFS[245]), 24 items, 1-5 scale (1=Completely untrue, 5=Completely true).	We used the six basic psychological needs as predictors both because of their fundamental nature[245] and because the self-determination theory that underpins them has played an important role in various contexts associated with robots, including technology adoption[246].
24-27.	**Boredom Susceptibility (BSSS_BS), Disinhibition (BSSS_D), Experience Seeking (BSSS_ES), Thrill and Adventure Seeking (BSSS_TA)**: Brief Sensation Seeking Scale (BSSS[247]), 8 items, 1-5 scale (1=Strongly disagree, 5=Strongly agree).	We used BSSS because sensation seeking is typically associated with openness to new and exciting experiences and inventions[247], and it may therefore also shape how people see technological innovations such as robots. As predictors, we used all BSSS

		subscales because we did not have sound arguments regarding which of them may be the most important ones.
28-29.	**Cognitive Reappraisal (ERQ_CR), Expressive Suppression (ERQ_ES)**: Emotion Regulation Questionnaire (ERQ[195]), 10 items, 1-7 scale (1=Strongly disagree, 7=Strongly agree).	ERQ_CR is typically associated with positive affectivity and higher life satisfaction, and ERQ_ES with negative affectivity and lower life satisfaction[195]. We therefore expected that these positive or negative affective inclinations may generalize to various domains of life, including how people perceive robots.
30-35.	**Concern over Mistakes (FMPS_CM), Doubts about Actions (FMPS_DA), Organization (FMPS_O), Parental Criticism (FMPS_PC), Parental Expectations (FMPS_PE), Personal Standards (FMPS_PS):** Frost Multidimensional Perfectionism Scale (FMPS[192]), 35 items, 1-5 scale (1=Strongly disagree, 5=Strongly agree).	We used FMPS because several psychological processes that we identified are indicative of perfectionism (e.g., accuracy, performance, level of advancement), and we hence wanted to see if this construct plays a role in how people react to robots. As predictors, we used all FMPS subscales because we did not have sound arguments regarding which of them may be the most important ones.
36-45.	**Conformity-Interpersonal (PVQ5X_CI), Conformity-Rules (PVQ5X_CR), Face (PVQ5X_F), Hedonism (PVQ5X_H), Power-Dominance (PVQ5X_PD), Security-Personal (PVQ5X_SP), Security-Societal (PVQ5X_SS), Tradition (PVQ5X_T), Universalism-Concern (PVQ5X_UC), Universalism-Nature (PVQ5X_UN)**: PVQ5X Value Survey[196], 30 items, 1-6 scale (1=Not like me at all, 6=Very much like me).	Considering that PVQ5X measures basic individual values, we used it to understand which core values underpin psychological processes regarding robots. Rather than assessing all the basic values covered by PVQ5X, we focused only on those that we found relevant in relation to robots. In that regard, we captured different aspects of conformity (PVQ5X_C and PVQ5X_CR) because we estimated that more conformist individuals would be less likely to react positively to disruptive technologies such as robots. For a similar reason, we measured PVQ5X_T because we assumed more traditional individuals would have fewer positive views of robots due to their innovativeness. Moreover, we expected that PVQ5X_F may be linked to how robots are perceived, because people high (vs. low) in this trait are concerned with maintaining one's public image and control over how they present themselves, whereas robots could potentially intrude into their privacy and reveal personal secrets. We also assessed PVQ5X_PD because this value captures people's inclination to control and dominate others, and robots are machines that can be subjected to control and

		domination. In addition, we measured PVQ5X_H because this construct concerns valuing pleasure and sensuous gratification, and robots are machines that can be used for the purpose of pleasure by providing fun and acting as helpers or assistants. We found different aspects of security (PVQ5X_SP and PVQ5X_SS) important because these values capture people's needs to stay safe or live in a safe environment, and robots can be used both to make people safe (e.g., policing and military) and to decrease safety by compromising privacy. Finally, we found two different aspects of universalism (PVQ5X_UC and PVQ5X_UN) important because they are related to protecting others and protecting nature, and robots have implications for both constructs. For example, as already mentioned, they can be used to make people safe and protected but also to invade their privacy, and they can impact nature through the amount of energy they use and the pollution they may create.
46-49.	**Contra-Trait Antiegalitarianism (SDO_CTA), Contra-Trait Dominance (SDO_CTD), Pro-Trait Antiegalitarianism (SDO_PTA), Pro-Trait Dominance (SDO_PTD)**: Short Social Dominance Orientation Scale ($SDO_{7(s)}$[248]), 8 items, 1-7 scale (1=Strongly Oppose, 7=Strongly Favor).	We used $SDO_{7(s)}$ because social dominance orientation is linked to people being less open to new experiences[249] and may more generally indicate resistance to new technologies such as robots. As predictors, we used all $SDO_{7(s)}$ subscales because we did not have sound arguments regarding which of them may be the most important ones.
50-52.	**Current Focus (TFS_CF), Future Focus (TFS_FF), Past Focus (TFS_PF)**: Temporal Focus Scale (TFS[250]), 12 items, 1-7 scale (1=Never, 7=Always).	Considering that our research showed robots are associated with future-related psychological processes (e.g., future orientation, progress, novelty), we measured different aspects of temporal focus (TFS_CF, TFS_FF, TFS_PF) because we expected this construct may be relevant to people's responses to robots.
53-56.	**Empathic Concern (IRI_EC), Fantasy (IRI_F), Personal Distress (IRI_PD), Perspective Taking (IRI_PT)**: Interpersonal Reactivity Index (IRI[251]), 28 items, 0-4 scale (0=Does not describe me well, 4=Describes me very well).	Considering that people can feel empathy toward robots[21,22], we wanted to examine whether individual differences in this trait as measured by IRI predict people's psychological processes. We measured all four dimensions of this construct (IRI_EC, IRI_F,

		IRI_PD, IRI_PT) to get a more nuanced understanding regarding the role of empathy in responses to robots.
57.	**General Risk Propensity (GRP)**: General Risk Propensity Scale (GRiPS[191]), 11 items, 1-5 scale (1=Strongly disagree, 5=Strongly agree).	Robots are associated with numerous risks, such as potential negative consequences for human employment and privacy[252,253]. For that reason, we wanted to examine whether GRP would predict how people react to robots.
58.	**General Self-Efficacy (GSE)**: New General Self-Efficacy Scale (NGSE[254]), 8 items, 1-5 scale (1=Strongly disagree, 5=Strongly agree).	We tested GSE because people high (vs. low) in this trait have higher confidence in their skills and abilities[254], and may therefore be less scared of potential consequences of automation and robots taking their jobs. For this reason, GSE may be associated with how people view robots.
59.	**Honesty-Humility (HEXACO_HH)**: HEXACO–60[255], 10 items, 1-5 scale (1=Strongly disagree, 5=Strongly agree).	We primarily tested this individual difference because we were interested in examining the link between core personality traits and the PRR scale, and HEXACO_HH is one of the few such traits that are not captured by the Big Five inventory[240].
60.	**Intolerance of Uncertainty (IUS)**: Short Intolerance of Uncertainty Scale (IUS-12[256]), 12 items, 1-5 scale (1=Not at all characteristic of me, 5=Entirely characteristic of me).	Although various projections exist regarding how robots may impact the future[257,258], potential consequences of increased robot adoption are uncertain because it is not possible to precisely predict the scope and impact of this change. IUS may therefore shape how people react to robots.
61-63.	**Intrinsic Religiosity (DUREL_IR), Non-Organisational Religious Activity (DUREL_NORA), Organisational Religious Activity (DUREL_ORA)**: The Duke University Religion Index (DUREL[259]), 5 items, 1-5 scale (1=Definitely not true, 5=Definitely true of me), 1-6 scale (1=Rarely or never, 6=More than once a day), and 1-6 scale (1=Never, 6=More than once a week).	We used DUREL because previous research showed that religious beliefs are associated with negative psychological processes (i.e., feelings of eeriness[220]) regarding robots. As predictors, we used all DUREL subscales because we did not have sound arguments regarding which of them may be the most important ones.
64.	**Life Orientation (LOT)**: Life Orientation Test (LOT[260]), 10 items, 0-4 scale (0=Strongly disagree, 4=Strongly agree).	We used LOT because it captures optimism about the future[260], and we therefore expected that people high (vs. low) in this individual difference would be less pessimistic about the future impact of robots on human lives, which may shape their psychological processes concerning robots.

65.	**Life Satisfaction (LST)**: Life Satisfaction[261], 1 item, 0-10 scale (0=Completely dissatisfied, 10=Completely satisfied).	Life satisfaction is associated with how positive or negative people feel in many specific domains of their life (e.g., health, job, family[262]). This individual difference may therefore color their responses to various topics and situations, including robots.
66.	**Lifespan Self-Esteem (LSE)**: Lifespan Self-Esteem Scale (LSE[263]), 4 items, 1-5 scale (1=Really Sad, 5=Really Happy).	We tested LSE because people high (vs. low) in this trait feel good about themselves and the kind of person they are[263] and may therefore be more optimistic about their role in a future society marked by increased automation. For this reason, LSE may be associated with how people view robots.
67.	**Locus of Control (LCT)**: Locus of Control Scale[264], 8 items, 1-7 scale (1=Strongly disagree, 7=Strongly agree).	Because people high (vs. low) in LCT feel that they have more control over their lives and future[264], they may feel more in control over potential impact of automation on their lives and thus view robots and various other aspects of automation in a more positive light.
68-70.	**Machiavellianism (SD3_M), Narcissism (SD3_N), Psychopathy (SD3_P)**: Short Dark Triad (SD3[194]), 27 items, 1-5 scale (1=Disagree strongly, 5=Agree strongly).	SD3_M, SD3_N, and SD3_P are three socially aversive traits that tap into how manipulative and mean to others someone is to achieve their goals, and how highly they think of themselves compared to others[194]. Because these individual differences have large implications for how an individual responds to others and interacts with them, we also expected that they may be important for how people respond to and interact with robots.
71.	**Need for Cognition (NCS)**: Short Need for Cognition Scale (NCS-6[265]), 6 items, 1-5 scale (1=Extremely uncharacteristic of me, 5= Extremely characteristic of me).	In ref.[219], NCS was negatively related to negative attitudes toward robots as measured via the negative attitudes toward robots scale (NARS[266]), so we expected this trait may also be relevant to one or more dimensions assessed via the PRR scale in our research.
72-73.	**Other-Interest (SOII_OI), Self-Interest (SOII_SI)**: Self- and Other-Interest Inventory (SOII[267]), 18 items, 1-7 scale (1=Strongly disagree, 7=Strongly agree).	We expected that SOII_OI and SOII_SI may be relevant to how people perceive robots because individuals who are high (vs. low) in SOII_OI may be more focused on how robots will impact other individuals and their society when evaluating robots, whereas those who are high (vs. low) in SOII_SI may be more focused on the consequences that robots could have for them individually.

74.	**Self-Monitoring (SM)**: Self-Monitoring Scale[268], 18 items, True (1)/False (0).	Because people high (vs. low) in SM monitor their behavior in social situations to leave a desired impression[268], they may feel less positive toward robots if they generally assume that robots could collect the data that would expose them. Alternatively, they may feel more positive toward robots if they generally assume that they do not have to pretend and monitor their behavior in front of robots.
75-76.	**Social Comparison Ability (INCOM_A), Opinions (INCOM_O)**: Iowa-Netherlands Comparison Orientation Measure (INCOM[269]), 11 items, 1-5 scale (1=Strongly disagree, 5=Strongly agree).	INCOM_A and INCOM_O are typically associated with negative affectivity and lower wellbeing[269]. We therefore speculated that the negative affective inclinations linked to social comparisons may generalize to various domains of life, including how people perceive robots.
77.	**Trait Anxiety (STAIT_TA)**: Short State-Trait Anxiety Inventory (STAIT-5[270]), 5 items, 1-4 scale (1=Not at all, 4=Very much so).	Considering that potential consequences of increased robot adoption are uncertain because it is not possible to precisely predict them, and that increased uncertainty is typically experienced as anxiety[271], we speculated that individuals high (vs. low) in STAIT_TA may be more sensitive to the uncertainties associated with robots, which may shape how they respond to robots.
78-79.	**Trait Negative Affect (PANAS_TNA), Trait Positive Affect (PANAS_TPA)**: Positive and Negative Affect Schedule (PANAS[193]), 20 items, 1-5 scale (1=Very slightly or not at all, 5=Extremely).	People who are high in trait negative affect are generally more likely to experience negative state affect across different situations[193,230]. Such people may therefore be inclined to experience negative psychological processes regarding robots simply because they generally feel more negative.
	Covariates	
80.	**Age (AGE)**: in years.	Previous research showed that age plays a role in how people perceive robots[272,273], and we therefore expected this variable may be a potential confound of the relationship between the individual differences tested and the PRR scale.

81. **Annual Income After Taxes (INCM)**: assessed in $1,000s using a slider from 0 (=$0) to 150 (=$150,000 or more).	Various studies have shown that low (vs. high) income individuals tend to find automation more threatening[274]. Therefore, annual income may confound the link between individual differences and how people respond to robots.
82. **Political Orientation (LBCN)**: Graham et al.[275], 1 item, 1-7 scale (1=Extremely liberal, 7=Extremely conservative).	We measured this variable because conservatism is typically associated with negative attitudes toward technology[276], and we therefore expected political orientation may confound the link between individual difference predictors and the PRR scale.
83. **Familiarity with the Robot (FAM)**: 1 item "How familiar are you with the robot from the example you were given?", 1-5 scale (1=Very slightly or not at all, 5=Extremely).	Familiarity with a stimulus is typically associated with positive attitudes toward the stimulus[277,278], which indicates that people familiar with robots may see them as more favorable. This variable may therefore potentially confound the link between individual differences and psychological reactions to robots.
84. **Frequency of Interaction with the Robot (FI)**: 1 item "How frequently do you usually interact with the type of robot from the example you were given?", 1-5 scale (1=Never, 5=Very often).	According to the mere exposure effect, repeated exposure to a stimulus makes people's attitudes toward that stimulus more favorable[279], which indicates that people who frequently interact with robots may see them as more favorable. This variable may therefore be a potential confound.
85. **Descriptive Norms (DN)**: 1 item "In my society, people frequently interact with the type of robot from the example I was given.", 1-7 scale (1=Strongly disagree, 7=Strongly agree).	DN are a highly important construct in psychology when it comes to understanding intentions and behavior[280] and may therefore shape how people respond to robots, thus confounding the link between various individual differences and the PRR scale.
86. **Injunctive Norms (IN)**: 1 item "In my society, interacting with the type of robot from the example I was given is widely accepted.", 1-7 scale (1=Strongly disagree, 7=Strongly agree).	IN are a highly important construct in psychology when it comes to understanding intentions and behavior[280] (e.g., Miller & Prentice, 2016) and may therefore shape how people respond to robots, thus confounding the link between various individual differences and the PRR scale.

Note. Variables 83-86 were created by the authors of this paper.

Supplementary Table 12: The Most Predictive Machine Learning Models and Their 30 Most Important Predictors (Study 6)

CR Forest		Linear LS		Ridge		Lasso		E Net		R Forest	
Predictor	VarI	Predictor	VarI	Predictor	VarI	Predictor	VarI	Predictor	VarI	Predictor	VarI
DV = Positive Dimension											
FI (COV.)	100.00	IN (COV.)	100.00	IN (COV.)	100.00	IN (COV.)	100.00	IN (COV.)	100.00	FI (COV.)	100.00
IN (COV.)	38.421	IDAQ	85.114	FI (COV.)	89.897	FI (COV.)	96.435	FI (COV.)	93.389	SOII_SI	38.183
IDAQ	31.443	FI (COV.)	70.450	IDAQ	77.952	IDAQ	73.214	IDAQ	75.174	FAM (COV.)	29.482
SOII_SI	23.046	FAM (COV.)	44.153	FAM (COV.)	63.315	FAM (COV.)	54.068	FAM (COV.)	56.415	IDAQ	16.770
FAM (COV.)	23.037	FSMI_AI	41.067	GRP	43.844	GRP	41.576	GRP	42.619	IN (COV.)	16.536
DN (COV.)	16.257	GRP	38.352	FSMI_AI	40.661	ATQ_AV	37.874	ATQ_AV	38.753	SD3_N	14.492
SOII_OI	13.680	FMPS_PS	37.754	BSSS_TA	37.849	FMPS_PS	35.724	FMPS_PS	38.130	SOII_OI	13.365
SD3_N	8.625	SDO_PTA	36.110	ATQ_AV	36.947	FSMI_AI	35.632	FSMI_AI	37.569	PVQ5X_PD	9.203
ATQ_AP	8.374	FMPS_PE	33.556	SDO_PTA	36.813	IUS	34.065	IUS	34.381	DN (COV.)	7.842
LSE	5.781	ATQ_AV	33.294	FMPS_PS	34.748	SDO_PTA	31.389	PVQ5X_UC	34.239	FSMI_S	5.877
GRP	5.623	PVQ5X_UC	32.758	SOII_SI	34.461	PVQ5X_UC	31.188	SDO_PTA	33.789	INCM (COV.)	5.389
PANAS_TPA	3.734	IUS	31.242	IUS	34.355	BPAQ_H	30.874	BSSS_TA	32.807	ATQ_AP	5.289
SDO_CTA	3.449	HEXACO_HH	29.216	IRI_PD	33.453	BSSS_TA	29.641	BPAQ_H	31.390	GRP	5.131
LCT	3.389	BSSS_TA	29.114	SOII_OI	33.445	FMPS_PE	29.381	FMPS_PE	30.910	LCT	4.084
PVQ5X_PD	3.102	BPAQ_H	27.934	PVQ5X_UC	33.352	SOII_SI	28.275	SOII_SI	30.268	PANAS_TPA	3.724
BSSS_TA	3.037	PVQ5X_CI	27.877	FMPS_PE	32.594	PVQ5X_CI	27.878	SOII_OI	29.814	AGE (COV.)	3.176
FSMI_S	2.870	PANAS_TPA	27.174	HEXACO_HH	31.442	IRI_PD	27.611	IRI_PD	29.494	FMPS_PE	2.797
PVQ5X_F	2.757	IRI_PD	26.760	PANAS_TPA	30.915	SOII_OI	27.440	PANAS_TPA	28.350	ERQ_ES	2.722
BSSS_D	2.749	ATQ_AP	26.035	BPAQ_H	30.673	PANAS_TPA	26.744	PVQ5X_CI	27.632	HEXACO_HH	2.664
FMPS_PE	2.544	SDO_CTA	25.203	DN (COV.)	29.697	HEXACO_HH	26.261	HEXACO_HH	27.582	ERQ_CR	2.471
HEXACO_HH	2.319	SOII_OI	24.466	SDO_CTA	29.649	SDO_CTA	23.782	SDO_CTA	25.815	FSMI_AI	2.231
LST	2.295	BPNSFS_CS	24.266	PVQ5X_CI	28.983	ATQ_AP	22.226	ATQ_AP	24.423	FMPS_PS	2.017
PVQ5X_T	2.285	PANAS_TNA	23.659	LSE	27.301	BFI_A	21.675	BFI_A	22.907	GSE	2.010
ERQ_CR	2.258	BPNSFS_AS	22.278	ATQ_AP	26.276	LSE	18.929	BPNSFS_CS	22.159	LSE	1.976
PVQ5X_CI	2.029	SOII_SI	22.154	BFI_A	25.816	ERQ_ES	18.861	LSE	21.573	TFS_FF	1.933
INCM (COV.)	1.955	AGE (COV.)	22.060	BPNSFS_CS	25.307	BPNSFS_CS	18.008	BPNSFS_AS	20.247	IRI_PT	1.907
DUREL_IR	1.632	FSMI_AEC	21.785	SD3_N	25.266	BPNSFS_AS	17.804	ERQ_ES	19.978	INCOM_A	1.873
PVQ5X_UN	1.579	LSE	21.548	BSSS_D	24.692	PANAS_TNA	16.775	PANAS_TNA	19.749	FSMI_AEC	1.853
LBCN (COV.)	1.539	ERQ_ES	20.611	ERQ_ES	23.258	SD3_N	15.641	FSMI_AEC	18.608	IUS	1.835
BFI_A	1.494	FMPS_DA	19.591	PVQ5X_PD	22.721	FSMI_AEC	15.160	SD3_N	18.321	SDO_CTA	1.835
DV = Negative Dimension											
IN (COV.)	100.00	IN (COV.)	100.00	IN (COV.)	100.00	IN (COV.)	100.00	IN (COV.)	100.00	IN (COV.)	100.00
PANAS_TNA	13.379	IDAQ	27.098	PANAS_TNA	30.933	PANAS_TNA	27.340	PANAS_TNA	29.110	PANAS_TNA	45.863

SD3_P	11.037	PANAS_TNA	26.864	IDAQ	26.598	IDAQ	22.808	IDAQ	24.551	SD3_P	39.806
BPNSFS_RF	9.400	DN (COV.)	23.261	SD3_P	24.596	DN (COV.)	21.987	SD3_P	22.775	BPNSFS_RF	27.025
SDO_PTD	6.032	BSSS_BS	20.992	BSSS_BS	23.018	SD3_P	19.943	DN (COV.)	21.180	SDO_PTD	23.696
SDO_PTA	4.504	ERQ_ES	19.463	ERQ_ES	20.016	BSSS_BS	19.206	BSSS_BS	21.071	IDAQ	22.650
FMPS_DA	4.069	SD3_P	18.290	SOII_SI	18.183	SDO_PTD	17.099	ERQ_ES	17.520	BPAQ_H	19.591
BSSS_BS	3.473	INCOM_O	17.131	DN (COV.)	17.875	ERQ_ES	14.576	SDO_PTD	16.690	INCM (COV.)	18.393
IRI_PD	3.430	SOII_OI	16.565	SDO_PTD	17.090	DSR_ARD	13.335	SOII_SI	16.309	FSMI_AI	15.668
IDAQ	3.099	DSR_ARD	15.765	INCOM_O	17.003	IRI_F	12.007	SOII_OI	14.458	FMPS_CM	14.527
DN (COV.)	3.066	SOII_SI	14.973	SOII_OI	16.881	IRI_PD	11.667	INCOM_O	14.301	LOT	12.981
INCM (COV.)	2.675	SDO_PTD	14.350	DSR_ARD	16.290	BPNSFS_RF	11.476	DSR_ARD	14.170	FSMI_KF	12.899
FMPS_CM	2.317	IRI_F	13.170	IRI_F	14.540	LSE	8.643	BPNSFS_RF	13.101	FMPS_DA	12.772
SDO_CTD	1.980	PVQ5X_CI	12.713	BPNSFS_RF	14.116	INCOM_O	8.338	IRI_F	13.072	IUS	11.636
FMPS_PC	1.974	INCOM_A	12.470	LSE	14.019	BPAQ_VA	8.007	LSE	11.497	BPNSFS_CF	10.954
BSSS_TA	1.891	BPAQ_VA	12.114	BPAQ_VA	13.587	PVQ5X_T	7.277	BPAQ_VA	11.389	HEXACO_HH	10.856
ERQ_ES	1.692	LOT	11.618	PVQ5X_T	12.784	BFI_O	7.168	INCOM_A	11.051	BPAQ_A	10.533
FSMI_KF	1.691	LSE	11.074	INCOM_A	12.746	FSMI_AI	7.067	IRI_PD	10.888	SDO_PTA	10.513
BPAQ_H	1.652	BPNSFS_RF	10.313	BPAQ_H	12.480	SOII_SI	6.803	PVQ5X_T	10.083	ERQ_ES	9.968
BPNSFS_AF	1.642	PVQ5X_H	10.147	DUREL_ORA	11.399	SOII_OI	6.732	PVQ5X_CI	9.910	FMPS_PC	9.758
SDO_CTA	1.628	DUREL_ORA	10.115	BFI_N	11.303	SDO_CTD	6.519	BPAQ_H	9.827	IRI_PD	9.568
BFI_N	1.570	DUREL_NORA	10.062	LOT	10.995	DUREL_IR	6.290	BFI_N	9.407	SD3_N	9.359
FSMI_AI	1.306	BPAQ_A	10.038	IRI_PD	10.934	FMPS_DA	6.108	TFS_PF	8.984	IRI_F	9.355
BPAQ_A	1.259	BPAQ_H	9.778	PVQ5X_CI	10.847	LBCN (COV.)	5.978	LOT	8.948	DSR_ARD	9.158
DUREL_IR	1.229	PVQ5X_SS	9.621	TFS_PF	10.583	TFS_PF	5.968	DUREL_ORA	8.755	ATQ_AV	8.801
BPNSFS_CF	1.176	PVQ5X_T	9.485	PVQ5X_SS	10.124	INCOM_A	5.935	BFI_O	8.685	ERQ_CR	8.566
PVQ5X_T	1.170	TFS_PF	9.433	BFI_O	9.889	DUREL_ORA	5.594	FSMI_AI	8.062	SOII_OI	8.531
IRI_F	1.138	IRI_PD	8.963	DUREL_IR	9.765	PVQ5X_CI	5.011	DUREL_IR	7.968	AGE (COV.)	8.412
DSR_ARD	1.132	BPNSFS_RS	8.955	IRI_PT	9.667	BFI_N	4.660	PVQ5X_SS	7.923	PVQ5X_T	8.193
BFI_A	1.099	LBCN (COV.)	8.948	ATQ_AP	9.425	BSSS_ES	4.343	IRI_PT	7.743	GRP	8.016
DV = Competence Dimension											
IN (COV.)	100.00	IN (COV.)	100.00	IN (COV.)	100.00	IN (COV.)	100.00	IN (COV.)	100.00	IN (COV.)	100.00
ATQ_AP	36.328	FAM (COV.)	32.451	FAM (COV.)	36.861	ATQ_AP	37.844	ATQ_AP	37.665	SOII_SI	53.329
SOII_SI	7.880	ATQ_AP	31.761	ATQ_AP	36.760	FAM (COV.)	31.205	FAM (COV.)	31.445	ATQ_AP	50.821
SDO_CTA	7.855	AGE (COV.)	28.124	SDO_CTA	28.471	PVQ5X_SS	28.439	PVQ5X_SS	28.625	SOII_OI	24.825
ERQ_CR	5.943	PVQ5X_SS	27.487	PVQ5X_SS	28.309	SDO_CTA	26.982	SDO_CTA	27.316	ERQ_CR	15.694
FAM (COV.)	5.613	STAIT_TA	25.356	ERQ_CR	26.863	SOII_SI	22.374	SOII_SI	22.404	GSE	14.143
SOII_OI	5.502	ERQ_CR	25.049	IUS	24.293	ERQ_CR	21.056	ERQ_CR	21.654	LCT	13.344
PANAS_TPA	5.044	INCOM_O	23.694	SOII_SI	23.570	IUS	18.641	IUS	18.850	PANAS_TPA	13.010
LCT	4.364	TFS_PF	22.816	TFS_PF	23.266	STAIT_TA	18.263	TFS_PF	17.931	TFS_FF	12.023
PVQ5X_SS	3.707	SDO_CTA	22.547	STAIT_TA	23.197	TFS_PF	17.627	STAIT_TA	17.892	SDO_CTA	10.072
FSMI_KF	3.694	BPNSFS_AF	21.013	INCOM_O	22.904	INCOM_O	17.541	INCOM_O	17.843	INCM (COV.)	9.909
SD3_N	3.087	FI (COV.)	20.769	AGE (COV.)	21.899	AGE (COV.)	16.839	AGE (COV.)	16.636	FSMI_KF	9.276

PVQ5X_CI	2.930	IUS	19.738	LCT	18.535	BFI_N	16.743	BFI_N	16.310	AGE (COV.)	8.901
INCM (COV.)	2.925	SOII_SI	19.003	BPNSFS_AF	18.327	PVQ5X_CI	14.033	PVQ5X_CI	14.277	IDAQ	8.866
IRI_F	2.860	BFI_N	18.134	BFI_N	17.607	BFI_C	12.953	BFI_C	13.089	IRI_F	8.573
BPNSFS_RS	2.712	BFI_E	17.830	BFI_E	17.309	BPNSFS_AF	12.310	LCT	12.855	PVQ5X_UN	7.754
PVQ5X_UC	2.645	FSMI_AEC	16.456	BPNSFS_RS	16.233	BPNSFS_RS	12.258	BPNSFS_RS	12.631	BPNSFS_RS	7.668
TFS_FF	2.536	INCOM_A	15.835	SDO_PTA	15.761	LCT	12.214	BPNSFS_AF	12.455	SD3_N	7.520
PVQ5X_UN	2.206	FMPS_PC	15.160	FMPS_PC	15.633	SDO_PTA	12.136	SDO_PTA	12.344	PVQ5X_SS	7.245
PVQ5X_T	2.161	HEXACO_HH	14.810	HEXACO_HH	14.837	BSSS_TA	11.085	BFI_E	11.318	NCS	6.945
LSE	2.146	DUREL_IR	14.297	BSSS_TA	14.728	BFI_E	11.028	BSSS_TA	10.939	DN (COV.)	6.322
DN (COV.)	2.017	BSSS_TA	13.414	BFI_C	14.633	FSMI_AEC	10.706	FSMI_AEC	10.608	FSMI_AI	6.279
BFI_C	1.890	PVQ5X_CI	13.176	FSMI_AEC	14.386	FMPS_PC	9.964	FMPS_PC	10.245	PVQ5X_CI	6.204
PVQ5X_H	1.879	BFI_C	12.954	INCM (COV.)	14.176	FSMI_KF	9.648	FSMI_KF	9.985	IUS	6.198
PVQ5X_F	1.804	SDO_PTA	12.561	PVQ5X_CI	13.801	INCM (COV.)	9.124	INCM (COV.)	9.306	PVQ5X_H	6.067
FMPS_O	1.766	PVQ5X_PD	11.876	PVQ5X_PD	13.524	LBCN (COV.)	8.817	LBCN (COV.)	9.025	PVQ5X_PD	5.868
TFS_PF	1.715	LCT	11.636	DN (COV.)	12.758	BPAQ_PA	8.729	BPAQ_PA	8.590	BFI_O	5.835
HEXACO_HH	1.620	INCM (COV.)	11.619	FSMI_KF	12.749	BPNSFS_RF	8.547	BPNSFS_RF	8.561	IRI_PT	5.739
PVQ5X_PD	1.612	BSSS_BS	11.552	LBCN (COV.)	12.581	HEXACO_HH	7.378	HEXACO_HH	7.623	INCOM_A	5.711
INCOM_O	1.582	FMPS_PE	11.501	BPNSFS_AS	12.322	FI (COV.)	6.990	PVQ5X_PD	6.506	HEXACO_HH	5.702

Note. CR Forest = Conditional Random Forest; Linear LS = Linear Last Squares; E Net = Elastic Net; R Forest = Random Forest; DV = dependent variable. VarI indicates the importance of a variable computed using the VarImp function in R[163,164]. For clarification of predictor name abbreviations, see Supplementary Table 11. The cells that are highlighted in grey indicate variables identified as the most robust predictors because these were among the 30 most important predictors across all six models, while remaining statistically significant in the Linear LS models (Supplementary Tables 13-15) after applying the FDR correction[170] (see pp.37-42). Considering that covariates were used as predictors in all models but were not the main point of interest, they are labelled with (COV.) to be easily distinguished from the key individual difference predictors.

Supplementary Table 13: Coefficients for the Liner Least Squares Model Computed for the Positive Dimension (Study 6)

Variable	b	SE b	95% CI	t	p
(Intercept)	4.175	0.019	4.137 – 4.213	215.209	<.001
PANAS_TPA	0.074	0.031	0.013 – 0.135	2.370	.018†
PANAS_TNA	-0.063	0.031	-0.123 – -0.003	-2.066	.039†
LST	-0.022	0.033	-0.087 – 0.044	-0.643	.521
BFI_N	-0.025	0.036	-0.096 – 0.046	-0.692	.489
BFI_E	-0.024	0.025	-0.073 – 0.024	-0.975	.329
BFI_C	0.050	0.031	-0.010 – 0.110	1.624	.105
BFI_A	0.047	0.031	-0.013 – 0.107	1.529	.127
BFI_O	-0.011	0.027	-0.064 – 0.041	-0.426	.670
ATQ_AP	0.074	0.033	0.010 – 0.138	2.271	.023†
ATQ_AV	-0.112	0.039	-0.187 – -0.036	-2.899	.004
BSSS_ES	-0.023	0.027	-0.075 – 0.030	-0.842	.400
BSSS_BS	-0.023	0.027	-0.076 – 0.030	-0.863	.388
BSSS_TA	0.085	0.034	0.019 – 0.151	2.537	.011†
BSSS_D	0.050	0.036	-0.020 – 0.121	1.403	.161
BPNSFS_AS	0.065	0.033	-0.001 – 0.130	1.946	.052
BPNSFS_AF	-0.036	0.031	-0.098 – 0.025	-1.158	.247
BPNSFS_RS	-0.054	0.031	-0.115 – 0.008	-1.707	.088
BPNSFS_RF	0.001	0.037	-0.072 – 0.073	0.019	.985
BPNSFS_CS	-0.081	0.038	-0.156 – -0.006	-2.118	.034†
BPNSFS_CF	-0.047	0.040	-0.125 – 0.032	-1.167	.243
PVQ5X_SS	0.005	0.032	-0.058 – 0.068	0.145	.884
PVQ5X_H	-0.006	0.029	-0.063 – 0.051	-0.215	.830
PVQ5X_CI	0.070	0.029	0.013 – 0.126	2.430	.015†
PVQ5X_UC	-0.095	0.033	-0.161 – -0.030	-2.853	.004
PVQ5X_PD	0.053	0.035	-0.016 – 0.121	1.514	.130
PVQ5X_UN	-0.017	0.028	-0.072 – 0.037	-0.629	.530
PVQ5X_F	-0.026	0.031	-0.087 – 0.036	-0.817	.414
PVQ5X_SP	0.035	0.032	-0.028 – 0.098	1.097	.273
PVQ5X_CR	0.028	0.030	-0.030 – 0.086	0.942	.346
PVQ5X_T	0.033	0.031	-0.027 – 0.094	1.076	.282
NCS	0.011	0.026	-0.040 – 0.061	0.418	.676
FSMI_AEC	-0.059	0.031	-0.121 – 0.002	-1.903	.057
FSMI_AI	-0.092	0.026	-0.143 – -0.042	-3.571	<.001
FSMI_S	-0.022	0.039	-0.099 – 0.054	-0.572	.568
FSMI_KF	0.011	0.027	-0.042 – 0.064	0.397	.692
IUS	0.089	0.033	0.025 – 0.153	2.721	.007
STAIT_TA	0.056	0.035	-0.012 – 0.125	1.612	.107
IRI_F	-0.001	0.025	-0.050 – 0.048	-0.041	.967
IRI_EC	0.036	0.032	-0.026 – 0.098	1.130	.259
IRI_PT	0.035	0.028	-0.019 – 0.090	1.275	.202
IRI_PD	0.071	0.031	0.011 – 0.131	2.334	.020†
SDO_PTD	0.038	0.030	-0.021 – 0.096	1.260	.208

SDO_CTD	-0.004	0.026	-0.055 – 0.047	-0.165	.869
SDO_PTA	-0.090	0.029	-0.146 – -0.034	-3.142	.002
SDO_CTA	0.067	0.030	0.007 – 0.126	2.199	.028†
SD3_M	0.025	0.034	-0.042 – 0.092	0.736	.462
SD3_N	0.051	0.036	-0.020 – 0.122	1.411	.158
SD3_P	-0.041	0.039	-0.117 – 0.035	-1.059	.290
GRP	0.110	0.033	0.045 – 0.175	3.336	.001
BPAQ_PA	-0.006	0.036	-0.077 – 0.065	-0.163	.871
BPAQ_VA	-0.039	0.032	-0.101 – 0.024	-1.211	.226
BPAQ_A	0.051	0.038	-0.023 – 0.125	1.350	.177
BPAQ_H	0.095	0.039	0.019 – 0.172	2.435	.015†
IDAQ	0.178	0.024	0.131 – 0.225	7.381	<.001
LOT	-0.037	0.032	-0.100 – 0.026	-1.138	.255
DSR_ARD	0.020	0.023	-0.025 – 0.064	0.866	.387
FMPS_CM	-0.011	0.038	-0.086 – 0.064	-0.298	.766
FMPS_PS	-0.110	0.034	-0.176 – -0.045	-3.285	.001
FMPS_PE	0.088	0.030	0.029 – 0.146	2.922	.004
FMPS_PC	-0.004	0.030	-0.063 – 0.056	-0.119	.905
FMPS_DA	0.060	0.035	-0.009 – 0.128	1.714	.087
FMPS_O	-0.016	0.026	-0.068 – 0.036	-0.610	.542
ERQ_CR	0.040	0.026	-0.010 – 0.091	1.566	.117
ERQ_ES	0.046	0.025	-0.004 – 0.096	1.802	.072
SM	0.026	0.024	-0.021 – 0.072	1.092	.275
HEXACO_HH	-0.079	0.031	-0.139 – -0.018	-2.546	.011†
TFS_PF	0.014	0.026	-0.037 – 0.065	0.536	.592
TFS_CF	0.010	0.026	-0.041 – 0.062	0.399	.690
TFS_FF	-0.040	0.030	-0.099 – 0.019	-1.339	.181
SOII_SI	0.083	0.043	-0.001 – 0.167	1.935	.053
SOII_OI	0.073	0.034	0.006 – 0.140	2.135	.033†
DUREL_ORA	-0.004	0.031	-0.065 – 0.057	-0.141	.888
DUREL_NORA	-0.019	0.030	-0.078 – 0.040	-0.638	.524
DUREL_IR	0.032	0.032	-0.031 – 0.094	1.000	.317
INCOM_A	-0.027	0.031	-0.088 – 0.035	-0.844	.399
INCOM_O	0.018	0.028	-0.038 – 0.074	0.638	.523
LSE	0.067	0.035	-0.003 – 0.136	1.883	.060
GSE	0.007	0.036	-0.064 – 0.079	0.203	.839
LCT	-0.003	0.029	-0.061 – 0.054	-0.116	.908
AGE (COVARIATE)	0.056	0.029	-0.001 – 0.112	1.927	.054
INCM (COVARIATE)	0.029	0.026	-0.021 – 0.079	1.138	.255
LBCN (COVARIATE)	-0.019	0.025	-0.067 – 0.030	-0.754	.451
FAM (COVARIATE)	0.117	0.030	0.057 – 0.176	3.838	<.001
FI (COVARIATE)	0.211	0.034	0.143 – 0.278	6.113	<.001
DN (COVARIATE)	0.011	0.030	-0.048 – 0.070	0.364	.716
IN (COVARIATE)	0.227	0.026	0.176 – 0.279	8.668	<.001

Note. Model $R^2 = .576$. The model had 2097 residual degrees of freedom. For clarification of predictor name abbreviations, see Supplementary Table 11. We used t-tests (two-sided) to assess the significance of the coefficients, and the significance criterion was $p < .05$. The table contains raw p-values: symbol † indicates coefficients that were initially significant but stopped being significant after the FDR correction[170] for multiple comparisons was applied (see pp.37-38). The cells that are highlighted in grey indicate individual differences identified as the most robust predictors because these were among the 30 most

important predictors across all six machine learning models tested in Study 6 (Supplementary Table 12) while remaining statistically significant in the linear least squares model presented in this table.

Supplementary Table 14: Coefficients for the Liner Least Squares Model Computed for the Negative Dimension (Study 6)

Variable	b	SE b	95% CI	t	p
(Intercept)	2.617	0.021	2.577 – 2.658	125.527	<.001
PANAS_TPA	-0.012	0.034	-0.078 – 0.053	-0.369	.712
PANAS_TNA	0.151	0.033	0.087 – 0.216	4.591	<.001
LST	-0.020	0.036	-0.090 – 0.051	-0.549	.583
BFI_N	0.056	0.039	-0.020 – 0.132	1.442	.149
BFI_E	0.017	0.027	-0.035 – 0.069	0.648	.517
BFI_C	-0.007	0.033	-0.072 – 0.058	-0.214	.831
BFI_A	-0.005	0.033	-0.069 – 0.060	-0.142	.887
BFI_O	-0.044	0.029	-0.100 – 0.013	-1.520	.129
ATQ_AP	-0.045	0.035	-0.114 – 0.024	-1.279	.201
ATQ_AV	-0.010	0.041	-0.091 – 0.071	-0.236	.813
BSSS_ES	-0.029	0.029	-0.085 – 0.027	-1.009	.313
BSSS_BS	0.104	0.029	0.047 – 0.161	3.589	<.001
BSSS_TA	-0.000	0.036	-0.071 – 0.071	-0.007	.995
BSSS_D	-0.023	0.039	-0.099 – 0.052	-0.603	.547
BPNSFS_AS	0.038	0.036	-0.032 – 0.108	1.061	.289
BPNSFS_AF	0.024	0.034	-0.042 – 0.090	0.726	.468
BPNSFS_RS	0.052	0.034	-0.014 – 0.118	1.535	.125
BPNSFS_RF	0.070	0.040	-0.008 – 0.148	1.767	.077
BPNSFS_CS	0.007	0.041	-0.073 – 0.087	0.172	.864
BPNSFS_CF	-0.000	0.043	-0.085 – 0.084	-0.011	.992
PVQ5X_SS	-0.057	0.034	-0.124 – 0.011	-1.648	.099
PVQ5X_H	0.054	0.031	-0.007 – 0.115	1.738	.082
PVQ5X_CI	-0.067	0.031	-0.127 – -0.007	-2.176	.030†
PVQ5X_UC	-0.006	0.036	-0.076 – 0.064	-0.168	.866
PVQ5X_PD	-0.024	0.037	-0.097 – 0.050	-0.633	.527
PVQ5X_UN	0.014	0.030	-0.044 – 0.073	0.481	.631
PVQ5X_F	-0.005	0.034	-0.071 – 0.061	-0.148	.883
PVQ5X_SP	-0.005	0.034	-0.073 – 0.062	-0.156	.876
PVQ5X_CR	0.020	0.032	-0.042 – 0.083	0.643	.520
PVQ5X_T	0.054	0.033	-0.011 – 0.120	1.625	.104
NCS	-0.008	0.028	-0.062 – 0.047	-0.281	.779
FSMI_AEC	0.015	0.034	-0.051 – 0.081	0.441	.659
FSMI_AI	-0.036	0.028	-0.091 – 0.018	-1.309	.191
FSMI_S	0.051	0.042	-0.031 – 0.133	1.225	.221
FSMI_KF	-0.034	0.029	-0.091 – 0.023	-1.177	.239
IUS	0.025	0.035	-0.044 – 0.094	0.711	.477
STAIT_TA	-0.032	0.038	-0.106 – 0.042	-0.852	.394
IRI_F	-0.060	0.027	-0.113 – -0.008	-2.254	.024†
IRI_EC	0.007	0.034	-0.061 – 0.074	0.191	.849
IRI_PT	0.044	0.030	-0.014 – 0.103	1.494	.135
IRI_PD	0.051	0.033	-0.014 – 0.115	1.536	.125
SDO_PTD	0.079	0.032	0.016 – 0.141	2.455	.014†

SDO_CTD	-0.035	0.028	-0.090 – 0.019	-1.263	.207
SDO_PTA	0.003	0.031	-0.058 – 0.063	0.085	.932
SDO_CTA	-0.035	0.033	-0.099 – 0.029	-1.069	.285
SD3_M	0.024	0.037	-0.048 – 0.096	0.649	.516
SD3_N	0.036	0.039	-0.040 – 0.113	0.926	.354
SD3_P	0.130	0.042	0.048 – 0.211	3.128	.002
GRP	-0.033	0.035	-0.102 – 0.037	-0.926	.355
BPAQ_PA	-0.028	0.039	-0.104 – 0.048	-0.721	.471
BPAQ_VA	0.071	0.034	0.004 – 0.139	2.074	.038†
BPAQ_A	-0.070	0.041	-0.150 – 0.010	-1.720	.086
BPAQ_H	0.070	0.042	-0.012 – 0.153	1.675	.094
IDAQ	0.120	0.026	0.069 – 0.171	4.631	<.001
LOT	0.069	0.035	0.001 – 0.137	1.989	.047†
DSR_ARD	0.066	0.024	0.018 – 0.114	2.697	.007†
FMPS_CM	0.007	0.041	-0.074 – 0.087	0.164	.870
FMPS_PS	0.009	0.036	-0.062 – 0.080	0.259	.796
FMPS_PE	-0.027	0.032	-0.090 – 0.037	-0.826	.409
FMPS_PC	0.018	0.033	-0.046 – 0.082	0.549	.583
FMPS_DA	0.036	0.038	-0.038 – 0.109	0.947	.344
FMPS_O	0.035	0.028	-0.020 – 0.091	1.249	.212
ERQ_CR	-0.022	0.028	-0.076 – 0.033	-0.790	.430
ERQ_ES	0.091	0.027	0.037 – 0.144	3.328	.001
SM	-0.024	0.026	-0.074 – 0.026	-0.937	.349
HEXACO_HH	0.049	0.033	-0.016 – 0.114	1.485	.138
TFS_PF	-0.046	0.028	-0.101 – 0.010	-1.616	.106
TFS_CF	-0.000	0.028	-0.056 – 0.055	-0.010	.992
TFS_FF	-0.026	0.032	-0.089 – 0.038	-0.801	.423
SOII_SI	-0.118	0.046	-0.208 – -0.028	-2.562	.010†
SOII_OI	0.104	0.037	0.032 – 0.176	2.833	.005
DUREL_ORA	0.058	0.033	-0.008 – 0.123	1.733	.083
DUREL_NORA	-0.056	0.032	-0.119 – 0.008	-1.724	.085
DUREL_IR	0.042	0.034	-0.025 – 0.109	1.225	.221
INCOM_A	0.072	0.034	0.006 – 0.138	2.135	.033†
INCOM_O	-0.089	0.031	-0.149 – -0.030	-2.930	.003
LSE	0.072	0.038	-0.002 – 0.147	1.896	.058
GSE	-0.033	0.039	-0.110 – 0.044	-0.836	.403
LCT	-0.021	0.031	-0.083 – 0.040	-0.684	.494
AGE (COVARIATE)	0.000	0.031	-0.060 – 0.061	0.010	.992
INCM (COVARIATE)	-0.029	0.027	-0.083 – 0.025	-1.068	.286
LBCN (COVARIATE)	0.041	0.026	-0.011 – 0.092	1.534	.125
FAM (COVARIATE)	0.022	0.033	-0.042 – 0.087	0.687	.492
FI (COVARIATE)	-0.039	0.037	-0.112 – 0.033	-1.062	.288
DN (COVARIATE)	0.129	0.032	0.065 – 0.192	3.976	<.001
IN (COVARIATE)	-0.481	0.028	-0.536 – -0.426	-17.070	<.001

Note. Model $R^2 = .331$. The model had 2097 residual degrees of freedom. For clarification of predictor name abbreviations, see Supplementary Table 11. We used t-tests (two-sided) to assess the significance of the coefficients, and the significance criterion was $p < .05$. The table contains raw p-values: symbol † indicates coefficients that were initially significant but stopped being significant after the FDR correction[170] for multiple comparisons was applied (see pp.39-40). The cells that are highlighted in grey indicate individual differences identified as the most robust predictors because these were amongst the 30 most

important predictors across all six machine learning models tested in Study 6 (see Supplementary Table 12) while remaining statistically significant in the linear least squares model presented in this table.

Supplementary Table 15: Coefficients for the Liner Least Squares Model Computed for the Competence Dimension (Study 6)

Variable	b	SE b	95% CI	t	p
(Intercept)	5.407	0.017	5.375 – 5.440	326.045	<.001
PANAS_TPA	0.027	0.027	-0.026 – 0.079	1.000	.318
PANAS_TNA	-0.018	0.026	-0.070 – 0.033	-0.699	.485
LST	-0.010	0.029	-0.066 – 0.046	-0.355	.722
BFI_N	-0.062	0.031	-0.123 – -0.002	-2.025	.043†
BFI_E	-0.042	0.021	-0.083 – -0.001	-1.991	.047†
BFI_C	0.038	0.026	-0.013 – 0.090	1.450	.147
BFI_A	-0.007	0.026	-0.059 – 0.044	-0.286	.775
BFI_O	0.020	0.023	-0.025 – 0.065	0.889	.374
ATQ_AP	0.099	0.028	0.044 – 0.153	3.538	<.001
ATQ_AV	-0.005	0.033	-0.070 – 0.059	-0.165	.869
BSSS_ES	-0.009	0.023	-0.054 – 0.036	-0.398	.691
BSSS_BS	-0.030	0.023	-0.075 – 0.015	-1.294	.196
BSSS_TA	0.043	0.029	-0.013 – 0.100	1.501	.134
BSSS_D	0.037	0.031	-0.023 – 0.097	1.209	.227
BPNSFS_AS	0.030	0.029	-0.026 – 0.086	1.065	.287
BPNSFS_AF	0.063	0.027	0.010 – 0.115	2.345	.019†
BPNSFS_RS	0.029	0.027	-0.024 – 0.081	1.068	.285
BPNSFS_RF	-0.033	0.032	-0.095 – 0.029	-1.049	.294
BPNSFS_CS	-0.035	0.033	-0.099 – 0.029	-1.083	.279
BPNSFS_CF	-0.031	0.034	-0.098 – 0.036	-0.908	.364
PVQ5X_SS	0.084	0.027	0.030 – 0.138	3.064	.002
PVQ5X_H	-0.017	0.025	-0.066 – 0.031	-0.698	.486
PVQ5X_CI	0.036	0.024	-0.012 – 0.084	1.474	.140
PVQ5X_UC	-0.007	0.029	-0.063 – 0.048	-0.261	.794
PVQ5X_PD	0.039	0.030	-0.019 – 0.098	1.330	.184
PVQ5X_UN	-0.015	0.024	-0.061 – 0.032	-0.612	.540
PVQ5X_F	-0.004	0.027	-0.057 – 0.048	-0.160	.873
PVQ5X_SP	-0.004	0.027	-0.058 – 0.049	-0.153	.879
PVQ5X_CR	0.010	0.025	-0.039 – 0.060	0.412	.680
PVQ5X_T	0.003	0.027	-0.049 – 0.055	0.098	.922
NCS	-0.001	0.022	-0.044 – 0.042	-0.051	.959
FSMI_AEC	-0.049	0.027	-0.101 – 0.003	-1.839	.066
FSMI_AI	-0.021	0.022	-0.065 – 0.022	-0.972	.331
FSMI_S	-0.027	0.033	-0.092 – 0.038	-0.815	.415
FSMI_KF	0.021	0.023	-0.025 – 0.066	0.898	.369
IUS	0.062	0.028	0.007 – 0.116	2.203	.028†
STAIT_TA	0.084	0.030	0.026 – 0.143	2.827	.005†
IRI_F	0.014	0.021	-0.027 – 0.056	0.670	.503
IRI_EC	0.017	0.027	-0.036 – 0.071	0.628	.530
IRI_PT	0.006	0.024	-0.041 – 0.052	0.243	.808
IRI_PD	0.013	0.026	-0.038 – 0.064	0.494	.621
SDO_PTD	-0.015	0.025	-0.065 – 0.034	-0.607	.544

SDO_CTD	-0.007	0.022	-0.050 – 0.037	-0.305	.761
SDO_PTA	-0.035	0.025	-0.083 – 0.014	-1.406	.160
SDO_CTA	0.065	0.026	0.014 – 0.116	2.515	.012†
SD3_M	-0.001	0.029	-0.059 – 0.056	-0.050	.960
SD3_N	-0.000	0.031	-0.061 – 0.060	-0.011	.991
SD3_P	-0.030	0.033	-0.094 – 0.035	-0.896	.370
GRP	0.034	0.028	-0.022 – 0.089	1.189	.234
BPAQ_PA	-0.026	0.031	-0.086 – 0.035	-0.828	.408
BPAQ_VA	-0.021	0.027	-0.075 – 0.033	-0.766	.444
BPAQ_A	0.008	0.032	-0.055 – 0.071	0.249	.803
BPAQ_H	-0.005	0.033	-0.071 – 0.060	-0.155	.877
IDAQ	0.020	0.021	-0.020 – 0.061	0.991	.322
LOT	-0.017	0.028	-0.071 – 0.037	-0.612	.540
DSR_ARD	0.005	0.019	-0.033 – 0.043	0.273	.785
FMPS_CM	0.013	0.033	-0.051 – 0.077	0.393	.694
FMPS_PS	-0.030	0.029	-0.086 – 0.027	-1.027	.304
FMPS_PE	0.033	0.026	-0.017 – 0.083	1.289	.198
FMPS_PC	-0.044	0.026	-0.095 – 0.007	-1.695	.090
FMPS_DA	0.008	0.030	-0.051 – 0.066	0.252	.801
FMPS_O	-0.002	0.023	-0.046 – 0.043	-0.068	.946
ERQ_CR	0.062	0.022	0.018 – 0.105	2.793	.005†
ERQ_ES	0.002	0.022	-0.041 – 0.044	0.076	.939
SM	0.007	0.020	-0.033 – 0.047	0.337	.736
HEXACO_HH	-0.044	0.026	-0.095 – 0.008	-1.656	.098
TFS_PF	0.057	0.022	0.013 – 0.101	2.545	.011†
TFS_CF	-0.012	0.023	-0.056 – 0.032	-0.522	.602
TFS_FF	-0.015	0.026	-0.065 – 0.036	-0.573	.566
SOII_SI	0.078	0.037	0.006 – 0.150	2.122	.034†
SOII_OI	-0.005	0.029	-0.062 – 0.052	-0.177	.860
DUREL_ORA	0.009	0.027	-0.043 – 0.061	0.326	.744
DUREL_NORA	0.028	0.026	-0.023 – 0.078	1.080	.280
DUREL_IR	-0.044	0.027	-0.097 – 0.010	-1.599	.110
INCOM_A	-0.048	0.027	-0.100 – 0.005	-1.770	.077
INCOM_O	0.064	0.024	0.017 – 0.112	2.642	.008†
LSE	-0.003	0.030	-0.063 – 0.056	-0.115	.908
GSE	0.021	0.031	-0.041 – 0.082	0.659	.510
LCT	0.032	0.025	-0.016 – 0.081	1.304	.193
AGE (COVARIATE)	0.077	0.025	0.029 – 0.125	3.134	.002
INCM (COVARIATE)	0.028	0.022	-0.014 – 0.071	1.302	.193
LBCN (COVARIATE)	-0.027	0.021	-0.068 – 0.014	-1.278	.201
FAM (COVARIATE)	0.094	0.026	0.043 – 0.145	3.615	<.001
FI (COVARIATE)	-0.068	0.029	-0.126 – -0.010	-2.318	.021†
DN (COVARIATE)	-0.007	0.026	-0.057 – 0.044	-0.253	.800
IN (COVARIATE)	0.249	0.022	0.205 – 0.293	11.115	<.001

Note. Model R^2 = .374. The model had 2097 residual degrees of freedom. For clarification of predictor name abbreviations, see Supplementary Table 11. We used t-tests (two-sided) to assess the significance of the coefficients, and the significance criterion was $p < .05$. The table contains raw p-values: symbol † indicates coefficients that were initially significant but stopped being significant after the FDR correction[170] for multiple comparisons was applied (see pp.41-42). The cells that are highlighted in grey indicate individual differences identified as the most robust predictors because these were amongst the 30 most

important predictors across all six machine learning models tested in Study 6 (see Supplementary Table 12) while remaining statistically significant in the linear least squares model presented in this table.

Supplementary Table 16: Main Individual Difference Predictors of the Positive, Negative, and Competence Dimensions (Study 7)

Variable	b	SE b	99% CI	t	p	f^2
DV = Positive Dimension						
Model 1: GRP positively predicts the DV						
(Constant)	2.938	0.086	2.717 – 3.159	34.329	<.001	1.102
GRP	0.145	0.035	0.054 – 0.236	4.099	<.001	0.016
Model 2: IDAQ positively predicts the DV						
(Constant)	2.791	0.068	2.615 – 2.967	40.888	<.001	1.564
IDAQ	0.178	0.023	0.120 – 0.236	7.868	<.001	0.058
Model 3: FMPS_PE positively predicts the DV						
(Constant)	2.786	0.107	2.511 – 3.061	26.137	<.001	0.639
FMPS_PE	0.158	0.034	0.071 – 0.245	4.684	<.001	0.021
DV = Negative Dimension						
Model 4: PANAS_TNA positively predicts the DV						
(Constant)	2.090	0.078	1.888 – 2.292	26.734	<.001	0.669
PANAS_TNA	0.124	0.047	0.003 – 0.245	2.634	.009	0.006
Model 5: IDAQ positively predicts the DV						
(Constant)	2.133	0.060	1.979 – 2.287	35.714	<.001	1.193
IDAQ	0.056	0.020	0.005 – 0.107	2.840	0.005	0.008
Model 6: SD3_P positively predicts the DV						
(Constant)	1.707	0.098	1.456 – 1.959	17.496	<.001	0.286
SD3_P	0.296	0.048	0.172 – 0.420	6.152	<.001	0.035
Model 7: ERQ_ES positively predicts the DV						
(Constant)	2.041	0.083	1.827 – 2.255	24.586	<.001	0.565
ERQ_ES	0.064	0.021	0.010 – 0.117	3.085	.002	0.009
DV = Competence Dimension						
Model 8: ATQ_AP positively predicts the DV						
(Constant)	4.470	0.151	4.079 – 4.860	29.527	<.001	0.816
ATQ_AP	0.157	0.029	0.081 – 0.233	5.345	<.001	0.027
Model 9: PVQ5X_SS positively predicts the DV						
(Constant)	4.859	0.106	4.586 – 5.133	45.797	<.001	1.962
PVQ5X_SS	0.097	0.024	0.034 – 0.160	3.962	<.001	0.015

Note. DV = dependent variable. Model 1 R^2 = .015; Model 2 R^2 =.055; Model 3 R^2 = .020; Model 4 R^2 = .006; Model 5 R^2 = .007; Model 6 R^2 = .034; Model 7 R^2 = .009; Model 8 R^2 = .026; and Model 9 R^2 = .014. All models had 1069 residual degrees of freedom. In all models, we used t-tests (two-sided) to assess the significance of the coefficients, and the significance criterion was p < .010 based on the Benjamini-Yekutieli (B-Y) correction[173,174] for multiple comparisons. The table contains raw p-values that are statistically significant if they meet this benchmark; therefore, all nine predictors reached statistical significance. f^2 refers to Cohen's f^2 effect size[176]. Predictors: GRP = general risk propensity[191]; IDAQ = anthropomorphism[46]; FMPS_PE = parental expectations[192]; PANAS_TNA = trait negative affect[193]; SD3_P = psychopathy[194]; ERQ_ES = expressive

suppression[195]; ATQ_AP = approach temperament[97]; and PVQ5X_SS = security-societal[196]. GRP and FMPS_PE were measured on a 1-5 scale (1=Strongly disagree, 5=Strongly agree); IDAQ was measured on a 0-10 scale (0=Not at all, 10=Very much); PANAS_TNA was measured on a 1-5 scale (1=Strongly disagree, 5=Strongly agree); SD3_P was measured on a 1-5 scale (1=Disagree strongly, 5=Agree strongly); ERQ_ES and ATQ_AP were measured on a 1-7 scale (1=Strongly disagree, 7=Strongly agree); and PVQ5X_SS was measured on a 1-6 scale (1=Not like me at all, 6=Very much like me).

Supplementary Table 17: All Variables that Were Tested as Mediators, Their Mediated Effects (in Parentheses), and Their Justification, Listed Under the Relevant Individual Difference Predictors of the Positive, Negative, and Competence Dimensions (Study 7)

Mediator	Justification
DV = Positive Dimension	
Mediators Tested for the Predictor GRP	
GRP_M1 (ab = 0.001, 99% $CI_{bootstrapped}$ = [-0.006, 0.010], $ab_{\%}$ = 0.007). I think robots do not pose a risk to me.	People high (vs. low) in risk propensity may undertake more risky behaviors because they perceive such behaviors as less risky[281,282]. In line with this rationale, people high in risk propensity may be more desensitized to risk and thus perceive robots as less risky and threatening to them, which would result in more positive psychological processes toward robots.
GRP_M2 (ab < 0.001, 99% $CI_{bootstrapped}$ = [-0.016, 0.016], $ab_{\%}$ = 0.002). I think robots do not pose a risk to society.	Similar to the explanation above, people who are high in risk propensity may be more desensitized to risk and thus feel that robots pose less risk to their society, which would result in more positive psychological processes.
GRP_M3 (ab = 0.057, 99% $CI_{bootstrapped}$ = [0.029, 0.093], $ab_{\%}$ = 0.393). I think robot adoption has its risks, but these risks are what makes robots appealing.	One of the core characteristics of high risk propensity is being attracted to risky behaviors and choices[191]. In that regard, people high in risk propensity may actually perceive robots as risky and thus experience more positive psychological processes because they are attracted to the risk.
GRP_M4 (ab = 0.013, 99% $CI_{bootstrapped}$ = [0.001, 0.032], $ab_{\%}$ = 0.090). I am curious to see how robots will change the world.	Risk taking is typically associated with curiosity and openness to experience[191,283], and risk takers may have curiosity about how robots will change the world, thus experiencing positive psychological processes toward them.
GRP_M5 (ab = -0.003, 99% $CI_{bootstrapped}$ = [-0.018, 0.009], $ab_{\%}$ = -0.021). I think robot adoption has its risks, but the potential rewards are high.	Willingness to pursue risks is typically associated with promotion focus (i.e., the motivation to attain rewards and positive outcomes[284,285]). In that regard, people high in risk propensity may experience positive

	psychological processes because they associate robot adoption with high potential rewards.
GRP_M6 (ab = -0.001, 99% $CI_{bootstrapped}$ = [-0.010, 0.006], $ab_{\%}$ = -0.007). The benefits of robots outweigh their risks.	This mediator represents a more general conceptualization of the mediator above, according to which people may generally weigh the benefits and risks associated with robots, and for those high in risk propensity the benefits may outweigh the risks, thus leading to more positive psychological processes.
GRP_M7 (ab = 0.020, 99% $CI_{bootstrapped}$ = [-0.004, 0.048], $ab_{\%}$ = 0.138). a) I feel that technology helps me to align with my ideal self; and b) I feel that technology helps me to succeed in my endeavors. [a]	Considering that high risk propensity goes hand in hand with promotion focus[284,285], and that attaining success and aligning with the ideal self are amongst core promotion focus goals[286], it is plausible that high risk-takers may value robots because technology helps them achieve success and align with their ideal self.
Mediators Tested for the Predictor IDAQ [b]	
IDAQ_M1 (ab = 0.004, 99% $CI_{bootstrapped}$ = [-.009, .019], $ab_{\%}$ = 0.022). When I interact with a non-human entity (e.g., robots, machines, nature, animals), I can experience strong emotions that I would normally experience toward human beings.	This mediator was created to try to explain why IDAQ predicted participants' responses regarding both the positive and negative dimensions. According to the concept of affect intensity, people differ regarding the strength of feelings they can experience[287]. For example, people high (vs. low) in this trait can experience stronger positive and negative affect. Considering that people high (vs. low) in anthropomorphism should be more likely to get emotionally connected to non-living entities such as robots[45,46,288], perhaps they also have a higher propensity to experience more intense feelings regarding robots, regardless of their valence. In that regard, such individuals would be able to experience both more positive and more negative psychological reactions to robots.
IDAQ_M2 (ab = 0.021, 99% $CI_{bootstrapped}$ = [0.008, 0.039], $ab_{\%}$ = 0.118). Interacting with non-human entities (e.g., robots, machines, nature, animals) helps me fulfil the need to experience strong emotions regularly.	IDAQ_M2 expands the mediator above by connecting it to the concept of need for affect[289], which captures the importance of experiencing strong emotions (positive or negative) regularly. In that respect, it is possible that individuals high (vs. low) in anthropomorphism have the need to experience strong emotions regularly, and they are more likely to

	experience emotions toward non-living entities to assuage this need. As a result, high trait anthropomorphism may be reflected in both more positive and more negative psychological processes regarding robots.
IDAQ_M3 (ab = 0.035, 99% $CI_{bootstrapped}$ = [0.016, 0.060], $ab_{\%}$ = 0.197). When I see a non-human entity (e.g., robots, machines, nature, animals) that has human characteristics, I experience positive feelings.	One of the assumptions that can be inferred from theorizing about anthropomorphism[45,46,288] is that this construct is linked to more positive experiences of non-living entities (e.g., robots). In that regard, people high (vs. low) in anthropomorphism should experience more positive feelings whenever they encounter a robot, which should be reflected in generally more positive psychological processes. Therefore, IDAQ_M3 should mediate the link between anthropomorphism and the positive dimension, but not the negative dimension.
IDAQ_M4 (ab = -0.001, 99% $CI_{bootstrapped}$ = [-.007, .004], $ab_{\%}$ = -0.006). When I see a non-human entity (e.g., robots, machines, nature, animals) that has human characteristics, I experience negative feelings.	Although anthropomorphism should theoretically be associated with more positive psychological processes toward robots[45,46,288], one way to explain its association with the negative psychological processes would be by examining it from the perspective of the uncanny valley[3,205,206,290,291]. According to this construct, robots closely resembling humans but without a realistic human-like appearance evoke eeriness or creepiness and other negative feelings. In that regard, people high (vs. low) in anthropomorphism may see robots as more human-like and may therefore be more likely to experience the uncanny valley, which would result in various negative psychological processes. If this logic were accurate, IDAQ_M4 would mediate the link between anthropomorphism and the negative dimension, but not the positive dimension. It is important to emphasize that the uncanny valley has been criticized, as various studies have failed to support it[207,208], and we assessed IDAQ_M4 that is underpinned by this construct just to ensure we did not miss any options we could identify.
Mediators Tested for the Predictor FMPS_PE	
FMPS_PE_M1 (ab = 0.031, 99% $CI_{bootstrapped}$ = [0.009, 0.058], $ab_{\%}$ = 0.196). I value robots because they are closer to perfection than humans.	Considering that parental expectations is a dimension of perfectionism and indicates to what degree people's family environment was characterized by perfectionistic expectations[192], one possibility is that

	participants who score high (vs. low) on this trait perceive robots more positively because they associate robots with perfectionism to a greater extent than human beings.
FMPS_PE_M2 ($ab = 0.027$, 99% $CI_{bootstrapped} = [0.006, 0.055]$, $ab_{\%} = 0.171$). I value robots because I believe they can help me fulfil my own high expectations.	Similarly, considering that robots can be efficient in what they do and aid people in various tasks and pursuits[39–42], people high (vs. low) in parental expectations may perceive robots more positively because robots can help them fulfill their own high expectations in life (e.g., by assisting them in various tasks and pursuits where they want to excel, by making their life easier so they can focus on their goals, etc.).
FMPS_PE_M3 ($ab = 0.011$, 99% $CI_{bootstrapped} = [-0.024, 0.048]$, $ab_{\%} = 0.070$). I value robots because I believe they can help me fulfil my parents' high expectations.	This mediator is an alternative version of the mediator above and was created to understand more clearly whether the positive link between parental expectations and the positive dimension is driven by robots being helpful to participants in fulfilling their own high expectations, or their parents' high expectations. This is important to discern because items assessing FMPS_PE do not make it clear whether this individual difference shapes the positive dimension due to the parents' high expectations, or due to participants' own high expectations they adopted from their parents.
FMPS_PE_M4 ($ab = 0.009$, 99% $CI_{bootstrapped} = [-0.013, 0.032]$, $ab_{\%} = 0.057$). I value robots because their superiority over humans allows me to become superior over others.	This mediator is an extension of FMPS_PE_M2 and FMPS_PE_M3. Given that perfectionism has been associated with the urge to feel superior over others[292,293], one possibility is that people high (vs. low) in parental expectations may perceive robots positively because they think that robots can enable them to be superior to other humans.
FMPS_PE_M5 ($ab = 0.026$, 99% $CI_{bootstrapped} = [-0.012, 0.065]$, $ab_{\%} = 0.165$). I value robots because I believe they help me better cope with my parents' high expectations of me.	Given that perfectionism typically has negative consequences for wellbeing[294], it is possible that people high (vs. low) in parental expectations may feel positive toward robots because they think robots can help them cope with their parents' high expectations of themselves (e.g., by providing them emotional support as companions or sources of entertainment, or by assisting them in various tasks and pursuits where they are expected to excel).

FMPS_PE_M6 (ab = 0.033, 99% $CI_{bootstrapped}$ = [0.011, 0.062], $ab_{\%}$ = 0.209). I value robots because I believe they help me better cope with my own high expectations of myself.	Alternatively, people high (vs. low) in parental expectations may feel positive toward robots because they think robots can help them cope with their own high expectations of themselves (e.g., by providing them emotional support as companions or sources of entertainment, or by assisting them in various tasks and pursuits where they want to excel).
DV = Negative Dimension	
Mediators Tested for the Predictor PANAS_TNA	
Activated Displeasure—12-PAC_AD (ab = 0.231, 99% $CI_{bootstrapped}$ = [0.029, 0.450], $ab_{\%}$ = 1.863); Deactivated Displeasure—12-PAC_DD (ab = -0.019, 99% $CI_{bootstrapped}$ = [-0.180, 0.147], $ab_{\%}$ = -0.153); Displeasure—12-PAC_D (ab = -0.030, 99% $CI_{bootstrapped}$ = [-0.228, 0.173], $ab_{\%}$ = -0.242); Unpleasant Activation—12-PAC_UA (ab = -0.073, 99% $CI_{bootstrapped}$ = [-0.231, 0.081], $ab_{\%}$ = -0.589); Unpleasant Deactivation—12-PAC_UD (ab = 0.033, 99% $CI_{bootstrapped}$ = [-0.054, 0.130], $ab_{\%}$ = 0.266) [c] Measured using 12-Point Affect Circumplex[197].	People who are high in trait negative affect are generally more likely to experience negative state affect across different situations[193,230]. Such people may therefore be inclined to experience negative psychological processes regarding robots simply because they generally feel more negative. To test this premise, we measured state negative affect using 12-PAC[197] because this measure comprises its most nuanced conceptualization. We used all five 12-PAC[197] subscales concerning state negative affect because there was no theoretical guidance regarding which one might be most important.
Mediators Tested for the Predictor IDAQ [b]	
IDAQ_M1 (ab = 0.001, 99% $CI_{bootstrapped}$ = [-0.011, 0.014], $ab_{\%}$ = 0.018); IDAQ_M2 (ab = 0.008, 99% $CI_{bootstrapped}$ = [-0.002, 0.022], $ab_{\%}$ = 0.143); IDAQ_M3 (ab = -0.011, 99% CI = [-0.026, -0.001], $ab_{\%}$ = -0.196); IDAQ_M4 (ab = -0.005, 99% $CI_{bootstrapped}$ = [-0.021, 0.011], $ab_{\%}$ = -0.089). [d] Same as for "Positive Dimension: IDAQ"	For mediator justifications, see "Positive Dimension: Mediators Tested for the Predictor IDAQ"
Mediators Tested for the Predictor SD3_P	
SD3_P_M1 (ab = -0.011, 99% $CI_{bootstrapped}$ = [-0.075, 0.050], $ab_{\%}$ = -0.037). I tend to have negative feelings toward other people.	People high (vs. low) in psychopathy tend to exploit and pick on other people[194], and they generally dislike others[295]. They may project these negative feelings to robots, thus experiencing more negative psychological processes toward them.

SD3_P_M2 (ab = 0.122, 99% $CI_{bootstrapped}$ = [0.070, 0.183], $ab_{\%}$ = 0.412). I tend to have negative feelings toward other people's creations and inventions.	Negative feelings that people high (vs. low) in psychopathy have toward other humans[295] may generalize to any creations and inventions made by humans (e.g., robots), thus leading to more negative perceptions of robots.
SD3_P_M3 (ab = 0.022, 99% $CI_{bootstrapped}$ = [0.005, 0.050], $ab_{\%}$ = 0.074). Using technologies that I am not proficient in makes me feel inferior.	Psychopaths tend to be characterized by a high desire for control[296,297]. Advanced technologies such as robots, in which they are not necessarily proficient, might make them feel inferior and result in negative psychological processes.
SD3_P_M4 (ab = -0.008, 99% $CI_{bootstrapped}$ = [-0.036, 0.020], $ab_{\%}$ = -0.027). Technology can expose me for who I am.	Psychopaths generally hide their "true selves"—they tend to lie to get what they want and are good at hiding their emotions[194,298]. Therefore, individuals high (vs. low) in psychopathy may perceive robots more negatively because technology makes them feel exposed and vulnerable as it makes their hiding more difficult (e.g., by collecting and analyzing the data about their physiological responses they cannot easily hide, by inferring their personality traits from their digital footprints, etc[299,300].
12-PAC_AD (ab = 0.064, 99% $CI_{bootstrapped}$ = [0.010, 0.136], $ab_{\%}$ = 0.216); 12-PAC_DD (ab = -0.005, 99% $CI_{bootstrapped}$ = [-0.061, 0.051], $ab_{\%}$ = -0.017); 12-PAC_D (ab = -0.028, 99% $CI_{bootstrapped}$ = [-0.098, 0.037], $ab_{\%}$ = -0.095); 12-PAC_UA (ab = -0.017, 99% $CI_{bootstrapped}$ = [-0.067, 0.022], $ab_{\%}$ = -0.057); 12-PAC_UD (ab = -0.003, 99% $CI_{bootstrapped}$ = [-0.037, 0.029], $ab_{\%}$ = -0.010). [c] Same as for "Negative Dimension: PANAS_TNA"[197].	Psychopathy is associated with high levels of depression and negative affect (e.g., ref.[301]). Therefore, similar to the individuals high in trait negative affect, those high (vs. low) in psychopathy may experience negative psychological processes because they generally feel more negative. To test this premise, we again used all five negative affect subscales from 12-PAC[197] because there was no theoretical guidance regarding which ones might be the most important as mediators.
Mediators Tested for the Predictor ERQ_ES	
ERQ_ES_M1 (ab = 0.007, 99% $CI_{bootstrapped}$ = [-0.010, 0.029], $ab_{\%}$ = 0.109). At the moment, I feel mentally exhausted.	Research indicates that suppressing emotions can result in a state of depletion[302,303]. In that regard, people high (vs. low) in expressive suppression may feel mental exhaustion, which is an indicator of depletion, and thus experience more negative psychological processes regarding their surroundings, including robots.
ERQ_ES_M2 (ab = 0.007, 99% $CI_{bootstrapped}$ = [-0.012, 0.027], $ab_{\%}$ = 0.109). At the moment, I feel emotionally exhausted.	This mediator is a different variant of the one above, in which we are referring to emotional rather than mental exhaustion.

12-PAC_AD (ab = 0.017, 99% $CI_{bootstrapped}$ = [-0.002, 0.043], $ab_{\%}$ = 0.266); 12-PAC_DD (ab = -0.005, 99% $CI_{bootstrapped}$ = [-0.026, 0.013], $ab_{\%}$ = -0.078); 12-PAC_D (ab = -0.008, 99% $CI_{bootstrapped}$ = [-0.037, 0.014], $ab_{\%}$ = -0.125); 12-PAC_UA (ab = -0.007, 99% $CI_{bootstrapped}$ = [-0.023, 0.003], $ab_{\%}$ = -0.109); 12-PAC_UD (ab = -0.001, 99% $CI_{bootstrapped}$ = [-0.013, 0.009], $ab_{\%}$ = -0.016). [c] Same as for "Negative Dimension: PANAS_TNA"[197].	Expressive suppression is related to lower life satisfaction, depressive symptoms, and rumination[195]. Therefore, similar to individuals high in trait negative affect and psychopathy, those high (vs. low) in psychopathy may experience negative psychological processes because they generally feel more negative. To test this premise, we again used all five negative affect subscales from 12-PAC[197] because there was no theoretical guidance regarding which ones might be the most important as mediators.
DV = Competence Dimension	
Mediators Tested for the Predictor ATQ_AP	
ATQ_AP_M1 (ab = 0.008, 99% $CI_{bootstrapped}$ = [-0.004, 0.026], $ab_{\%}$ = 0.051). I value robots that can help me perform better than others.	People high (vs. low) in approach temperament are motivated by good performance and performing better than others[68,97,304]. As a result, they may place a higher value on robots that can help them perform better than others, thus seeing these robots as more competent.
ATQ_AP_M2 (ab = 0.015, 99% $CI_{bootstrapped}$ = [-0.007, 0.042], $ab_{\%}$ = 0.096). I value robots that can help me become better at a task, goal, or skill that I want to accomplish or master.	As people high (vs. low) in approach temperament are interested in performing well and improving their performance[68,97,304], they may place higher value on robots who can help them in this regard, thus seeing these robots as more competent.
ATQ_AP_M3 (ab = -0.010, 99% $CI_{bootstrapped}$ = [-0.029, 0.005], $ab_{\%}$ = -0.064). When evaluating other people, it is important to me how good they are at what they do.	Considering that approach temperament is linked to exceptional performance[97,304,305], people high (vs. low) in this temperament may place higher value on people who are good at what they do, and this perception may also extend to robots, thus leading to robots being perceived as more competent.
ATQ_AP_M4 (ab = 0.012, 99% $CI_{bootstrapped}$ = [-0.001, 0.030], $ab_{\%}$ = 0.076). When evaluating robots, it is important to me how good they are at what they do.	This mediator is a more direct version of the mediator above and concerns evaluating robots themselves rather than humans in terms of their competence at what they do.
ATQ_AP_M5 (ab = 0.020, 99% $CI_{bootstrapped}$ = [0.002, 0.042], $ab_{\%}$ = 0.127). I highly value exceptional skills and competencies.	If people high (vs. low) in approach temperament value and try to attain exceptional performance[97,304,305], then perceiving robots as more competent may reflect the high importance they place on these

	characteristics, assuming that they generally associate robots with exceptional skills and competencies.
ATQ_AP_M6 (ab = 0.003, 99% $CI_{bootstrapped}$ = [-0.021, 0.027], $ab_{\%}$ = 0.019). When I see a human that can accomplish something challenging, I react strongly to it.	Considering that people high (vs. low) in approach temperament value exceptional performance[97,304,305], they may generally react to human competence with strong emotions, which may be reflected in an elevated appraisal of this quality. This intense reaction may also extend to robots, thus leading to robots being perceived as more competent.
ATQ_AP_M7 (ab = -0.006, 99% $CI_{bootstrapped}$ = [-0.032, 0.020], $ab_{\%}$ = -0.038). When I see a robot that can accomplish something challenging, I react strongly to it.	This mediator is a more direct version of the mediator above and concerns evaluating robot competence directly rather than in association with humans.
ATQ_AP_M8 (ab = 0.011, 99% $CI_{bootstrapped}$ = [-0.012, 0.037], $ab_{\%}$ = 0.070). When I see the potential for robots to improve human life, I get excited.	Approach temperament is inherently about moving toward something positive[68,97]. In that regard, people high (vs. low) in approach temperament may get excited in relation to robots' potential to improve human life, which may in turn lead to inflated estimates of robots' competence.
ATQ_AP_M9 (ab = 0.017, 99% $CI_{bootstrapped}$ = [-0.010, 0.047], $ab_{\%}$ = 0.108). When I encounter robots or other inventions that can better my life, I react strongly to it.	This mediator is a self-centered version of the mediator above, with the focus on the improvement of participants' own lives rather than human life in general.
ATQ_AP_M10 (ab = 0.018, 99% $CI_{bootstrapped}$ = [-0.002, 0.043], $ab_{\%}$ = 0.115). I am thrilled when seeing robots helping society to achieve tasks that are often difficult to accomplish.	Considering that approach temperament is about accomplishments and achievements[68,97,304], it is possible that people high (vs. low) in this trait are thrilled in relation to robots' potential to help society achieve difficult tasks, which may in turn lead to inflated estimates of robots' competence.
Mediators Tested for the Predictor PVQ5X_SS	
PVQ5X_SS_M1 (ab = 0.009, 99% $CI_{bootstrapped}$ = [-0.005, 0.027], $ab_{\%}$ = 0.093). I think advanced technology (e.g., robots, machines, devices) can make the country more powerful.	Security-societal is about valuing the ability of one's country or society to protect itself and its citizens, and to maintain stability[196]. In that regard, people high in this trait may think that advanced technology such as robots may make the country more powerful, which could lead to inflated estimates of their competence.

PVQ5X_SS_M2 (ab = 0.016, 99% $CI_{bootstrapped}$ = [-0.008, 0.042], $ab_{\%}$ = 0.165). With effective use of advanced technology (e.g., robots, machines, devices), the country maintains its strength to defend its citizens.	Considering that people high in security-societal value their country's safety[196], they may associate advanced technology such as robots with the country's ability to maintain safety, which could lead to inflated estimates of robots' competence.
PVQ5X_SS_M3 (ab = 0.004, 99% $CI_{bootstrapped}$ = [-0.003, 0.015], $ab_{\%}$ = 0.041). I think advanced technology (e.g., robots, machines, devices) can create order and stability.	Similar to the item above, considering that people high in security-societal value order and stability[196], they may associate advanced technology such as robots with their society's ability to maintain order and stability, which could lead to inflated estimates of robots' competence.
PVQ5X_SS_M4 (ab = 0.015, 99% $CI_{bootstrapped}$ = [<0.001, 0.036], $ab_{\%}$ = 0.155). Advanced technology (e.g., robots, machines, devices) is a reflection of how powerful our society is.	Advanced technology, such as robots, may also be a reflection of how powerful one's society is. In that regard, considering that people high (vs. low) in security-societal value their society being powerful (i.e., being able to protect itself and its citizens[196]), they may inflate the competence of robots because they see this quality as an indicator of their society's power.
PVQ5X_SS_M5 (ab = 0.007, 99% $CI_{bootstrapped}$ = [-0.001, 0.019], $ab_{\%}$ = 0.072). Being surrounded by advanced technology (e.g., robots, machines, devices) that is effective at what it does makes me feel safe.	People high in security-societal want to feel protected and safe[196], and hence may overvalue how competent the technology that surrounds them is, given that this makes them feel safe.

Note. DV = dependent variable. For each mediator, we first present its name, followed by its mediated effect (ab) in parentheses, and the item or measure used to assess it.
Predictors: GRP = general risk propensity[191]; IDAQ = anthropomorphism[46]; FMPS_PE = parental expectations[192]; PANAS_TNA = trait negative affect[193]; SD3_P = psychopathy[194]; ERQ_ES = expressive suppression[195]; ATQ_AP = approach temperament[97]; PVQ5X_SS = security-societal[196].
Most mediators, predictors, and DVs were scored on a 1-7 scale (1=Strongly disagree, 7=Strongly agree). The exceptions were 12-PAC mediators, measured on a 1-5 scale (1=Not at all, 5=Extremely); GRP and FMPS_PE, measured on a 1-5 scale (1=Strongly disagree, 5=Strongly agree); IDAQ, measured on a 0-10 scale (0=Not at all, 10=Very much); PANAS_TNA, measured on a 1-5 scale (1=Strongly disagree, 5=Strongly agree); SD3_P, measured on a 1-5 scale (1=Disagree strongly, 5=Agree strongly); and PVQ5X_SS, measured on a 1-6 scale (1=Not like me at all, 6=Very much like me).
The mediated effects are presented in raw units. For example, for GRP_M3 (ab = 0.057, 99% CI = [0.029, 0.093], $ab_{\%}$ = 0.393), the mediated effect ab indicates that for 1 unit increase in GRP as a predictor, the Positive Dimension increased by 0.057 units, which is the effect that can be accounted for by the mediator (GRP_M3). For an easier understanding of the magnitude of each mediated effect, $ab_{\%}$ is also reported and indicates the percentage of the total effect between a predictor and DV (i.e., coefficients b in Supplementary Table 16) that the mediator explained. In some cases, $ab_{\%}$ can exceed 1 (i.e., 100%), which means that the effect that travels through the mediator is larger than the total effect itself. A mediated effect is significant only if its 99% $CI_{bootstrapped}$ does not contain 0[172]. Some mediated effects (ab and $ab_{\%}$) are negative; this means they are in the opposite direction to the effect between a predictor and DV, and therefore did not explain their relationship. All mediators that successfully explained a portion of the relationship between a predictor and DV (i.e., mediated effects that are positive and whose 99% $CI_{bootstrapped}$ does not contain 0) are highlighted in grey.
[a] The two items for GRP_M7 were averaged into a composite score.

[b] For IDAQ as a predictor, we used the same mediators for the positive and negative dimension, considering that we wanted to ensure that any potential differences between the mechanisms for these two dimensions are not a consequence of different mediators being used in the mediation models.

[c] The five 12-PAC mediators capture state affect because they were assessed in relation to how people currently felt.

[d] Although the mediated effect of IDAQ_M3 was significant, the direction of this effect was negative (ab = -0.011) and thus opposite to the positive direction of the relationship between IDAQ and the negative domain (Supplementary Table 16). Therefore, the mediator failed to explain this relationship.

Supplementary Table 18: Skewness and Kurtosis for Items Assessing Psychological Processes Regarding Robots That Were Used in Statistical Analyses in Study 4 (Sample 1)

Variable	Skewness	Kurtosis
Item 1	0.302	-0.630
Item 2	-0.609	-0.064
Item 3	0.731	-0.027
Item 4	-0.991	1.282
Item 5	-1.215	1.685
Item 6	-1.044	1.381
Item 7	-0.534	0.227
Item 8	-0.426	-0.322
Item 9	-0.241	-0.620
Item 10	-0.690	0.293
Item 11	0.730	-0.378
Item 12	0.988	0.267
Item 13	0.871	-0.111
Item 14	-0.14	-0.755
Item 15	-0.435	0.124
Item 16	-0.457	-0.411
Item 17	-0.275	-0.878
Item 18	0.481	-0.510
Item 19	-0.854	0.466
Item 20	-0.800	0.370
Item 21	1.710	2.620
Item 22	1.489	1.539
Item 23	-0.623	-0.256
Item 24	0.037	-1.033
Item 25	0.532	-0.631
Item 26	0.658	-0.592
Item 27	0.726	-0.498
Item 28	-0.663	-0.228
Item 29	-0.480	-0.403
Item 30	1.042	0.374
Item 31	1.177	0.967
Item 32	-0.193	-0.488
Item 33	1.066	0.435
Item 34	1.242	0.650
Item 35	0.539	-0.675
Item 36	-0.926	0.129
Item 37	-0.394	-0.738
Item 38	-0.040	-0.656
Item 39	-0.535	-0.615
Item 40	-0.175	-0.953
Item 41	0.032	-0.780
Item 42	-0.327	-0.599
Item 43	-0.886	0.364

Item 44	-0.665	0.185
Item 45	-0.596	-0.048
Item 46	-0.334	-0.449
Item 47	-0.254	-0.582
Item 48	1.049	0.445
Item 49	1.315	1.252
Item 50	1.331	1.388
Item 51	-0.097	-1.066
Item 52	0.155	-0.781
Item 53	0.497	-0.622
Item 54	-0.083	-0.139
Item 55	-1.112	1.893
Item 56	-0.362	-0.646
Item 57	-0.161	-0.322
Item 58	0.713	-0.087
Item 59	1.063	0.523
Item 60	0.003	-0.705
Item 61	0.261	-1.010
Item 62	-0.251	-0.783
Item 63	-0.328	-0.355
Item 64	-0.425	-0.549
Item 65	-0.308	-0.309
Item 66	-0.114	-0.862
Item 67	-0.159	-0.633
Item 68	-0.614	0.037
Item 69	-0.648	-0.111
Item 70	-0.837	0.350
Item 71	-0.633	-0.241
Item 72	0.781	-0.233
Item 73	0.710	0.171
Item 74	0.938	0.064
Item 75	-0.381	-0.511
Item 76	0.654	-0.397
Item 77	-0.446	0.048
Item 78	1.074	0.582
Item 79	0.659	-0.381
Item 80	-0.659	0.331
Item 81	0.861	0.152
Item 82	-0.688	0.058
Item 83	-0.409	-0.431
Item 84	0.088	-0.930
Item 85	0.252	-0.899
Item 86	-0.713	-0.009
Item 87	-0.540	-0.126
Item 88	0.087	-0.815
Item 89	-0.247	-0.650
Item 90	-0.278	0.152
Item 91	1.504	1.975

Item 92	1.231	0.927
Item 93	-0.848	0.458
Item 94	-0.970	0.902
Item 95	-0.156	-0.275
Item 96	-0.132	-0.549
Item 97	-0.519	-0.102
Item 98	-0.407	-0.603
Item 99	-0.540	-0.505
Item 100	-0.235	-0.695
Item 101	-0.963	1.180
Item 102	1.178	0.695
Item 103	0.737	-0.485
Item 104	0.496	-0.466
Item 105	0.262	-0.594
Item 106	-0.369	-0.192
Item 107	-0.003	-0.702
Item 108	-0.592	0.037
Item 109	-0.657	0.199
Item 110	-0.636	0.447
Item 111	1.170	0.724
Item 112	-0.062	-0.827
Item 113	0.892	0.144
Item 114	0.647	-0.215
Item 115	0.044	-0.846
Item 116	0.361	-0.765
Item 117	-0.066	-1.031
Item 118	-0.346	-0.928
Item 119	0.949	0.272
Item 120	-0.145	-1.079
Item 121	-0.510	-0.083
Item 122	-0.242	-0.933
Item 123	0.068	-1.194
Item 124	-0.391	-0.255
Item 125	-0.273	-0.749
Item 126	0.715	-0.252
Item 127	-0.597	-0.314
Item 128	-0.475	-0.578
Item 129	0.891	-0.017
Item 130	0.636	-0.521
Item 131	0.063	-0.918
Item 132	0.026	-0.785
Item 133	0.591	-0.194
Item 134	0.006	-0.873
Item 135	0.779	-0.020
Item 136	0.975	0.229
Item 137	-0.398	-0.006
Item 138	1.296	1.467
Item 139	-0.305	-0.613

Item 140	0.202	-0.953
Item 141	-0.543	-0.129
Item 142	-0.196	-0.942
Item 143	-0.084	-0.663
Item 144	-0.486	-0.130
Item 145	0.931	0.151
Item 146	-0.749	-0.317
Item 147	0.927	0.224
Item 148	0.809	-0.383
Item 149	1.019	0.144

Note. For more detailed item information, see Supplementary Table 6.

Supplementary Table 19: Skewness and Kurtosis for Items Assessing Psychological Processes Regarding Robots That Were Used in Statistical Analyses in Study 4 (Sample 2)

Variable	Skewness	Kurtosis
Item 1	0.492	-0.583
Item 2	-0.621	-0.100
Item 3	0.730	-0.172
Item 4	-1.077	1.423
Item 5	-1.231	1.637
Item 6	-1.165	1.552
Item 7	-0.707	0.747
Item 8	-0.463	-0.256
Item 9	-0.234	-0.844
Item 10	-0.709	0.168
Item 11	0.750	-0.436
Item 12	1.083	0.392
Item 13	0.756	-0.346
Item 14	-0.173	-0.884
Item 15	-0.458	-0.178
Item 16	-0.530	-0.339
Item 17	-0.166	-0.958
Item 18	0.631	-0.457
Item 19	-0.710	0.188
Item 20	-0.862	0.612
Item 21	1.679	2.211
Item 22	1.524	1.543
Item 23	-0.619	-0.454
Item 24	0.022	-1.136
Item 25	0.654	-0.567
Item 26	0.358	-1.031
Item 27	0.438	-0.96
Item 28	-0.618	-0.458
Item 29	-0.331	-0.707
Item 30	1.156	0.661
Item 31	1.399	1.508
Item 32	-0.197	-0.729
Item 33	1.170	0.760
Item 34	0.846	-0.443
Item 35	0.408	-0.917
Item 36	-0.836	-0.048
Item 37	-0.367	-0.829
Item 38	-0.062	-0.809
Item 39	-0.679	-0.447
Item 40	-0.305	-0.848
Item 41	-0.075	-0.862
Item 42	-0.266	-0.725
Item 43	-0.910	0.263

Item 44	-0.631	0.029
Item 45	-1.043	1.036
Item 46	-0.330	-0.662
Item 47	-0.327	-0.588
Item 48	1.177	0.688
Item 49	1.198	0.763
Item 50	1.425	1.546
Item 51	0.006	-0.843
Item 52	0.149	-0.879
Item 53	0.543	-0.699
Item 54	-0.309	0.101
Item 55	-1.080	1.439
Item 56	-0.488	-0.533
Item 57	-0.234	-0.574
Item 58	0.868	0.065
Item 59	1.222	0.695
Item 60	-0.180	-0.714
Item 61	0.034	-1.057
Item 62	-0.441	-0.640
Item 63	-0.384	-0.413
Item 64	-0.460	-0.668
Item 65	-0.381	-0.188
Item 66	-0.197	-0.882
Item 67	-0.329	-0.422
Item 68	-0.708	0.321
Item 69	-0.767	0.235
Item 70	-0.961	0.611
Item 71	-0.797	-0.014
Item 72	0.933	-0.016
Item 73	0.935	0.293
Item 74	1.030	0.315
Item 75	-0.432	-0.668
Item 76	0.645	-0.401
Item 77	-0.532	-0.101
Item 78	1.020	0.227
Item 79	0.751	-0.393
Item 80	-0.691	0.150
Item 81	0.964	0.129
Item 82	-0.758	0.098
Item 83	-0.492	-0.322
Item 84	-0.113	-0.958
Item 85	0.148	-0.861
Item 86	-0.597	-0.352
Item 87	-0.497	-0.328
Item 88	0.003	-0.869
Item 89	-0.346	-0.678
Item 90	-0.360	0.042
Item 91	1.501	1.757

Item 92	1.226	0.550
Item 93	-0.934	0.722
Item 94	-1.045	0.791
Item 95	-0.219	-0.516
Item 96	-0.237	-0.652
Item 97	-0.637	-0.090
Item 98	-0.583	-0.383
Item 99	-0.755	-0.270
Item 100	-0.173	-0.691
Item 101	-1.121	1.390
Item 102	1.318	1.078
Item 103	0.669	-0.709
Item 104	0.832	0.054
Item 105	0.411	-0.499
Item 106	-0.433	-0.452
Item 107	-0.013	-0.907
Item 108	-0.588	-0.119
Item 109	-0.699	0.174
Item 110	-0.721	0.447
Item 111	1.115	0.481
Item 112	0.023	-0.951
Item 113	0.965	0.020
Item 114	0.723	-0.311
Item 115	0.176	-0.884
Item 116	0.328	-0.748
Item 117	0.024	-1.141
Item 118	-0.237	-1.004
Item 119	1.095	0.507
Item 120	-0.255	-0.974
Item 121	-0.577	-0.015
Item 122	-0.413	-0.671
Item 123	-0.096	-1.204
Item 124	-0.352	-0.200
Item 125	-0.484	-0.681
Item 126	0.745	-0.368
Item 127	-0.629	-0.269
Item 128	-0.547	-0.479
Item 129	0.902	-0.161
Item 130	0.665	-0.566
Item 131	0.195	-0.941
Item 132	-0.053	-0.953
Item 133	0.583	-0.470
Item 134	0.107	-1.041
Item 135	1.010	0.270
Item 136	0.959	-0.068
Item 137	-0.346	-0.339
Item 138	1.219	0.878
Item 139	-0.237	-0.689

Item 140	0.289	-1.014
Item 141	-0.553	-0.271
Item 142	0.023	-1.073
Item 143	-0.073	-0.749
Item 144	-0.674	0.212
Item 145	1.027	0.277
Item 146	-0.807	-0.182
Item 147	1.180	0.780
Item 148	0.861	-0.300
Item 149	0.782	-0.435

Note. For more detailed item information, see Supplementary Table 6.

Supplementary Table 20: Skewness and Kurtosis for the Psychological Responses to Robots (PRR) Scale Items Used in Statistical Analyses in Study 5 (Sample 1)

Variable	Skewness	Kurtosis
PRR Scale Item 2	-0.711	0.107
PRR Scale Item 4	-0.848	1.041
PRR Scale Item 7	-0.366	0.006
PRR Scale Item 8	-0.433	-0.293
PRR Scale Item 13	0.614	-0.486
PRR Scale Item 17	-0.269	-0.920
PRR Scale Item 25	0.520	-0.680
PRR Scale Item 27	0.541	-0.758
PRR Scale Item 32	-0.188	-0.382
PRR Scale Item 35	0.375	-0.835
PRR Scale Item 40	-0.247	-0.708
PRR Scale Item 48	0.864	0.075
PRR Scale Item 52	0.203	-0.772
PRR Scale Item 54	0.061	-0.276
PRR Scale Item 55	-0.942	1.223
PRR Scale Item 59	0.966	0.133
PRR Scale Item 63	-0.277	-0.446
PRR Scale Item 66	-0.196	-0.843
PRR Scale Item 72	0.758	-0.192
PRR Scale Item 76	0.522	-0.467
PRR Scale Item 84	-0.013	-0.983
PRR Scale Item 88	0.024	-0.882
PRR Scale Item 91	1.365	1.386
PRR Scale Item 96	-0.147	-0.616
PRR Scale Item 104	0.501	-0.549
PRR Scale Item 107	0.066	-0.651
PRR Scale Item 113	0.887	0.244
PRR Scale Item 114	0.593	-0.347
PRR Scale Item 115	0.056	-0.887
PRR Scale Item 116	0.279	-0.728
PRR Scale Item 125	-0.458	-0.578
PRR Scale Item 126	0.618	-0.413
PRR Scale Item 132	0.032	-0.833
PRR Scale Item 135	0.629	-0.350
PRR Scale Item 142	-0.199	-0.985
PRR Scale Item 145	0.838	-0.181
PRR Scale Item 149	0.851	-0.258

Note. For more detailed item information, see Supplementary Table 8.

Supplementary Table 21: Skewness and Kurtosis for the Psychological Responses to Robots (PRR) Scale Items Used in Statistical Analyses in Study 5 (Sample 2)

Variable	Skewness	Kurtosis
PRR Scale Item 2	-0.695	0.156
PRR Scale Item 4	-0.951	1.484
PRR Scale Item 7	-0.538	0.449
PRR Scale Item 8	-0.413	-0.100
PRR Scale Item 13	0.707	-0.400
PRR Scale Item 17	-0.355	-0.849
PRR Scale Item 25	0.775	-0.304
PRR Scale Item 27	0.279	-1.015
PRR Scale Item 32	-0.233	-0.234
PRR Scale Item 35	0.434	-0.795
PRR Scale Item 40	-0.450	-0.496
PRR Scale Item 48	1.193	0.857
PRR Scale Item 52	0.306	-0.901
PRR Scale Item 54	-0.122	-0.066
PRR Scale Item 55	-1.104	1.849
PRR Scale Item 59	1.263	0.995
PRR Scale Item 63	-0.457	-0.105
PRR Scale Item 66	-0.304	-0.691
PRR Scale Item 72	1.035	0.494
PRR Scale Item 76	0.622	-0.471
PRR Scale Item 84	-0.116	-0.891
PRR Scale Item 88	-0.038	-0.841
PRR Scale Item 91	1.538	2.231
PRR Scale Item 96	-0.275	-0.567
PRR Scale Item 104	0.862	0.165
PRR Scale Item 107	-0.068	-0.751
PRR Scale Item 113	0.918	0.129
PRR Scale Item 114	0.869	0.233
PRR Scale Item 115	0.308	-0.791
PRR Scale Item 116	0.333	-0.681
PRR Scale Item 125	-0.665	-0.319
PRR Scale Item 126	0.867	0.098
PRR Scale Item 132	-0.073	-0.795
PRR Scale Item 135	0.997	0.400
PRR Scale Item 142	0.104	-1.012
PRR Scale Item 145	1.110	0.590
PRR Scale Item 149	0.891	-0.072

Note. For more detailed item information, see Supplementary Table 8.

Supplementary Table 22: Skewness and Kurtosis for the Psychological Responses to Robots (PRR) Scale Items Used in Measurement Invariance Testing (Study 6)

Variable	Skewness	Kurtosis
PRR Scale Item 2	-0.802	0.268
PRR Scale Item 4	-1.040	1.543
PRR Scale Item 7	-0.668	0.452
PRR Scale Item 8	-0.542	-0.205
PRR Scale Item 13	0.806	-0.231
PRR Scale Item 17	-0.405	-0.787
PRR Scale Item 25	0.800	-0.357
PRR Scale Item 27	0.159	-1.127
PRR Scale Item 32	-0.395	-0.469
PRR Scale Item 35	0.110	-1.143
PRR Scale Item 40	-0.592	-0.507
PRR Scale Item 48	1.241	0.918
PRR Scale Item 52	0.296	-0.927
PRR Scale Item 54	-0.330	-0.104
PRR Scale Item 55	-1.128	1.876
PRR Scale Item 59	1.308	1.124
PRR Scale Item 63	-0.542	-0.247
PRR Scale Item 66	-0.384	-0.794
PRR Scale Item 72	1.094	0.447
PRR Scale Item 76	0.623	-0.561
PRR Scale Item 84	-0.263	-0.915
PRR Scale Item 88	-0.206	-1.041
PRR Scale Item 91	1.735	2.810
PRR Scale Item 96	-0.369	-0.659
PRR Scale Item 104	0.898	0.107
PRR Scale Item 107	-0.214	-0.886
PRR Scale Item 113	1.211	0.763
PRR Scale Item 114	0.906	0.056
PRR Scale Item 115	0.438	-0.795
PRR Scale Item 116	0.205	-1.003
PRR Scale Item 125	-0.827	-0.117
PRR Scale Item 126	1.047	0.374
PRR Scale Item 132	-0.171	-0.910
PRR Scale Item 135	1.067	0.531
PRR Scale Item 142	0.231	-1.068
PRR Scale Item 145	1.189	0.725
PRR Scale Item 149	0.530	-0.868

Note. For more detailed item information, see Supplementary Table 8.

Supplementary Table 23: Skewness and Kurtosis for the Items Used to Examine the Relationships Between the Most Predictive Individual Differences and the Positive-Negative-Competence (PNC) Dimensions (Study 7)

Variable	Skewness	Kurtosis
Positive Dimension	0.324	-0.165
Negative Dimension	1.006	0.992
Competence Dimension	-0.751	0.899
GRP	0.542	-0.634
IDAQ	0.667	0.559
FMPS_PE	-0.037	-0.644
PANAS_TNA	1.825	3.827
SD3_P	0.472	-0.155
ERQ_ES	-0.077	-0.750
ATQ_AP	-0.759	1.079
PVQ5X_SS	-0.321	-0.594

Note. Positive, Negative, and Competence dimension were assessed using the PRR scale (Supplementary Table 8). For clarification of name abbreviations and measurement of the remaining items, see Supplementary Table 11.

Supplementary References

1. Bartneck, C. & Forlizzi, J. A design-centred framework for social human-robot interaction. in *RO-MAN 2004. 13th IEEE international workshop on robot and human interactive communication (IEEE Catalog* 591–594 (IEEE, 2004). doi:10.1109/ROMAN.2004.1374827.
2. Bendel, O. SSML for Sex Robots. in *International Conference on Love and Sex with Robots* 1–11 (Springer, 2017). doi:10.1007/978-3-319-76369-9_1.
3. Broadbent, E. Interactions with robots: The truths we reveal about ourselves. *Annu. Rev. Psychol.* **68**, 627–652 (2017).
4. *Robots and art: Exploring an unlikely symbiosis*. (Springer, 2016).
5. Kamide, H., Takubo, T., Ohara, K., Mae, Y. & Arai, T. Impressions of humanoids: The development of a measure for evaluating a humanoid. *Int. J. Soc. Robot.* **6**, 33–44 (2014).
6. Young, J. E., Hawkins, R., Sharlin, E. & Igarashi, T. Toward acceptable domestic robots: Applying insights from social psychology. *Int. J. Soc. Robot.* **1**, 95–108 (2009).
7. Lo, K.-H. Selling Techno-futurism: Exploring Pepper's Images and Discourses Taiwanese News Media Make. in *Love and Sex with Robots* (eds. Cheok, A. D. & Levy, D.) 83–95 (Springer International Publishing, 2018).
8. IEEE Standard Ontologies for Robotics and Automation. *IEEE Std 1872-2015* 1–60 (2015) doi:10.1109/IEEESTD.2015.7084073.
9. Jackson, J. C., Castelo, N. & Gray, K. Could a rising robot workforce make humans less prejudiced? *Am. Psychol.* **75**, 969–982 (2020).
10. McClure, P. K. You're fired," says the robot: The rise of automation in the workplace, technophobes, and fears of unemployment. *Soc. Sci. Comput. Rev.* **36**, 139–156 (2018).
11. Savela, N., Oksanen, A., Pellert, M. & Garcia, D. Emotional reactions to robot colleagues in a role-playing experiment. *Int. J. Inf. Manag.* **60**, 102361 (2021).
12. Broadbent, E., MacDonald, B., Jago, L., Juergens, M. & Mazharullah, O. Human reactions to good and bad robots. in *2007 IEEE/RSJ International Conference on Intelligent Robots and Systems* 3703–3708 (2007). doi:10.1109/IROS.2007.4398982.
13. Nomura, T., Kanda, T., Suzuki, T. & Kato, K. Prediction of Human Behavior in Human–Robot Interaction Using Psychological Scales for Anxiety and Negative Attitudes Toward Robots. *IEEE Trans. Robot.* **24**, 442–451 (2008).
14. MacDorman, K. F. & Chattopadhyay, D. Reducing consistency in human realism increases the uncanny valley effect; increasing category uncertainty does not. *Cognition* **146**, 190–205 (2016).
15. Stock-Homburg, R. Survey of emotions in human–robot interactions: Perspectives from robotic psychology on 20 years of research. *Int. J. Soc. Robot.* **14**, 389–411 (2021).
16. Bonarini, A., Clasadonte, F., Garzotto, F., Gelsomini, M. & Romero, M. Playful interaction with Teo, a Mobile Robot for Children with Neurodevelopmental Disorders. in *Proceedings of the 7th International Conference on Software Development and Technologies for Enhancing Accessibility and Fighting Info-exclusion* 223–231 (Association for Computing Machinery, 2016). doi:10.1145/3019943.3019976.

17. Liu, S. X., Shen, Q. & Hancock, J. Can a social robot be too warm or too competent? Older Chinese adults' perceptions of social robots and vulnerabilities. *Comput. Hum. Behav.* **125**, 106942 (2021).

18. Shank, D. B., Graves, C., Gott, A., Gamez, P. & Rodriguez, S. Feeling our way to machine minds: People's emotions when perceiving mind in artificial intelligence. *Comput. Hum. Behav.* **98**, 256–266 (2019).

19. Sawabe, T. *et al.* Robot touch with speech boosts positive emotions. *Sci. Rep.* **12**, 1–8 (2022).

20. Smith, E. R., Sherrin, S., Fraune, M. R. & Šabanović, S. Positive emotions, more than anxiety or other negative emotions, predict willingness to interact with robots. *Pers. Soc. Psychol. Bull.* **46**, 1270–1283 (2020).

21. Suzuki, Y., Galli, L., Ikeda, A., Itakura, S. & Kitazaki, M. Measuring empathy for human and robot hand pain using electroencephalography. *Sci. Rep.* **5**, 15924 (2015).

22. Riek, L. D., Rabinowitch, T. C., Chakrabarti, B. & Robinson, P. How anthropomorphism affects empathy toward robots. in *Proceedings of the 4th ACM/IEEE international conference on Human robot interaction* 245–246 (ACM, 2009).

23. Seo, S. H., Geiskkovitch, D., Nakane, M., King, C. & Young, J. E. Poor Thing! Would You Feel Sorry for a Simulated Robot? A comparison of empathy toward a physical and a simulated robot. in *Proceedings of the Tenth Annual ACM/IEEE International Conference on Human-Robot Interaction* 125–132 (Association for Computing Machinery, 2015). doi:10.1145/2696454.2696471.

24. Darling, K., Nandy, P. & Breazeal, C. Empathic concern and the effect of stories in human-robot interaction. in *2015 24th IEEE International Symposium on Robot and Human Interactive Communication (RO-MAN)* 770–775 (2015). doi:10.1109/ROMAN.2015.7333675.

25. You, S. & Robert, L. Emotional attachment, performance, and viability in teams collaborating with embodied physical action (EPA) robots. *J. Assoc. Inf. Syst.* **19**, 377–407 (2017).

26. Weiss, A., Wurhofer, D. & Tscheligi, M. I love this dog"—children's emotional attachment to the robotic dog AIBO. *Int. J. Soc. Robot.* **1**, 243–248 (2009).

27. Döring, N. & Poeschl, S. Love and sex with robots: a content analysis of media representations. *Int. J. Soc. Robot.* **11**, 665–677 (2019).

28. McArthur, N. & Twist, M. L. The rise of digisexuality: Therapeutic challenges and possibilities. *Sex. Relatsh. Ther.* **32**, 334–344 (2017).

29. Szczuka, J. M. & Krämer, N. C. Not only the lonely—how men explicitly and implicitly evaluate the attractiveness of sex robots in comparison to the attractiveness of women, and personal characteristics influencing this evaluation. *Multimodal Technol. Interact.* **1**, 3 (2017).

30. Woodward, S. Digisexuality, erotobotics and the future of intimacy. *N. Z. Sociol.* **35**, 99–119 (2020).

31. Scheunemann, M. M., Cuijpers, R. H. & Salge, C. Warmth and competence to predict human preference of robot behavior in physical human-robot interaction. in *2020 29th IEEE International Conference on Robot and Human Interactive Communication (RO-MAN* 1340–1347 (IEEE, 2020).

32. Spatola, N. *et al.* National stereotypes and robots. in *perception: the "made in" effect. Frontiers in Robotics and AI* vol. 6 21 (2019).

33. Spatola, N. & Urbanska, K. God-like robots: the semantic overlap between representation of divine and artificial entities. *Ai Soc.* **35**, 329–341 (2020).

34. Puntoni, S., Reek, R. W., Giesler, M. & Botti, S. Consumers and artificial intelligence: An experiential perspective. *J. Mark.* **85**, 131–151 (2021).

35. Aymerich-Franch, L. Why it is time to stop ostracizing social robots. *Nat. Mach. Intell.* **2**, 364–364 (2020).

36. Borenstein, J. & Arkin, R. C. Nudging for good: robots and the ethical appropriateness of nurturing empathy and charitable behavior. *Ai Soc.* **32**, 499–507 (2017).

37. Torras, C. Service robots for citizens of the future. *Eur. Rev.* **24**, 17–30 (2016).

38. Zemmar, A., Lozano, A. M. & Nelson, B. J. The rise of robots in surgical environments during COVID-19. *Nat. Mach. Intell.* **2**, 566–572 (2020).

39. de Graaf, M. M. A. & Ben Allouch, S. Exploring influencing variables for the acceptance of social robots. *Robot. Auton. Syst.* **61**, 1476–1486 (2013).

40. Pandey, A., Kaushik, A., Jha, A. K. & Kapse, G. A technological survey on autonomous home cleaning robots. *Int. J. Sci. Res. Publ.* **4**, 1–7 (2014).

41. Ray, C., Mondada, F. & Siegwart, R. What do people expect from robots? in *2008 IEEE/RSJ International Conference on Intelligent Robots and Systems* 3816–3821 (IEEE, 2008).

42. Söderlund, M. Service robots with (perceived) theory of mind: An examination of humans' reactions. *J. Retail. Consum. Serv.* **67**, 102999 (2022).

43. Gnambs, T. & Appel, M. Are robots becoming unpopular? Changes in attitudes towards autonomous robotic systems in Europe. *Comput. Hum. Behav.* **93**, 53–61 (2019).

44. Papadopoulos, I., Koulouglioti, C., Lazzarino, R. & Ali, S. Enablers and barriers to the implementation of socially assistive humanoid robots in health and social care: a systematic review. *BMJ Open* **10**, 033096 (2020).

45. Epley, N., Waytz, A. & Cacioppo, J. T. On seeing human: A three-factor theory of anthropomorphism. *Psychol. Rev.* **114**, 864–886 (2007).

46. Waytz, A., Cacioppo, J. & Epley, N. Who sees human? The stability and importance of individual differences in anthropomorphism. *Perspect. Psychol. Sci.* **5**, 219–232 (2010).

47. Blut, M., Wang, C., Wünderlich, N. V. & Brock, C. Understanding anthropomorphism in service provision: a meta-analysis of physical robots, chatbots, and other AI. *J. Acad. Mark. Sci.* **49**, 632–658 (2021).

48. Damiano, L. & Dumouchel, P. Anthropomorphism in human–robot co-evolution. *Front. Psychol.* **9**, 468 (2018).

49. Yam, K. C. *et al.* Robots at work: People prefer—and forgive—service robots with perceived feelings. *J. Appl. Psychol.* **106**, 1557–1572 (2021).

50. Yam, K. C. *et al.* When your boss is a robot: Workers are more spiteful to robot supervisors that seem more human. *J. Exp. Soc. Psychol.* **102**, 104360 (2022).

51. Gray, H. M., Gray, K. & Wegner, D. M. Dimensions of mind perception. *Science* **315**, 619–619 (2007).

52. Li, Y. & Wang, C. Effect of customer's perception on service robot acceptance. *Int. J. Consum. Stud.* **46**, 1241–1261 (2022).

53. Ötting, S. K., Masjutin, L., Steil, J. J. & Maier, G. W. Let's work together: a meta-analysis on robot design features that enable successful human–robot interaction at work. *Hum. Factors* **0018720820966433**, (2020).

54. Bennett, B. & Daly, A. Recognising rights for robots: Can we? Will we? Should we? *Law Innov. Technol.* **12**, 60–80 (2020).

55. Darling, K. Extending legal protection to social robots: The effects of anthropomorphism, empathy, and violent behavior towards robotic objects. in *Robot Law* 213–232 (Edward Elgar Publishing, 2016).

56. Gordon, J. S. & Pasvenskiene, A. Human rights for robots? A literature review. *AI Ethics* **1**, 579–591 (2021).

57. Gunkel, D. J. The other question: can and should robots have rights? *Ethics Inf. Technol.* **20**, 87–99 (2018).

58. McNally, P. & Inayatullah, S. The rights of robots: Technology, culture and law in the 21st century. *Futures* **20**, 119–136 (1988).

59. Brondi, S., Pivetti, M., Battista, S. & Sarrica, M. What do we expect from robots? Social representations, attitudes and evaluations of robots in daily life. *Technol. Soc.* **66**, 101663 (2021).

60. Szollosy, M. Freud, Frankenstein and our fear of robots: projection in our cultural perception of technology. *Ai Soc.* **32**, 433–439 (2017).

61. Coeckelbergh, M. Can we trust robots? *Ethics Inf. Technol.* **14**, 53–60 (2012).

62. Naneva, S., Sarda Gou, M., Webb, T. L. & Prescott, T. J. A systematic review of attitudes, anxiety, acceptance, and trust towards social robots. *Int. J. Soc. Robot.* **12**, 1179–1201 (2020).

63. Plaks, J. E., Rodriguez, L. B. & Ayad, R. Identifying psychological features of robots that encourage and discourage trust. *Comput. Hum. Behav.* **134**, 107301 (2022).

64. Lutz, C. & Tamò-Larrieux, A. Do privacy concerns about social robots affect use intentions? Evidence from an experimental vignette study. *Front. Robot. AI* **8**, 627958 (2021).

65. Rueben, M. & Smart, W. D. Privacy in human-robot interaction survey and future work. in *We Robot 2016: the Fifth Annual Conference on Legal and Policy Issues relating to Robotics* (University of Miami School of Law, 2016).

66. Vitale, J. *et al.* Be more transparent and users will like you: A robot privacy and user experience design experiment. in *2018 13th ACM/IEEE International Conference on Human-Robot Interaction (HRI* 379–387 (IEEE, 2018). doi:10.1145/3171221.3171269.

67. Birnbaum, G. E. *et al.* Machines as a source of consolation: Robot responsiveness increases human approach behavior and desire for companionship. in *2016 11th ACM/IEEE International Conference on Human-Robot Interaction (HRI* 165–172 (IEEE, 2016). doi:10.1109/HRI.2016.7451748.

68. Elliot, A. J. Approach and avoidance motivation and achievement goals. *Educ. Psychol.* **34**, 169–189 (1999).

69. Elliot, A. J., Gable, S. L. & Mapes, R. R. Approach and avoidance motivation in the social domain. *Pers. Soc. Psychol. Bull.* **32**, 378–391 (2006).

70. Conchinha, C. & Freitas, J. C. Robots & NEE: Learning by playing with robots in an inclusive school setting. in *2015 International Symposium on Computers in Education (SIIE* 86–91 (IEEE, 2015). doi:10.1109/SIIE.2015.7451654.

71. Grau, A., Indri, M., Bello, L. L. & Sauter, T. Robots in industry: The past, present, and future of a growing collaboration with humans. *IEEE Ind. Electron. Mag.* **15**, 50–61 (2020).

72. Brščić, D., Kidokoro, H., Suehiro, Y. & Kanda, T. Escaping from children's abuse of social robots. in *Proceedings of the tenth annual ACM/IEEE international conference on human-robot interaction – HRI'15* 59–66 (ACM Press, 2015). doi:10.1145/2696454.2696468.

73. Nomura, T., Kanda, T., Kidokoro, H., Suehiro, Y. & Yamada, S. Why do children abuse robots? *Interact. Stud.* **17**, 347–369 (2016).

74. Salvini, P. *et al.* How safe are service robots in urban environments? Bullying a robot. in *RO-MAN, 2010 IEEE* 1–7 (IEEE, 2010).

75. Haddadin, S., Albu-Schäffer, A. & Hirzinger, G. Requirements for safe robots: Measurements, analysis and new insights. *Int. J. Robot. Res.* **28**, 1507–1527 (2009).

76. Robla-Gómez, S. *et al.* Working together: A review on safe human-robot collaboration in industrial environments. *IEEE Access* **5**, 26754–26773 (2017).

77. Buhrmester, M. D., Kwang, T. & Gosling, S. D. Amazon's Mechanical Turk: A new source of inexpensive, yet high-quality, data? *Perspect. Psychol. Sci.* **6**, 3–5 (2011).

78. Buhrmester, M. D., Talaifar, S. & Gosling, S. D. An evaluation of Amazon's Mechanical Turk, its rapid rise, and its effective use. *Perspect. Psychol. Sci.* **13**, 149–154 (2018).

79. Casler, K., Bickel, L. & Hackett, E. Separate but equal? A comparison of participants and data gathered via Amazon's Mturk, social media, and face-to-face behavioral testing. *Comput. Hum. Behav.* **29**, 2156–2160 (2013).

80. Hauser, D. J. & Schwarz, N. Attentive Turkers: Mturk participants perform better on online attention checks than do subject pool participants. *Behav. Res. Methods* **48**, 400–407 (2016).

81. Aust, F., Diedenhofen, B., Ullrich, S. & pie, J. Seriousness checks are useful to improve data validity in online research. *Behav. Res. Methods* **45**, 527–535 (2013).

82. Kung, F. Y., Kwok, N. & Brown, D. J. Are attention check questions a threat to scale validity? *Appl. Psychol.* **67**, 264–283 (2018).

83. Meade, A. W. & Craig, S. B. Identifying careless responses in survey data. *Psychol. Methods* **17**, 437–455 (2012).

84. Thomas, K. A. & Clifford, S. Validity and Mechanical Turk: An assessment of exclusion methods and interactive experiments. *Comput. Hum. Behav.* **77**, 184–197 (2017).

85. Storozuk, A., Ashley, M., Delage, V. & Maloney, E. A. Got bots? Practical recommendations to protect online survey data from bot attacks. *Quant. Methods Psychol.* **16**, 472–481 (2020).

86. Elo, S. *et al.* Qualitative content analysis: A focus on trustworthiness. *SAGE Open* **4**, 2158244014522633 (2014).

87. Faulkner, S. L. & Trotter, S. P. Data saturation. in *The international Encyclopedia of Communication Research Methods* 1–2 (2017).

88. Fugard, A. J. B. & Potts, H. W. W. Supporting thinking on sample sizes for thematic analyses: a quantitative tool. *Int. J. Soc. Res. Methodol.* **18**, 669–684 (2015).

89. Guest, G., Namey, E. & Chen, M. A simple method to assess and report thematic saturation in qualitative research. *PLOS ONE* **15**, e0232076 (2020).

90. Hennink, M. & Kaiser, B. N. Sample sizes for saturation in qualitative research: A systematic review of empirical tests. *Soc. Sci. Med.* **292**, 114523 (2022).

91. Mayring, P. Qualitative Content Analysis: Demarcation, Varieties, Developments. *Forum Qual. Sozialforschung Forum Qual. Soc. Res.* **20**, (2019).

92. van Rijnsoever, F. J. (I Can't Get No) Saturation: A simulation and guidelines for sample sizes in qualitative research. *PLOS ONE* **12**, e0181689 (2017).

93. Dalmaijer, E. S., Nord, C. L. & Astle, D. E. Statistical power for cluster analysis. *BMC Bioinformatics* **23**, 1–28 (2022).

94. Parrigon, S., Woo, S. E., Tay, L. & Wang, T. CAPTION-ing the situation: A lexically-derived taxonomy of psychological situation characteristics. *J. Pers. Soc. Psychol.* **112**, 642–681 (2017).

95. Weidman, A. C., Cheng, J. T. & Tracy, J. L. The psychological structure of humility. *J. Pers. Soc. Psychol.* **114**, 153–178 (2018).

96. Maruskin, L. A., Thrash, T. M. & Elliot, A. J. The chills as a psychological construct: Content universe, factor structure, affective composition, elicitors, trait antecedents, and consequences. *J. Pers. Soc. Psychol.* **103**, 135–157 (2012).

97. Elliot, A. J. & Thrash, T. M. Approach and avoidance temperament as basic dimensions of personality. *J. Pers.* **78**, 865–906 (2010).

98. Kaufman, L. & Rousseeuw, P. J. *Finding groups in data: an introduction to cluster analysis*. (John Wiley & Sons, 2005).

99. Nielsen, F. Hierarchical clustering. in *Introduction to HPC with MPI for Data Science* (ed. Nielsen, F.) 195–211 (Springer, 2016).

100. Šulc, Z. & Řezanková, H. Comparison of similarity measures for categorical data in hierarchical clustering. *J. Classif.* **36**, 58–72 (2019).

101. Gower, J. C. A general coefficient of similarity and some of its properties. *Biometrics* **27**, 857–871 (1971).

102. Struyf, A., Hubert, M. & Rousseeuw, P. J. Integrating robust clustering techniques in S-PLUS. *Comput. Stat. Data Anal.* **26**, 17–37 (1997).

103. Murtagh, F. & Contreras, P. Algorithms for hierarchical clustering: an overview. *Wiley Interdiscip. Rev. Data Min. Knowl. Discov.* **2**, 86–97 (2012).

104. Murtagh, F. & Legendre, P. Ward's hierarchical agglomerative clustering method: which algorithms implement Ward's criterion? *J. Classif.* **31**, 274–295 (2014).

105. Schubert, E. & Rousseeuw, P. J. Faster k-medoids clustering: improving the PAM, CLARA, and CLARANS algorithms. in *International conference on similarity search and applications* 171–187 (Springer, 2019).

106. Šulc, Z., Cibulková, J., Procházka, J. & Řezanková, H. Internal evaluation criteria for categorical data in hierarchical clustering: optimal number of clusters determination. *Adv. Methodol. Stat.* **15**, 1–20 (2018).

107. Elo, S. & Kyngäs, H. The qualitative content analysis process. *J. Adv. Nurs.* **62**, 107–115 (2008).

108. Hsieh, H. F. & Shannon, S. E. Three approaches to qualitative content analysis. *Qual. Health Res.* **15**, 1277–1288 (2005).

109. Mayring, P. Qualitative content analysis. *Companion Qual. Res.* **1**, 159–176 (2004).

110. Vaismoradi, M., Turunen, H. & Bondas, T. Content analysis and thematic analysis: Implications for conducting a qualitative descriptive study. *Nurs. Health Sci.* **15**, 398–405 (2013).

111. Mason, M. Sample size and saturation in PhD studies using qualitative interviews. *Forum Qual. SozialforschungForum Qual. Soc. Res.* **11**, (2010).

112. Neale, J. Iterative categorization (IC): a systematic technique for analysing qualitative data. *Addiction* **111**, 1096–1106 (2016).

113. Preston, C. C. & Colman, A. M. Optimal number of response categories in rating scales: reliability, validity, discriminating power, and respondent preferences. *Acta Psychol. (Amst.)* **104**, 1–15 (2000).

114. Simms, L. J., Zelazny, K., Williams, T. F. & Bernstein, L. Does the number of response options matter? Psychometric perspectives using personality questionnaire data. *Psychol. Assess.* **31**, 557–566 (2019).

115. Carlson, M. *et al.* Psychometric properties of reverse-scored items on the CES-D in a sample of ethnically diverse older adults. *Psychol. Assess.* **23**, 558–562 (2011).

116. Ebesutani, C. *et al.* The loneliness questionnaire–short version: An evaluation of reverse-worded and non-reverse-worded items via item response theory. *J. Pers. Assess.* **94**, 427–437 (2012).

117. Wong, N., Rindfleisch, A. & Burroughs, J. E. Do reverse-worded items confound measures in cross-cultural consumer research? The case of the material values scale. *J. Consum. Res.* **30**, 72–91 (2003).

118. Zhang, X. & Savalei, V. Improving the factor structure of psychological scales: The Expanded format as an alternative to the Likert scale format. *Educ. Psychol. Meas.* **76**, 357–386 (2016).

119. Kam, C. C. S. & Meyer, J. P. How careless responding and acquiescence response bias can influence construct dimensionality: The case of job satisfaction. *Organ. Res. Methods* **18**, 512–541 (2015).

120. Costello, A. B. & Osborne, J. W. Best practices in exploratory factor analysis: Four recommendations for getting the most from your analysis. *Pract. Assess. Res. Eval.* **10**, 1–9 (2005).

121. Hogarty, K. Y., Hines, C. V., Kromrey, J. D., Ferron, J. M. & Mumford, K. R. The quality of factor solutions in exploratory factor analysis: The influence of sample size, communality, and overdetermination. *Educ. Psychol. Meas.* **65**, 202–226 (2005).

122. Kyriazos, T. A. Applied psychometrics: sample size and sample power considerations in factor analysis (EFA, CFA) and SEM in general. *Psychology* **9**, 2207 (2018).

123. MacCallum, R. C., Widaman, K. F., Zhang, S. & Hong, S. Sample size in factor analysis. *Psychol. Methods* **4**, 84–99 (1999).

124. Reio Jr., T. G. & Shuck, B. Exploratory factor analysis: Implications for theory, research, and practice. *Adv. Dev. Hum. Resour.* **17**, 12–25 (2015).

125. Everitt, B. S. Multivariate analysis: The need for data, and other problems. *Br. J. Psychiatry* **126**, 237–240 (1975).

126. Gorsuch, R. L. *Factor analysis*. (Erlbaum, 1983).

127. Gorsuch, R. L. *Factor Analysis: Classic Edition*. (Routledge, 2014). doi:10.4324/9781315735740.

128. Gorsuch, R. L. Factor analysis. in *Handbook of psychology: Research methods in psychology* (eds. Schinka, J. A. & Velicer, W. F.) vol. 2 143–164 (Wiley, 2003).

129. Dimitrov, D. M. *Statistical Methods for Validation of Assessment Scale Data in Counseling and Related Fields*. (American Counseling Association, 2012).

130. Guadagnoli, E. & Velicer, W. F. Relation of sample size to the stability of component patterns. *Psychol. Bull.* **103**, 265–275 (1988).

131. Beavers, A. S. *et al.* Practical considerations for using exploratory factor analysis in educational research. *Pract. Assess. Res. Eval.* **18**, 1–13 (2013).

132. Dinno, A. Exploring the sensitivity of Horn's parallel analysis to the distributional form of simulated data. *Multivar. Behav. Res.* **44**, 362–388 (2009).

133. Horn, J. L. A rationale and test for the number of factors in factor analysis. *Psychometrika* **30**, 179–185 (1965).

134. Revelle, W. & Rocklin, T. Very Simple Structure: An Alternative Procedure For Estimating The Optimal Number Of Interpretable Factors. *Multivar. Behav. Res.* **14**, 403–414 (1979).

135. Zwick, W. R. & Velicer, W. F. Comparison of five rules for determining the number of components to retain. *Psychol. Bull.* **99**, 432–442 (1986).

136. Raîche, G., Walls, T. A., Magis, D., Riopel, M. & Blais, J.-G. Non-Graphical Solutions for Cattell's Scree Test. *Methodology* **9**, 23–29 (2013).

137. Luo, L., Arizmendi, C. & Gates, K. M. Exploratory Factor Analysis (EFA) Programs in R. *Struct. Equ. Model. Multidiscip. J.* **26**, 819–826 (2019).

138. Cattell, R. B. The Scree Test For The Number Of Factors. *Multivar. Behav. Res.* **1**, 245–276 (1966).

139. Rauthmann, J. F. *et al.* The Situational Eight DIAMONDS: A taxonomy of major dimensions of situation characteristics. *J. Pers. Soc. Psychol.* **107**, 677–718 (2014).

140. Goretzko, D., Pham, T. T. H. & Bühner, M. Exploratory factor analysis: Current use, methodological developments and recommendations for good practice. *Curr. Psychol.* **40**, 3510–3521 (2021).

141. Schmitt, T. A. & Sass, D. A. Rotation criteria and hypothesis testing for exploratory factor analysis: Implications for factor pattern loadings and interfactor correlations. *Educ. Psychol. Meas.* **71**, 95–113 (2011).

142. Hendrickson, A. E. & White, P. O. Promax: A quick method for rotation to oblique simple structure. *Br. J. Stat. Psychol.* **17**, 65–70 (1964).

143. Kaiser, H. F. The varimax criterion for analytic rotation in factor analysis. *Psychometrika* **23**, 187–200 (1958).

144. Schmitt, T. A., Sass, D. A., Chappelle, W. & Thompson, W. Selecting the "best" factor structure and moving measurement validation forward: An illustration. *J. Pers. Assess.* **100**, 345–362 (2018).

145. Tabachnick, B. G., Fidell, L. S. & Ullman, J. B. *Using multivariate statistics*. (Pearson, 2019).

146. Tabachnick, B. G., Fidell, L. S. & Ullman, J. B. *Using multivariate statistics*. (Pearson, 2007).

147. Hair, J. F., Black, W. C., Babin, B. J. & Anderson, R. E. *Multivariate Data Analysis*. (Prentice Hall, 2010).

148. Hair, J., Black, W., Anderson, R. & Babin, B. *Multivariate Data Analysis*. (Cengage Learning EMEA, 2018).

149. Peterson, R. A. A meta-analysis of variance accounted for and factor loadings in exploratory factor analysis. *Mark. Lett.* **11**, 261–275 (2000).

150. Muthén, L. K. & Muthén, B. O. How to use a Monte Carlo study to decide on sample size and determine power. *Struct. Equ. Model.* **9**, 599–620 (2002).

151. Muthén, L. K. & Muthén, B. O. *Mplus user's guide*. (Muthén & Muthén, 2017).

152. Wang, J. & Wang, X. *Structural equation modeling: Applications using Mplus*. (John Wiley & Sons, 2019).

153. Asparouhov, T. & Muthén, B. Exploratory structural equation modeling. *Struct. Equ. Model. Multidiscip. J.* **16**, 397–438 (2009).

154. Hopwood, C. J. & Donnellan, M. B. How should the internal structure of personality inventories be evaluated? *Personal. Soc. Psychol. Rev.* **14**, 332–346 (2010).

155. Marsh, H. W., Morin, A. J., Parker, P. D. & Kaur, G. Exploratory structural equation modeling: An integration of the best features of exploratory and confirmatory factor analysis. *Annu. Rev. Clin. Psychol.* **10**, 85–110 (2014).

156. Browne, M. W. An overview of analytic rotation in exploratory factor analysis. *Multivar. Behav. Res.* **36**, 111–150 (2001).

157. Xiao, Y., Liu, H. & Hau, K. T. A comparison of CFA, ESEM, and BSEM in test structure analysis. *Struct. Equ. Model. Multidiscip. J.* **26**, 665–677 (2019).

158. Hu, L. T. & Bentler, P. M. Cutoff criteria for fit indexes in covariance structure analysis: Conventional criteria versus new alternatives. *Struct. Equ. Model. Multidiscip. J.* **6**, 1–55 (1999).

159. Hooper, D., Coughlan, J. & Mullen, M. R. Structural equation modelling: Guidelines for determining model fit. *Electron. J. Bus. Res. Methods* **6**, 53–60 (2008).

160. Jackson, D. L., Gillaspy Jr., J. A. & Purc-Stephenson, R. Reporting practices in confirmatory factor analysis: An overview and some recommendations. *Psychol. Methods* **14**, 6–23 (2009).

161. Chen, F. F. Sensitivity of goodness of fit indexes to lack of measurement invariance. *Struct. Equ. Model. Multidiscip. J.* **14**, 464–504 (2007).

162. Song, Q. C., Tang, C. & Wee, S. Making sense of model generalizability: A tutorial on cross-validation in R and Shiny. *Adv. Methods Pract. Psychol. Sci.* **4**, 2515245920947067 (2021).

163. Kuhn, M. Building predictive models in R using the caret package. *J. Stat. Softw.* **28**, 1–26 (2008).

164. Kuhn, M. caret: Classification and Regression Training. (2023).

165. de Rooij, M. & Weeda, W. Cross-Validation: A Method Every Psychologist Should Know. *Adv. Methods Pract. Psychol. Sci.* **3**, 248–263 (2020).

166. Jacobucci, R., Brandmaier, A. M. & Kievit, R. A. A practical guide to variable selection in structural equation modeling by using regularized multiple-indicators, multiple-causes models. *Adv. Methods Pract. Psychol. Sci.* **2**, 55–76 (2019).

167. McNeish, D. M. Using lasso for predictor selection and to assuage overfitting: A method long overlooked in behavioral sciences. *Multivar. Behav. Res.* **50**, 471–484 (2015).

168. Orrù, G., Monaro, M., Conversano, C., Gemignani, A. & Sartori, G. Machine learning in psychometrics and psychological research. *Front. Psychol.* **10**, 2970 (2020).

169. Sheetal, A., Feng, Z. & Savani, K. Using machine learning to generate novel hypotheses: Increasing optimism about COVID-19 makes people less willing to justify unethical behaviors. *Psychol. Sci.* **31**, 1222–1235 (2020).

170. Benjamini, Y. & Hochberg, Y. Controlling the false discovery rate: a practical and powerful approach to multiple testing. *J. R. Stat. Soc. Ser. B Methodol.* **57**, 289–300 (1995).

171. Cabin, R. J. & Mitchell, R. J. To Bonferroni or not to Bonferroni: when and how are the questions. *Bull. Ecol. Soc. Am.* **81**, 246–248 (2000).

172. Hayes, A. F. *Introduction to mediation, moderation, and conditional process analysis: A regression-based approach*. (Guilford Press, 2018).

173. Benjamini, Y. & Yekutieli, D. The control of the false discovery rate in multiple testing under dependency. *Ann. Stat.* **29**, 1165–1188 (2001).

174. Narum, S. R. Beyond Bonferroni: less conservative analyses for conservation genetics. *Conserv. Genet.* **7**, 783–787 (2006).

175. Faul, F., Erdfelder, E., Buchner, A. & Lang, A. G. Statistical power analyses using G* Power 3.1: Tests for correlation and regression analyses. *Behav. Res. Methods* **41**, 1149–1160 (2009).

176. Cohen, J. *Statistical power analysis for the behavioral sciences*. (Lawrence Earlbaum Associates, 1988).

177. Bonarini, A. & Besio, S. *Robot Play for All: Developing Toys and Games for Disability*. (Springer International Publishing, 2022). doi:10.1007/978-3-031-05042-8.

178. Bonarini, A. Can my robotic home cleaner be happy? Issues about emotional expression in non-bio-inspired robots. *Adapt. Behav.* **24**, 335–349 (2016).

179. Levy, D. *Love and Sex with Robots: The Evolution of Human-Robot Relationships*. (Harper Collins, 2007).

180. Sullivan, A. & Bers, M. U. Dancing robots: integrating art, music, and robotics in Singapore's early childhood centers. *Int. J. Technol. Des. Educ.* **28**, 325–346 (2018).

181. Westfall, J., Kenny, D. A. & Judd, C. M. Statistical power and optimal design in experiments in which samples of participants respond to samples of stimuli. *J. Exp. Psychol. Gen.* **143**, 2020–2045 (2014).

182. Westfall, J., Judd, C. M. & Kenny, D. A. Replicating studies in which samples of participants respond to samples of stimuli. *Perspect. Psychol. Sci.* **10**, 390–399 (2015).

183. Alamer, A. Exploratory structural equation modeling (ESEM) and bifactor ESEM for construct validation purposes: Guidelines and applied example. *Res. Methods Appl. Linguist.* **1**, 100005 (2022).

184. van Zyl, L. E. & ten Klooster, P. M. Exploratory Structural Equation Modeling: Practical Guidelines and Tutorial With a Convenient Online Tool for Mplus. *Front. Psychiatry* **12**, (2022).

185. Rodriguez, A., Reise, S. P. & Haviland, M. G. Applying bifactor statistical indices in the evaluation of psychological measures. *J. Pers. Assess.* **98**, 223–237 (2016).

186. Rodriguez, A., Reise, S. P. & Haviland, M. G. Evaluating bifactor models: Calculating and interpreting statistical indices. *Psychol. Methods* **21**, 137–150 (2016).

187. Dueber, D. Bifactor Indices Calculator: A Microsoft Excel-Based Tool to Calculate Various Indices Relevant to Bifactor CFA Models. (2017).

188. Dueber, D. M. & Toland, M. D. A bifactor approach to subscore assessment. *Psychol. Methods* **28**, 222–241 (2023).

189. Chen, F. F. What happens if we compare chopsticks with forks? The impact of making inappropriate comparisons in cross-cultural research. *J. Pers. Soc. Psychol.* **95**, 1005–1018 (2008).

190. Putnick, D. L. & Bornstein, M. H. Measurement invariance conventions and reporting: the state of the art and future directions for psychological research. *Dev. Rev.* **41**, 71–90 (2016).

191. Zhang, D. C., Highhouse, S. & Nye, C. D. Development and validation of the general risk propensity scale (GriPS. *J. Behav. Decis. Mak.* **32**, 152–167 (2019).

192. Frost, R. O., Marten, P., Lahart, C. & Rosenblate, R. The dimensions of perfectionism. *Cogn. Ther. Res.* **14**, 449–468 (1990).

193. Watson, D., Clark, L. A. & Tellegen, A. Development and validation of brief measures of positive and negative affect: the PANAS scales. *J. Pers. Soc. Psychol.* **54**, 1063–1070 (1988).

194. Jones, D. N. & Paulhus, D. L. Introducing the short dark triad (SD3) a brief measure of dark personality traits. *Assessment* **21**, 28–41 (2014).

195. Gross, J. J. & John, O. P. Individual differences in two emotion regulation processes: Implications for affect, relationships, and well-being. *J. Pers. Soc. Psychol.* **85**, 348–362 (2003).

196. Schwartz, S. H. *et al.* Refining the theory of basic individual values. *J. Pers. Soc. Psychol.* **103**, 663–688 (2012).

197. Yik, M., Russell, J. A. & Steiger, J. H. A 12-point circumplex structure of core affect. *Emotion* **11**, 705–731 (2011).

198. Kirkpatrick, L. A. & Feeney, B. C. *A simple guide to IBM SPSS statistics for version 23.0*. (Cengage Learning, 2015).

199. MacKinnon, D. P., Fairchild, A. J. & Fritz, M. S. Mediation analysis. *Annu. Rev. Psychol.* **58**, 593–614 (2007).

200. Schmitt, T. A. Current methodological considerations in exploratory and confirmatory factor analysis. *J. Psychoeduc. Assess.* **29**, 304–321 (2011).

201. Locke, E. A. The case for inductive theory building. *J. Manag.* **33**, 867–890 (2007).

202. ISO 8373:2021 Robotics — Vocabulary. (2021).

203. Bartneck, C., Kulić, D., Croft, E. & Zoghbi, S. Measurement instruments for the anthropomorphism, animacy, likeability, perceived intelligence, and perceived safety of robots. *Int. J. Soc. Robot.* **1**, 71–81 (2009).

204. Carpinella, C. M., Wyman, A. B., Perez, M. A. & Stroessner, S. J. The Robotic Social Attributes Scale (RoSAS): Development and Validation. in *Proceedings of the 2017 ACM/IEEE International Conference on Human-Robot Interaction* 254–262 (Association for Computing Machinery, 2017). doi:10.1145/2909824.3020208.

205. Mori, M., MacDorman, K. F. & Kageki, N. The uncanny valley [from the field. *IEEE Robot. Autom. Mag.* **19**, 98–100 (2012).

206. Lischetzke, T., Izydorczyk, D., Hüller, C. & Appel, M. The topography of the uncanny valley and individuals' need for structure: A nonlinear mixed effects analysis. *J. Res. Personal.* **68**, 96–113 (2017).

207. Cheetham, M., Suter, P. & Jäncke, L. The Human Likeness Dimension of the "Uncanny Valley Hypothesis": Behavioral and Functional MRI Findings. *Front. Hum. Neurosci.* **5**, (2011).

208. Bartneck, C., Kanda, T., Ishiguro, H. & Hagita, N. My robotic doppelgänger - a critical look at the Uncanny Valley. in *RO-MAN 2009 - The 18th IEEE International Symposium on Robot and Human Interactive Communication* 269–276 (2009). doi:10.1109/ROMAN.2009.5326351.

209. Fiske, S. T., Cuddy, A. J. & Glick, P. Universal dimensions of social cognition: Warmth and competence. *Trends Cogn. Sci.* **11**, 77–83 (2007).

210. Cuddy, A. J. C., Fiske, S. T. & Glick, P. Warmth and Competence as Universal Dimensions of Social Perception: The Stereotype Content Model and the BIAS Map. in *Advances in Experimental Social Psychology* vol. 40 61–149 (Academic Press, 2008).

211. Reeves, B. & Nass, C. I. *The media equation: How people treat computers, television, and new media like real people and places.* xiv, 305 (Cambridge University Press, 1996).

212. Nass, C., Steuer, J. & Tauber, E. R. Computers Are Social Actors. in *Proceedings of the SIGCHI Conference on Human Factors in Computing Systems* 72–78 (Association for Computing Machinery, 1994). doi:10.1145/191666.191703.

213. Gambino, A., Fox, J. & Ratan, R. A. Building a stronger CASA: Extending the computers are social actors paradigm. *Hum.-Mach. Commun.* **1**, 71–85 (2020).

214. Clark, H. H. & Fischer, K. Social robots as depictions of social agents. *Behav. Brain Sci.* **46**, e21 (2023).

215. Esterwood, C., Essenmacher, K., Yang, H., Zeng, F. & Robert, L. P. A Meta-Analysis of Human Personality and Robot Acceptance in Human-Robot Interaction. in *Proceedings of the 2021 CHI Conference on Human Factors in Computing Systems* 1–18 (2021). doi:10.1145/3411764.3445542.

216. Morsunbul, U. Human-robot interaction: How do personality traits affect attitudes towards robot? *J. Hum. Sci.* **16**, 499–504 (2019).

217. Robert Jr., L. P. *et al.* A Review of Personality in Human–Robot Interactions. *Found. Trends® Inf. Syst.* **4**, 107–212 (2020).

218. Reich, N. & Eyssel, F. Attitudes towards service robots in domestic environments: The role of personality characteristics, individual interests, and demographic variables. *Paladyn J. Behav. Robot.* **4**, 123–130 (2013).

219. Nicolas, S. & Agnieszka, W. The personality of anthropomorphism: How the need for cognition and the need for closure define attitudes and anthropomorphic attributions toward robots. *Comput. Hum. Behav.* **122**, 106841 (2021).

220. MacDorman, K. F. & Entezari, S. O. Individual differences predict sensitivity to the uncanny valley. *Interact. Stud.* **16**, 141–172 (2015).

221. Jack, R. E., Crivelli, C. & Wheatley, T. Data-driven methods to diversify knowledge of human psychology. *Trends Cogn. Sci.* **22**, 1–5 (2018).

222. Paetzel-Prüsmann, M., Perugia, G. & Castellano, G. The Influence of robot personality on the development of uncanny feelings. *Comput. Hum. Behav.* **120**, 106756 (2021).

223. R. Wullenkord, M. R. Fraune, F. Eyssel, & S. Šabanović. Getting in Touch: How imagined, actual, and physical contact affect evaluations of robots. in *2016 25th IEEE International Symposium on Robot and Human Interactive Communication (RO-MAN)* 980–985 (2016). doi:10.1109/ROMAN.2016.7745228.

224. Willig, C. What can qualitative psychology contribute to psychological knowledge? *Psychol. Methods* **24**, 796–804 (2019).

225. Henrich, J., Heine, S. J. & Norenzayan, A. Most people are not WEIRD. *Nature* **466**, 29–29 (2010).

226. Hofstede, G. Dimensionalizing Cultures: The Hofstede Model in Context. *Online Read. Psychol. Cult.* **2**, (2011).

227. Fiedler, K., Harris, C. & Schott, M. Unwarranted inferences from statistical mediation tests–An analysis of articles published in 2015. *J. Exp. Soc. Psychol.* **75**, 95–102 (2018).

228. Yzerbyt, V., Muller, D., Batailler, C. & Judd, C. M. New recommendations for testing indirect effects in mediational models: The need to report and test component paths. *J. Pers. Soc. Psychol.* **115**, 929–943 (2018).

229. Boyce, C. J., Wood, A. M., Daly, M. & Sedikides, C. Personality change following unemployment. *J. Appl. Psychol.* **100**, 991–1011 (2015).

230. Leger, K. A., Turiano, N. A., Bowling, W., Burris, J. L. & Almeida, D. M. Personality traits predict long-term physical health via affect reactivity to daily stressors. *Psychol. Sci.* **32**, 755–765 (2021).

231. Newman, A., Bavik, Y. L., Mount, M. & Shao, B. Data Collection via Online Platforms: Challenges and Recommendations for Future Research. *Appl. Psychol.* **70**, 1380–1402 (2021).

232. Lovakov, A. & Agadullina, E. R. Empirically derived guidelines for effect size interpretation in social psychology. *Eur. J. Soc. Psychol.* **51**, 485–504 (2021).

233. Funder, D. C. & Ozer, D. J. Evaluating Effect Size in Psychological Research: Sense and Nonsense. *Adv. Methods Pract. Psychol. Sci.* **2**, 156–168 (2019).

234. Liu, W. & Yao, M. Gender identity and influence in human-machine communication:A mixed-methods exploration. *Comput. Hum. Behav.* **144**, 107750 (2023).

235. Fortunati, L., Edwards, A., Edwards, C., Manganelli, A. M. & de Luca, F. Is Alexa female, male, or neutral? A cross-national and cross-gender comparison of perceptions of Alexa's gender and status as a communicator. *Comput. Hum. Behav.* **137**, 107426 (2022).

236. Neel, R., Kenrick, D. T., White, A. E. & Neuberg, S. L. Individual differences in fundamental social motives. *J. Pers. Soc. Psychol.* **110**, 887 (2016).

237. F. Eyssel & N. Reich. Loneliness makes the heart grow fonder (of robots) — On the effects of loneliness on psychological anthropomorphism. in *2013 8th ACM/IEEE International Conference on Human-Robot Interaction (HRI)* 121–122 (2013). doi:10.1109/HRI.2013.6483531.

238. Li, S., Yu, F. & Peng, K. Effect of State Loneliness on Robot Anthropomorphism: Potential Edge of Social Robots Compared to Common Nonhumans. *J. Phys. Conf. Ser.* **1631**, 012024 (2020).

239. Odekerken-Schröder, G., Mele, C., Russo-Spena, T., Mahr, D. & Ruggiero, A. Mitigating loneliness with companion robots in the COVID-19 pandemic and beyond: an integrative framework and research agenda. *J. Serv. Manag.* **31**, 1149–1162 (2020).

240. Lang, F. R., John, D., Lüdtke, O., Schupp, J. & Wagner, G. G. Short assessment of the Big Five: Robust across survey methods except telephone interviewing. *Behav. Res. Methods* **43**, 548–567 (2011).

241. Buss, A. H. & Perry, M. The aggression questionnaire. *J. Pers. Soc. Psychol.* **63**, 452–459 (1992).

242. Olatunji, B. O. *et al.* The Disgust Scale: item analysis, factor structure, and suggestions for refinement. *Psychol. Assess.* **19**, 281 (2007).

243. Elliot, A. J. & Thrash, T. M. Approach-avoidance motivation in personality: Approach and avoidance temperaments and goals. *J. Pers. Soc. Psychol.* **82**, 804–818 (2002).

244. Lin, X., Lu, L., Ozer, M. & Tang, H. Am I motivated to share knowledge for better innovative performance? An approach and avoidance framework. *J. Appl. Psychol.* **108**, 138–151 (2023).

245. Chen, B. *et al.* Basic psychological need satisfaction, need frustration, and need strength across four cultures. *Motiv. Emot.* **39**, 216–236 (2015).

246. Lee, Y., Lee, J. & Hwang, Y. Relating motivation to information and communication technology acceptance: Self-determination theory perspective. *Comput. Hum. Behav.* **51**, 418–428 (2015).

247. Hoyle, R. H., Stephenson, M. T., Palmgreen, P., Lorch, E. P. & Donohew, R. L. Reliability and validity of a brief measure of sensation seeking. *Personal. Individ. Differ.* **32**, 401–414 (2002).

248. Ho, A. K. *et al.* The nature of social dominance orientation: Theorizing and measuring preferences for intergroup inequality using the new SDO7 scale. *J. Pers. Soc. Psychol.* **109**, 1003–1028 (2015).

249. Klimstra, T. A., Hale III, W. W., Raaijmakers, Q. A. W., Branje, S. J. T. & Meeus, W. H. J. A developmental typology of adolescent personality. *Eur. J. Personal.* **24**, 309–323 (2010).

250. Shipp, A. J., Edwards, J. R. & Lambert, L. S. Conceptualization and measurement of temporal focus: The subjective experience of the past, present, and future. *Organ. Behav. Hum. Decis. Process.* **110**, 1–22 (2009).

251. Davis, M. H. A multidimensional approach to individual differences in empathy. *Cat. Sel. Doc. Psychol.* **10**, 85 (1980).

252. Denning, T., Matuszek, C., Koscher, K., Smith, J. R. & Kohno, T. A Spotlight on Security and Privacy Risks with Future Household Robots: Attacks and Lessons. in *Proceedings of the 11th International Conference on Ubiquitous Computing* 105–114 (Association for Computing Machinery, 2009). doi:10.1145/1620545.1620564.

253. Paolillo, A. *et al.* How to compete with robots by assessing job automation risks and resilient alternatives. *Sci. Robot.* **7**, eabg5561.

254. Chen, G., Gully, S. M. & Eden, D. Validation of a new general self-efficacy scale. *Organ. Res. Methods* **4**, 62–83 (2001).

255. Ashton, M. C. & Lee, K. The HEXACO–60: A short measure of the major dimensions of personality. *J. Pers. Assess.* **91**, 340–345 (2009).

256. Hale, W. *et al.* Resolving uncertainty about the Intolerance of Uncertainty Scale–12: Application of modern psychometric strategies. *J. Pers. Assess.* **98**, 200–208 (2016).

257. Munde, S. *Robotics market research report: Forecast till 2030*. https://www.marketresearchfuture.com/reports/robotics-market/toc (2021).

258. Smith, A. & Anderson, J. AI, Robotics, and the Future of Jobs. *Pew Res. Cent.* (2014).

259. Koenig, H. G. & Büssing, A. The Duke University Religion Index (DUREL): a five-item measure for use in pielberger cal studies. *Religions* **1**, 78–85 (2010).

260. Scheier, M. F., Carver, C. S. & Bridges, M. W. Distinguishing optimism from neuroticism (and trait anxiety, self-mastery, and self-esteem): a reevaluation of the Life Orientation Test. *J. Pers. Soc. Psychol.* **67**, 1063–1078 (1994).

261. Cheung, F. & Lucas, R. E. Assessing the validity of single-item life satisfaction measures: Results from three large samples. *Qual. Life Res.* **23**, 2809–2818 (2014).

262. Rojas, M. Life satisfaction and satisfaction in domains of life: Is it a simple relationship? *J. Happiness Stud. Interdiscip. Forum Subj. Well-Being* **7**, 467–497 (2006).

263. Harris, M. A., Donnellan, M. B. & Trzesniewski, K. H. The Lifespan Self-Esteem Scale: Initial validation of a new measure of global self-esteem. *J. Pers. Assess.* **100**, 84–95 (2018).

264. Levenson, H. Differentiating among internality, powerful others, and chance. in *Research with the locus of control construct* (ed. Lefcourt, H. M.) vol. 1 15–63 (Academic Press, 1981).

265. Lins de Holanda Coelho, G., H. P. Hanel, P. & J. Wolf, L. The Very Efficient Assessment of Need for Cognition: Developing a Six-Item Version. *Assessment* **27**, 1870–1885 (2020).

266. Nomura, T., Suzuki, T., Kanda, T. & Kato, K. Measurement of negative attitudes toward robots. *Interact. Stud. Soc. Behav. Commun. Biol. Artif. Syst.* **7**, 437–454 (2006).

267. Gerbasi, M. E. & Prentice, D. A. The self-and other-interest inventory. *J. Pers. Soc. Psychol.* **105**, 495–514 (2013).

268. Snyder, M. & Gangestad, S. On the nature of self-monitoring: Matters of assessment, matters of validity. *J. Pers. Soc. Psychol.* **51**, 125–139 (1986).

269. Gibbons, F. X. & Buunk, B. P. Individual differences in social comparison: Development of a scale of social comparison orientation. *J. Pers. Soc. Psychol.* **76**, 129–142 (1999).

270. Zsido, A. N., Teleki, S. A., Csokasi, K., Rozsa, S. & Bandi, S. A. Development of the short version of the pielberger state—trait anxiety inventory. *Psychiatry Res.* **291**, 113223 (2020).

271. Hirsh, J. B., Mar, R. A. & Peterson, J. B. Psychological entropy: A framework for understanding uncertainty-related anxiety. *Psychol. Rev.* **119**, 304–320 (2012).

272. Chien, S.-E. *et al.* Age Difference in Perceived Ease of Use, Curiosity, and Implicit Negative Attitude toward Robots. *J Hum-Robot Interact* **8**, (2019).

273. Schweinberger, S. R., Pohl, M. & Winkler, P. Autistic traits, personality, and evaluations of humanoid robots by young and older adults. *Comput. Hum. Behav.* **106**, 106256 (2020).

274. Ghimire, R., Skinner, J. & Carnathan, M. Who perceived automation as a threat to their jobs in metro Atlanta: Results from the 2019 Metro Atlanta Speaks survey. *Technol. Soc.* **63**, 101368 (2020).

275. Graham, J., Haidt, J. & Nosek, B. A. Liberals and conservatives rely on different sets of moral foundations. *J. Pers. Soc. Psychol.* **96**, 1029–1046 (2009).

276. Castelo, N. & Ward, A. F. Conservatism predicts aversion to consequential Artificial Intelligence. *PLOS ONE* **16**, e0261467 (2021).

277. Imamoğlu, Ç. & Imamoğlu, E. O. Relationship between Familiarity, Attitudes and Preferences: Assisted Living Facilities as Compared to Nursing Homes. *Soc. Indic. Res.* **79**, 235–254 (2006).

278. Rindfleisch, A. & Inman, J. Explaining the Familiarity-Liking Relationship: Mere Exposure, Information Availability, or Social Desirability? *Mark. Lett.* **9**, 5–19 (1998).

279. Bohner, G. & Dickel, N. Attitudes and Attitude Change. *Annu. Rev. Psychol.* **62**, 391–417 (2011).

280. Miller, D. T. & Prentice, D. A. Changing Norms to Change Behavior. *Annu. Rev. Psychol.* **67**, 339–361 (2016).

281. Horvath, P. & Zuckerman, M. Sensation seeking, risk appraisal, and risky behavior. *Personal. Individ. Differ.* **14**, 41–52 (1993).

282. Reniers, R. L. E. P., Murphy, L., Lin, A., Bartolomé, S. P. & Wood, S. J. Risk Perception and Risk-Taking Behaviour during Adolescence: The Influence of Personality and Gender. *PLOS ONE* **11**, e0153842 (2016).

283. FitzGibbon, L., Lau, J. K. L. & Murayama, K. The seductive lure of curiosity: information as a motivationally salient reward. *Curiosity Explore Vs Exploit* **35**, 21–27 (2020).

284. Hamstra, M. R. W., Bolderdijk, J. W. & Veldstra, J. L. Everyday risk taking as a function of regulatory focus. *J. Res. Personal.* **45**, 134–137 (2011).

285. To, C., Kilduff, G. J., Ordoñez, L. & Schweitzer, M. E. Going for it on Fourth Down: Rivalry Increases Risk Taking, Physiological Arousal, and Promotion Focus. *Acad. Manage. J.* **61**, 1281–1306 (2018).

286. Higgins, E. T. Promotion and Prevention: Regulatory Focus as A Motivational Principle. in *Advances in Experimental Social Psychology* (ed. Zanna, M. P.) vol. 30 1–46 (Academic Press, 1998).

287. Larsen, R. J. & Diener, E. Affect intensity as an individual difference characteristic: A review. *J. Res. Personal.* **21**, 1–39 (1987).

288. Wan, E. W. & Chen, R. P. Anthropomorphism and object attachment. *Object Attach.* **39**, 88–93 (2021).

289. Maio, G. R. & Esses, V. M. The Need for Affect: Individual Differences in the Motivation to Approach or Avoid Emotions. *J. Pers.* **69**, 583–614 (2001).

290. Duffy, B. R. Anthropomorphism and the social robot. *Socially Interact. Robots* **42**, 177–190 (2003).

291. Broadbent, E. *et al.* Robots with Display Screens: A Robot with a More Humanlike Face Display Is Perceived To Have More Mind and a Better Personality. *PLOS ONE* **8**, e72589 (2013).

292. Stoeber, J. How Other-Oriented Perfectionism Differs from Self-Oriented and Socially Prescribed Perfectionism: Further Findings. *J. Psychopathol. Behav. Assess.* **37**, 611–623 (2015).

293. Çimşir, E. & Ülker Tümlü, G. The roles of latent perfectionism classes in academicians' tendencies toward workaholism, useless superiority effort and narcissism. *J. Gen. Psychol.* **149**, 524–549 (2022).

294. Curran, T. & Hill, A. P. Young people's perceptions of their parents' expectations and criticism are increasing over time: Implications for perfectionism. *Psychol. Bull.* **148**, 107–128 (2022).

295. Garofalo, C., Neumann, C. S., Zeigler-Hill, V. & Meloy, J. R. Spiteful and contemptuous: A new look at the emotional experiences related to psychopathy. *Personal. Disord. Theory Res. Treat.* **10**, 173–184 (2019).

296. Fanti, K. A., Kyranides, M. N., Drislane, L. E., Colins, O. F. & Andershed, H. Validation of the Greek Cypriot translation of the Triarchic Psychopathy Measure. *J. Pers. Assess.* **98**, 146–154 (2016).

297. Palmen, D. G. C., Kolthoff, E. W. & Derksen, J. J. L. The need for domination in psychopathic leadership: A clarification for the estimated high prevalence of psychopathic leaders. *Aggress. Violent Behav.* **61**, (2021).

298. Porter, S., ten Brinke, L., Baker, A. & Wallace, B. Would I lie to you? "Leakage" in deceptive facial expressions relates to psychopathy and emotional intelligence. *Personal. Individ. Differ.* **51**, 133–137 (2011).

299. D'Mello, S., Kappas, A. & Gratch, J. The Affective Computing Approach to Affect Measurement. *Emot. Rev.* **10**, 174–183 (2018).

300. Hinds, J. & Joinson, A. Human and computer personality prediction from digital footprints. *Curr. Dir. Psychol. Sci.* **28**, 204–211 (2019).

301. Love, A. B. & Holder, M. D. Psychopathy and subjective well-being. *Personal. Individ. Differ.* **66**, 112–117 (2014).

302. Muraven, M., Tice, D. M. & Baumeister, R. F. Self-control as a limited resource: Regulatory depletion patterns. *J. Pers. Soc. Psychol.* **74**, 774–789 (1998).

303. Johns, M., Inzlicht, M. & Schmader, T. Stereotype threat and executive resource depletion: Examining the influence of emotion regulation. *J. Exp. Psychol. Gen.* **137**, 691–705 (2008).

304. Elliot, A. J., Murayama, K. & Pekrun, R. A 3 × 2 achievement goal model. *J. Educ. Psychol.* **103**, 632–648 (2011).

305. Elliot, A. J., Shell, M. M., Henry, K. B. & Maier, M. A. Achievement Goals, Performance Contingencies, and Performance Attainment: An Experimental Test. *J. Educ. Psychol.* **97**, 630–640 (2005).
