# Supplementary material for: The positive–negative–competence (PNC) model of psychological responses to representations of robots
Source: Nat Hum Behav. 2023 Oct 2;7(11):1933–54. doi: 10.1038/s41562-023-01705-7 (PMC10663151; doi:10.1038/s41562-023-01705-7)
Supplement: Supplementary file 2 — Reporting Summary [file 41562_2023_1705_MOESM2_ESM.pdf]

nature portfolio

Corresponding author(s): Dario Krpan

Last updated by author(s): 21 August 2023

Reporting Summary

Nature Portfolio wishes to improve the reproducibility of the work that we publish. This form provides structure for consistency and transparency in reporting. For further information on Nature Portfolio policies, see our Editorial Policies and the Editorial Policy Checklist.

Statistics

For all statistical analyses, confirm that the following items are present in the figure legend, table legend, main text, or Methods section.

n/a	Confirmed	
☐	☒	The exact sample size (*n*) for each experimental group/condition, given as a discrete number and unit of measurement
☐	☒	A statement on whether measurements were taken from distinct samples or whether the same sample was measured repeatedly
☐	☒	The statistical test(s) used AND whether they are one- or two-sided<br>*Only common tests should be described solely by name; describe more complex techniques in the Methods section.*
☐	☒	A description of all covariates tested
☐	☒	A description of any assumptions or corrections, such as tests of normality and adjustment for multiple comparisons
☐	☒	A full description of the statistical parameters including central tendency (e.g. means) or other basic estimates (e.g. regression coefficient) AND variation (e.g. standard deviation) or associated estimates of uncertainty (e.g. confidence intervals)
☐	☒	For null hypothesis testing, the test statistic (e.g. *F*, *t*, *r*) with confidence intervals, effect sizes, degrees of freedom and *P* value noted<br>*Give P values as exact values whenever suitable.*
☒	☐	For Bayesian analysis, information on the choice of priors and Markov chain Monte Carlo settings
☒	☐	For hierarchical and complex designs, identification of the appropriate level for tests and full reporting of outcomes
☐	☒	Estimates of effect sizes (e.g. Cohen's *d*, Pearson's *r*), indicating how they were calculated

Our web collection on statistics for biologists contains articles on many of the points above.

Software and code

Policy information about availability of computer code

Data collection	Data for all studies were collected using Qualtrics (https://www.qualtrics.com/). The following Qualtrics versions were used: Study 1 (Sample 1) - version [February, March 2019]; Study 1 (Sample 2) - version [July 2019]; Study 2 - version [September 2019]; Study 3 - version [October 2019]; Study 4 (Sample 1) - version [March 2021]; Study 4 (Sample 2) - version [April 2021]; Study 5 (Sample 1) - version [June, July, 2021]; Study 5 (Sample 2) - version [June, July 2021]; Study 6 - version [September, October, 2021]; Study 7 - version [December 2021].<br>The original surveys in Qualtrics that were used for data collection are available via the following link (for each study, check the folder "Materials"): https://osf.io/2ntdy/?view_only=2cacc7b1cf2141cf8c343f3ee28dab1d<br>Qualtrics is a commercial survey platform, and we did not use any of our own code to collect the data.
Data analysis	The data were analyzed using the following software (and packages where relevant):<br>Study 1 (Sample 2) - R software (version 4.2.1): packages dplyr (version 1.1.1), cluster (version 2.1.3), dendextend (version 1.16.0), and ape (version 5.6-2).<br>Study 4 (Samples 1 and 2) - R software (version 4.2.1): packages psych (version 2.2.5), paran (version 1.5.2), nFactors (version 2.4.1.1), GPArotation (version 2023.3-1), and MVN (version 5.9).<br>Study 5 (Samples 1 and 2) - Mplus (version 8.6); R software (version 4.2.1): packages psych (version 2.2.5) and MVN (version 5.9); Bifactor Indices Calculator (version 10-4-2017).<br>Study 6 - R software (version 4.2.1): packages psych (version 2.2.5), caret (version 6.0-93), tidyverse (version 1.3.2), rsample (version 1.1.0), skimr (version 2.1.4), ggplot2 (version 3.4.2), ggthemes (version 4.2.4), ggpubr (version 0.4.0), glmnet (version 4.1-4), party (version 1.3-11), randomForest (version 4.7-1.1), forecast (version 8.18), fabletools (version 0.3.2), h2o (version 3.38.0.1), and MVN (version 5.9); Mplus (version 8.6).<br>Study 7 - R software (version 4.2.1): packages psych (version 2.2.5), sensemakr (version 0.1.4), sjPlot (version 2.8.14), and MVN (version 5.9); SPSS (version 23): package Process (version 3.4.1).

For more information about R software, see https://www.r-project.org/; for more information about Mplus, see https://www.statmodel.com/; for more information about SPSS, see https://www.ibm.com/products/spss-statistics; and for more information about Bifactor Indices Calculator, see https://uknowledge.uky.edu/edp_tools/1/
Studies 1 (Sample 1), 2, and 3 involved only qualitative analyses, and therefore no statistical software was used in these studies. All analyses codes are available via the following link: https://osf.io/2ntdy/?view_only=2cacc7b1cf2141cf8c343f3ee28dab1d

For manuscripts utilizing custom algorithms or software that are central to the research but not yet described in published literature, software must be made available to editors and reviewers. We strongly encourage code deposition in a community repository (e.g. GitHub). See the Nature Portfolio guidelines for submitting code & software for further information.

Data

Policy information about availability of data

All manuscripts must include a data availability statement. This statement should provide the following information, where applicable:

- Accession codes, unique identifiers, or web links for publicly available datasets
- A description of any restrictions on data availability
- For clinical datasets or third party data, please ensure that the statement adheres to our policy

The data that support the findings from all the studies are publicly available via the Open Science Framework (OSF) using the following link: https://osf.io/2ntdy/?view_only=2cacc7b1cf2141cf8c343f3ee28dab1d

Human research participants

Policy information about studies involving human research participants and Sex and Gender in Research.

Reporting on sex and gender

Findings apply to both male and female gender. Participants also had the option to identify themselves using a different gender label, although few of them selected that option. Gender was assessed in each study using self-reports; Table 1 includes the breakdown of gender and other demographic information for all studies. In a nutshell, as can be seen from Table 1, the number of males and females who participated in each study was similar. Overall, 5283 female and 4965 male participants completed the studies, whereas 4832 female and 4392 male participants were included in analyses. Moreover, 43 participants who completed the studies identified themselves as “Other”, whereas 42 participants who were included in analyses identified themselves as “Other”. Finally, the data for 11 participants who completed the studies, and 8 participants who were included in analyses, were missing or were not disclosed.

Population characteristics

See above.

Recruitment

The information regarding how participants were recruited is summarized in Table 1 in the article. In Studies 1 (Sample 1), 4 (Samples 1 and 2), 5 (Samples 1 and 2), and 6, participants were recruited via Pureprofile (https://www.pureprofile.com/). In Studies 1 (Sample 2), 2, and 3, participants were recruited via Amazon Mechanical Turk (https://www.mturk.com/). In Study 7, participants were recruited via Prolific (https://www.prolific.co/). Therefore, all data were collected using online participant panels. It is possible that such panels attract specific types of participants, and that certain self-selection biases might have been present (e.g., individuals who are more confident with technology being more likely to participate). We aimed to minimize any potential impact of such biases on our findings by measuring various relevant variables and using them in statistical analyses. For example, one of the covariates we used in the machine learning models (Study 6; see Supplementary Tables 11-12) was a variable indicative of technological proficiency involving robots (i.e., people’s previous frequency of interaction with robots). It is also important to emphasize that these panels generally contain more diverse (Buhrmester, Kwang, & Gosling, 2011; Buhrmester, Talaifar, & Gosling, 2018; Casler, Bickel, & Hackett, 2013) and more attentive participants than typical university research pools (Hauser & Schwarz, 2016) and are widely used in psychological and behavioural sciences research. Moreover, it is not a given that other modes of recruitment (e.g., participant pools of university research labs) would avoid technological proficiency as a potential bias of online recruitment panels, considering that research participation is often advertised online and participants such as students tend to use technology for their studies.

Ethics oversight

This research complies with the ethics policy and procedures of the London School of Economics and Political Science and has also been approved by its Research Ethics Committee (ref. 20810).

Note that full information on the approval of the study protocol must also be provided in the manuscript.

Field-specific reporting

Please select the one below that is the best fit for your research. If you are not sure, read the appropriate sections before making your selection.

☐ Life sciences ☒ Behavioural & social sciences ☐ Ecological, evolutionary & environmental sciences

For a reference copy of the document with all sections, see nature.com/documents/nr-reporting-summary-flat.pdf

Behavioural & social sciences study design

All studies must disclose on these points even when the disclosure is negative.

Study description	- Study 1: Developing a definition of robots based on how participants perceive them. This study employed mixed-methods and therefore produced both qualitative and quantitative data. Two different samples of participants were tested. In Sample 1, participants were asked to generate as many characteristics of robots as possible. Then, we recruited Sample 2 and asked them to group the characteristics identified by the previous sample into common categories. Using hierarchical cluster analysis (Kaufman & Rousseeuw, 2005; Nielsen, 2016; Šulc & Řezanková, 2019), we then identified the main clusters that comprise the robot characteristics and used them to construct the robot definition.<br><br>- Study 2: Identifying all domains of human functioning in which robots operate. This study was qualitative and therefore produced qualitative data. In the study, we used the robot definition developed in Study 1 to identify a comprehensive list of all domains of human functioning in which robots can be encountered. Participants were presented with the definition and asked to generate all such domains they could think of. To develop an extensive inventory of domains, we analyzed their responses using inductive content analysis (Elo & Kyngäs, 2008; Elo et al., 2014; Hsieh & Shannon, 2005; Mayring, 2004; Vaismoradi, Turunen, & Bondas, 2013).<br><br>- Study 3: Mapping the content space of psychological processes toward robots. This study was qualitative and therefore produced qualitative data. In this study, the aim was to identify a comprehensive range of psychological processes regarding robots. Participants were asked to write about any feelings, thoughts, and behaviors they could think of in relation to robots from the domains developed in Study 2. Their responses were analyzed using iterative categorization (Neale, 2016) to generate the final list of psychological processes.<br><br>- Study 4: Establishing dimensions of the psychological processes. This was a quantitative study (i.e., it used a cross-sectional, correlational design) and therefore produced quantitative data. Two different samples of participants were tested. We randomly allocated participants from each sample to an example of a robot from one of the 28 domains established in Study 2 and asked them to answer questions measuring each of the 149 psychological processes established in Study 3 in relation to this specific robot. To identify the dimensions, the data were analyzed using exploratory factor analyses (EFAs; Schmitt, 2011).<br><br>- Study 5: Confirming the dimensions of the psychological processes. This was a quantitative study (i.e., it used a cross-sectional, correlational design) and therefore produced quantitative data. We tested two samples to confirm the dimensions established in Study 4 using exploratory structural equation modeling (ESEM; Asparouhov & Muthén, 2009).<br><br>- Study 6: Determining the main individual difference predictors of the dimensions confirmed in Study 5. This was a quantitative study (i.e., it used a cross-sectional, correlational design) and therefore produced quantitative data. To select the most predictive individual differences from the ones we tested, we employed a range of commonly used machine learning algorithms (e.g., lasso, random forests; Helwig, 2017; Jacobucci, Brandmaier, & Kievit, 2019; Joel et al., 2020; Kuhn, 2008, 2022) in combination with k-fold cross validation (de Rooij & Weeda, 2020).<br><br>- Study 7: Confirming the predictors and establishing the mechanism. This was a quantitative study (i.e., it used a longitudinal, correlational design in 2 waves) and therefore produced quantitative data. In this study, we aimed to explain the relationship between the most predictive individual differences from Study 6 and the dimensions of psychological responses regarding robots identified in Studies 4 and 5. The study therefore consisted of two waves. In wave 1, we measured the individual differences, and in wave 2 we first assessed a range of potential mediators and then asked participants to answer the items measuring the dimensions. Linear regressions and linear regression-based mediation analyses (Hayes, 2018) were used to analyze the data.
Research sample	As indicated under "Recruitment" (see the "Human research participants" section), the information regarding how participants were recruited is summarized in Table 1 in the manuscript. In Studies 1 (Sample 1), 4 (Samples 1 and 2), 5 (Samples 1 and 2), and 6, participants were recruited via Pureprofile (https://www.pureprofile.com/). In Studies 1 (Sample 2), 2, and 3, participants were recruited via Amazon Mechanical Turk (https://www.mturk.com/). In Study 7, participants were recruited via Prolific (https://www.prolific.co/). Therefore, all data were collected using online participant panels. In Studies 1 (Sample 1), 4 (Sample 1), and 5 (Sample 1) participants were UK adults, and in Studies 1 (Sample 2), 2, 3, 4 (Sample 2), 5 (Sample 2), 6, and 7 participants were US adults. Participants in Studies 4-6 were recruited to be reasonably representative of the UK/US populations in terms of age, gender, and geographical region, whereas for Study 1 (Sample 1) the focus was on gender only. Supplementary Tables 1-2 contain more comprehensive breakdowns of these variables, the criteria that were used to guide representative sampling, and various demographic characteristics. We targeted specifically UK and US samples because the type of online panels we used to recruit participants are typically able to provide large and in some cases reasonably representative samples from these countries, which can be more difficult when it comes to recruiting participant from other countries. As stated in the Discussion section of the present article when discussing the limitations, since our research proposed and investigated a construct (i.e., psychological processes regarding robots) from scratch, our priority was to establish its foundations, and combining the investigation of cultural differences with this agenda using equally meticulous methods would have exceeded the scope of a single article.<br><br>Overall, Table 1 in the article provides basic demographic information for our participants, whereas Supplementary Tables 1 and 2 contain more comprehensive information in this regard. Below we present mean age, standard deviation of age, and the number of female, male, other, and undisclosed participants who completed each study (see Table 1 in the article).<br>- Study 1 (Sample 1): 49.496, 13.598, 132, 133, 1, 0<br>- Study 1 (Sample 2): 36.510, 10.566, 42, 58, 0, 0<br>- Study 2: 36.257, 10.270, 31, 39, 0, 0<br>- Study 3: 40.693, 12.194, 193, 153, 1, 3<br>- Study 4 (Sample 1): 47.932, 16.611, 852, 812, 4, 0

- Study 4 (Sample 2): 48.004, 16.772, 976, 830, 2, 0
- Study 5 (Sample 1): 46.648, 16.616, 590, 601, 6, 3
- Study 5 (Sample 2): 46.656, 16.914, 616, 598, 5, 0
- Study 6: 47.405, 17.262, 1299, 1186, 15, 5
- Study 7: 42.910, 13.535, 552, 555, 9, 0

Sampling strategy

As indicated above, participants were recruited via online panels commonly used in psychological and behavioural research (Prolific, Pureprofile, and Amazon Mechanical Turk). These and other online participant panels generally use some form of convenience sampling (e.g., Chandler & Shapiro 2016; Armitage & Eerola, 2020; see also https://researcher-help.prolific.co/hc/en-gb/articles/360009223133-Is-online-crowdsourcing-a-legitimate-alternative-to-lab-based-research-), and the sampling strategy used in the present research was therefore convenience sampling. More information about the composition of our participant samples is provided in the section “Research sample” above.

In the Methods section for each study in the article, there is a section on "Sample size" that explains how the sample size was predetermined (see also Supplementary Methods). For studies that had qualitative elements (Study 1, Sample 1; Study 2; and Study 3), we recruited sample sizes larger than 50 participants, given that simulations have indicated that sample sizes larger than 30-50 participants (Mayring, 2019; van Rijnsoever, 2017) tend to reach the point of data saturation, which implies that adding new participants beyond this number produces very little new information (Faulkner & Trotter, 2017). For Study 1 (Sample 2; see section “Sample Size” for that study in the article), in which we used hierarchical cluster analysis, the sample size was based on recent simulations, according to which the most important determinant of power seems to be the number of observations per cluster, with 20 observations yielding sufficient power to detect a cluster (Dalmaijer et al., 2022). For Study 4 (see section “Sample Size” for that study in the article), we consulted several resources to determine the number of participants to test for each sample because there is no consensus regarding sample size requirements for EFA (Costello & Osborne, 2005; Hogarty, Hines, Kromrey, Ferron, & Mumford, 2005; Kyriazos, 2018; MacCallum, Widaman, Zhang, & Hong, 1999; Reio Jr & Shuck, 2015). First, a few resources posit that the ratio of the number of participants to the number of items should be at least 10:1 (Everitt, 1975; Gorsuch, 1983; Reio Jr & Shuck, 2015). Second, some studies estimated that, if the ratio of the number of items to the number of factors is larger than 10:3, recruiting approximately 400 participants leads to high power, even under low communalities (MacCallum et al., 1999). Third, it has been proposed that a sample size larger than 300 is sufficient for a wide range of factor solutions (Dimitrov, 2012; Guadagnoli & Velicer, 1988). Our sample sizes for Study 4 met all these criteria. For Study 5, we determined the number of participants to test using Monte Carlo simulations (Muthén & Muthén, 2002) based on the data from Samples 1 and 2 (Study 4). Concerning Study 6, there are no clear guidelines for the use of machine learning algorithms combined with cross-validation regarding sample size and power. In a series of simulations, Song, Tang, and Wee (2021) showed that, for 10-fold cross-validations that we were planning to use, a sample size of 2000 leads to high generalizability (i.e., likelihood that the results will apply to other samples from the same population) without inflating time taken to run the models. We therefore aimed to recruit a sample that would result in roughly 2200 participants after applying the exclusion criteria, in case of any additional missing data. Finally, we determined the sample size for Study 7 by computing a-priori power analyses (Faul, Erdfelder, Buchner, & Lang, 2009) based on the data from Study 6.

Data collection

Qualtrics (https://www.qualtrics.com/) was used to collect the data (for the versions of Qualtrics that were used, see the "Data collection" field above). This is an online survey software widely used by universities across the world. Participants were anonymous and completed the study in their own surroundings. Participation was allowed on PCs, laptops, and tablets, but not on mobile phones. The researchers (i.e., authors of this paper) were not blinded to study predictions and aims. However, since the participants were anonymous and there was no contact between the researchers and participants, it is implausible that experimenter demand effects played a role in the present research. Importantly, since the present research used a data-driven approach as described in the Introduction section of the article, the majority of studies did not have a priori predictions. Only Study 5, in which we aimed to confirm the dimensions of psychological processes established in Study 4, and Study 7, in which we aimed to corroborate the main individual difference predictors identified in Study 6, were confirmatory. This is another reason why experimenter demand effects concerning study predictions were unlikely to play a role in the present research.

Timing

Start and stop dates for data collection in each study:
- Study 1 (Sample 1): 27 February – 4 March 2019
- Study 1 (Sample 2): 26 July – 27 July 2019
- Study 2: 24 September 2019
- Study 3: 21 October – 24 October 2019
- Study 4 (Sample 1): 4 March – 11 March 2021
- Study 4 (Sample 2): 8 April – 24 April 2021
- Study 5 (Sample 1): 29 June – 3 July 2021
- Study 5 (Sample 2): 24 June – 2 July 2021
- Study 6: 23 September – 15 October 2021
- Study 7: 13 December 2021

Data exclusions

The exclusion criteria were pre-established (e.g., see pre-registration for Study 7: https://osf.io/nejvm?view_only=79b6eeee42e24cb2a977927712bdcdd2). They are comprehensively described in the Methods section in the article and in Supplementary Methods. In general, participants were excluded from analyses if they did not correctly answer seriousness checks (Aust, Diedenhofen, Ullrich, & Musch, 2013), instructed-response items (Kung, Kwok, & Brown, 2018; Meade & Craig, 2012; Thomas & Clifford, 2017), and understanding checks in which they were asked to identify the main topic of the study amongst a range of dummy topics. Table 1 in the article summarizes participants who completed the study and who were included in analyses after the exclusion criteria were applied. From the participants who completed the study, the following number of participants were excluded from data analyses:
- Study 1 (Sample 1): 42
- Study 1 (Sample 2): 5
- Study 2: 3

- Study 3: 16
- Study 4 (Sample 1): 140
- Study 4 (Sample 2): 271
- Study 5 (Sample 1): 93
- Study 5 (Sample 2): 111
- Study 6: 302
- Study 7: 45

Non-participation	Considering that participation in the present research took place anonymously and online, we only have knowledge of participants who completed the study (see Table 1 in the article). In some cases, online participants recruited via the online panels we used (Prolific, Pureprofile, and Amazon Mechanical Turk) test the survey and answer one or few questions and then leave - these data are captured under incomplete data but we are not aware of whether and how many of these participants are unique participants. Overall, non-participation data for the present research are not available.
Randomization	As can be seen under "Study description", the present research was not experimental. Therefore, there were no different conditions to which participants could be randomized. However, it is important to emphasize that in Studies 4-7, in which participants were allocated to robot examples from 28 possible robot domains, this allocation was random.

Reporting for specific materials, systems and methods

We require information from authors about some types of materials, experimental systems and methods used in many studies. Here, indicate whether each material, system or method listed is relevant to your study. If you are not sure if a list item applies to your research, read the appropriate section before selecting a response.

Materials & experimental systems

n/a	Involved in the study
☒	☐ Antibodies
☒	☐ Eukaryotic cell lines
☒	☐ Palaeontology and archaeology
☒	☐ Animals and other organisms
☒	☐ Clinical data
☒	☐ Dual use research of concern

Methods

n/a	Involved in the study
☒	☐ ChIP-seq
☒	☐ Flow cytometry
☒	☐ MRI-based neuroimaging
